# Supplementary material for: Berberine bridge enzyme-like oxidase-catalysed double bond isomerization acts as the pathway switch in cytochalasin synthesis
Source: Nat Commun. 2022 Jan 11;13:225. doi: 10.1038/s41467-021-27931-z (PMC8752850; doi:10.1038/s41467-021-27931-z)
Supplement: Supplementary file 1 — Supplementary Information [file 41467_2021_27931_MOESM1_ESM.pdf]

Supplementary Information

Berberine bridge enzyme-like oxidase-catalysed double bond isomerization acts as the pathway switch in cytochalasin synthesis

Jin-Mei Zhang[1,3], Xuan Liu[1,3], Qian Wei[1], Chuanteng Ma[2], Dehai Li[2], and Yi Zou[*,1]

[1] College of Pharmaceutical Sciences, Southwest University, Chongqing 400715, P. R. China

[2] Key Laboratory of Marine Drugs, Chinese Ministry of Education, School of Medicine and Pharmacy, Ocean University of China, Qingdao 266003, P. R. China

[3] These authors contributed equally: Jin-Mei Zhang, Xuan Liu.

* E-mail: zouyi31@swu.edu.cn

Table of contents

1. Supplementary Methods 7

1.1 Strains and culture conditions 7

1.2 Plasmid construction 7

1.3 Bioinformatics analysis 9

1.4 The protein expression of AspoA in *E. coli* 9

1.5 Measurement of the kinetic parameters of AspoD toward **12** and **11** 10

1.6 The in vitro biochemical assay of AspoA in D_2O buffer 10

1.7 The nonenzymatic reactions in D_2O buffer. 10

1.8 Purification and structural characterization of compounds 10

2. Supplementary Tables 14

Supplementary Table 1. Stains or plasmids used in this study. 14

Supplementary Table 2. Primers used in this study. 15

Supplementary Table 3. Recombinant plasmids used in this study 17

Supplementary Table 4. NMR data of compound **1** in $CDCl_3$. 18

Supplementary Table 5. NMR data of compound **2** in $CDCl_3$. 19

Supplementary Table 6. NMR data of compound **3** in $CDCl_3$. 20

Supplementary Table 7. NMR data of compound **6** in $CDCl_3$. 22

Supplementary Table 8. NMR data of compound **7** in DMSO-d_6. 23

Supplementary Table 9. NMR data of compound **8** in DMSO-d_6. 24

Supplementary Table 10. NMR data of compound **11** in $CDCl_3$. 25

Supplementary Table 11. NMR data of compound **12** in $CDCl_3$. 26

Supplementary Table 12. NMR data of compound **14** in $CDCl_3$. 27

3. Supplementary Figures 28

Supplementary Figure 1. Schematic diagram of plasmids used in this study. 28

Supplementary Figure 2. Identified CYT biosynthetic gene clusters containing aliphatic amino acid-type and aromatic amino acid-type clusters. 29

Supplementary Figure 3. Previously unsuccessful examples that reconstitution of aromatic ammino acid-type *cyt* BGCs in heterologous hosts. 30

Supplementary Figure 4. Representative aromatic amino acid-type and aliphatic amino acid-type cytochalasans isolated from *A. flavipes* KLA03. 31

Supplementary Figure 5. Comparison of *cyto* (cluster 1) gene cluster with *ccs* gene cluster (**a**) and A domain codes predicted for substrate recognition of PKS-NRPS proteins in *cyt* BGCs (**b**)...32

Supplementary Figure 6. Comparison of *aspo* (cluster 2) gene cluster with *ffs* cluster and *phm* cluster (**a**) and A domain codes predicted for substrate recognition of PKS-NRPS proteins in three gene clusters (**b**)..33

Supplementary Figure 7. a The isotope chemical feeding assays in *AN-aspoEH* and *AN-aspoEHB* showed that the [1, 2-^{13}C]-L-leucine was introduced into **3** and **6** respectively. The molecular weight of **3** and **6** could increase 2 amu. **b** The UV absorption spectrum of compound **3** and **6**...34

Supplementary Figure 8. Compounds **7** and **8** were converted into **2** and **1** in $CDCl_3$ after 48 h. 35

Supplementary Figure 9. Characterization and analyses of BBE-like oxidases.................37

Supplementary Figure 10. SDS-PAGE analyses of the heterogeneously expressed proteins in this study...39

Supplementary Figure 11. The cell free bioconversion assays of compound **7** or **8** to **11** or **12**. 40

Supplementary Figure 12. The overall structure of the template MtVAO615 (cyan, PDB: 6F72) and model of AspoA (green). 41

Supplementary Figure 13. The proposed mechanism that AspoA uses Tyr_{160} as the base to abstract the C_{18} hydrogen. 42

Supplementary Figure 14. The AspoA-catalysed conversion of **7** to **11** in D_2O buffer......43

Supplementary Figure 15. The spontaneous conversion of **7** to **2** in D_2O buffer...............44

Supplementary Figure 16. The kinetic parameters of AspoD toward **12** (**a**) and **11** (**b**).....45

Supplementary Figure 17. UV absorption and HRMS spectrum (positive ionization) of compound **1**..46

Supplementary Figure 18. UV absorption and HRMS spectrum (positive ionization) of compound **2**......46

Supplementary Figure 19. UV absorption and HRMS spectrum (negative ionization) of compound **3**......47

Supplementary Figure 20. UV absorption and HRMS spectrum (positive ionization) of compound **6**......47

Supplementary Figure 21. UV absorption and HRMS spectrum (positive ionization) of compound **7**......48

Supplementary Figure 22. UV absorption and HRMS spectrum (positive ionization) of compound **8**......48

Supplementary Figure 23. UV absorption and HRMS spectrum (positive ionization) of compound **9**......49

Supplementary Figure 24. UV absorption and HRMS spectrum (positive ionization) of compound **10**......49

Supplementary Figure 25. UV absorption and HRMS spectrum (positive ionization) of compound **11**......50

Supplementary Figure 26 UV absorption and HRMS spectrum (positive ionization) of compound **12**......50

Supplementary Figure 27. UV absorption and HRMS spectrum (positive ionization) of compound **14**......51

Supplementary Figure 28. HRMS spectrum (positive ionization) of compound ^{2}H-**2**. 52

Supplementary Figure 29. HRMS spectrum (positive ionization) of compound ^{2}H-**11**. 52

Supplementary Figure 30. ^{1}H NMR spectrum of compound **1** in $CDCl_3$ (400 MHz).........53

Supplementary Figure 31. ^{13}C NMR spectrum of compound **1** in $CDCl_3$ (100 MHz)........54

Supplementary Figure 32. DEPT-135° spectrum of compound **1** in $CDCl_3$......................55

Supplementary Figure 33. HSQC spectrum of compound **1** in $CDCl_3$..............................56

Supplementary Figure 34. HMBC spectrum of compound **1** in $CDCl_3$..............................57

Supplementary Figure 35. ^{1}H-^{1}H COSY spectrum of compound **1** in $CDCl_3$. 58

Supplementary Figure 36. ^1H-^1H NOESY spectrum of compound **1** in $CDCl_3$.................59

Supplementary Figure 37. ^1H NMR spectrum of compound **2** in $CDCl_3$ (400 MHz).........60

Supplementary Figure 38. ^{13}C NMR spectrum of compound **2** in $CDCl_3$ (100 MHz)........61

Supplementary Figure 39. DEPT-135° spectrum of compound **2** in $CDCl_3$......................62

Supplementary Figure 40. HSQC spectrum of compound **2** in $CDCl_3$..............................63

Supplementary Figure 41. HMBC spectrum of compound **2** in $CDCl_3$.............................64

Supplementary Figure 42. ^1H-^1H COSY spectrum of compound **2** in $CDCl_3$. 65

Supplementary Figure 43. ^1H-^1H NOESY spectrum of compound **2** in $CDCl_3$.................66

Supplementary Figure 44. ^1H NMR spectrum of compound **3** in $CDCl_3$ (400 MHz).........67

Supplementary Figure 45. ^{13}C NMR spectrum of compound **3** in $CDCl_3$ (100 MHz)........68

Supplementary Figure 46. DEPT-135° spectrum of compound **3** in $CDCl_3$......................69

Supplementary Figure 47 HSQC spectrum of compound **3** in $CDCl_3$...............................70

Supplementary Figure 48. HMBC spectrum of compound **3** in $CDCl_3$.............................71

Supplementary Figure 49. ^1H-^1H COSY spectrum of compound **3** in $CDCl_3$. 72

Supplementary Figure 50. ^1H NMR spectrum of compound **6** in $CDCl_3$ (400 MHz).........73

Supplementary Figure 51. ^{13}C NMR spectrum of compound **6** in $CDCl_3$ (100 MHz)........74

Supplementary Figure 52. DEPT-135° spectrum of compound **6** in $CDCl_3$......................75

Supplementary Figure 53. HSQC spectrum of compound **6** in $CDCl_3$..............................76

Supplementary Figure 54. HMBC spectrum of compound **6** in $CDCl_3$.............................77

Supplementary Figure 55. ^1H-^1H COSY spectrum of compound **6** in $CDCl_3$. 78

Supplementary Figure 56. ^1H-^1H NOESY spectrum of compound **6** in $CDCl_3$.................79

Supplementary Figure 57. ^1H NMR spectrum of compound **7** in DMSO-d_6 (400 MHz). ..80

Supplementary Figure 58. ^{13}C NMR spectrum of compound **7** in DMSO-d_6 (100 MHz). .81

Supplementary Figure 59. DEPT-135° spectrum of compound **7** in DMSO-d_6. 82

Supplementary Figure 60. HSQC spectrum of compound **7** in DMSO-d_6..........................83

Supplementary Figure 61. HMBC spectrum of compound **7** in DMSO-d_6. 84

Supplementary Figure 62. ^1H-^1H COSY spectrum of compound **7** in DMSO-d_6..............85

Supplementary Figure 63. ^1H-^1H NOESY spectrum of compound **7** in DMSO-d_6. 86

Supplementary Figure 64. ^{1}H NMR spectrum of compound **8** in DMSO-d_6 (400 MHz). ..87

Supplementary Figure 65. ^{13}C NMR spectrum of compound **8** in DMSO-d_6 (100 MHz). .88

Supplementary Figure 66. DEPT-135° spectrum of compound **8** in DMSO-d_6. 89

Supplementary Figure 67. HSQC spectrum of compound **8** in DMSO-d_6..........................90

Supplementary Figure 68. HMBC spectrum of compound **8** in DMSO-d_6. 91

Supplementary Figure 69. ^{1}H-^{1}H COSY spectrum of compound **8** in DMSO-d_6..............92

Supplementary Figure 70. ^{1}H-^{1}H NOESY spectrum of compound **8** in DMSO-d_6. 93

Supplementary Figure 71. ^{1}H NMR spectrum of compound **11** in $CDCl_3$ (400 MHz).......94

Supplementary Figure 72. ^{13}C NMR spectrum of compound **11** in $CDCl_3$ (100 MHz)......95

Supplementary Figure 73. DEPT-135° spectrum of compound **11** in $CDCl_3$.....................96

Supplementary Figure 74. HSQC spectrum of compound **11** in $CDCl_3$. 97

Supplementary Figure 75. HMBC spectrum of compound **11** in $CDCl_3$. 98

Supplementary Figure 76. ^{1}H-^{1}H COSY spectrum of compound **11** in $CDCl_3$. 99

Supplementary Figure 77. ^{1}H-^{1}H NOESY spectrum of compound **11** in $CDCl_3$.............100

Supplementary Figure 78. ^{1}H NMR spectrum of compound **12** in $CDCl_3$ (400 MHz).....101

Supplementary Figure 79. ^{13}C NMR spectrum of compound **12** in $CDCl_3$ (100 MHz)....102

Supplementary Figure 80. HSQC spectrum of compound **12** in $CDCl_3$. 103

Supplementary Figure 81 . HMBC spectrum of compound **12** in $CDCl_3$. 104

Supplementary Figure 82. ^{1}H-^{1}H COSY spectrum of compound **12** in $CDCl_3$. 105

Supplementary Figure 83. ^{1}H-^{1}H NOESY spectrum of compound **12** in $CDCl_3$.............106

Supplementary Figure 84. ^{1}H NMR spectrum of compound **14** in $CDCl_3$ (400 MHz).....107

Supplementary Figure 85. ^{13}C NMR spectrum of compound **14** in $CDCl_3$ (100 MHz)....108

Supplementary Figure 86. DEPT-135° spectrum of compound **14** in $CDCl_3$...................109

Supplementary Figure 87. HSQC spectrum of compound **14** in $CDCl_3$. 110

Supplementary Figure 88. HMBC spectrum of compound **14** in $CDCl_3$. 111

Supplementary Figure 89. ^{1}H-^{1}H COSY spectrum of compound **14** in $CDCl_3$. 112

Supplementary Figure 90. ^{1}H-^{1}H NOESY spectrum of compound **14** in $CDCl_3$.............113

Sequence information 114

Supplementary references 128

1. Supplementary Methods

1.1 Strains and culture conditions

Aspergillus flavipes KLA03 was maintained on PDA medium (26 g/L Potato Dextrose Water, 20 g/L agar) at 25°C, 6 days for sporulation or culture at 25°C on PDB medium for 4 days to extract genome DNA (gDNA) and complementary DNA (cDNA). *Aspergillus nidulans* LO8030 was used as the host for heterologous expression the *aspo* gene cluster. *A. nidulans* LO8030 was culture at 37°C for 3-4 days on solid CD medium (10 g/L glucose, 50 mL/L 20 × nitrate salts, 1 mL/L trace elements, 20 g/L agar)) for sporulation or at 25 °C, 3 days on solid CD-ST medium (20 g/L starch, 10 g/L casein hydrolysate (acid), 50 mL/L nitrate salts, 1 mL/L trace elements, 20 g/L Agar) for heterologous expression and compounds production. *Saccharomyces cerevisiae* strain BJ5464-NpgA was used as the yeast cell free expression host of the *aspoA* or for heterologous recombination to construct the *A. nidulans* overexpression plasmids and was grown at 28 °C on yeast peptone dextrose medium (20 g/L glucose, 20 g/L tryptone, 10 g/L yeast extract). *Escherichia coli* BL21 was used for protein expression of the *aspoA* and *aspoD. E. coli* strain XL-1 was used for cloning. All *E. coli* strain was culture at 37 °C for grown or 16 °C and 25 °C for protein expression.

1.2 Plasmid construction

The primers used in this study are listed in **Supplementary Table 1**. The plasmids are summarized in **Supplementary Table 2**. Plasmid maps are shown **Supplementary Fig. 1**.

To construct expression plasmids of *aspoA,B,C,E,F and H* genes for *Aspergillus nidulans,* each gene with its terminator (~500 bp) was amplified from genomic DNA extract from the *Aspergillus flavipes* KLA03. The expression plasmids were gained through yeast homologous recombination in *S. cerevisiae* BJ5464-NpgA. The *glaA*, *gpdA*, *amyB* promoters were amplified from vectors pANU, pANR, pANP by using primer pairs *glaA*-F/*glaA*-R, *gpdA*-F/*gpdA*-R and *amyB*-F/*amyB*-R, respectively. Plasmid pANU was digested with *Not* I and plasmids pANR, pANP was digested with *BamH*I to use as vectors for insert genes. *AspoE* was amplified by PCR using primer pairs pANR-*aspoE*-F1/pANR-*aspoE*-R1, pANR-*aspoE*-F2/pANR-*aspoE*-R2, pANR-*aspoE*-F3/pANR-*aspoE*-R3, pANR-*aspoE*-F4/pANR-*aspoE*-R4 and and was cloned into vector pANR yielding plasmid pIM 8001. *AspoH* was amplified by PCR using primer pairs pANU-*aspoH*-F/pANU-*aspoH*-ZR and was cloned into vector pANU with *glaA* promoter to yield plasmid pIM 8002. Plasmid pIM 8006 was gained by same method. *AspoF* was amplified by PCR using primer pairs pANF-*aspoF*-F/pANU-

aspoF-ZR and was cloned into vector pANP with *AmyB* promoter. To construct plasmid 8003, genes *aspoH* and *aspoB* fragments were gained by PCR using the primer pairs pANU-*aspoH*-F/ U-*aspoH+B*-CR and pANU-*aspoB*-F/pANU-*aspoB*-R and cloned into vector pANU. *AspoH, aspo*C and aspoB were amplified by PCR using primer pairs pANU-*aspoH*-F/pANU-*aspoH*-R, pANU-*aspoC*-F/pANU-*aspoC*-R pANU-*aspoB*-F/pANU-*aspoB*-R and was cloned into vector pANU to yield plasmid pIM 8004. The above genes *aspoH* and *aspoC* fragments and *cytoF* fragment that was was amplified by PCR using primer pairs pANU-*cytoF*-F/pANU- *cytoF*-R was inserted into pANU to obtain plasmid 8005. The primer pairs pANP-*aspoF*-F/pANU-*aspoF*-R, pANP-*aspoA*-F/pANP-*aspoA*-R was used to amplify gene *aspoF* and *aspoA*, which the fragments were cloned in vector pANP yielding pIM8007. Above all plasmids, *aspoA*, *aspoC* and *aspoE* used the same promoter *gpdA*. *AspoB, aspoF* and *cytoF* used the promoter *amyB*, while promoter *glaA* was used for *aspoH*.

To express AspoA *in E. coli*, intron-free *aspoA* was amplified from the cDNA of *A. flavipes* KLA03 using primer pairs pColdI-*aspoA*-F/pColdI-*aspoA*-R, pQ8-*aspoA*-NF/ pQ8-*aspoA*-R and GST-FMO-F/GST-FMO-R, respectively. The fragments were digested with *Bam*H I-*Hind* III, *Hind* III-*Not* I or *Sma* I-*Not* I and then was ligated to the *Bam*H *I-Hind* III digested vector pColdI (a protein expression vector with His tag on N-terminus), the *Hind* III-*Not* I digested vector pQ8 (a protein expression vector with MBP tag on N-terminus) or the *Sma* I-*Not* I digested vector pGEX 4T-1 (a protein expression vector with GST tag on N-terminus) yielding plasmids pIM 8008-8010. The *aspoD* expression plasmids pIM 8011 were constructed by the same method. *AspoD* was amplified from the cDNA of *A. flavipes* KLA03 using pColdI-*aspoD*-F/pColdI-*aspoD*-R and the PCR products were digested with *Nde* I and *Kpn* I, and subsequently inserted into the *Nde* I and *Kpn* I digested pColdI.

Plasmids pIM8012-8016 were constructed to prove the function of *aspoA* and to confirm the crucial catalytic residues. AspoA was amplified from the cDNA of *A. flavipes* KLA03 using primer pairs pYEU-*aspoA*-F/pYEU-*aspoA*-R and insert into vector pYEU through yeast homologous recombination in *S. cerevisiae* BJ5464-NpgA to obtain plasmids pIM8012. The corresponding mutated overlapping fragments were amplified from the cDNA of *A. flavipes* KLA03 using primer pairs pYEU-*aspoA*-F/ pYEU-*aspoA*-H158A-R, pYEU-*aspoA*-H158A-F/pYEU-*aspoA*-R, pYEU-*aspoA*-F/ pYEU-*aspoA*-Y160A-R, pYEU-*aspoA*-Y160A-F/pYEU-*aspoA*-R, pYEU-*aspoA*-F/ pYEU-*aspoA*-E538A-R, pYEU-*aspoA*-E538A-F/pYEU-*aspoA*-R, pYEU-*aspoA*-F/ pYEU-*aspoA*-E538D-R, pYEU-*aspoA*-E538D-F/pYEU-*aspoA*-R, and then were cloned into vector pYEU by yeast homologous recombination in *S. cerevisiae* BJ5464-NpgA to generate pIM8013-8016.

All the plasmids were confirmed by restriction enzyme and DNA sequencing.

1.3 Bioinformatics analysis

The biosynthetic gene clusters for natural products of *A. flavipes* KLA03 was analyzed according to the antiSMASH fungal version online analysis. Two PKS-NRPS clusters were confirmed in this fungus. For gene cluster annotation of two PKS-NRPS, 2ndFind program was used to predict the open reading frame and intron, gene function was assigned based on BlastP search and domains of core genes were analyzed by *interpro* website. A domain codes for substrate recognition were analyzed with PKS/NRPS Analysis Web-site.

To analyze the Phylogenetic relationships of AspoA, phylogenetic tree analysis of fungal BBE-like enzyme was accomplished. The BBE-like enzyme sequences obtained from NCBI database and multiple sequences alignment were performed using ClustalW. Evolutionary analyses were conducted in MEGA7 software, and the phylogenetic tree was inferred by using neighbor-joining method. Analysis of AspoA active sites was performed using DNAMAN software. Domain of AspoA was analyzed by BlastP search of NCBI database.

Homology modeling and molecular docking of AspoA. The homology model of AspoA was constructed by SWISS-MODEL based on sequence similarity, crystal structure of MtVAO615 from *Myceliophthora thermophila* (PDB:6f72, https://www.rcsb.org/structure/6F72) was selected as a template to model the tertiary structure of AspoA. The molecular docking between AspoA model and compounds **7** or **8** were employed and analyzed by Discovery Studio (v2021, National Demonstration Center for Experimental Pharmacy Education (Southwest University)).

The amino acids sequence of AspoA was submitted into EFI-ENZYME SIMILARITY TOOL together with the fungal BBE-like enzymes from Pfam family PF08031. The SSN was generated using the UniRef90 database with alignment score of 120. The SSN was colored by Cytoscape v3.8.2.

antiSMASH: https://fungismash.secondarymetabolites.org/

2nd Find: http://biosyn.nih.go.jp/2ndFind/

NCBI BLAST: https://blast.ncbi.nlm.nih.gov/Blast.cgi

PKS/NRPS Analysis Web-site: http://nrps.igs.umaryland.edu/

interpro website: http://www.ebi.ac.uk/interpro/search/sequence/

SWISS-MODEL: https://swissmodel.expasy.org/

EFI-ENZYME SIMILARITY TOOL: https://efi.igb.illinois.edu/efi-est/

1.4 The protein expression of AspoA in *E. coli*

To obtain soluble protein of AspoA, recombinant plasmids pIM 8008-8010 were transformed into *E. coli* BL21 strain respectively. The same method as AspoD protein was used to induce protein expression. The stain containing plasmid pIM8009 was culture in LB medium with 50 μg/mL kanamycin and the stains containing plasmids pIM8008 or pIM8010 was culture in LB medium with 100 μg/mL ampicillin. GST-AspoA and MBP-AspoA proteins were induced with 0.2 mM IPTG at 25°C for 10 h and AspoA with His-tag protein were induced with 0.2 mM IPTG at 16°C for 20 h. The cells were gained by centrifugation at 4 °C, 3000 g for 5 min and resuspended in 5 mL buffer A (50 mM Tris-HCl, 500 mM NaCl, 10% glycerol, pH 7.5). Then, the cells were lysed through sonication on ice and centrifugated at 4 °C, 23000 g for 40 min to separate soluble fraction from precipitate. The precipitate was resuspended by a small amount of buffer A. The soluble and insoluble fraction were analyzed by SDS-PAGE and compared with *E. coli* BL21 to confirm soluble protein.

1.5 Measurement of the kinetic parameters of AspoD toward 12 and 11

To determine the kinetic parameters of AspoD toward **12**, 50 μl reaction mixtures containing 2 μM AspoD, 1 mM NADPH and different concentration of **12** (10, 20, 40, 100, 150 and 200 μM) were performed at pH 7.5, 25°C. To determine the kinetic parameters of AspoD toward **11**, 50 μl reaction mixtures containing 10 μM AspoD, 1 mM NADPH and different concentration of **11** (10, 20, 40, 100, 150, 400 and 600 μM) were performed at pH 7.5, 25 °C. After 3 min, 50 μl methanol was added and rigorously mixed by vortex. After centrifuge, 4 μl liquid supernatants were used for further LC-MS analysis and quantifed by a standard curve. The kinetics data were fitted to the Michaelis-Menten equation using GraphPad Prism 7.00 sofware. For each concentration of substrate, five replicates were performed.

1.6 The in vitro biochemical assay of AspoA in D_2O buffer

The AspoA yeast cells were incubated as same as the description in the method section of the maintext. The harvest cells were lysed by grinding, and the generated cellular debris were freezed drying overnight. Then the cellular debris was resuspended in D_2O buffer (95% D_2O) and the supernatant was harvested by centrifugation at 4 °C and 23000 g for 30 min. The compound **7** (100 μM) was added into the supernatant and the reaction was performed at 25 °C for 10 h. The enzymatic reaction mixtures were extracted by ethyl acetate. The extracts were evaporated to dryness, dissolved in methanol and analysed by LC-MS.

1.7 The nonenzymatic reactions in D_2O buffer.

The spontaneous reactions were performed in 100 μL pH 4 D_2O buffer, 100 μM compound **7** were added and the reactions were incubated at 25 °C for 10 h to obtain ^{2}H-**2**.

1.8 Purification and structural characterization of compounds

The compounds were separated by medium pressure liquid chromatography (MPLC) Reveleris® X2 (BUCHI, Inc) with a gradient of MeOH and H_2O at a flow rate of 25 mL/min on 80 g reversed phase silica gel (C18) following. The semi-preparation high performance liquid chromatograph (HPLC) used a Shimadzu LC-20AR Prominence UFLC system and a YMC ODS-A 5 μm 120A (10×250 mm) column was used at a flow

rate of 2.5 mL/min. MCI Column chromatography was performed on MCI gel CHP 20P/P120 (37-75 μm, Mitsubishi Chemical Corporation, Japan).

Isolation of compounds 3 and 6

For isolation of **3**, the *AN-aspoEH* transformant was cultured in 10 L solid CD-ST medium at 25 °C for 3 days and the cultures were extracted with ethyl acetate three times. The organic solvent was evaporated to dryness under vacuum to obtain the crude extract. The crude extract was subjected to silica gel column using n-hexane/EtOAc (1:0, 95:5, 9:1, 85:15, 8:2) as eluents to obtain five fractions. Fraction 3 (n-hexane/EtOAc, 9:1) was isolated by semi-preparative HPLC eluded with 95% MeOH-5%H_2O to gain compound **3** (2.3 mg, t_R=27 min).

The *AN-aspoEHB* transformant was cultured 8 L solid CD-ST medium at 25 °C for 3 days and extracted as described above for compound **3**. Purification was also done similarly by fractionation over silica flash chromatography followed by semi-preparative HPLC. Compound **6** was found in fraction 5 (*n*-hexane/EtOAc, 8:2). The fraction 5 was purified by semi-preparative HPLC with an isocratic concentration 90% MeOH-10% H_2O to yield **6** (2.1 mg, t_R=18 min).

Isolation of compounds 7, 8 and 1, 2

Compounds **7** and **8** was produced by *AN-aspoEHBCF* in 8 L CD-ST agar medium at 25 °C for 3 days. The same as above method was used to obtain residue. The residues were purified by MPLC with a linear gradient of 40% to 100% MeOH in H_2O over 50 min at 25 mL/min. The fraction containing compound **7** was purified by semi-preparative HPLC using isocratic program of 80% MeOH-20% H_2O to afford compound **7** (10 mg, t_R=21 min). The compounds **8** (8 mg, t_R=14 min) was gained by semi-preparative HPLC eluted with 60% MeCN-40% H_2O. Compounds **1** and **2** were got from the conversion of **8** and **7** in $CDCl_3$, respectively.

Isolation of compounds 11 and 12

Compounds **11** and **12** were purified from the extracts of *AN-aspoEHBCFA* in 8 L solid CD-ST medium at 25 °C for 3 days. The culture was treated with the above same methods. The crude extracts were initially separated by MCI column chromatographic with different ratio of MeOH/H_2O (6:4, 7:3, 8:2, 9:1, 95:5,1:0) as the eluting solvent system. The compound **11** and **12** was separately concentrated in 80% MeOH-20% H_2O and 95% MeOH-5% H_2O through the analysis of LC-MS. The fraction containing compound **11** were further purified by semi-preparative HPLC with an isocratic concentration 55% MeCN-45% H_2O to yield **11** (3.6 mg, t_R=40 min) and **12** (5 mg,

t_R=21 min) was gained by semi-preparative HPLC using an isocratic program 75% MeOH-25% H_2O.

Isolation of compound 14

To purify **14** for structural analysis, AspoD protein was purified from 2 L cultures of *E. coli* BL21 containing plasmid pIM 8011. The conversion assay was carried out in 10 ml buffer C (50 mM Tris-HCl, 50 mM NaCl, 5% glycerol, pH 7.5), consist of 10 mg compound **12** and 25 mg NADPH. The reaction was incubated at 25 °C for 8 h and extracted with ethyl acetate. The extracted ethyl acetate layer was evaporated to dryness and isolated by semi-preparative HPLC eluted with 77 % MeOH-23% H_2O to afford compound **14** (6 mg, t_R=19 min).

Isolation of compound ^{2}H-11

To purify ^{2}H-**11**, cell free conversion of **7** by AspoA was performed in D_2O buffer. The cells from 8 L cultures of *S. cerevisiae* containing plasmid pIM 8012 were lysed by grinding and the generated cellular debris were freezed drying overnight. Then the cellular debris were resuspended in 200 ml D_2O buffer (95% D_2O). The supernatant was harvested by centrifugation at 4 °C and 23000 g for 30 min. 10 mg compound **7** was added into the supernatant and the reaction was performed at 25 °C for 10 h. The enzymatic reaction mixtures were extracted by ethyl acetate. The extracts were evaporated to dryness, dissolved in methanol and isolated by semi-preparative HPLC eluted with 73 % MeOH-23% H_2O to afford ^{2}H-**11** (0.8 mg, t_R=19 min). The compound ^{2}H-**11** was analysed by LCMS and ^{1}H-NMR.

Isolation of compound ^{2}H-2

To purify ^{2}H-**2**, complete spontaneous conversion of **7** to **2** was performed in 50 mL pH 4 D_2O buffer at 25 °C for 20 h. The reaction mixtures were then extracted by ethyl acetate. The extracts were evaporated to dryness and was analysed by LCMS and ^{1}H-NMR.

HRMS data of compound **3** was performed on Fourier-transform ion cyclotron resonance-mass spectrometry (FT-ICR-MS) (Bruker SolariII, Bremen, Germany) at Chongqing University. HRMS data of other compounds were carried out on quadrupole time-of-flight (QTOF) mass spectrometer (Bruker IMPACT II, Bremen, Germany) at Southwest University School of Chemistry and Chemical Engineering. NMR characterization (1D and 2D) of all compounds were performed on Bruker AVANCE III NMR (400 MHz) with a 5 mm broadband probe at the Analysis and Testing Center of the Southwest University College of Pharmaceutical Sciences and Chinese Medicines.

NMR spectra of compounds **1, 2, 3, 6, 11, 12** and **14** were acquired in $CDCl_3$ with TMS as an internal standard. NMR spectra of compounds **7** and **8** were acquired in DMSO-d_6 with TMS as an internal standard.

2. Supplementary Tables

Supplementary Table 1. Stains or plasmids used in this study.

Stains or plasmids	characteristics	Reference or source
Aspergillus flavipes KLA03	Aspochalasin producing wild-type strain	[1]
Aspergillus nidulans LO8030	Host for heterologous expression	A gift from Prof. Wenbing Yin from the Institute of Microbiology, CAS
Saccharomyces cerevisiae strain BJ5464-NpgA	Host for the expression of aspoA and for heterologous recombination to construct the *A. nidulans* overexpression plasmids.	[2]
Escherichia coli BL21	Host for protein expression	Novagen
Escherichia coli XL-1	General cloning host	Stratagene
pColdI	Protein expression vector used in *E.coli*, encoding N-terminal 6 ×His-tag, ampicillin resistance	Takara
pQ8	Protein expression vector used in *E.coli*, encoding N-terminal His×6-MBP-His×6-tag, kanamycin resistance	[3]
pGEX-4T-1	Protein expression vector used in *E.coli*, encoding N-terminal GST-tag, ampicillin resistance	GE Healthcare
pYEU	*E. coli-Saccharomyces* shuttle vector, ampicillin resistance, *Ura3*	[4]
pANU	*E. coli-Saccharomyces-A. nidulans* shuttle vector for heterologous expression, ampicillin resistance, *Ura3* and *pyrG89*	[4]
pANR	*E. coli-Saccharomyces-A. nidulans* shuttle vector for heterologous expression, ampicillin resistance, *Ura3* and *riboB2*	[4]
pANP	*E. coli-Saccharomyces-A. nidulans* shuttle vector for heterologous expression, ampicillin resistance, *Ura3* and *pyroA4*	[4]

Supplementary Table 2. Primers used in this study.

Primer name	Primer sequence (5'→3')
pANR-*aspoE*-F1	ACCCCGCCACATAGACACATCTAAACAATGGCCACCACCTCTGCCACCTC
pANR-*aspoE*-F2	GATACGTATCTGCTGCCCTTGAAG
pANR-*aspoE*-F3	AGCTAATGTGCTGGAGTGTCTGCCCG
pANR-*aspoE*-F4	GGTTGGGGTCTTCCAGGATCCCTC
pANR-*aspoE*-R1	TCCTCGAGTGACCAACTTGACCGC
pANR-*aspoE*-R2	CAGCCGACAGAACTTTGCAGTTTG
pANR-*aspoE*-R3	CCAGAATAGTGCAGAAGAAGTCGG
pANR-*aspoE*-R4	AAAGGGTATCATCGAAAGGGAGTCATCCAATTTAAATGAATCTTGAGCAGTTGCCCTTG
pANU-*aspoH*-F	GCCTGAGCTTCATCCCCAGCATCATTACACCTCAGCAATGTCCCCCGCTCGCACATTAG
pANU-*aspoH*-R	CCGTCACCCAAATCAATTCACCGGAGTGCGGCCGCTCCTTCCACCGTCCGGCAGACAAC
pANU-*aspoC*-F	CCCCGCCACATAGACACATCTAAACAATGACCGGTTCCTCAGCAGTCCGC
pANU-*aspoC*-R	GCTCGTTCGGCACCTTTAATCATTTAAATGCGGCCGCTCGGAGTTCCGAACCCACATAC
pANU-*aspoB*-F	ACAATAAACCCCACAGAAGGCATTTATGACGCAAACCAAGCTCCTCCTTC
pANU-*aspoB*-R	AGTGGAGGACATACCCGTAATTTTCTGGGCATTTAAATGATCTGATATCTGGTGTATAC
pANU-*aspoH* -ZR	AGTGGAGGACATACCCGTAATTTTCTGGCGGCCGCTCCTTCCACCGTCCGGCAGACAAC
pANU-*aspoC* -ZR	AGTGGAGGACATACCCGTAATTTTCTGGCGGCCGCTCGGAGTTCCGAACCCACATACAG
pANU-*cytoF*-F	CTCCCTTCTCTGAACAATAAACCCCACAGAAGGCATTTATGCGGGAATTGTCTACTCTC
pANU-*cytoF*-R	CACAGTGGAGGACATACCCGTAATTTTCTGGGCATTTTGTTGATGGGTAGATGAGGATG
U-*aspoH*+B-CR	TCATTTATAGCTCGTTCGGCACCTTTAATCGCGGCCGCTCCTTCCACCGTCCGGCAGAC
pANP-*aspoF*-F	CTCTGAACAATAAACCCCACAGAAGGCATTTATGTTGCAAGATATCGTCGAGCAATG
pANP-*aspoF*-ZR	GTAGGAGTGATGAGACCCAACAACCATGATACCAGGGGAACGATCCGATTGGAGCGGTC
pANP-*aspoF*-R	CACCCAAATCAATTCACCGGAGTGTTTAAACGCGGCCGCAACGATCCGATTGGAGCGGTC
pANP-*aspoA*-F	CCCGCCACATAGACACATCTAAACAATGAGACTCACCATGAGACTCATC

pANP-*aspoA*-R	ATGAGACCCAACAACCATGATACCAGGGGGTTTAAACCGACCTACGTC GGGGGTGCCAG
amyB-F	GATTAAAGGTGCCGAACGAGC
amyB-R	AAATGCCTTCTGTGGGGTTTATTG
glaA-F	CCTGATCTTCCGAACTGGTCG
glaA-R	TGCTGAGGTGTAATGATGCTG
gpdA-F	ACTCCGGTGAATTGATTTGGG
gpdA-R	TGTTTAGATGTGTCTATGTGGC
pQ8-*aspoA*-NF	CCCAAGCTTGCATGAGACTCACCATGAGAC
pQ8-*aspoA*-R	AAGGAAAAAAGCGGCCGCAATCAACCTCCCCAAACACTTC
GST-FMO-F	GGGAATGAGACTCACCATGAGACTCATC
GST-FMO-R	ATAAGAATGCGGCCGCCTAAATCAACCTCCCCAAACAC
pColdI-*aspoA*-F	CGGGATCCATGAGACTCACCATGAGACTCATC
pColdI-*aspoA*-R	CCCAAGCTTCTAAATCAACCTCCCCAAACACTTC
pcoldI-*aspoD*-F	GGAATTCCATATGATGGCCGAAGCAATCACCTCC
pcoldI-*aspoD*-R	CGGGGTACCTCAACTCCCCTCCGACAGAAGCTTC
pYEU-*aspoA*-F	GGCTAGCGATTATAAGGATGATGATGATAAGACTAGTATGAGACTCAC CATGAGACTC
pYEU-*aspoA* -R	GTCATTTAAATTAGTGATGGTGATGGTGATGCACGTGAATCAACCTCCC CAAACAC
pYEU-*aspoA*-H158A-F	CATCAAAAACAGCGGCGCCGACTATAACGGCCGAAGCAGCGGACC
pYEU-*aspoA*-H158A-R	CTTCGGCCGTTATAGTCGGCGCCGCTGTTTTTG
pYEU-*aspoA*-Y160A-F	ATCAAAAACAGCGGCCACGACGCTAACGGCCGAAGCAGCGGACCAGG CTCTC
pYEU-*aspoA*-Y160A-R	TTCGGCCGTTAGCGTCGTGGCCGCTGTTTTTG
pYEU-*aspoA*-E538A-F	GAGTGGGGCGTATGCGAATGCAGCGCATCCGTG
pYEU-*aspoA*-E538A-R	CACGGATGCGCTGCATTCGCATAC
pYEU-*aspoA*-E538D-F	GAGTGGGGCGTATGCGAATGATGCGCATCCGTG
pYEU-*aspoA*-E538D-R	CAATCCTCCACCCACGGATGCGCATCATTCGCATAC

Supplementary Table 3. Recombinant plasmids used in this study.

Name	Description	Enzyme site	Aim
pIM 8001	*aspoE* gDNA with downstream 500 bp in pANR	*Bam*H I	*A. nidulans* overexpression
pIM 8002	*aspoH* gDNA with downstream 500 bp, with downstream 500 bp in pANU	*Not* I	*A. nidulans* overexpression
pIM 8003	*aspoH* gDNA with downstream 500 bp and *aspoB* gDNA with downstream 500 bp in pANU	*Not* I	*A. nidulans* overexpression
pIM 8004	*aspoH* gDNA with downstream 500 bp a, *aspoC* gDNA with downstream 89 bp and *aspoB* gDNA with downstream 500 bp in pANU	*Not* I	*A. nidulans* overexpression
pIM 8005	*aspoH* gDNA with downstream 500 bp, *aspoC* gDNA with downstream 89 bp and *cytoF* gDNA with downstream 320 bp in pANU	*Not* I	*A. nidulans* overexpression
pIM 8006	*aspoF* gDNA with downstream 500 bp in pANP	*Bam*H I	*A. nidulans* overexpression
pIM 8007	*aspoF* gDNA with downstream 500 bp and *aspoA* gDNA with downstream 500 bp in pANP	*Bam*H I	*A. nidulans* overexpression
pIM 8008	*aspoA* cDNA in pColdI with N-His tag	*Bam*H I-*Hind* III	protein expression in *E. coli*
pIM 8009	*aspoA* cDNA in pQ8 with N-MBP tag	*Hind* III-*Not* I	protein expression in *E. coli*
pIM 8010	*aspoA* cDNA in pGEX 4T-1 with N-GST tag	*Sma* I-*Not* I	protein expression in *E. coli*
pIM 8011	*aspoD* cDNA in pColdI with N-His tag	*Nde* I-*Kpn* I	protein expression in *E. coli*
pIM 8012	*aspoA* cDNA in pYEU with N-Flag and C-His tag	*Spe* I- *Pml* I	cell-free expression in *S. cerevisiae*
pIM 8013	*aspoA-mutation*(H158A) cDNA in pYEU with N-Flag and C-His tag	*Spe* I- *Pml* I	cell-free expression in *S. cerevisiae*
pIM 8014	*aspoA-mutation*(Y160A) cDNA in pYEU with N-Flag and C-His tag	*Spe* I- *Pml* I	cell-free expression in *S. cerevisiae*
pIM 8015	*aspoA-mutation*(E538A) cDNA in pYEU with N-Flag and C-His tag	*Spe* I- *Pml* I	cell-free expression in *S. cerevisiae*
pIM 8016	*aspoA-mutation*(E538D) cDNA in pYEU with N-Flag and C-His tag	*Spe* I- *Pml* I	cell-free expression in *S. cerevisiae*

Supplementary Table 4. NMR data of compound **1** in $CDCl_3$.

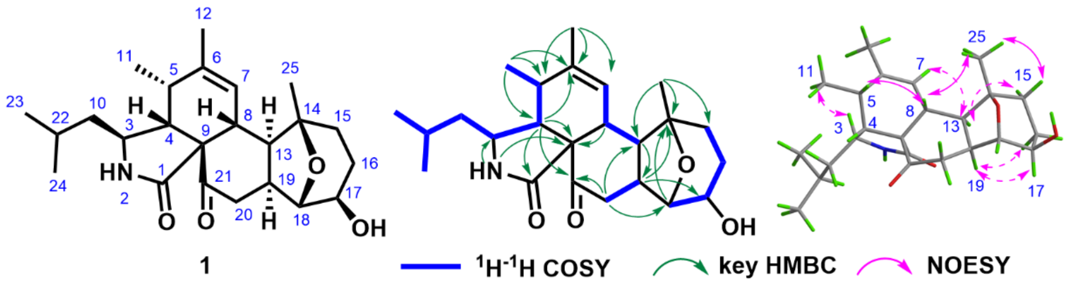

(400 MHz for ^{1}H NMR, 100 MHz for ^{13}C NMR)

NO.	δ_H, mult, (*J* in Hz)	^{13}C
1		173.8
2	6.21, s	
3	3.09, m	52
4	2.64, t (5.0)	51.9
5	2.35, m	35.2
6		140.1
7	5.41, s	127.2
8	2.44, d (11.0)	36.6
9		64.2
10	1.28, m<br>1.68, m	47.6
11	1.15, d (7.0)	13.8
12	1.76, s	20.4
13	2.92, o	42.8
14		82.9
15	1.89, td (6.0, 14.0)<br>1.43, dd (6.0, 14.0)	35.3
16	1.71, m<br>2.03, m	24.8
17	3.57, s	66.8
18	3.71, s	84.4
19	2.99, o	39
20	2.55, m	42.8
21		210.4
22	1.56, m	25.4
23	0.98 d, (7.0)	24
24	0.91 d, (7.0)	21.3
25	1.22, m	23.5

Supplementary Table 5. NMR data of compound **2** in $CDCl_3$.

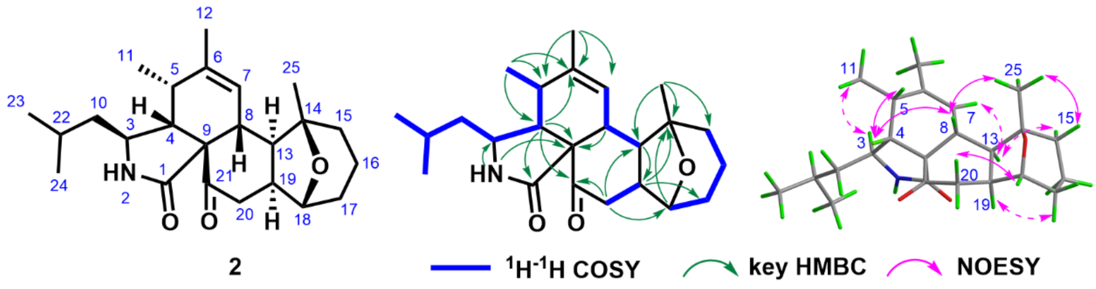

(400 MHz for ^{1}H NMR, 100 MHz for ^{13}C NMR)

**No.**	***δ*H, mult, (*J* in Hz)**	**^{13}C**
1		173.9
2	5.88, s	
3	3.09, dt (3.3, 10.3)	52.2
4	2.63, t (4.4)	51.9
5	2.35, m	35.2
6		139.6
7	5.44, s	127.7
8	2.42, d (12.2)	36.8
9		64.2
10	1.31, m<br>1.72, o	47.6
11	1.15, d (7.0)	13.8
12	1.76, s	20.4
13	2.94, dd (9.0, 12.0)	44.6
14		82.4
15	1.50, o<br>1.65, o	39.5
16	1.62, o<br>1.90, m	17.4
17	1.45, m<br>1.72, o	29.5
18	3.79,s	80.6
19	3.03, dd (9.0,17.9)	41.3
20	2.53, d (9.0)	43.2
21		211.4
22	1,56, m	25.5
23	0.92, d (6.5)	21.4
24	0.95, d (6.5)	23.8
25	1.18, s	23.9

Supplementary Table 6. NMR data of compound **3** in $CDCl_3$.

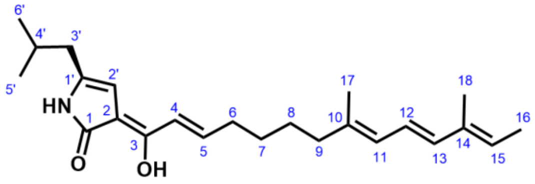

3

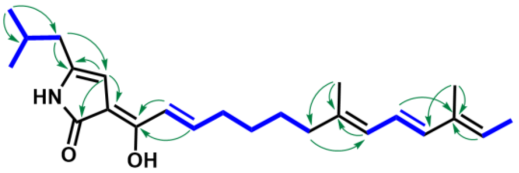

(400 MHz for ^{1}H NMR, 100 MHz for ^{13}C NMR)

NO.	***δ*H, mult, (*J* in Hz)**	**^{13}C**
1		172.6
NH	7.49, s	
2		107.0
3		162.4
4	6.17, d (15.4)	135.2
5	6.79, dt (7.2, 15.3)	143.1
6	2.28, m	33.1
7	1.48, m	28.3
8	1.48, m	27.6
9	2.09, t (6.6)	39.9
10		137.7
11	5.88, d (10.8)	125.8
12	6.32, dd (10.7, 15.3)	122.5
13	6.17, d (15.3)	121.7
14		135.7
15	5.53, o	126.4
16	1.74, d (7.0)	14.1
17	1.78, s	16.8
18	1.78, s	12.2
1'		134.0
2'	5.56, s	97.8
3'	2.23, d (7.0)	37.8
4'	1.83, m	27.6
5'	0.94, d (6.6)	22.5
6'	0.94, d (6.6)	22.5

Compound **3** was isolated as a yellow powder. HRMS gave an exact mass of 368.2585 for $[M - H]^-$ (calcd. 368.2595 for $C_{24}H_{34}NO_2$), suggesting a molecular formula of $C_{24}H_{35}NO_2$ and indicated eight degrees of unsaturation. The ^{1}H NMR spectrum showed the presence of an amide NH (δ_H 7.49, br s), seven olefinic protons [δ_H 5.53 (1H, o), 5.56 (1H, s), 5.88 (1H, d), 6.17 (2H, d), 6.32 (1H, dd), 6.79 (1H, d)], three methyl doublets (δ_H 0.94×2, and 1.74), two methyl singlets (δ_H 1.78×2), and 11 methine and/or methylene protons. Detailed analyses of ^{13}C NMR, DEPT and HSQC spectra revealed that it has five methyl, five methylene, eight methine, and five quaternary carbons in addition to an amide carbonyl (δ_C 172.6). Four of the quaternary carbons were olefinic (δ_C 107.0, 134.0, 135.7, 137.7), and one was oxygenated (δ_C 162.4). Besides seven degrees of unsaturation occupied by carbonyls and double

bonds (δ_C 97.8, 121.7, 122.5, 125.8, 126.4, 135.2, 143.1, 172.6), the remaining suggested compound **3** possessing one ring system. Analysis of the ^{1}H-^{1}H COSY NMR data of compound **3** identified the C-3'/C-4'/C-5'/C-6', C-4/C-5/C-6/C-7/C-8/C9, C-11/C-12/C-13 and C-15/C-16 four moieties. HMBC correlations from Me-17 to C-9/C-10, Me-18 to C-13/C-14, from olefinic proton H-11 and H-15 to olefinic quaternary carbon C-10 and C-14, and H-4 to oxygenated quaternary carbon C-3 established the long chain substructure. A Five membered lactam ring was confirmed by HMBC correlation from olefinic proton H-2' to olefinic quaternary carbon C-1'/C-2 and to amide carbonyl C-1. Isobutyl group was adjacent to C-1' supported by HMBC correlations among H-3' between C-1'. The double bonds configuration of compound **3** was determined based on ^{1}H-^{1}H coupling constants and NOESY experiments. The coupling constants of $J_{4,5}$ =15.4 Hz and $J_{12,13}$ = 15.3 Hz and the NOESY correlations of H-12 with Me-17 and Me-18, H-11 with H-13 indicated the presence of four E-configured double bonds. Therefore, the structure of compound **3** was determined.

Supplementary Table 7. NMR data of compound **6** in $CDCl_3$.

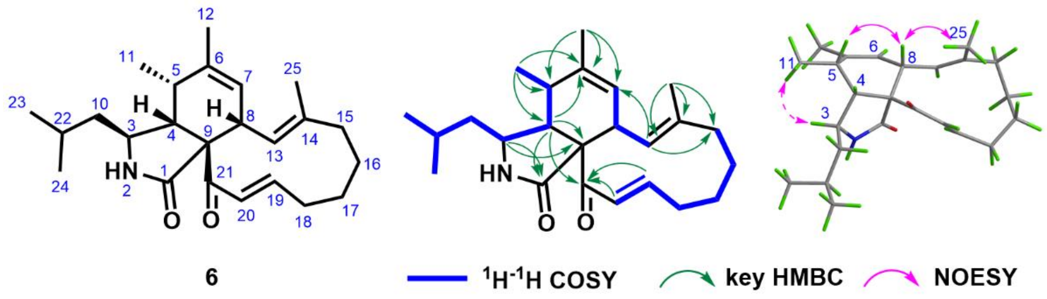

(400 MHz for ^{1}H NMR, 100 MHz for ^{13}C NMR)

No.	δ_H, mult, (J in Hz)	^{13}C
1		174.8
2	5.89, s	
3	3.12, d (4.6)	49.4
4	3.12, d (4.6)	51.1
5	2.46, m	35.1
6		140.5
7	5.44, s	126.3
8	2.84, d (10.8)	43.5
9		67.9
10	1.22, o	48.5
11	1.24, o	13.8
12	1.76, s	20.1
13	6.05, d (10.8)	125.2
14		138.2
15	1.94, dd (6.9, 13.0)<br>2.09, m	41.1
16	1.70, m<br>1.81, m	28.5
17	1.42, m<br>1.67, m	27.8
18	2.03, m<br>2.16, m	32.1
19	6.74, dt (6.9, 16.0)	142.9
20	7.31, d (16.0)	131.2
21		197.6
22	1.49, m	25.3
23	0.88, d (6.5)	23.8
24	0.90, d (6.5)	21.5
25	1.33, s	17.3

Supplementary Table 8. NMR data of compound **7** in DMSO-d_6.

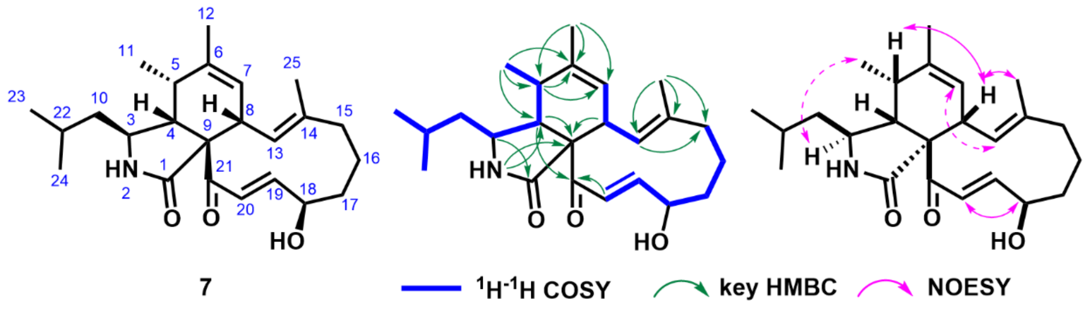

7

(400 MHz for ^{1}H NMR, 100 MHz for ^{13}C NMR)

No.	δ_H, mult, (J in Hz)[b]	^{13}C[b]
1		173.2
2	8.04,s	
3	3.03,s	50.1
4	2.86,dd (3.0, 5.7)	48.6
5	2.36,s	34.4
6		139.9
7	5.32,s	125.4
8	2.76,d (10.7)	42.7
9		67.7
10	1.06,t (6.8)	48.3
11	1.18,d (7.2)	13.2
12	1.72,s	19.6
13	5.93,d (10.8)	125.0
14		136.7
15	1.94,o	40.4
16	1.65,o<br>1.43,m	20.4
17	1.88,o<br>1.57,o	37.6
18	4.22,m	70.0
19	6.39,dd (16.4, 5.6)	146.8
20	7.10, d (16.4)	127.2
21		197.2
22	1.57,o	23.9
23	0.81,d (6.5)	23.5
24	0.83,d (6.5)	21.5
25	1.28,s	16.9
18-OH	5.00, s	

Supplementary Table 9. NMR data of compound **8** in DMSO-d_6.

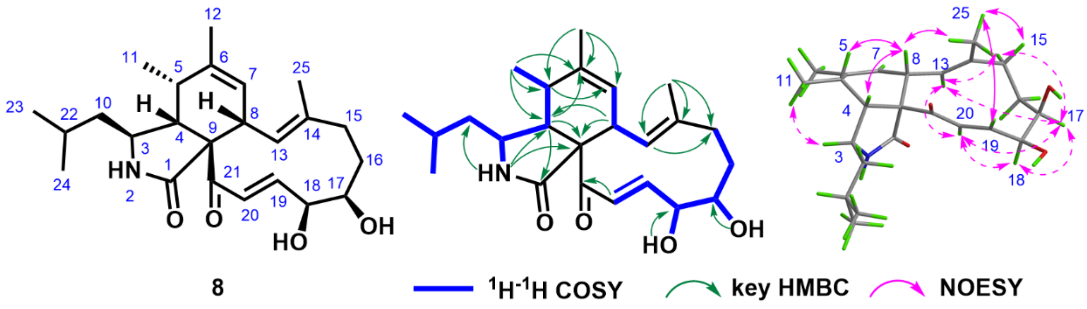

(400 MHz for ^{1}H NMR, 100 MHz for ^{13}C NMR)

No.	δ_H, mult, (*J* in Hz)	^{13}C
1		173.5
2	8.09, s	
3	3.04, t (6.2)	50.0
4	2.82, dd (2.4, 5.9)	48.5
5	2.36, t (6.6)	34.5
6		139.8
7	5.33, s	125.4
8	2.79, d (14.2)	42.8
9		67.9
10	1.05, t (7.0)	48.6
11	1.16, d (9.0)	13.1
12	1.72, s	19.6
13	5.89, d (10.8)	124.4
14		136.1
15	1.96, dd (11.0, 12.0)<br>2.04, dd (7.0,12.0)	39.4
16	1.33, dd (6.6, 13.0)<br>1.87, dd (3.4,12.0)	29.1
17	3.40, t (6.2)	78.6
OH-17	4.83, d (5.3)	
18	4.30, s	74.5
OH-18	5.01, d (4.0)	
19	6.17, dd (4.8, 16.7)	144.1
20	7.04, dd (1.2, 16.7)	128.3
21		197.2
22	1.57, m (6.7, 13.2)	23.9
23	0.83, d (2.0)	21.5
24	0.81, d (2.0)	23.5
25	1.19, s	15.2

Supplementary Table 10. NMR data of compound **11** in $CDCl_3$.

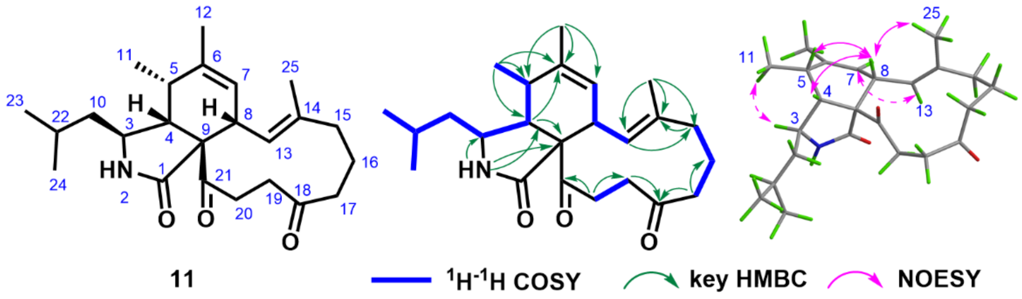

(400 MHz for ^{1}H NMR, 100 MHz for ^{13}C NMR)

**No.**	***δ*$_H$, mult, (*J* in Hz)**	**^{13}C**
1		175.8
2	5.88, s	
3	3.14, m	50.7
4	2.63, o	52.8
5	2.56, m	35.4
6		139.8
7	5.38, s	126.1
8	3.01, d (10.7)	43.3
9		67.1
10	1.16, m	48.8
11	1.19, d (7.0)	13.5
12	1.73, s	19.9
13	6.22, d (10.7)	124.5
14		137.8
15	1.99, td (3.8, 13.0)<br>2.16, m	41.3
16	1.57, o<br>2.27, m	20.1
17	2.08, dd (7.5, 17.0)<br>2.63, o	39.3
18		208.5
19	2.72, ddd (2.6, 8.0, 13.9)<br>2.80, ddd (2.6,11.5. 13.9)	38.1
20	2.42, ddd (2.8, 8.0, 17.6)<br>3.85, ddd (2.6,11.4,17.6)	37.7
21		208.2
22	1.52, o	25.2
23	0.90, d (2.0)	23.7
24	0.89, d (2.0)	21.7
25	1.35, s	14.9

Supplementary Table 11. NMR data of compound **12** in $CDCl_3$.

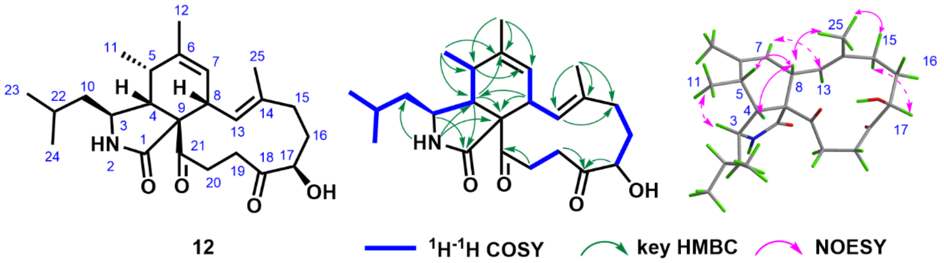

(400 MHz for ^1H NMR, 100 MHz for ^{13}C NMR)

**No.**	δ_H**, mult, (*J* in Hz)**	^{13}**C**
1		176.3
3	3.12, m	51.1
4	2.44, m	55.4
5	2.63, m	35.5
6		139.5
7	5.36, s	126.2
8	3.25, d (10.8)	42.3
9		68.7
10	1.24, m	49.0
11	1.18, d (7.0)	13.6
12	1.74, s	20.0
13	6.16 d(10.8)	125.1
14		137.1
15	2.01, m<br>2.23, o	30.7
16	2.17, o	32.9
17	4.23, d (5.7)	74.6
18		209.3
19	2.66, m<br>2.85, td (3.0,11.9)	37.3
20	2.38, m<br>4.37, ddd (2.3,11.4,13.9)	33.9
21		209.2
22	1.56, m	25.3
23	0.92, d (6.5)	23.7
24	0.90, d (6.5)	21.6
25	1.37, s	18.7

Supplementary Table 12. NMR data of compound **14** in $CDCl_3$.

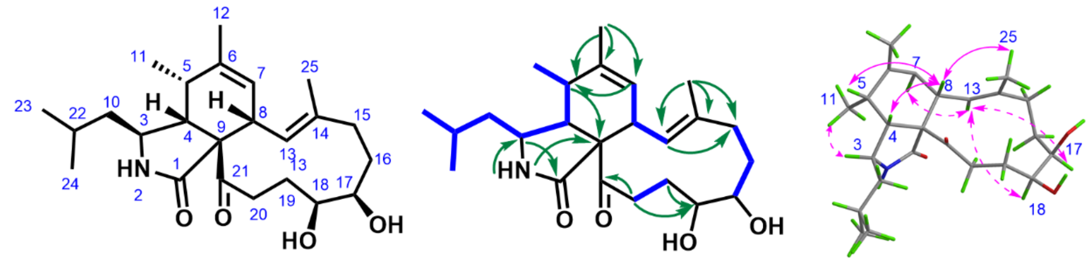

14 ^1H-^1H COSY   key HMBC   NOESY

(400 MHz for ^1H NMR, 100 MHz for ^{13}C NMR)

No.	δ_H, mult, (J in Hz)	^{13}C
1		176.2
2	6.17, s	
3	3.14 m	51.0
4	2.54, o	53.5
5	2.59, o	35.4
6		139.6
7	5.42 s	126.1
8	3.26 d (8.8)	43.3
9		68.4
10	1.18, m	49.0
11	1.19 d (7.2)	13.6
12	1.75 s	20.0
13	6.06 d (10.9)	125.0
14		136.9
15	2.15, m	37.8
16	1.60, o<br>1.69, o	28.7
17	3.92, t (4.8)	75.4
18	4.18, brs	73.2
19	1.38, m<br>1.82, m	26.7
20	2.02, m<br>3.33, o	34.9
21		214.0*
22	1.58, o	25.2
23	0.89 d (6.5)	21.7
24	0.91 d (6.5)	23.7
25	1.54 s	15.9

*Data from 2D NMR.

3. Supplementary Figures

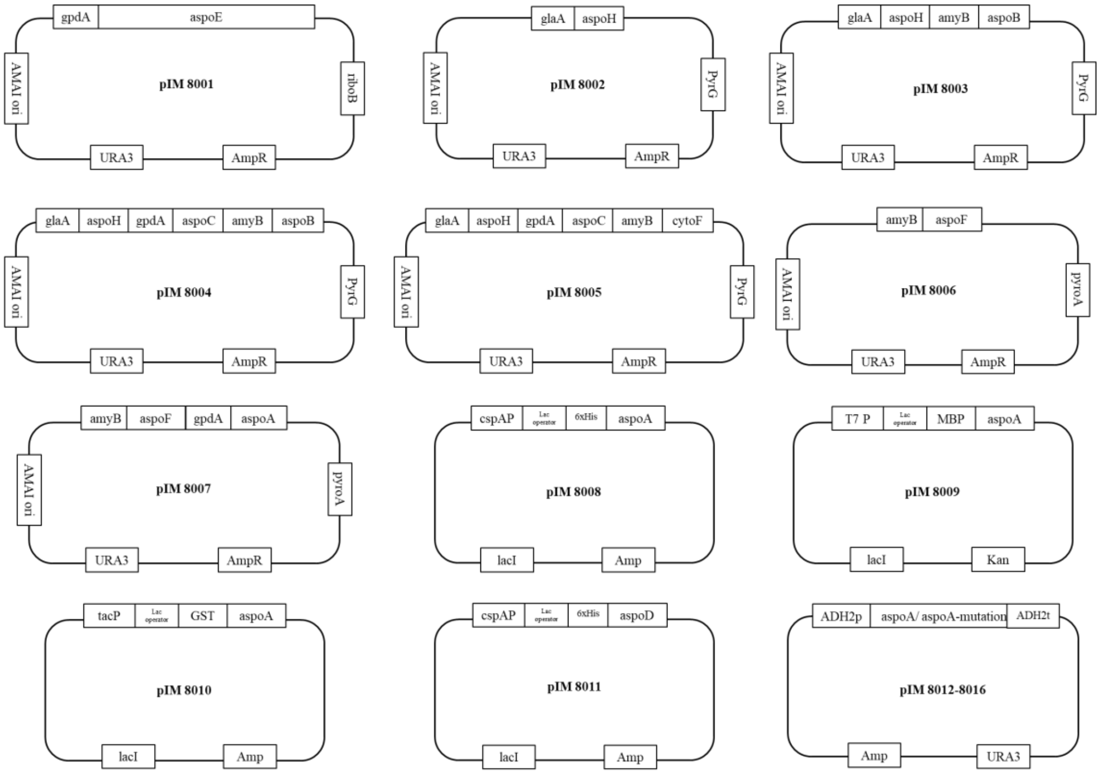

Supplementary Figure 1. Schematic diagram of plasmids used in this study.

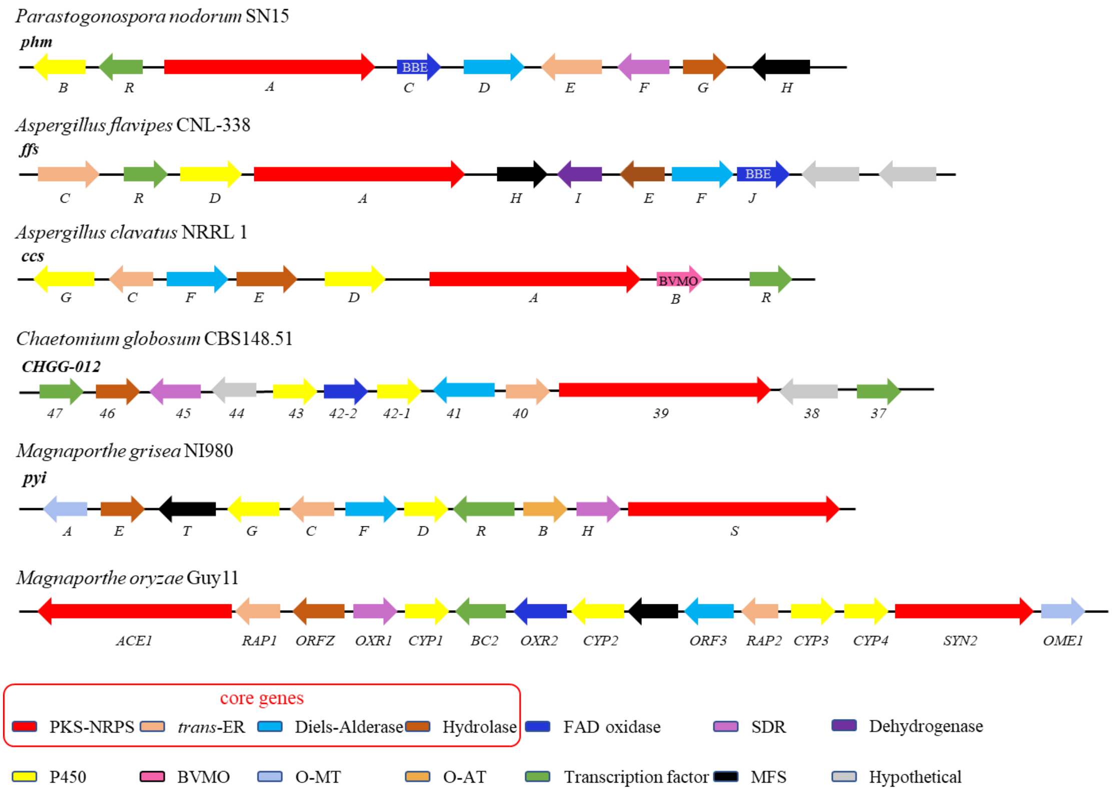

Supplementary Figure 2. Identified CYT biosynthetic gene clusters containing aliphatic amino acid-type and aromatic amino acid-type clusters.

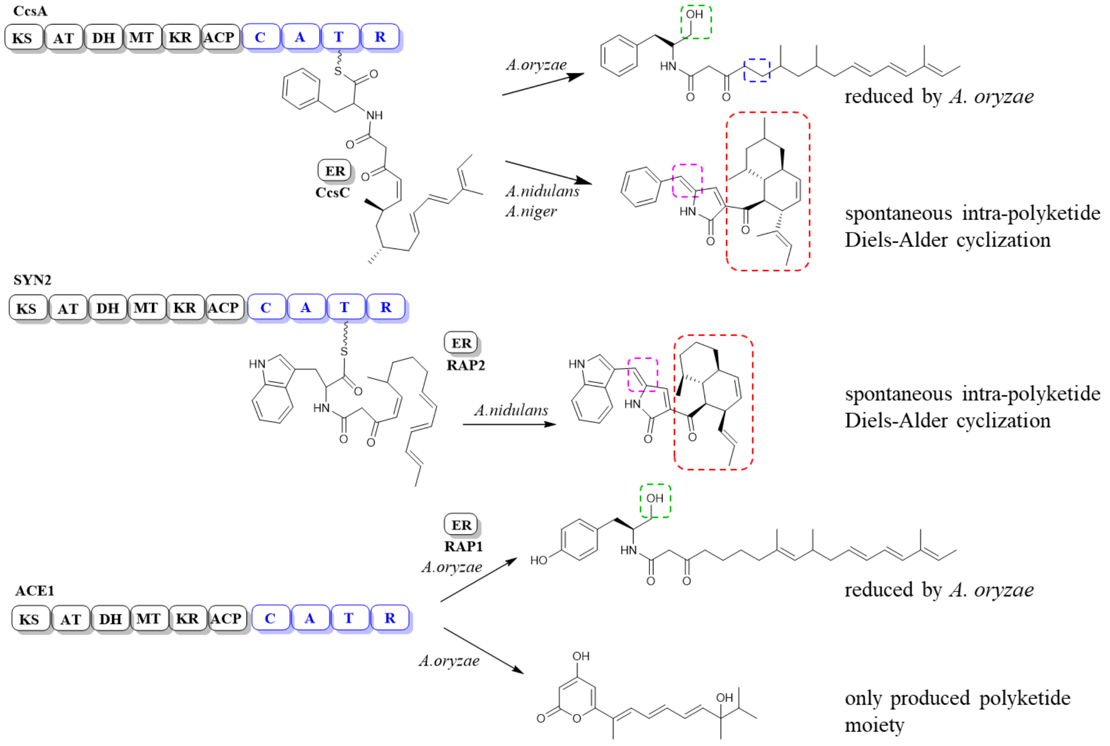

Supplementary Figure 3. Previously unsuccessful examples that reconstitution of aromatic ammino acid-type *cyt* BGCs in heterologous hosts. Some unexpected reduction and tailoring steps were highlighted in the dashed box.

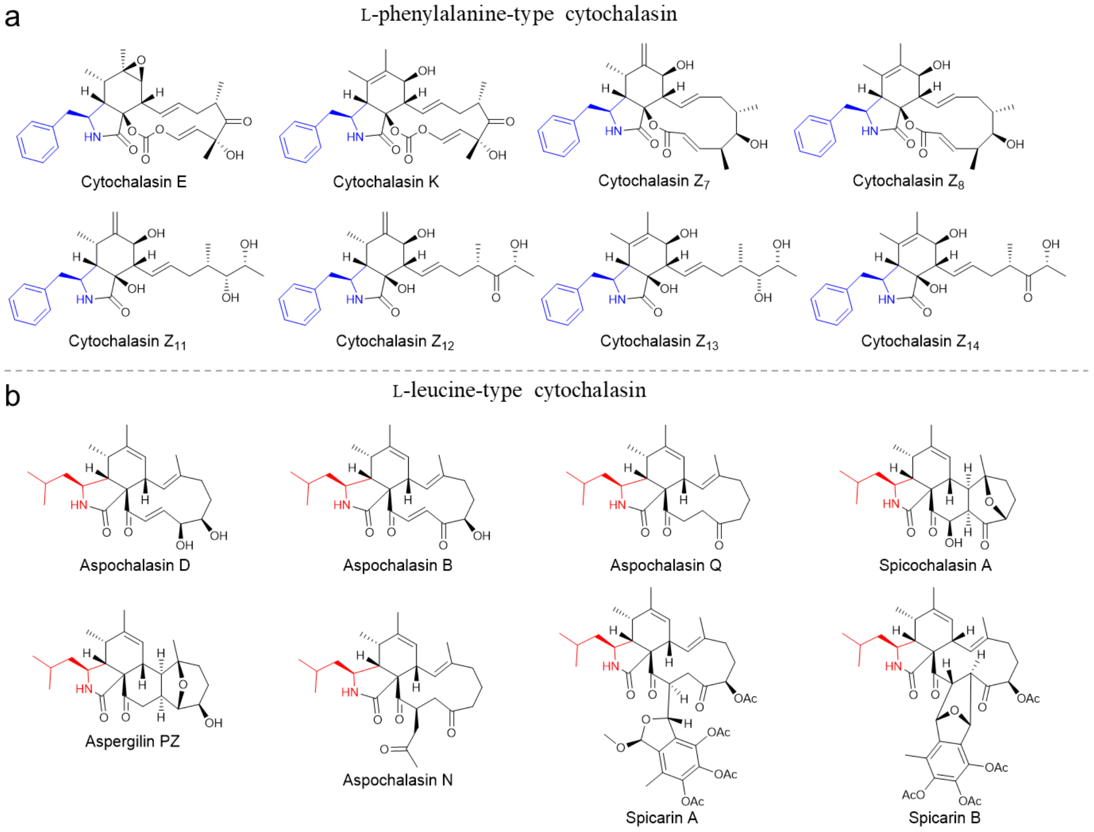

Supplementary Figure 4. Representative aromatic amino acid-type and aliphatic amino acid-type cytochalasans isolated from *A. flavipes* KLA03. **a** L-phenylalanine-type moCYTs; and **b** L-leucine-type aspochalasin family compounds containing moCYTs, pcCYTs and meCYTs.

a

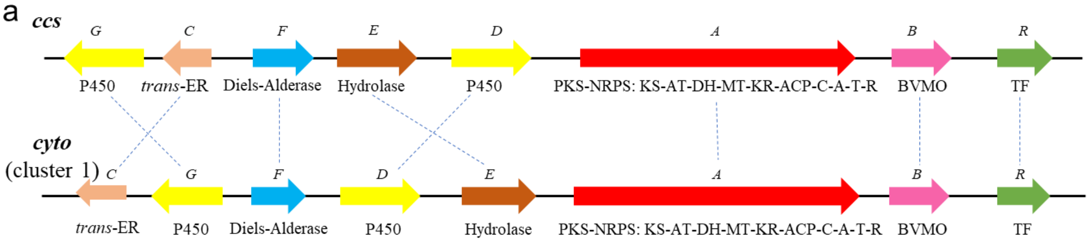

***cyto* cluster proteins**	***ccs* cluster proteins**	**Identity(%)**	**Deduced function**
CytoA	CcsA	62	PKS-NRPS
CytoB	CcsB	72	Baeyer–Villiger monooxygenase (BVMO)
CytoC	CcsC	79	*trans*-enoyl reductase(ER)
CytoD	CcsD	74	Cytochrome P450 monooxygenase
CytoE	CcsE	81	*α*/*β*-hydrolase
CytoF	CcsF	81	Diels-Alderase
CytoG	CcsG	67	Cytochrome P450 monooxygenase
CytoR	CcsR	39	Transcription regulator

b

**Protein name**	**A domain code**	**substrate**	**amino acid type**
CHGG_01239	D I F A L G D F	L-Trp	aromatic amino acids
PyiS	D M G S H G C F	L-Tyr	
ACE1	D M A Q L G G I	L-Tyr	
CcsA	D M S X V G C F	L-Phe	
CytoA	D M S E V G C F	L-Phe	

Supplementary Figure 5. Comparison of *cyto* (cluster 1) gene cluster with *ccs* gene cluster (**a**) and A domain codes predicted for substrate recognition of PKS-NRPS proteins in *cyt* BGCs (**b**).

a

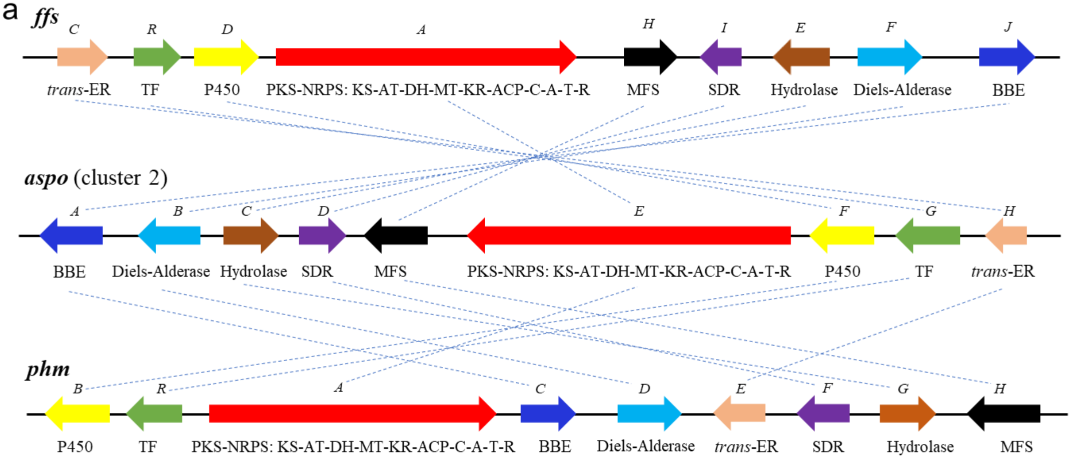

aspo cluster proteins	*ffs/phm* cluster proteins	Identity (%)	Deduced function
AspoA	FfsJ/PhmC	85/55	BBE-like oxidase (BBE/GlcD)
AspoB	FfsF/PhmD	96/75	Diels-Alderase
AspoC	FfsE/PhmG	97/74	α/β-hydrolase
AspoD	FfsI/PhmF	95/69	Short chain dehydrogenase
AspoE	FfsA/PhmA	94/65	PKS-NRPS
AspoF	FfsD/PhmB	98/64	Cytochrome P450 monooxygenase
AspoG	FfsR/PhmR	96/46	Transcription regulator
AspoH	FfsC/PhmE	99/74	*trans* enoyl reductase

b

Protein name	A domain code	substrate	amino acid type
PhmA	D I S Q M G A L	L-Leu	aliphatic amino acids
FfsA	D I S Q M G A L	L-Leu	
AspoE	D I S Q M G A L	L-Leu	

Supplementary Figure 6. Comparison of *aspo* (cluster 2) gene cluster with *ffs* cluster and *phm* cluster (**a**) and A domain codes predicted for substrate recognition of PKS-NRPS proteins in three gene clusters (**b**).

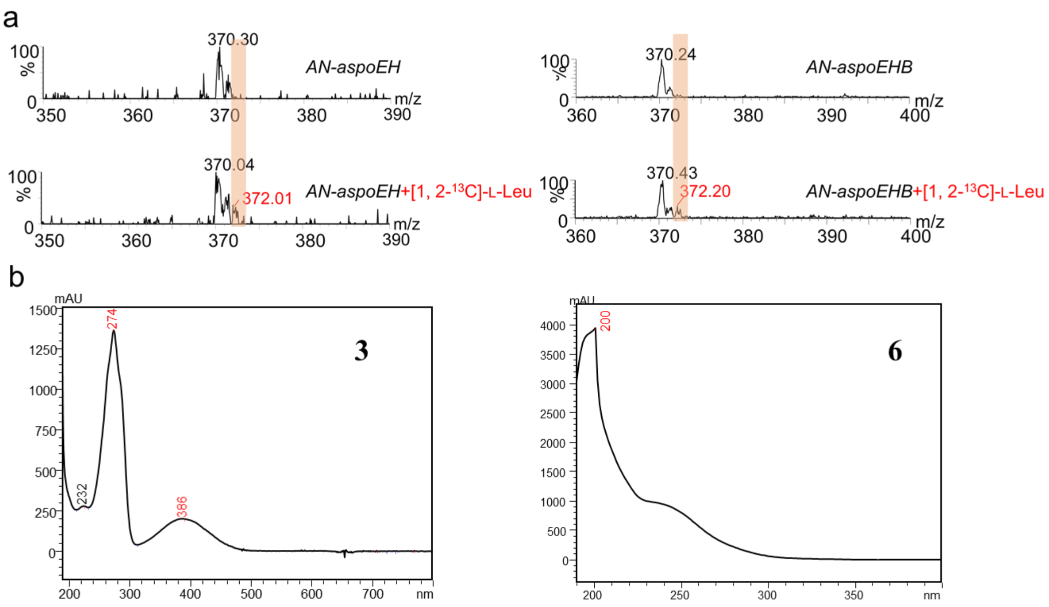

Supplementary Figure 7. a The isotope chemical feeding assays in *AN-aspoEH* and *AN-aspoEHB* showed that the [1, 2-^{13}C]-L-leucine was introduced into **3** and **6** respectively. The molecular weight of **3** and **6** could increase 2 amu. **b** The UV absorption spectrum of compound **3** and **6**.

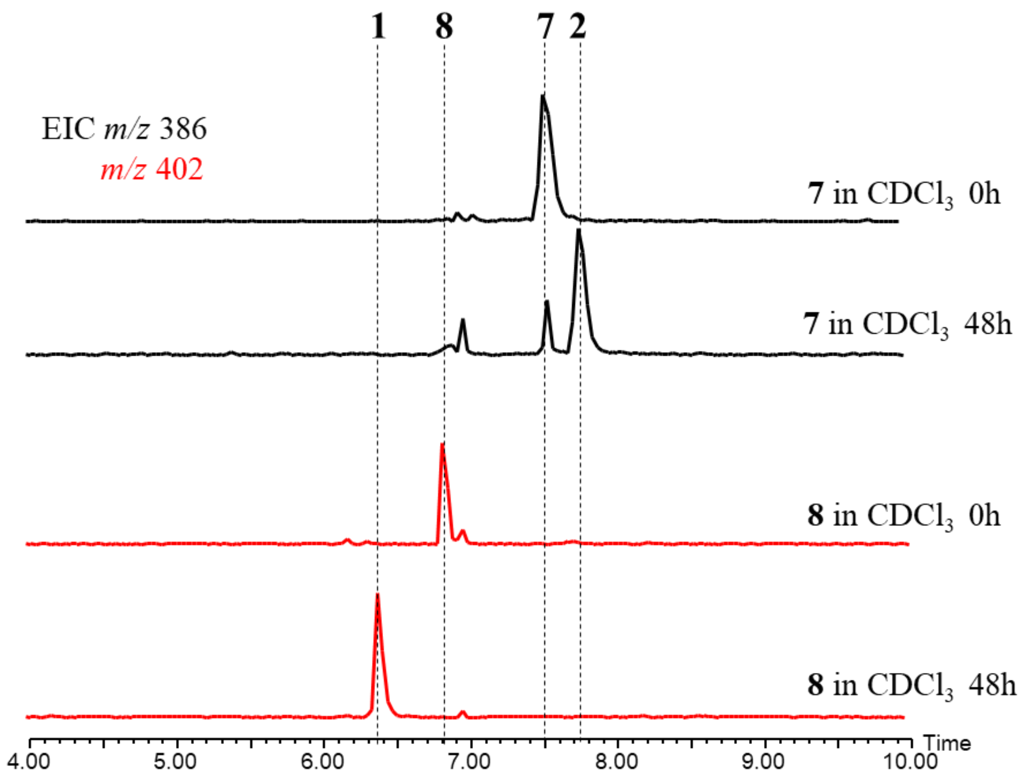

Supplementary Figure 8. Compounds **7** and **8** were converted into **2** and **1** in $CDCl_3$ after 48 h. The extracted ion chromatograms (EICs) were extracted at *m/z* 386 $[M+H]^+$ for **7** and **2**, *m/z* 402 $[M+H]^+$ for **8** and **1**.

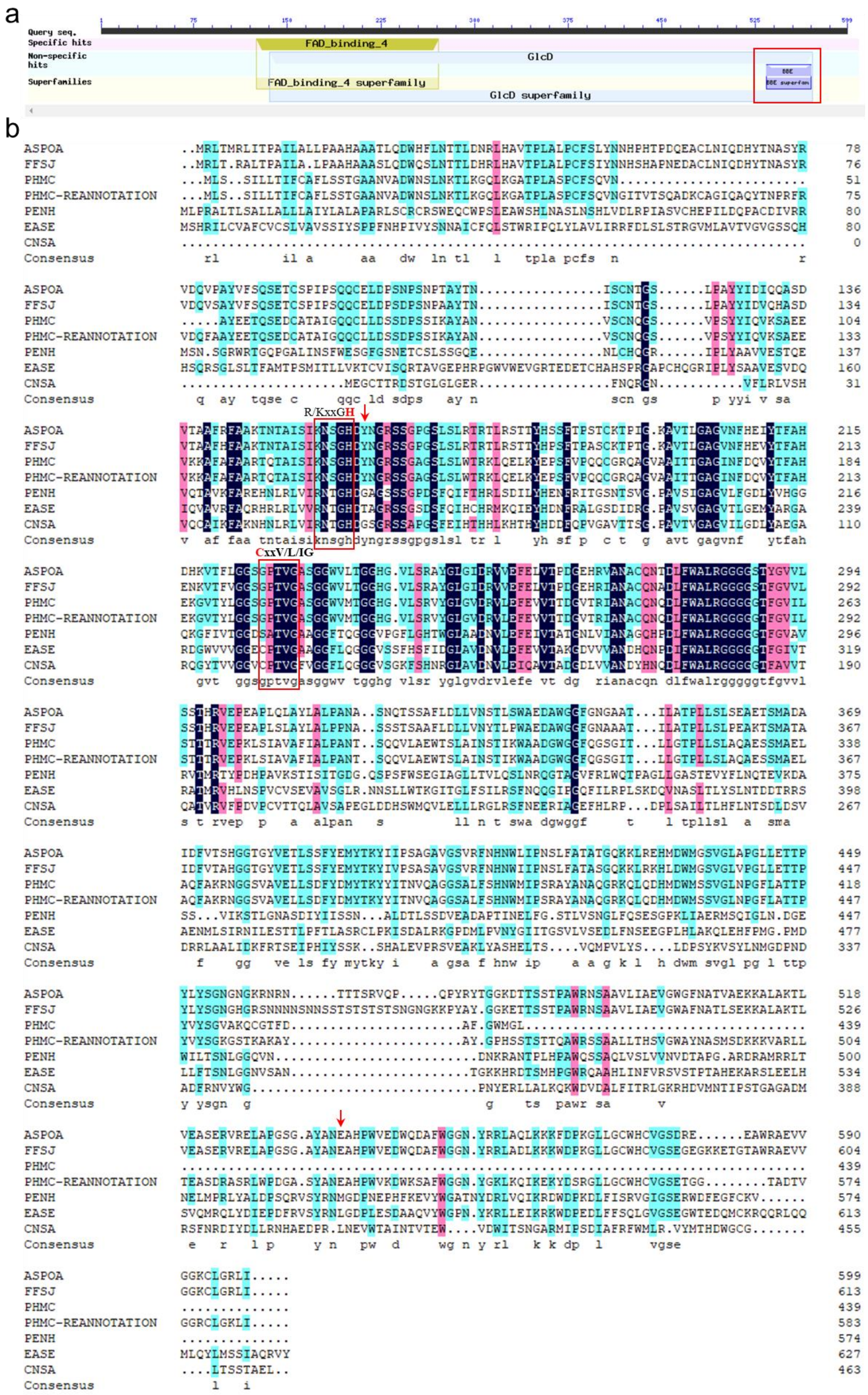
a
Query seq.
Specific hits
Non-specific hits
Superfamilies
FAD_binding_4
GlcD
FAD_binding_4 superfamily
GlcD superfamily
BBE
b
ASPOA
FFSJ
PHMC
PHMC-REANNOTATION
PENH
EASE
CNSA
Consensus
R/KxxGH
CxxV/L/IG

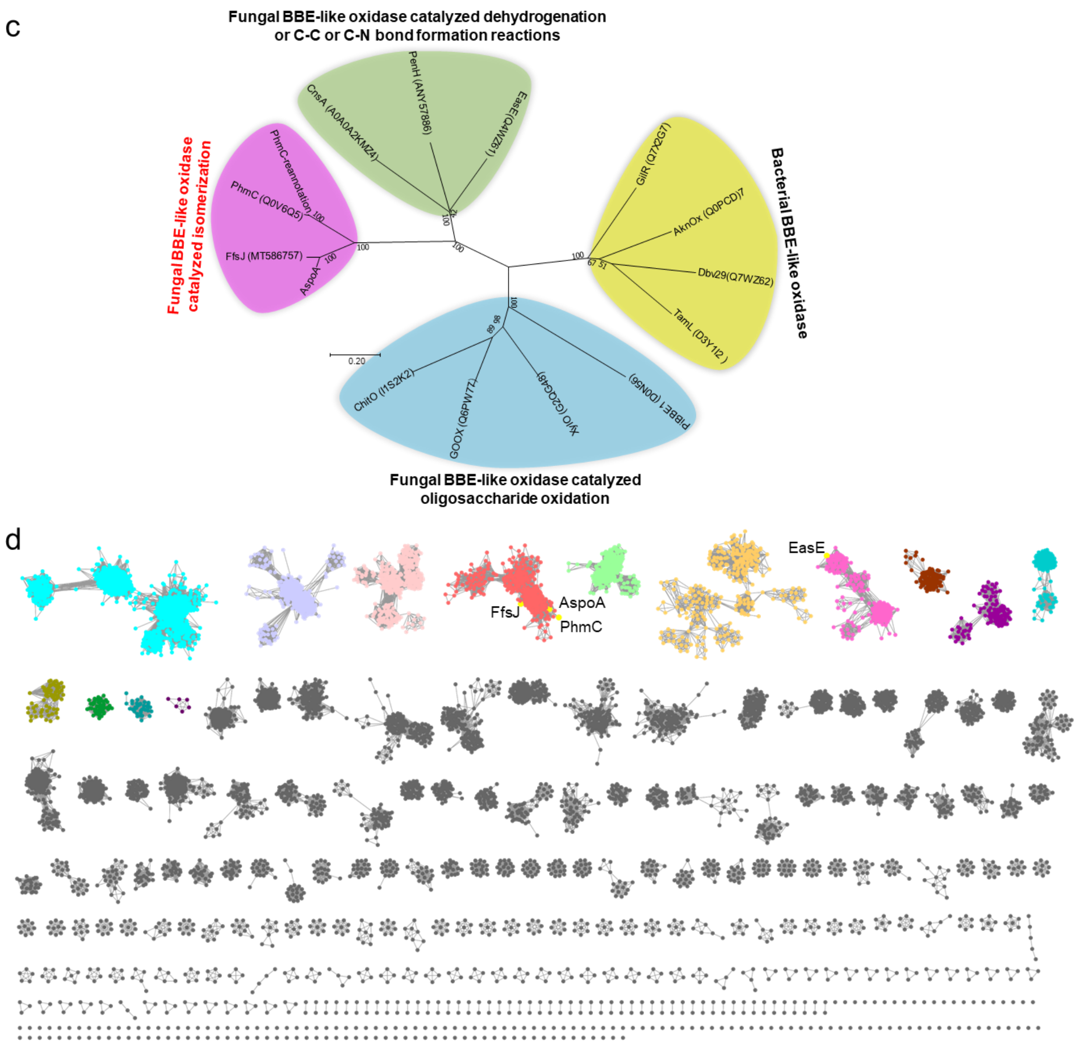

Supplementary Figure 9. Characterization and analyses of BBE-like oxidases. **a** Sequence analysis shows that AspoA contains the GlcD/BBE conserved domain. **b** Multiple sequence alignments toward AspoA homologous enzymes and classical BBE-like oxidases from fungi. Two conserved fingerprint motifs “R/KxxGH” and “CxxV/L/IG” of BBE-like oxidases were marked with red box. The key residues of Glu_{538} and Tyr_{160} were labeled with red arrows. **c** Phylogenetic tree analysis of identified BBE-like oxidases, the NCBI number of each protein is shown in figure.

CnsA (A0A0A2KMZ4, https://www.ncbi.nlm.nih.gov/protein/A0A0A2KMZ4.1),
PenH (ANY57886, https://www.ncbi.nlm.nih.gov/protein/ANY57886.1),
EasE (Q4WZ61.1, https://www.ncbi.nlm.nih.gov/protein/Q4WZ61),
PhmC (Q0V6Q5, https://www.ncbi.nlm.nih.gov/protein/Q0V6Q5),
FfsJ (MT586757, https://www.ncbi.nlm.nih.gov/nuccore/MT586757),
GilR (Q7X2G7, https://www.ncbi.nlm.nih.gov/protein/Q7X2G7),
AknOx (Q0PCD7, https://www.ncbi.nlm.nih.gov/protein/Q0PCD7),
Dbv29(Q7WZ62, https://www.ncbi.nlm.nih.gov/protein/Q7WZ62),

TamL (D3Y1I2, https://www.uniprot.org/uniprot/D3Y1I2),
ChitO (I1S2K2, https://www.ncbi.nlm.nih.gov/protein/I1S2K2),
GOOX (Q6PW77, https://www.ncbi.nlm.nih.gov/protein/Q6PW77),
XylO (G2QG48, https://www.ncbi.nlm.nih.gov/protein/G2QG48),
PiBBE1 (D0N56, https://www.uniprot.org/uniprot/D0N569).

d Sequence similarity network (SSN) analysis of fungal BBE-like oxidases further shows that AspoA is indeed divided into a separate evolutionary clade. SSN of 10438 fungal BBE-like oxidase homologs generated by Cytoscape (v3.8.2). Each node in the network represents a protein sequence and the alignment score is 120. AspoA, FfsJ, PhmC and EasE are the highlighted by a yellow point.

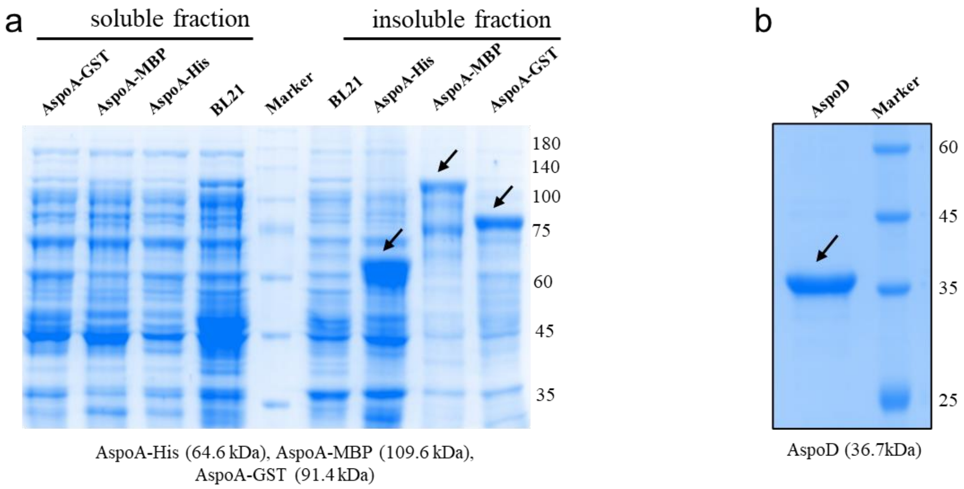

Supplementary Figure 10. SDS-PAGE analyses of the heterogeneously expressed proteins in this study. **a** The insoluble protein expression of AspoA with His-tag (64.6 kDa), GST-tag (91.4 kDa) and MBP-tag (109.6 kDa) in *E. coli* BL21. **b** AspoD (36.7 kDa) purified from *E. coli* BL21 with His-tag. All experiments were repeated independently more than three times with similar results.

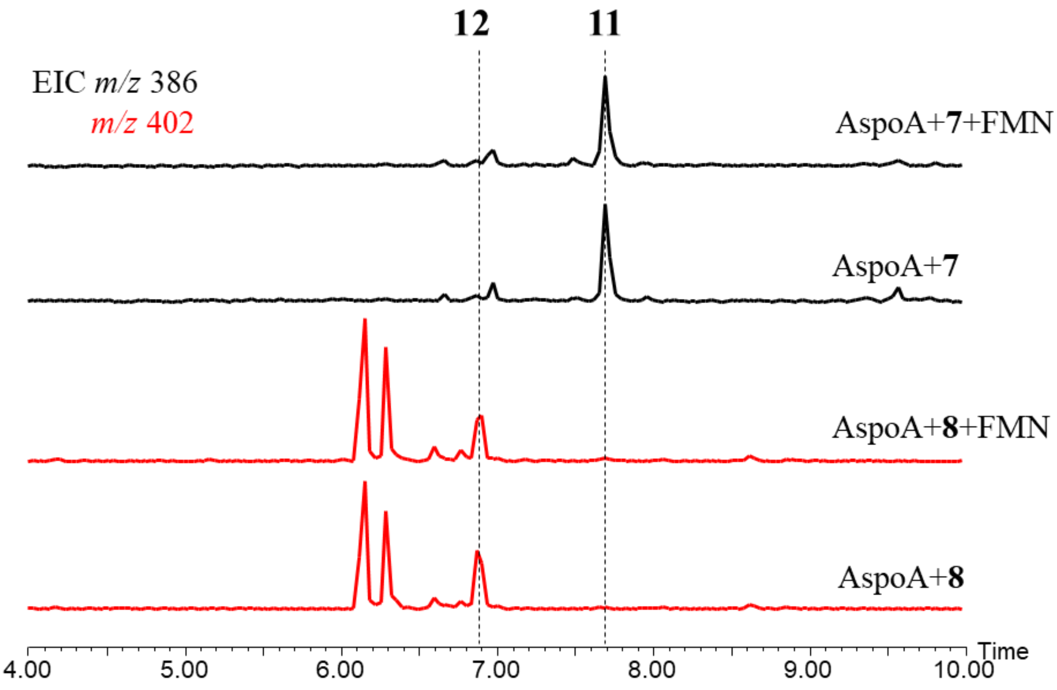

Supplementary Figure 11. The cell free bioconversion assays of compound **7** or **8** to **11** or **12**. The exogenous addition of FMN does not increase the activity of AspoA. The extracted ion chromatograms (EICs) were extracted at *m/z* 386 $[M+H]^+$ for **11**, *m/z* 402 $[M+H]^+$ for **12**.

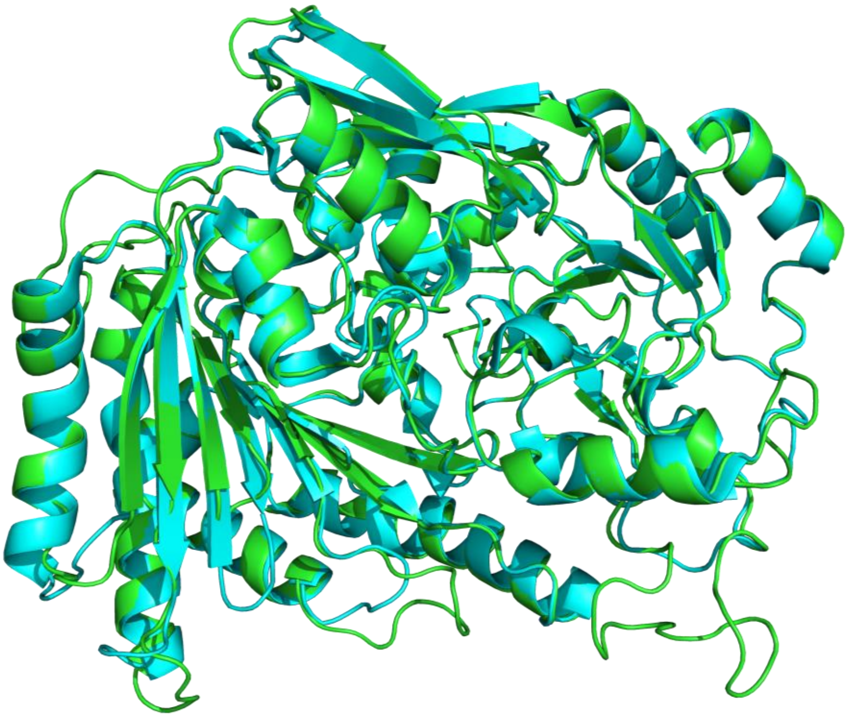

Supplementary Figure 12. The overall structure of the template MtVAO615 (cyan, PDB: 6F72) and model of AspoA (green). The template MtVAO615 was download from Protein Data Bank. The homology model of AspoA was constructed by SWISS-MODEL. The overlay was completed by PyMOL 2.2.0.

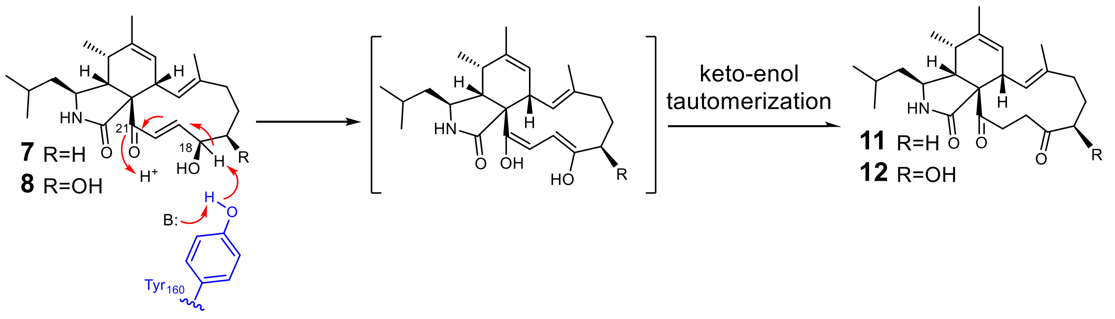

Supplementary Figure 13. The proposed mechanism that AspoA uses Tyr_{160} as the base to abstract the C_{18} hydrogen.

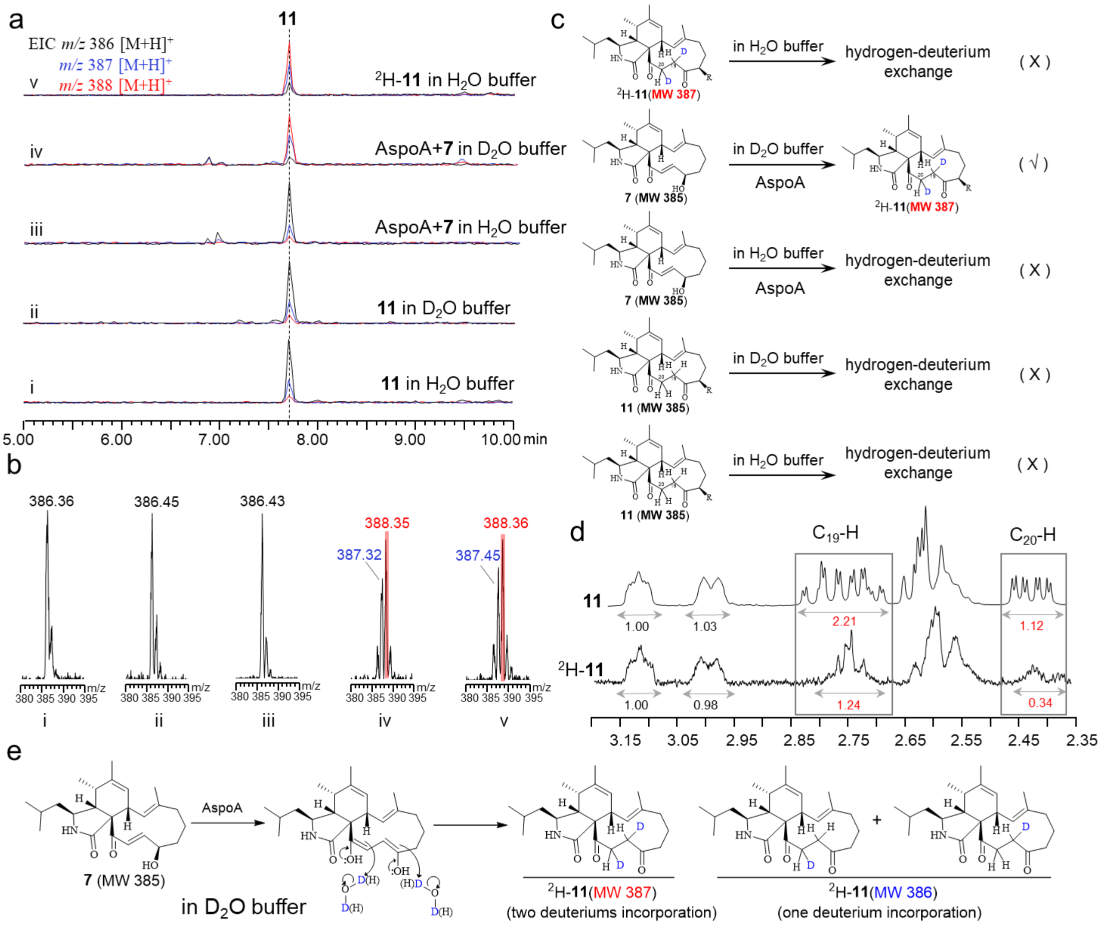

Supplementary Figure 14. The AspoA-catalysed conversion of **7** to **11** in D_2O buffer. **a** Biochemical assays show that AspoA uses **7** as the substrate to generate ^{2}H-**11** in D_2O buffer. **b** The MS data of **11** or ^{2}H-**11** in different buffer (corresponding to **a**). **c** The deuterium-hydrogen exchange occurs in AspoA-catalysed conversion of **7** to **11**. **d** The partial ^{1}H NMR spectrums of **11** and ^{2}H-**11** show that two deuteriums are incorporated into C_{19} and C_{20}. In comparison with **11**, both C_{19}-H and C_{20}-H signals of ^{2}H-**11** decreased suggesting one of the C_{19}-H and C_{20}-H of **11** was replaced by deuterium, respectively. **e** The proposed mechanism of deuterium-hydrogen exchange in AspoA-catalysed double bond isomerization of **7** to **11.**

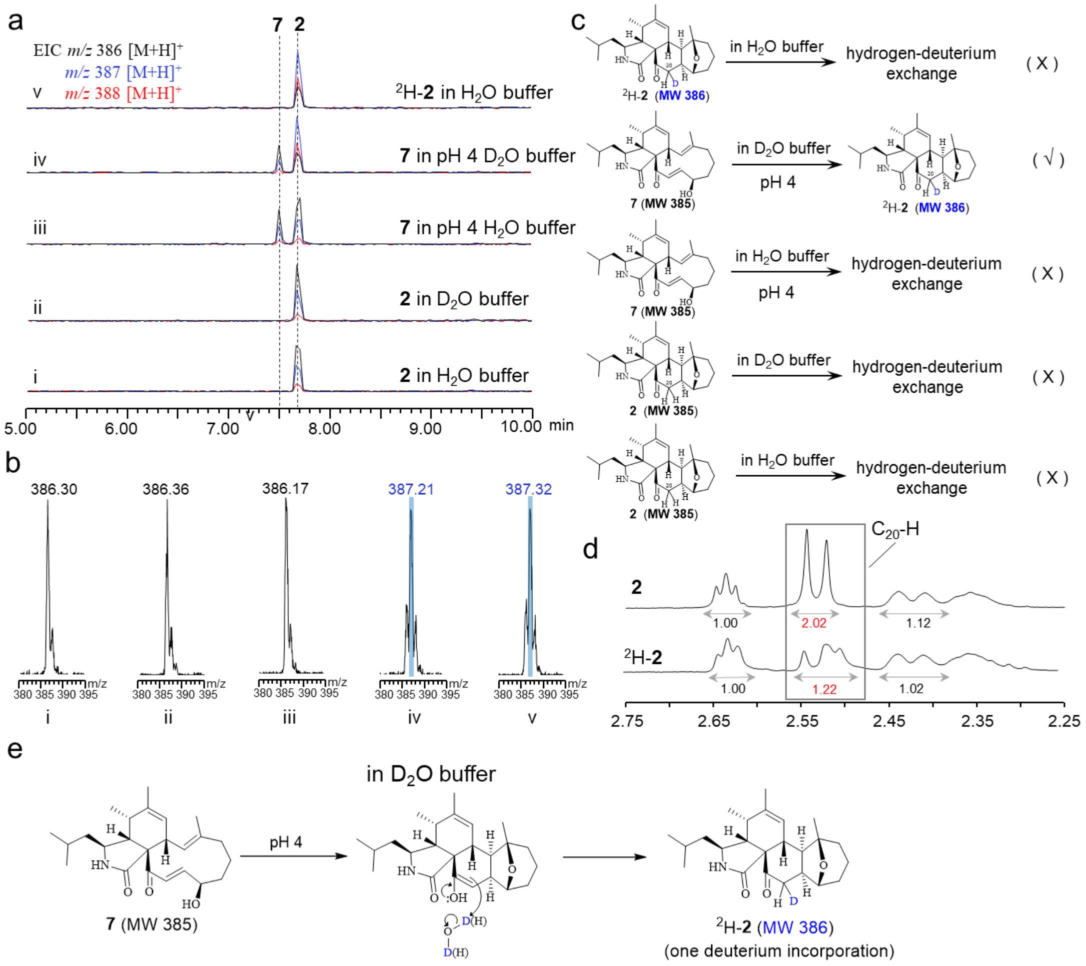

Supplementary Figure 15. The spontaneous conversion of **7** to **2** in D_2O buffer. **a** The spontaneous conversion of **7** to ^{2}H-**2** in pH 4 D_2O buffer. **b** The MS data of **2** or ^{2}H-**2** in different buffer (corresponding to **a**). **c** The deuterium-hydrogen exchange occurs in spontaneous conversion of **7** to **2** in pH 4 D_2O buffer. **d** The partial ^{1}H NMR spectrums of **2** and ^{2}H-**2** show that one deuterium is incorporated into C_{20}. In comparison with **2**, the C_{20}-H signals of ^{2}H-**2** decreased suggesting one of the C_{20}-H of **2** was replaced by deuterium. **e** The proposed mechanism of deuterium-hydrogen exchange in spontaneous conversion of **7** to **2**.

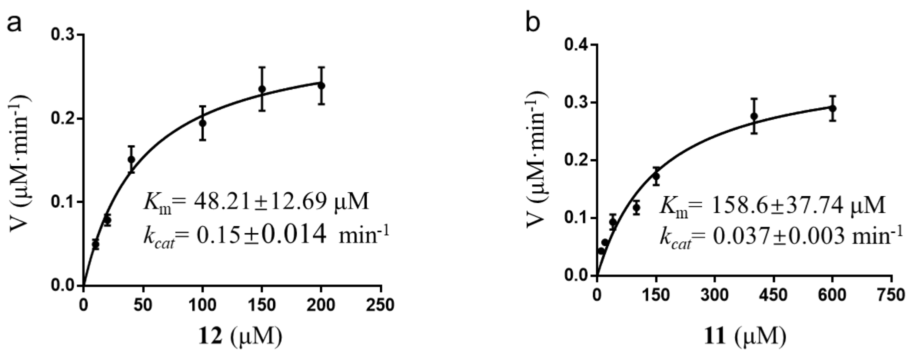

Supplementary Figure 16. The kinetic parameters of AspoD toward **12** (**a**) and **11** (**b**). These values were calculated by analyzing production of **14** and **13** by LC-MS analysis. This measurement was conducted in five times. K_M and k_{cat} values represent the mean ± SEM.

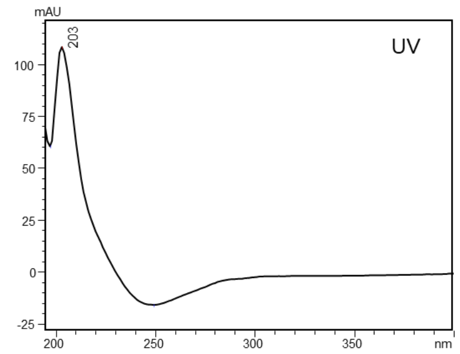

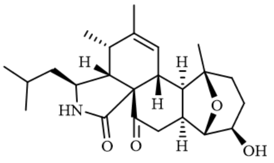

1

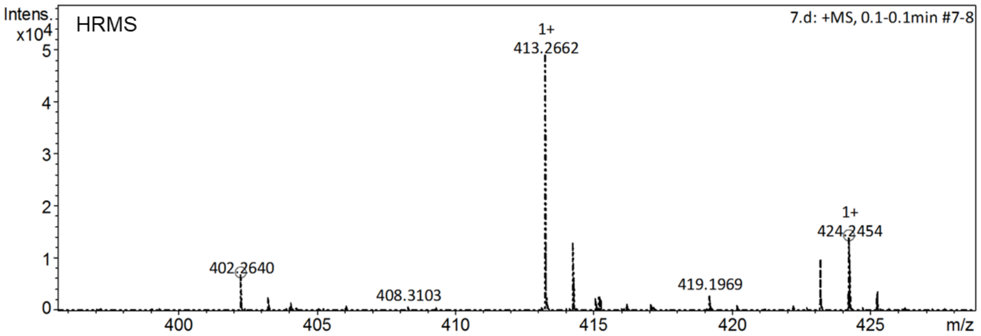

Meas. m/z	#	Ion Formula	Sum Formula	m/z	Adduct	err [ppm]	z	mSigma	Score	rdb	N-Rule	e⁻ Conf
402.2640	1	C24H36NO4	C24H35NO4	402.2639	M+H	-0.4	1+	25.0	100.00	8.0	ok	even
424.2454	1	C24H35NNaO4		424.2458	M+Na	1.0	1+	20.7	100.00	8.0	ok	even

Supplementary Figure 17. UV absorption and HRMS spectrum (positive ionization) of compound **1**.

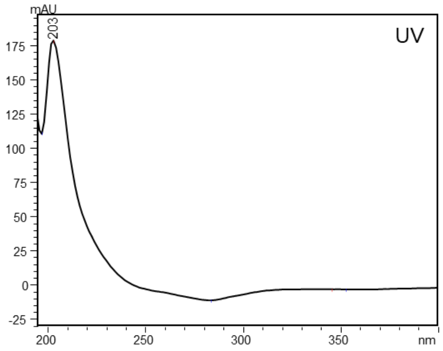

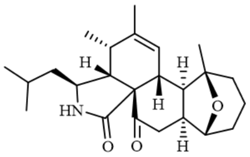

2

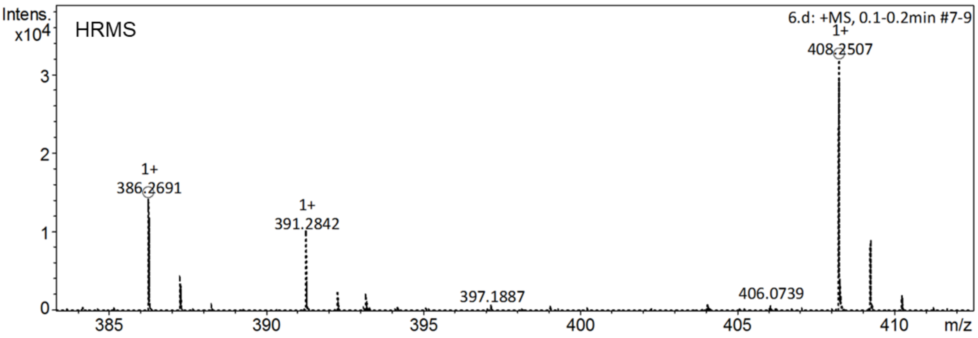

Meas. m/z	#	Ion Formula	Sum Formula	m/z	Adduct	err [ppm]	z	mSigma	Score	rdb	N-Rule	e⁻ Conf
386.2691	1	C24H36NO3	C24H35NO3	386.2690	M+H	-0.3	1+	3.4	100.00	8.0	ok	even
408.2507	1	C24H35NNaO3		408.2509	M+Na	0.6	1+	4.2	100.00	8.0	ok	even

Supplementary Figure 18. UV absorption and HRMS spectrum (positive ionization) of compound **2**.

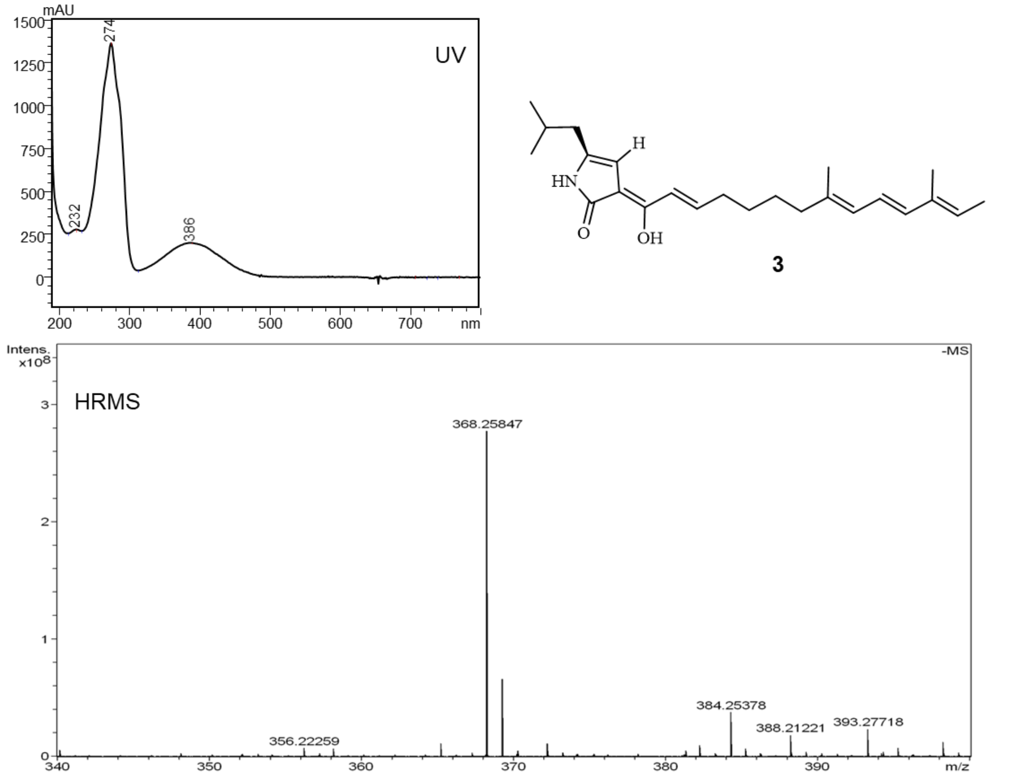

Meas. m/z	#	Formula	Score	m/z	err [ppm]	Mean err [ppm]	mSigma	rdb	e⁻ Conf	N-Rule
368.25847	1	C 24 H 34 N O 2	100.00	368.25950	2.80	2.60	20.3	8.5	even	ok

Supplementary Figure 19. UV absorption and HRMS spectrum (negative ionization) of compound **3**.

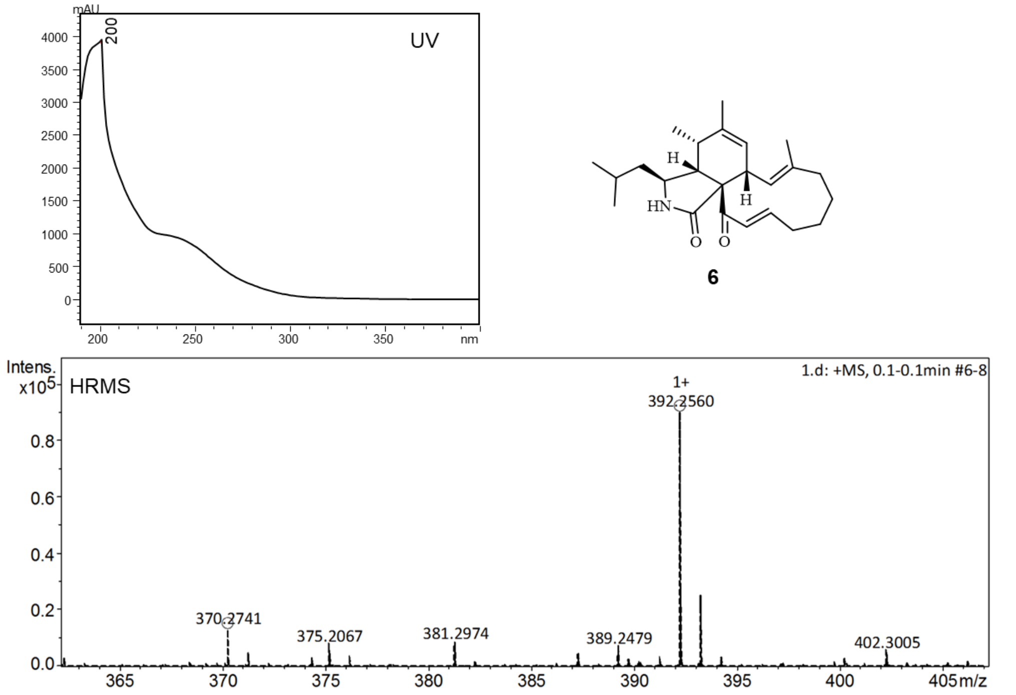

Meas. m/z	#	Ion Formula	Sum Formula	m/z	Adduct	err [ppm]	z	mSigma	Score	rdb	N-Rule	e⁻ Conf
370.2741	1	C24H36NO2	C24H35NO2	370.2741	M+H	-0.1	1+	5.0	100.00	8.0	ok	even
392.2560	1	C24H35NNaO2		392.2560	M+Na	-0.0	1+	2.8	100.00	8.0	ok	even

Supplementary Figure 20. UV absorption and HRMS spectrum (positive ionization) of compound **6**.

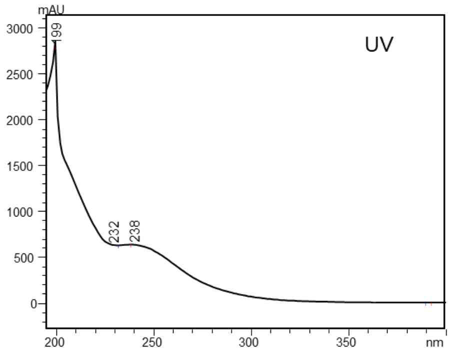

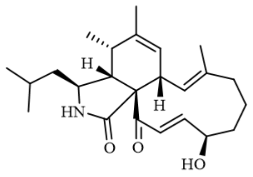

7

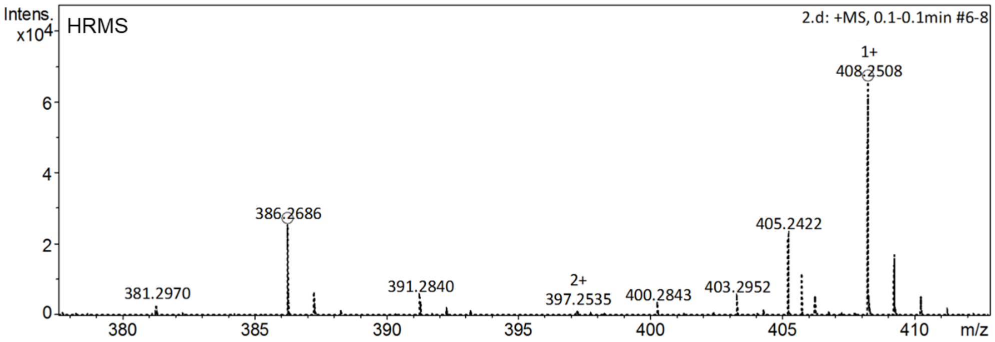

Meas. m/z	#	Ion Formula	Sum Formula	m/z	Adduct	err [ppm]	z	mSigma	Score	rdb	N-Rule	e⁻ Conf
386.2686	1	C24H36NO3	C24H35NO3	386.2690	M+H	0.9	1+	3.7	100.00	8.0	ok	even
408.2508	1	C24H35NNaO3		408.2509	M+Na	0.2	1+	24.4	100.00	8.0	ok	even

Supplementary Figure 21. UV absorption and HRMS spectrum (positive ionization) of compound **7**.

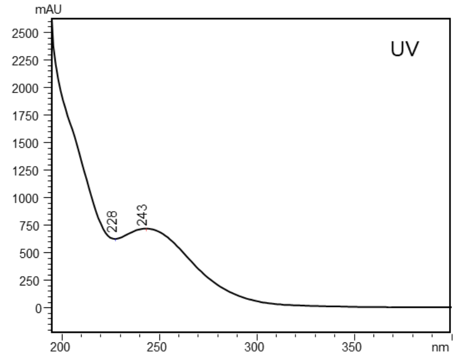

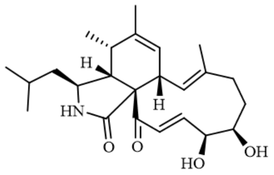

8

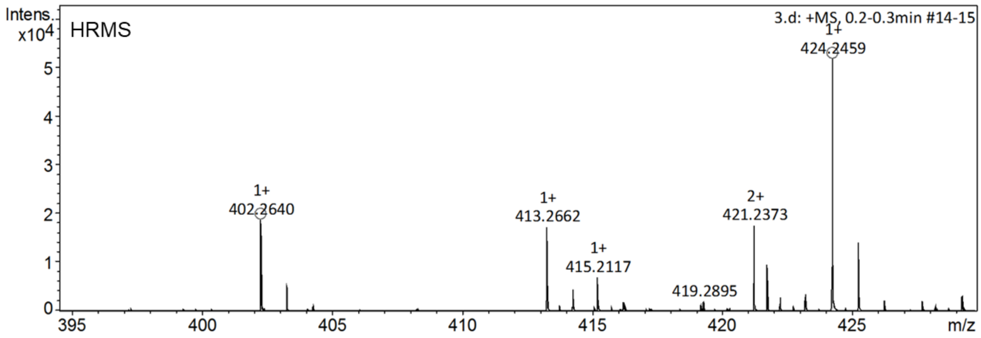

Meas. m/z	#	Ion Formula	Sum Formula	m/z	Adduct	err [ppm]	z	mSigma	Score	rdb	N-Rule	e⁻ Conf
402.2640	1	C24H36NO4	C24H35NO4	402.2639	M+H	-0.4	1+	3.1	100.00	8.0	ok	even
424.2459	1	C24H35NNaO4		424.2458	M+Na	-0.2	1+	1.8	100.00	8.0	ok	even

Supplementary Figure 22. UV absorption and HRMS spectrum (positive ionization) of compound **8**.

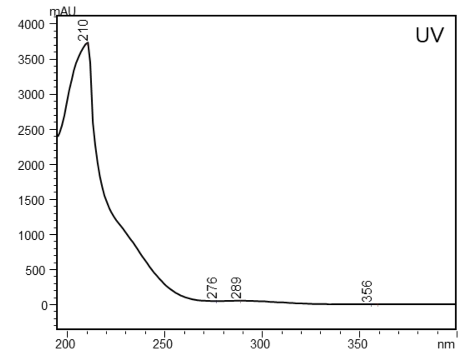

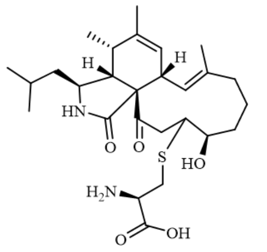

9

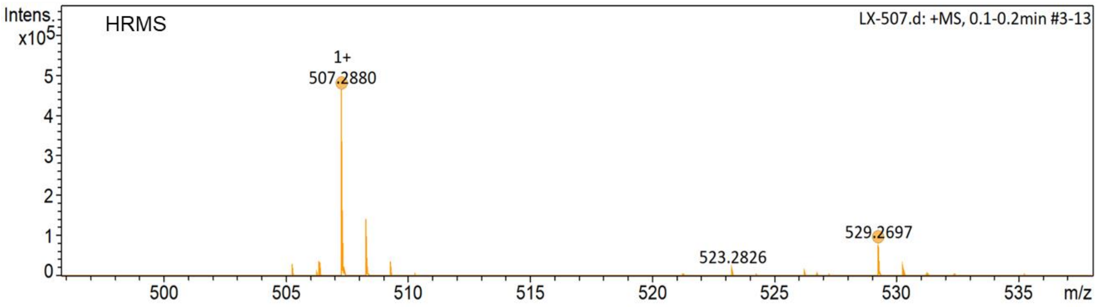

Meas. m/z	#	Ion Formula	Sum Formula	m/z	Adduct	err [ppm]	z	mSigma	Score	rdb	N-Rule	e¡¥ Conf
507.2880	1	C27H43N2O5S	C27H42N2O5S	507.2887	M+H	1.5	1+	11.3	100.00	8.0	ok	even
529.2697	1	C27H42N2NaO5S		529.2707	M+Na	1.8	1+	10.9	100.00	8.0	ok	even

Supplementary Figure 23. UV absorption and HRMS spectrum (positive ionization) of compound **9**.

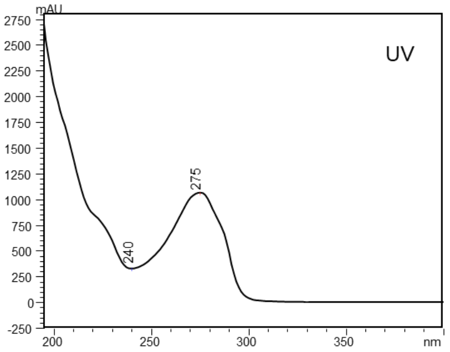

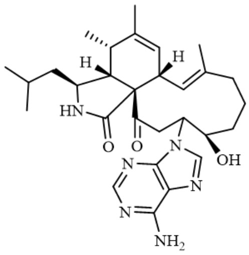

10

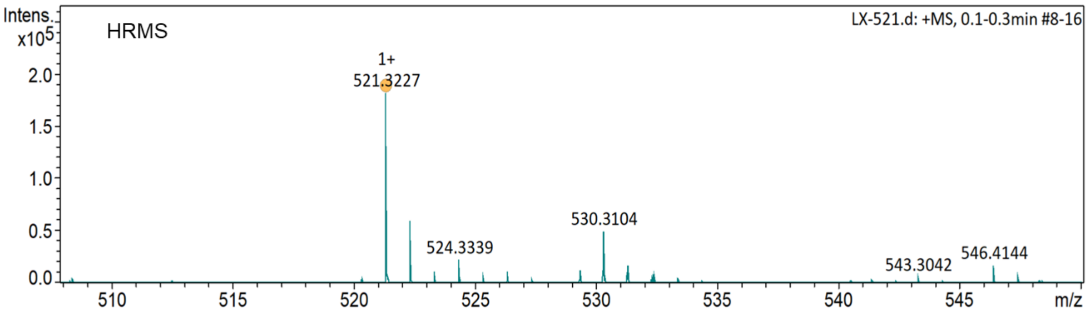

Meas. m/z	#	Ion Formula	Sum Formula	m/z	Adduct	err [ppm]	z	mSigma	Score	rdb	N-Rule	e¡¥ Conf
521.3227	1	C29H41N6O3	C29H40N6O3	521.3235	M+H	1.4	1+	57.7	100.00	13.0	ok	even

Supplementary Figure 24. UV absorption and HRMS spectrum (positive ionization) of compound **10**.

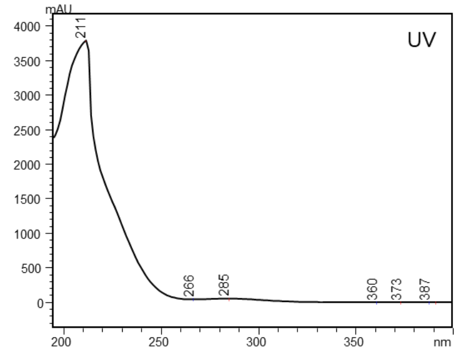

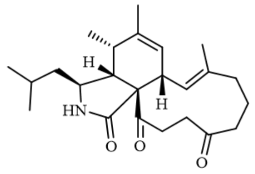

11

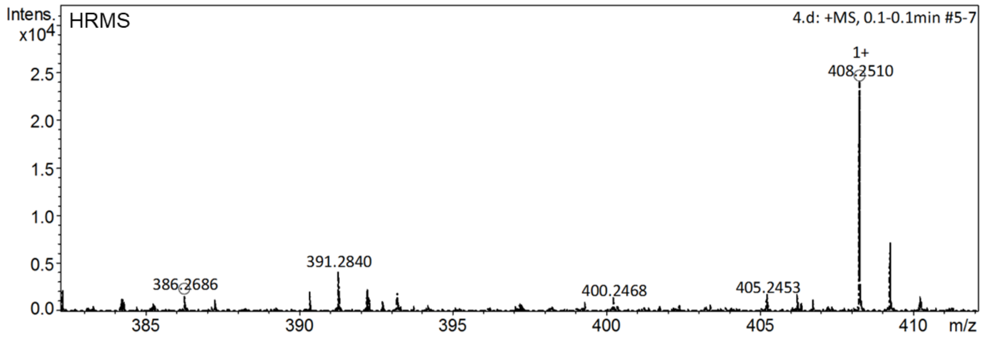

Meas. m/z	#	Ion Formula	Sum Formula	m/z	Adduct	err [ppm]	z	mSigma	Score	rdb	N-Rule	e⁻ Conf
386.2686	1	C24H36NO3	C24H35NO3	386.2690	M+H	0.9	1+	110.1	100.00	8.0	ok	even
408.2510	1	C24H35NNaO3		408.2509	M+Na	-0.3	1+	16.3	100.00	8.0	ok	even

Supplementary Figure 25. UV absorption and HRMS spectrum (positive ionization) of compound **11**.

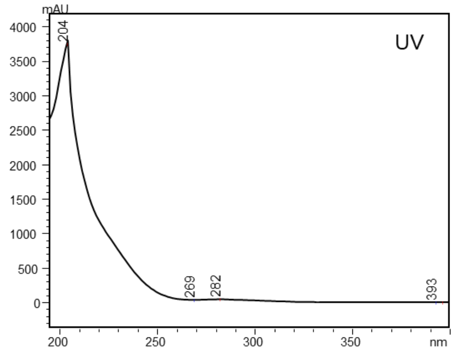

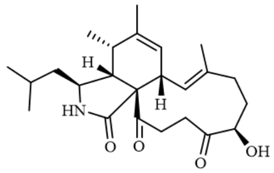

12

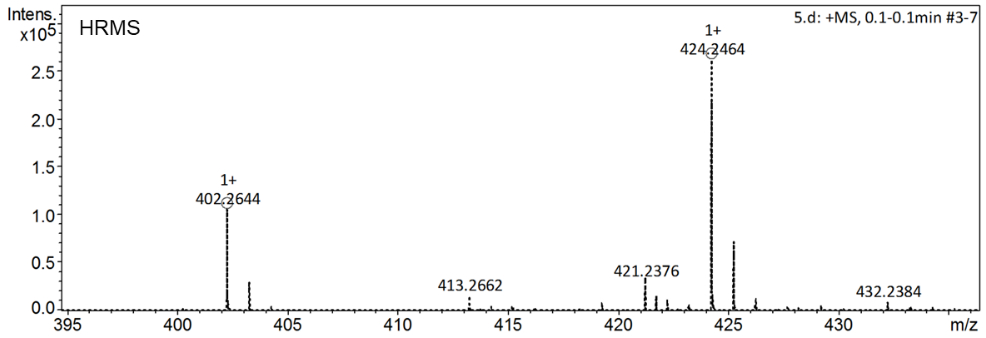

Meas. m/z	#	Ion Formula	Sum Formula	m/z	Adduct	err [ppm]	z	mSigma	Score	rdb	N-Rule	e⁻ Conf
402.2644	1	C24H36NO4	C24H35NO4	402.2639	M+H	-1.2	1+	7.4	100.00	8.0	ok	even
424.2464	1	C24H35NNaO4		424.2458	M+Na	-1.3	1+	4.0	100.00	8.0	ok	even

Supplementary Figure 26 UV absorption and HRMS spectrum (positive ionization) of compound **12**.

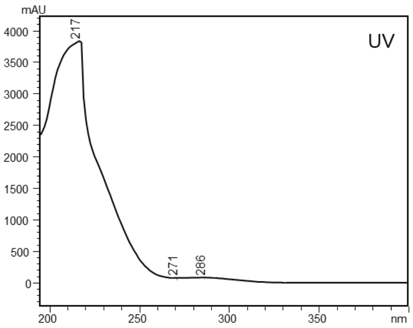

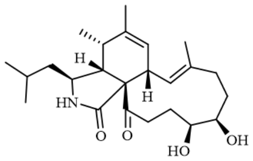

14

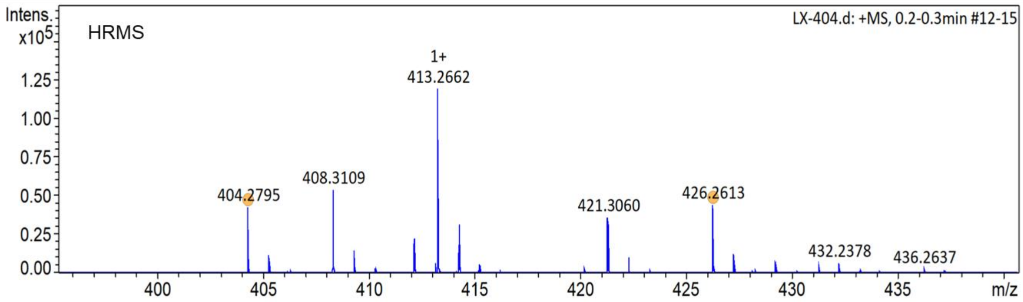

Meas. m/z	#	Ion Formula	Sum Formula	m/z	Adduct	err [ppm]	z	mSigma	Score	rdb	N-Rule	e¡¥ Conf
404.2795	1	C24H38NO4	C24H37NO4	404.2795	M+H	0.0	1+	2.0	100.00	7.0	ok	even
426.2613	1	C24H37NNaO4		426.2615	M+Na	0.5	1+	2.0	100.00	7.0	ok	even

Supplementary Figure 27. UV absorption and HRMS spectrum (positive ionization) of compound **14**.

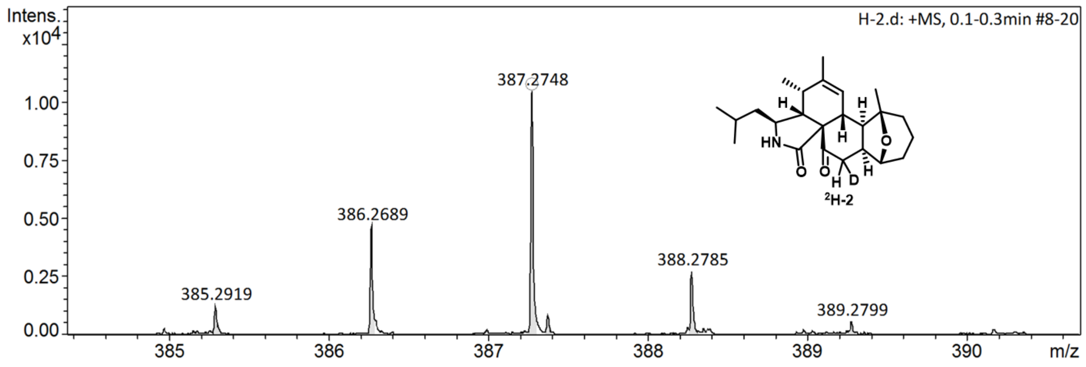

Meas. m/z	#	Ion Formula	Sum Formula	m/z	Adduct	err [ppm]	z	mSigma	Score	rdb	N-Rule	e⁻ Conf
387.2748	1	C24H35DNO3	C24H34DNO3	387.2752	M+H	1.3	1+	8.9	100.00	8.0	ok	even

Supplementary Figure 28. HRMS spectrum (positive ionization) of compound ^{2}H-**2**.

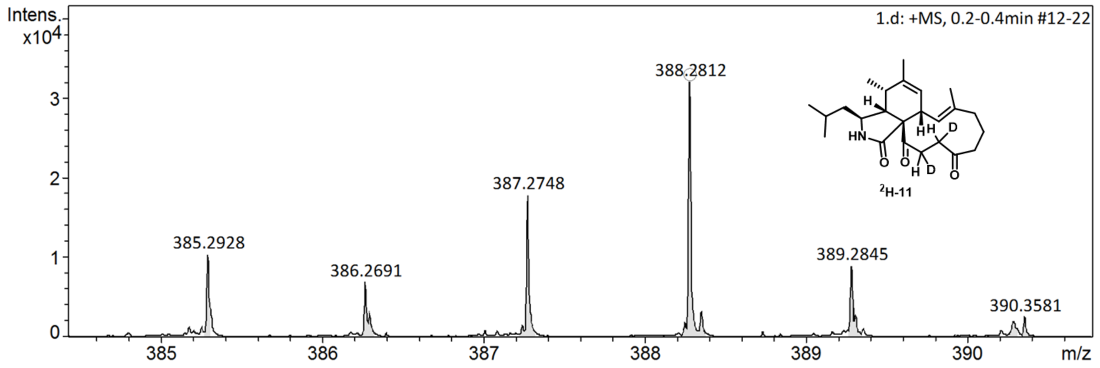

Meas. m/z	#	Ion Formula	Sum Formula	m/z	Adduct	err [ppm]	z	mSigma	Score	rdb	N-Rule	e⁻ Conf
388.2812	1	C24H34D2NO3	C24H33D2NO3	388.2815	M+H	0.8	1+	12.7	100.00	8.0	ok	even

Supplementary Figure 29. HRMS spectrum (positive ionization) of compound ^{2}H-**11**.

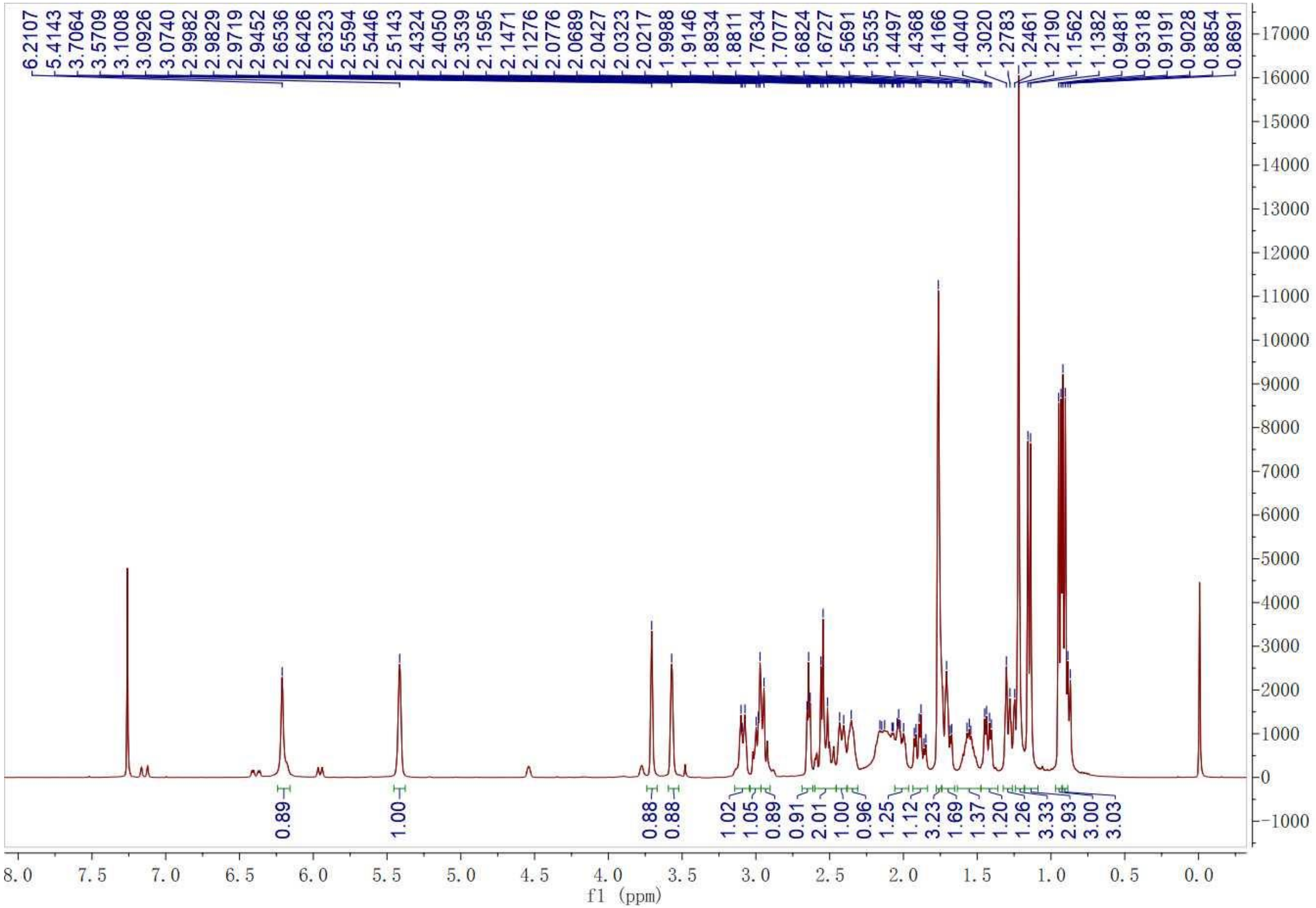

Supplementary Figure 30. ^{1}H NMR spectrum of compound **1** in $CDCl_3$ (400 MHz)

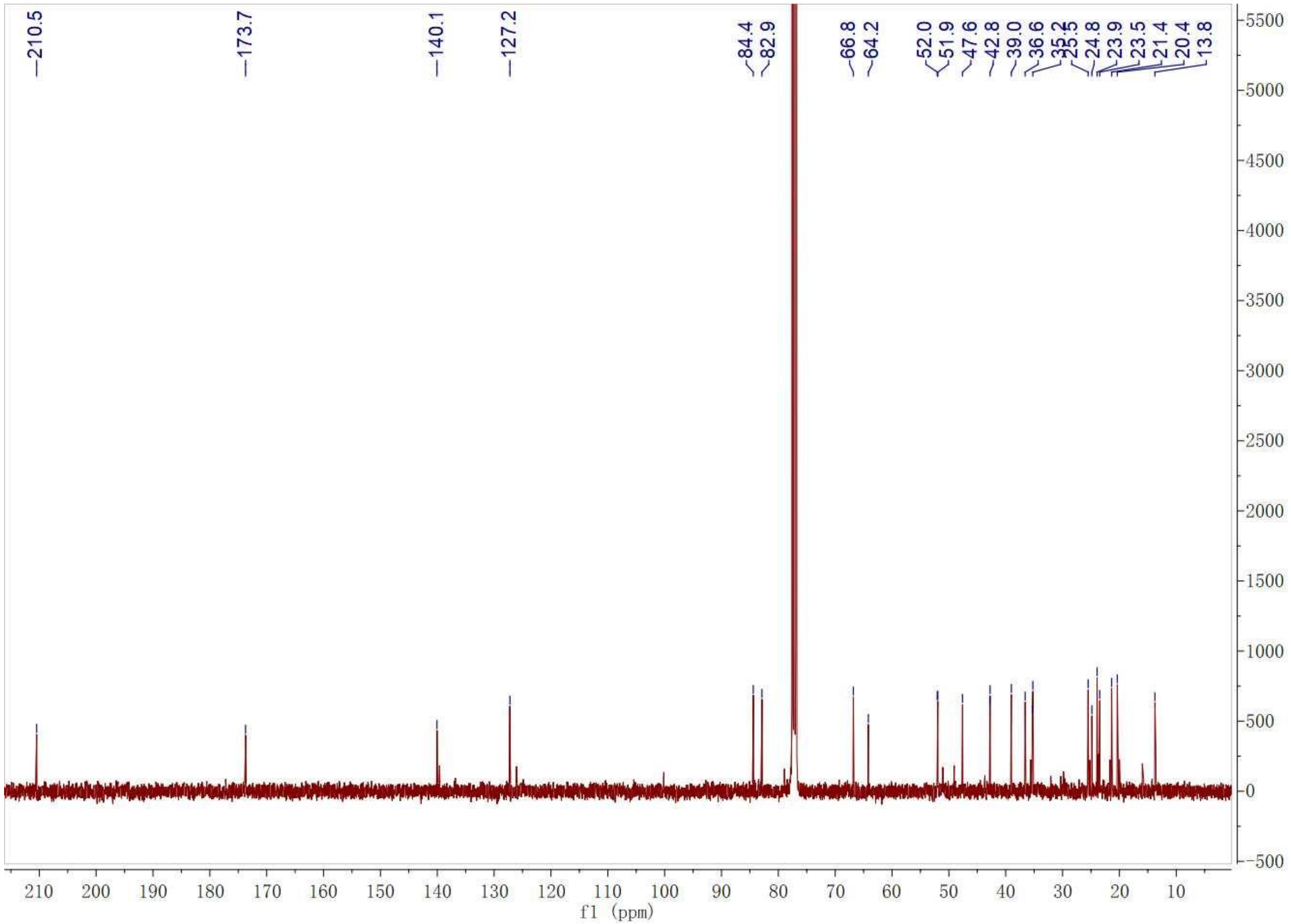

Supplementary Figure 31. ^{13}C NMR spectrum of compound **1** in $CDCl_3$ (100 MHz)

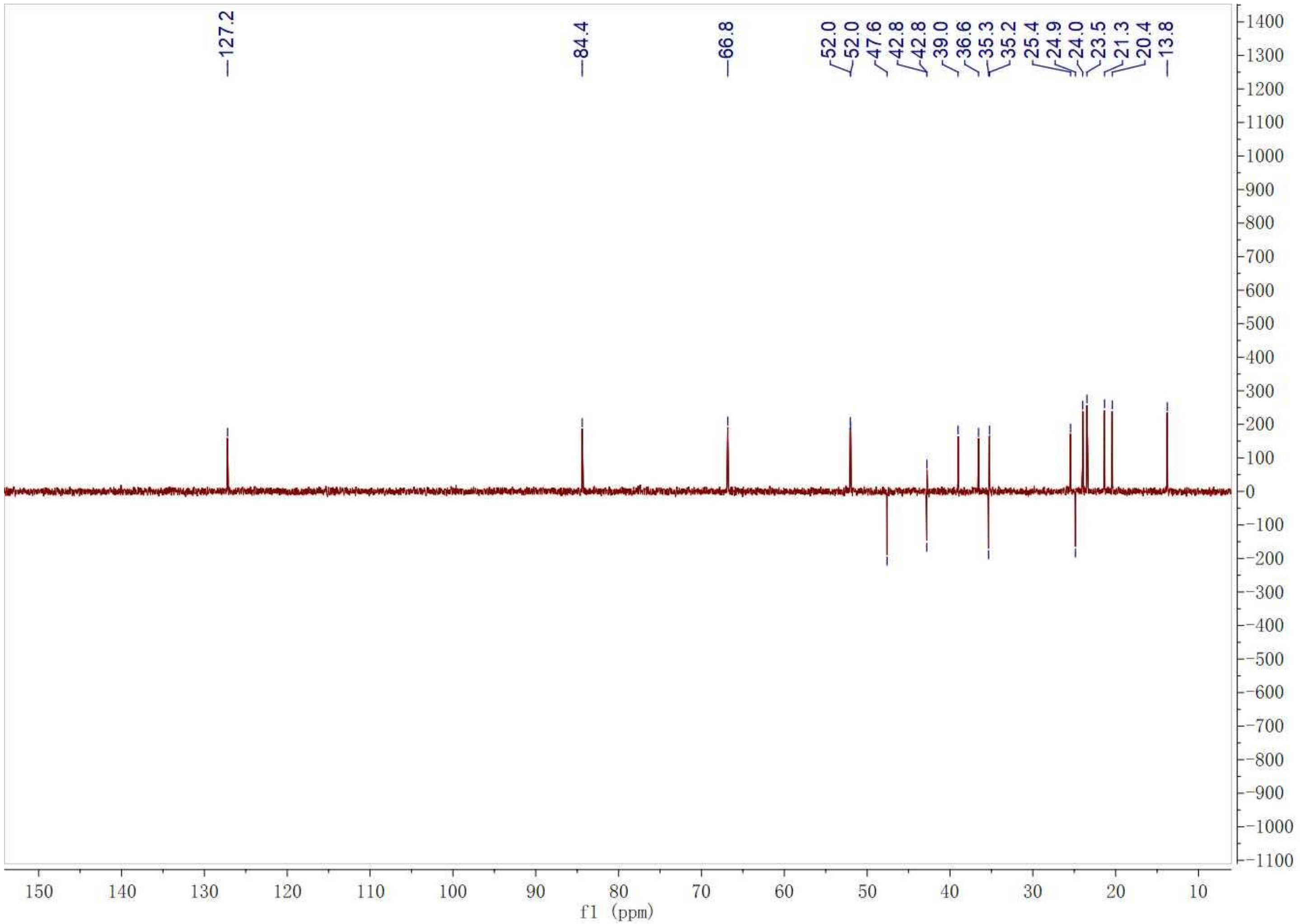

Supplementary Figure 32. DEPT-135° spectrum of compound **1** in $CDCl_3$.

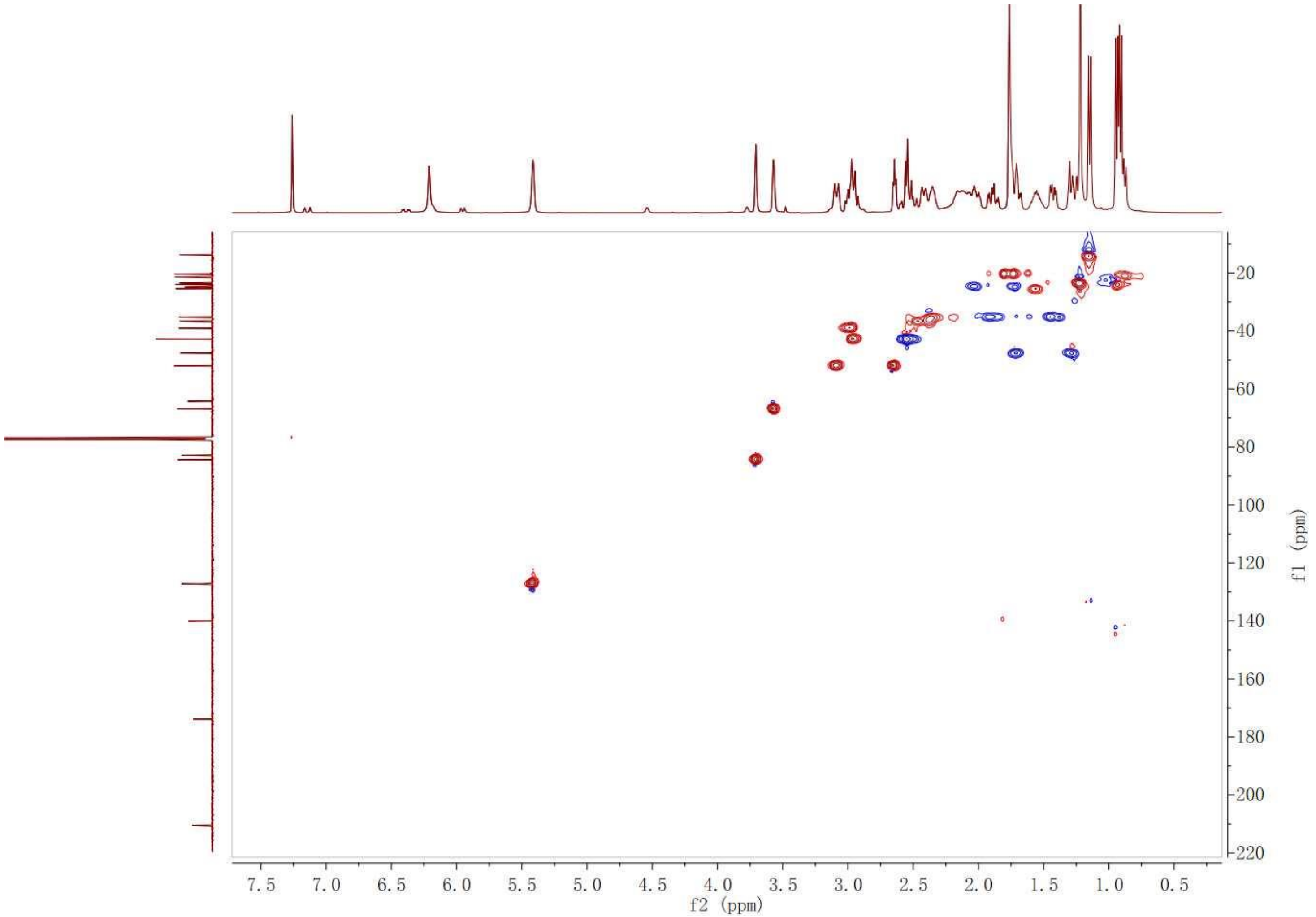

Supplementary Figure 33. HSQC spectrum of compound **1** in $CDCl_3$.

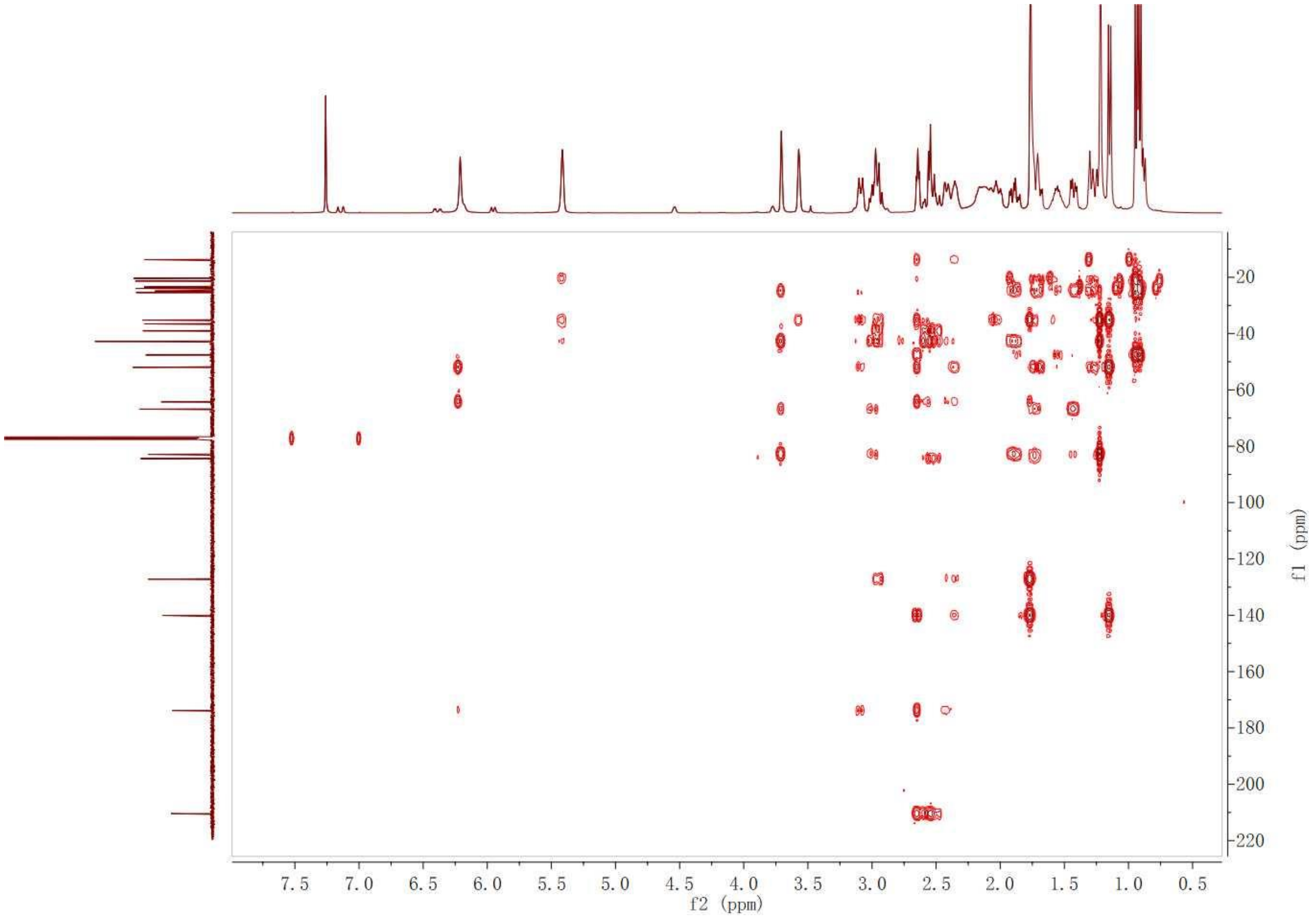

Supplementary Figure 34. HMBC spectrum of compound **1** in $CDCl_3$.

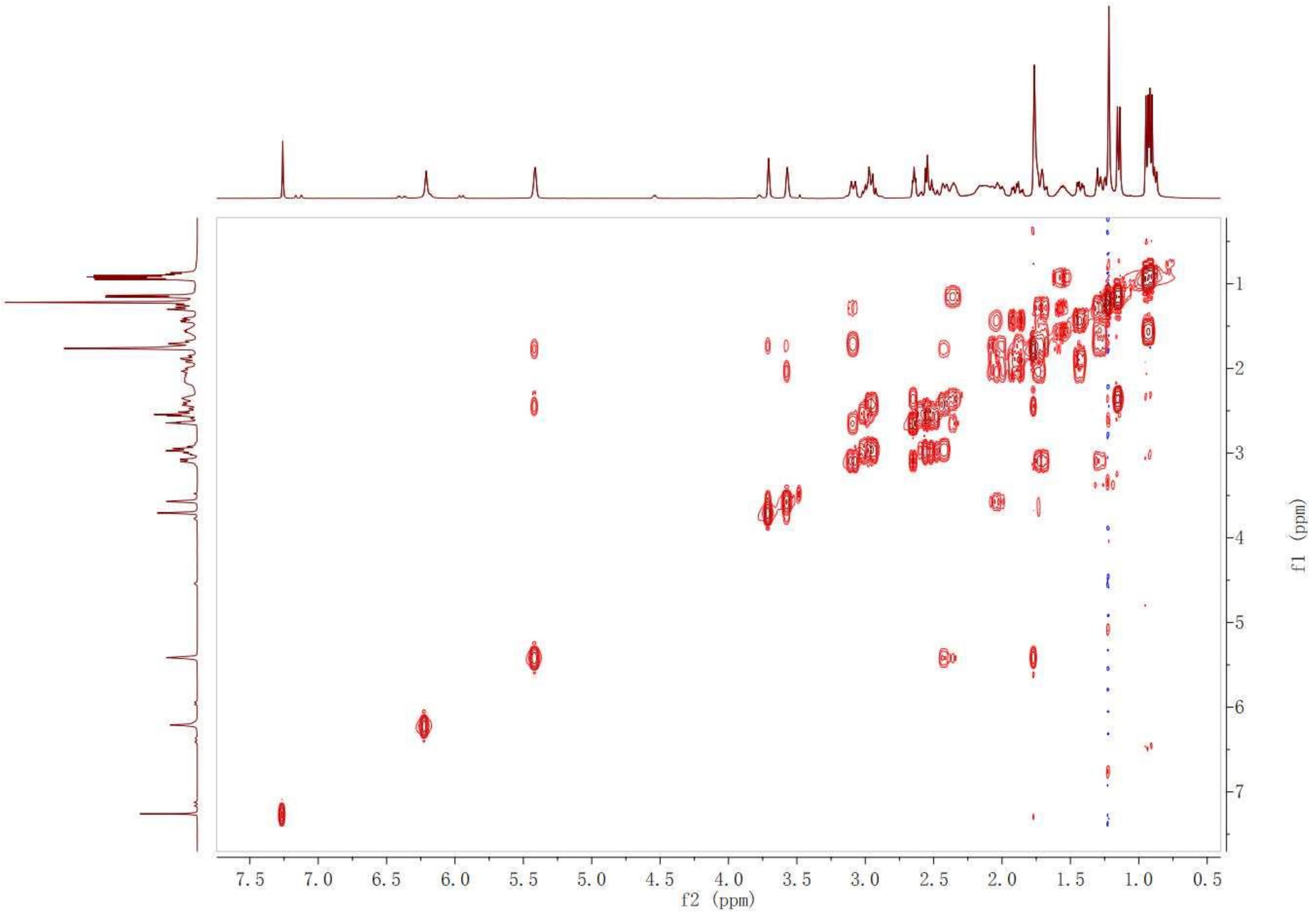

Supplementary Figure 35. ^{1}H-^{1}H COSY spectrum of compound **1** in $CDCl_3$.

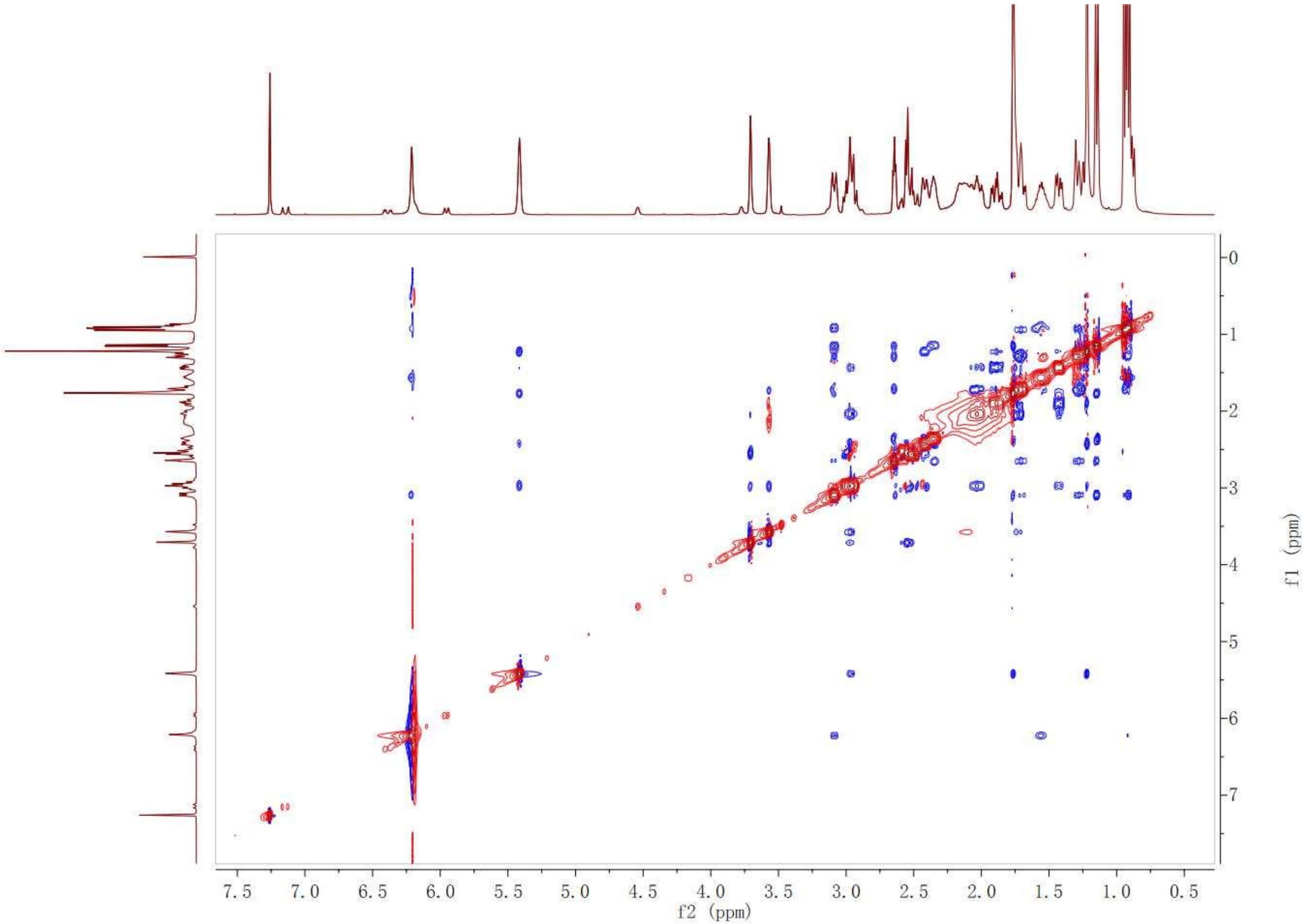

Supplementary Figure 36. ^1H-^1H NOESY spectrum of compound **1** in $CDCl_3$.

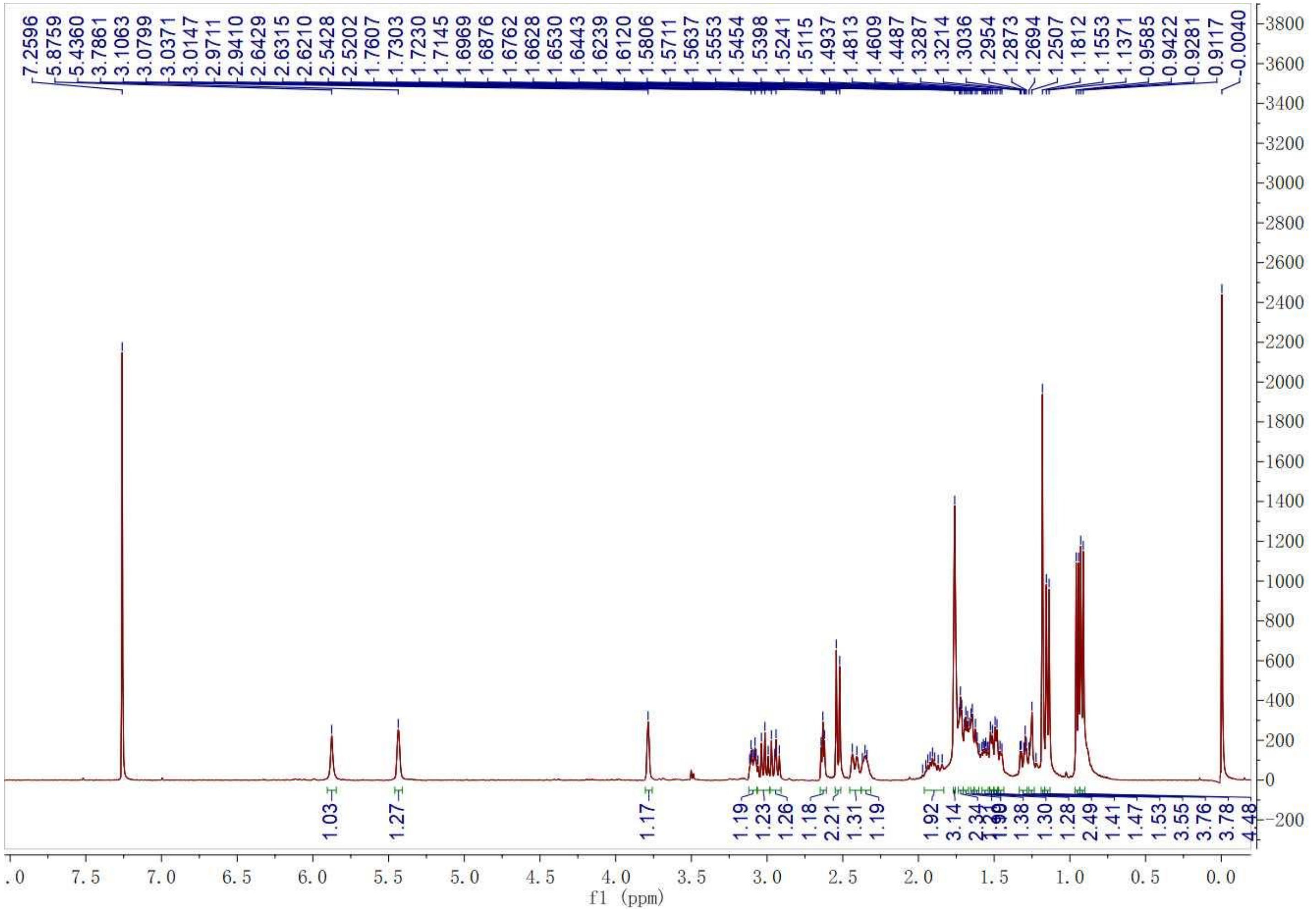

Supplementary Figure 37. ^{1}H NMR spectrum of compound **2** in $CDCl_3$ (400 MHz).

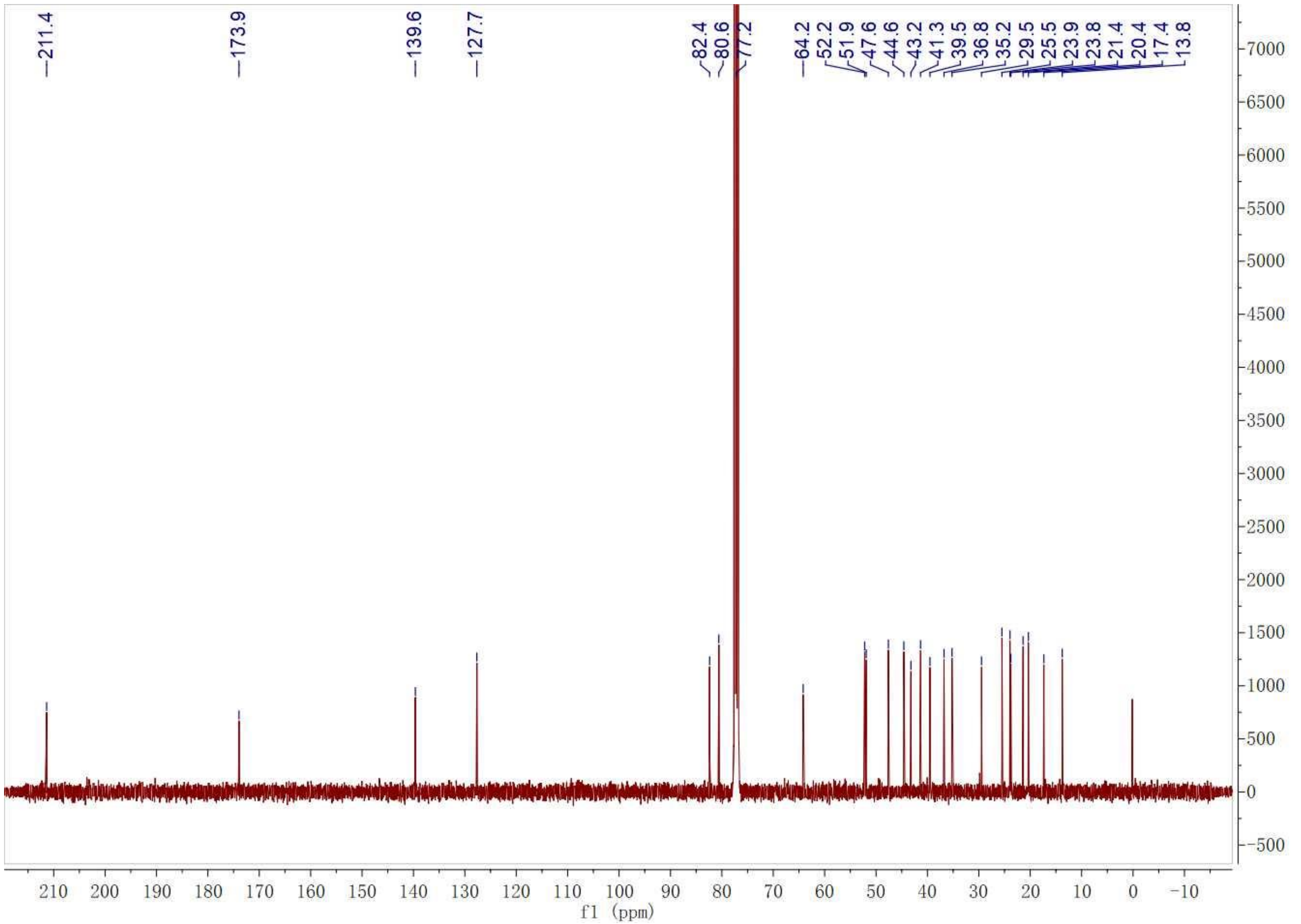

Supplementary Figure 38. ^{13}C NMR spectrum of compound **2** in $CDCl_3$ (100 MHz).

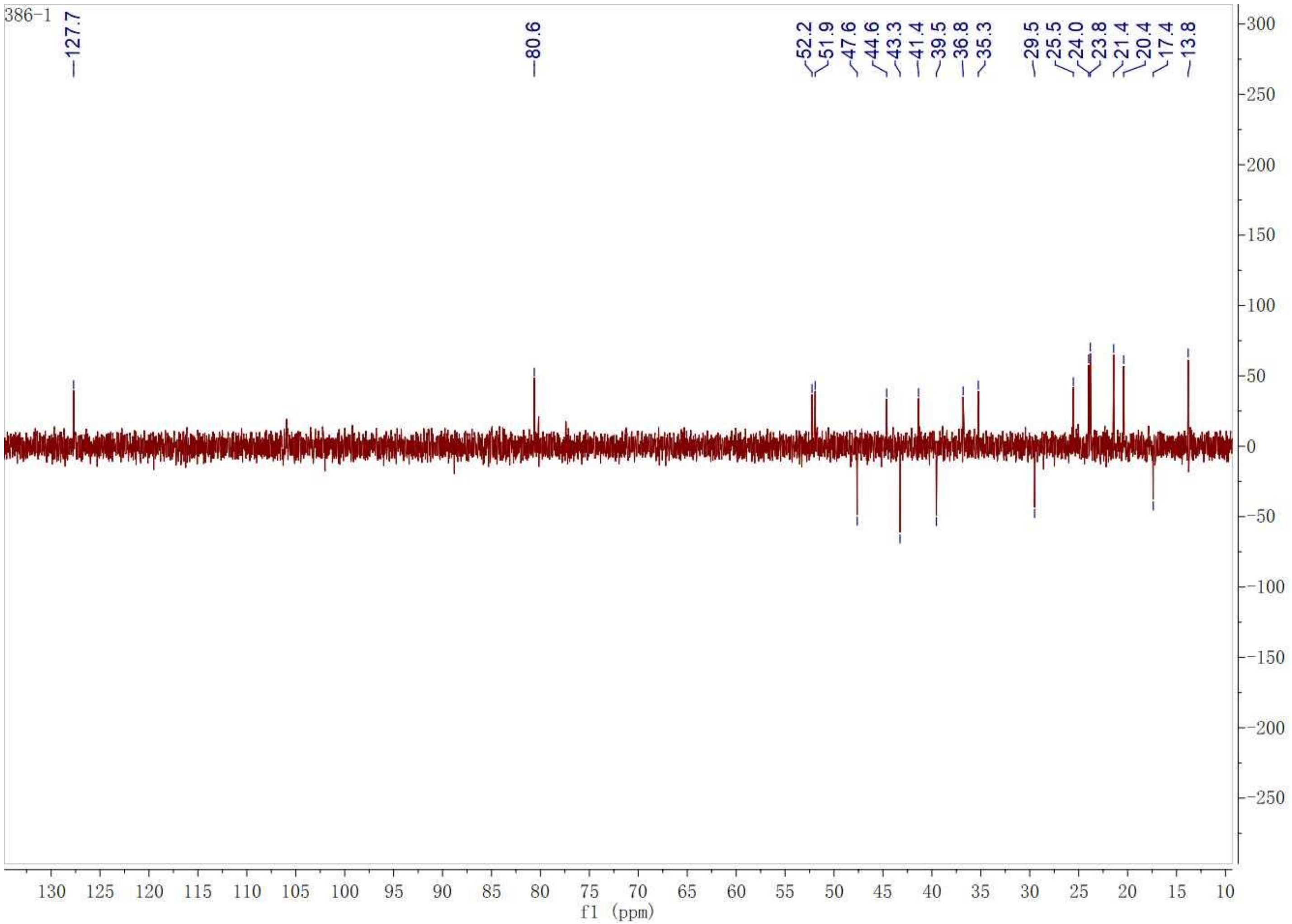

Supplementary Figure 39. DEPT-135° spectrum of compound **2** in $CDCl_3$.

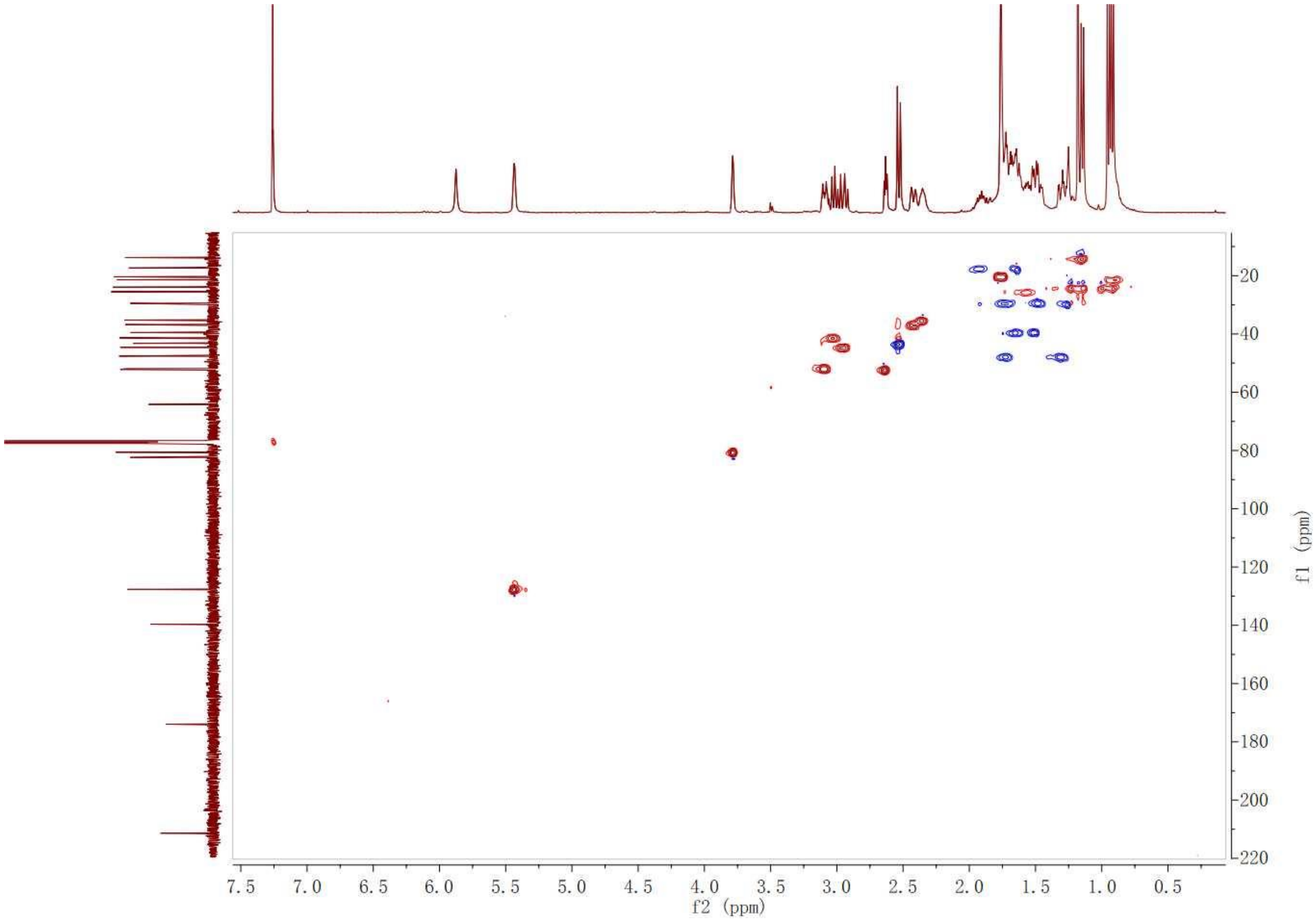

Supplementary Figure 40. HSQC spectrum of compound **2** in $CDCl_3$.

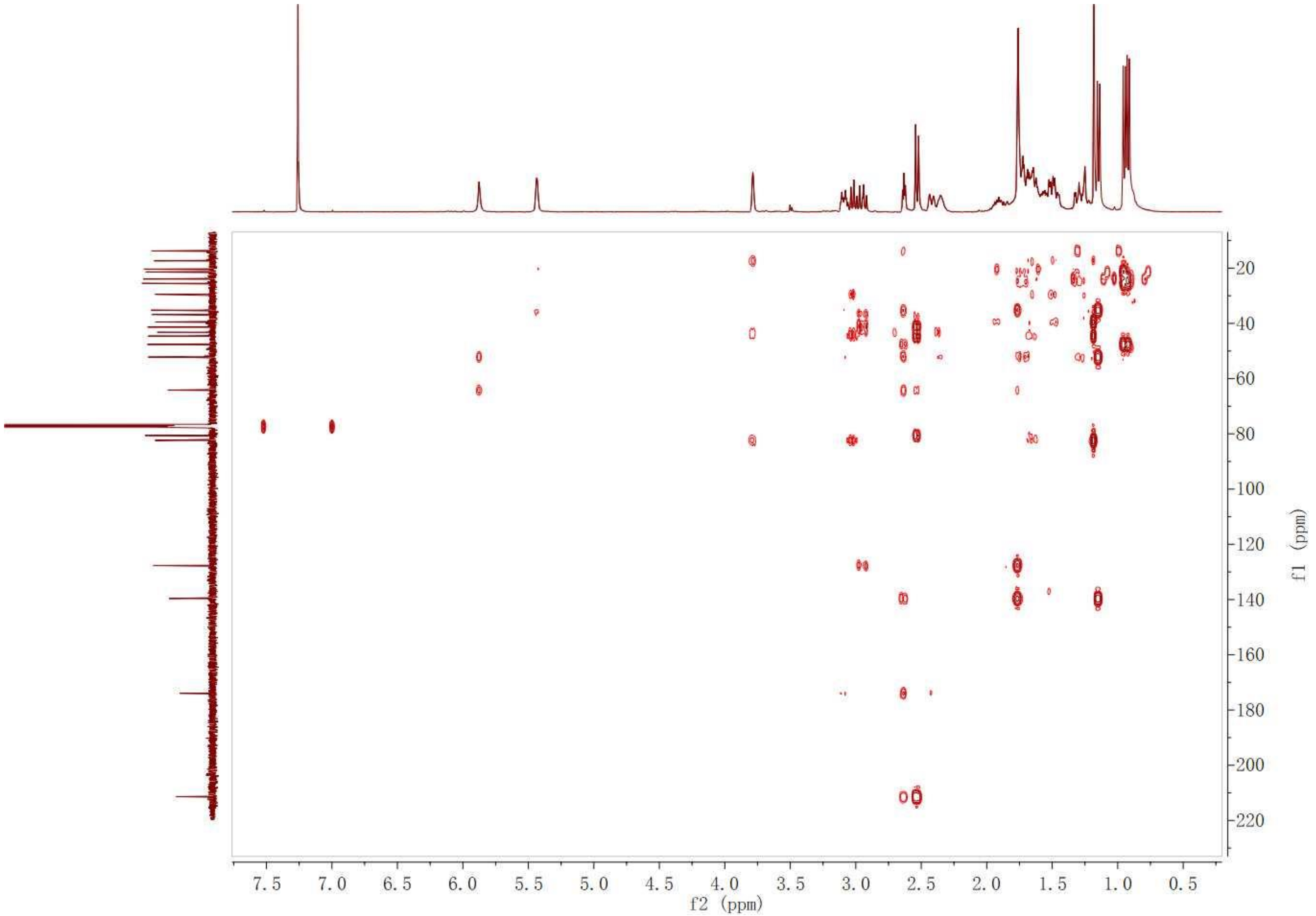

Supplementary Figure 41. HMBC spectrum of compound **2** in $CDCl_3$.

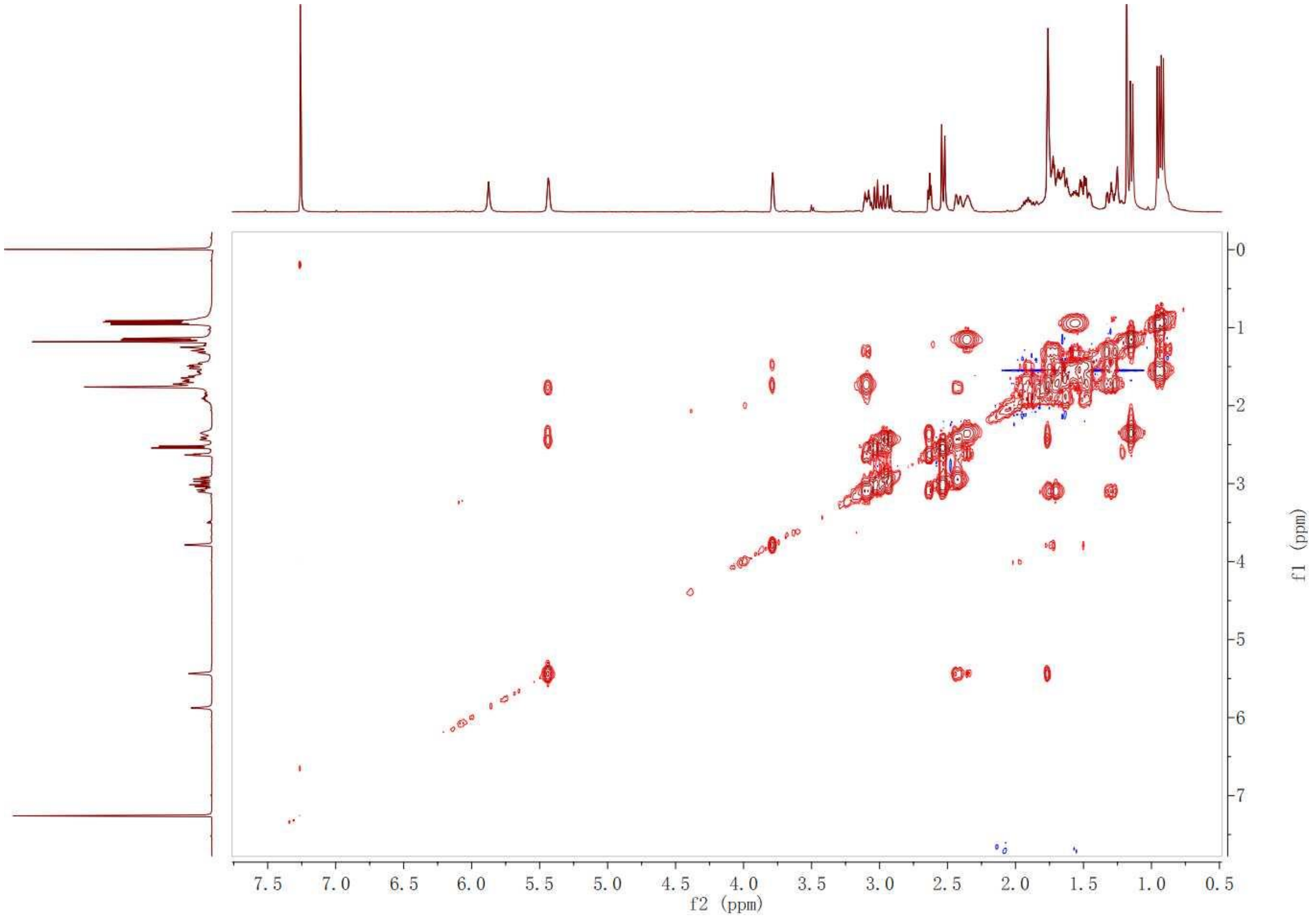

Supplementary Figure 42. ^{1}H-^{1}H COSY spectrum of compound **2** in $CDCl_3$.

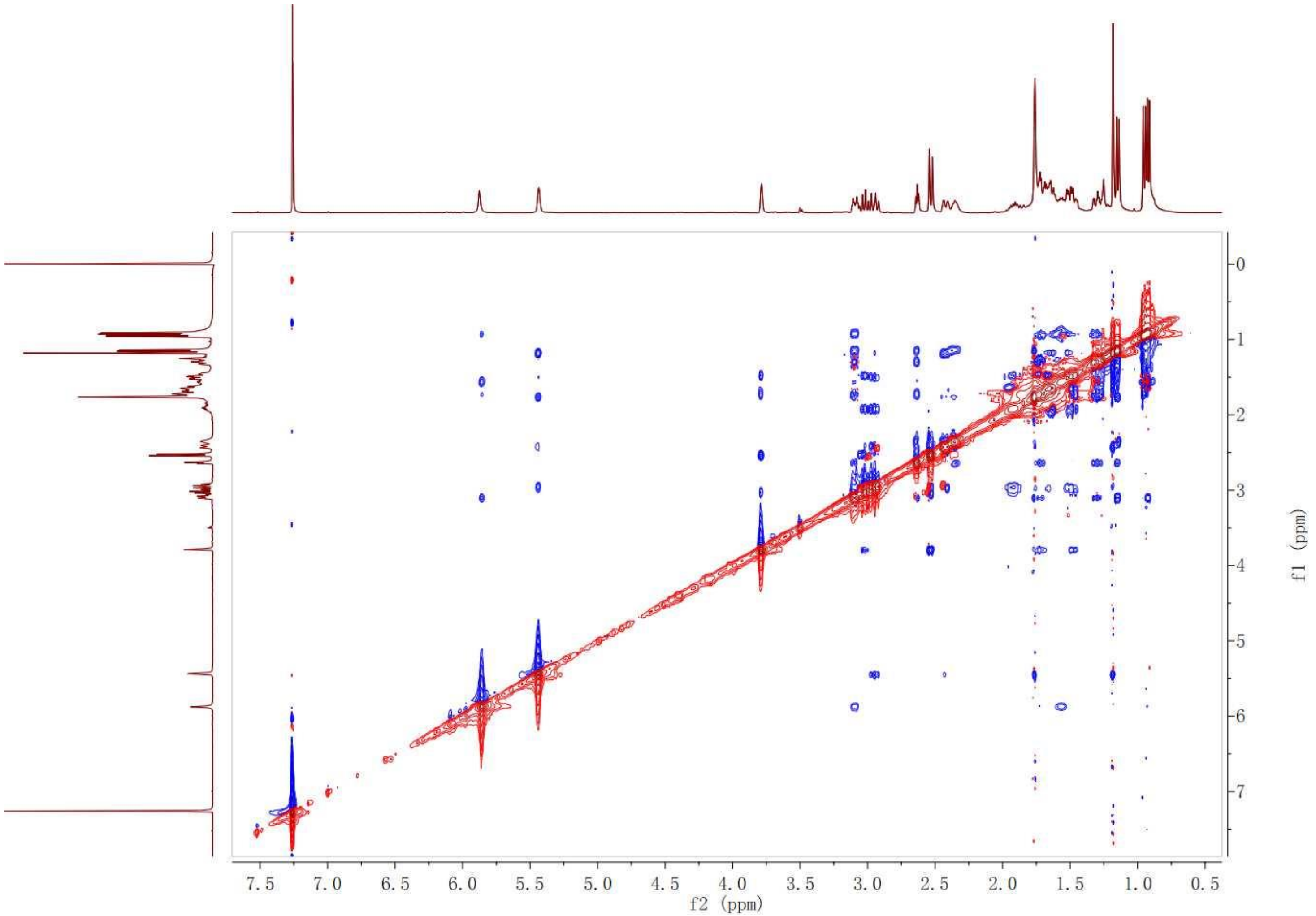

Supplementary Figure 43. ^{1}H-^{1}H NOESY spectrum of compound **2** in $CDCl_3$.

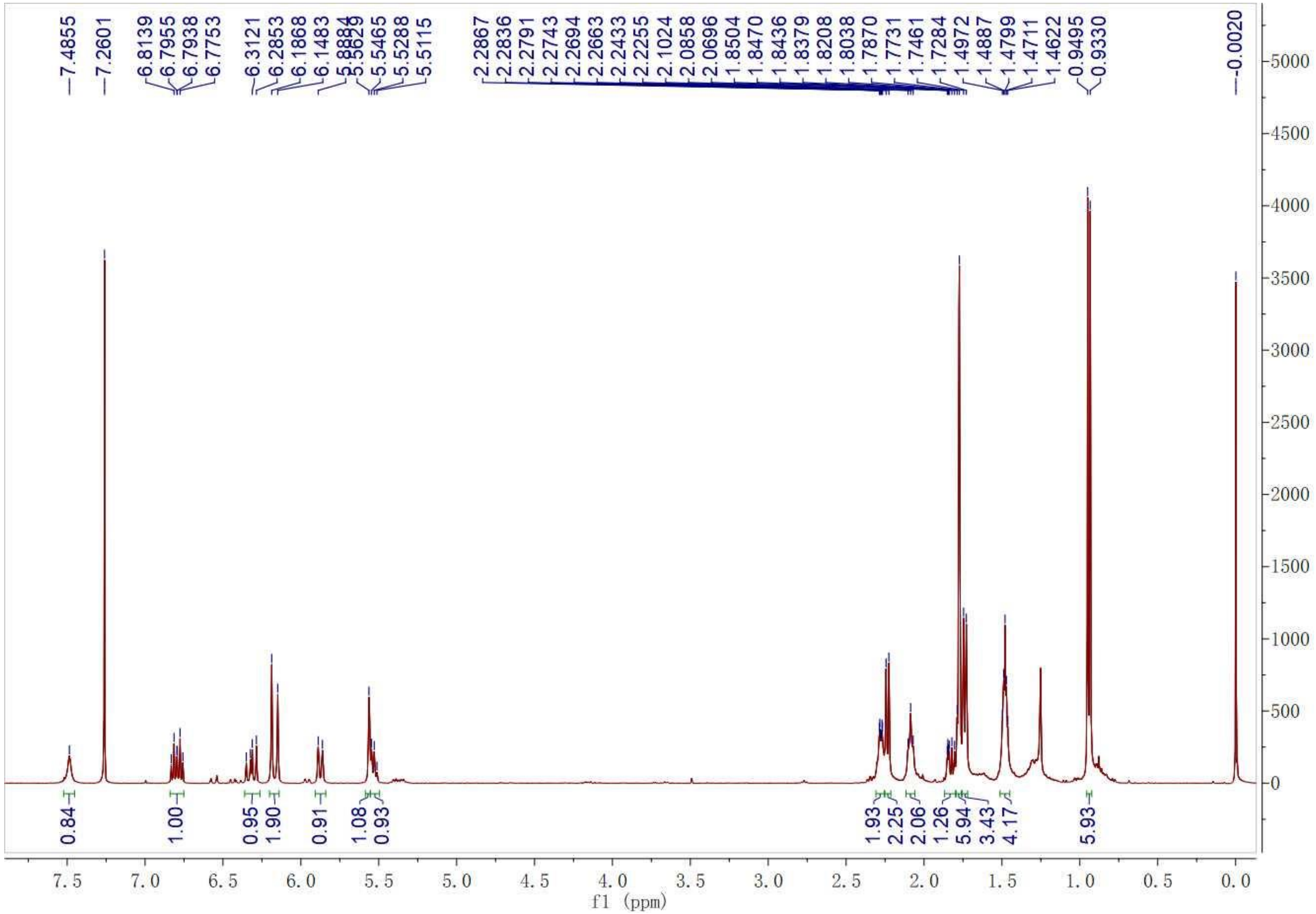

Supplementary Figure 44. ^{1}H NMR spectrum of compound **3** in $CDCl_3$ (400 MHz).

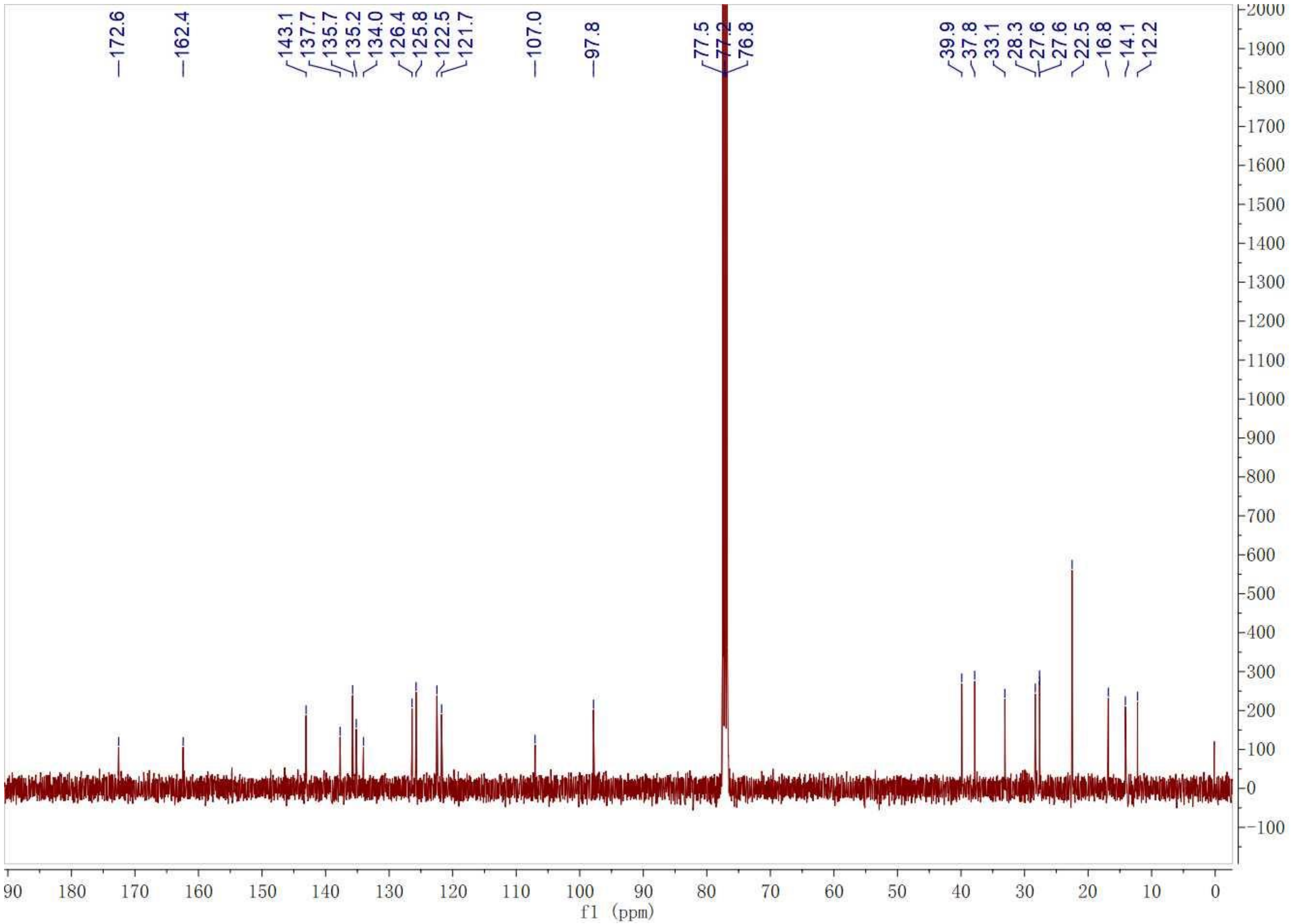

Supplementary Figure 45. ^{13}C NMR spectrum of compound **3** in $CDCl_3$ (100 MHz).

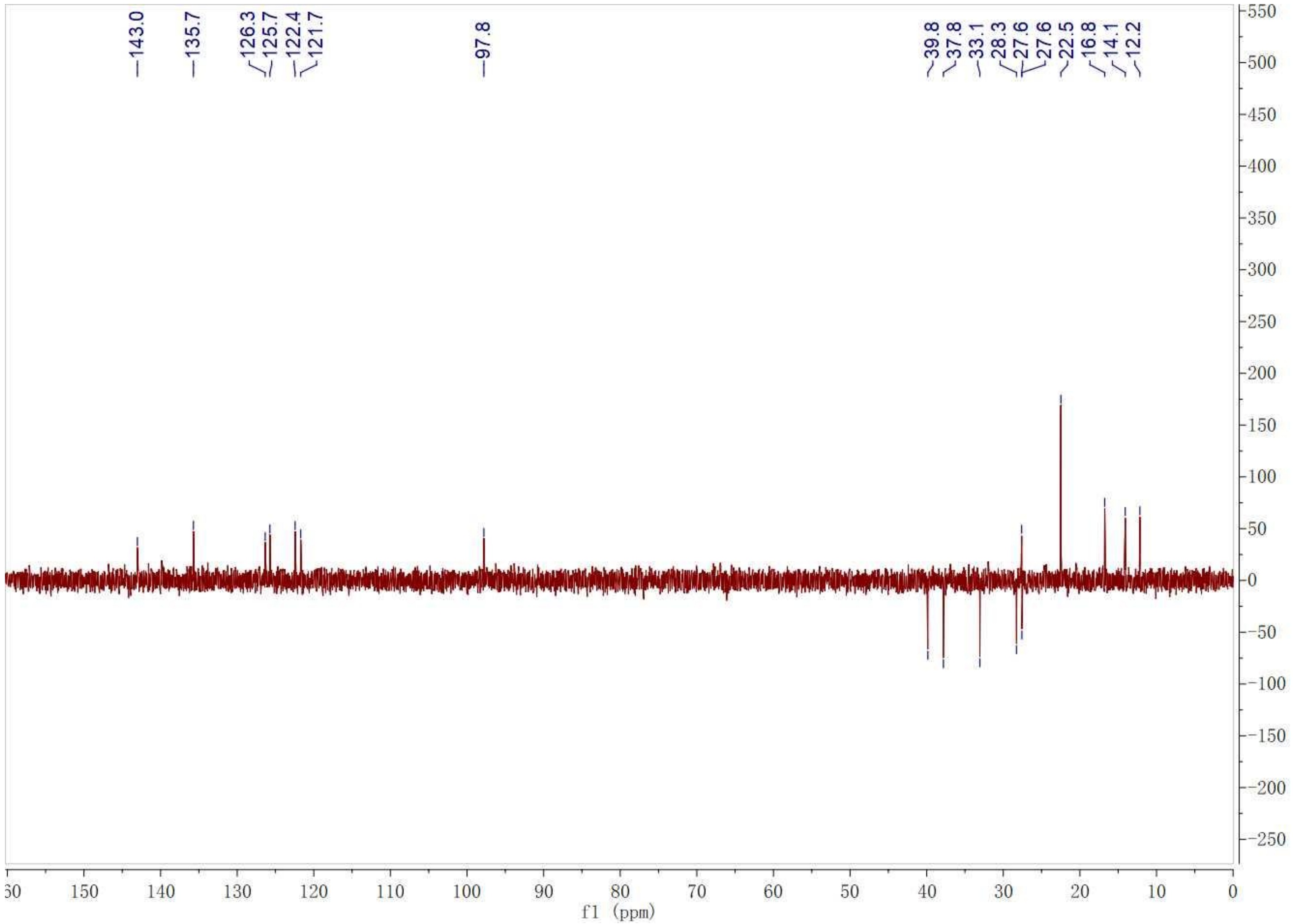

Supplementary Figure 46. DEPT-135° spectrum of compound **3** in $CDCl_3$.

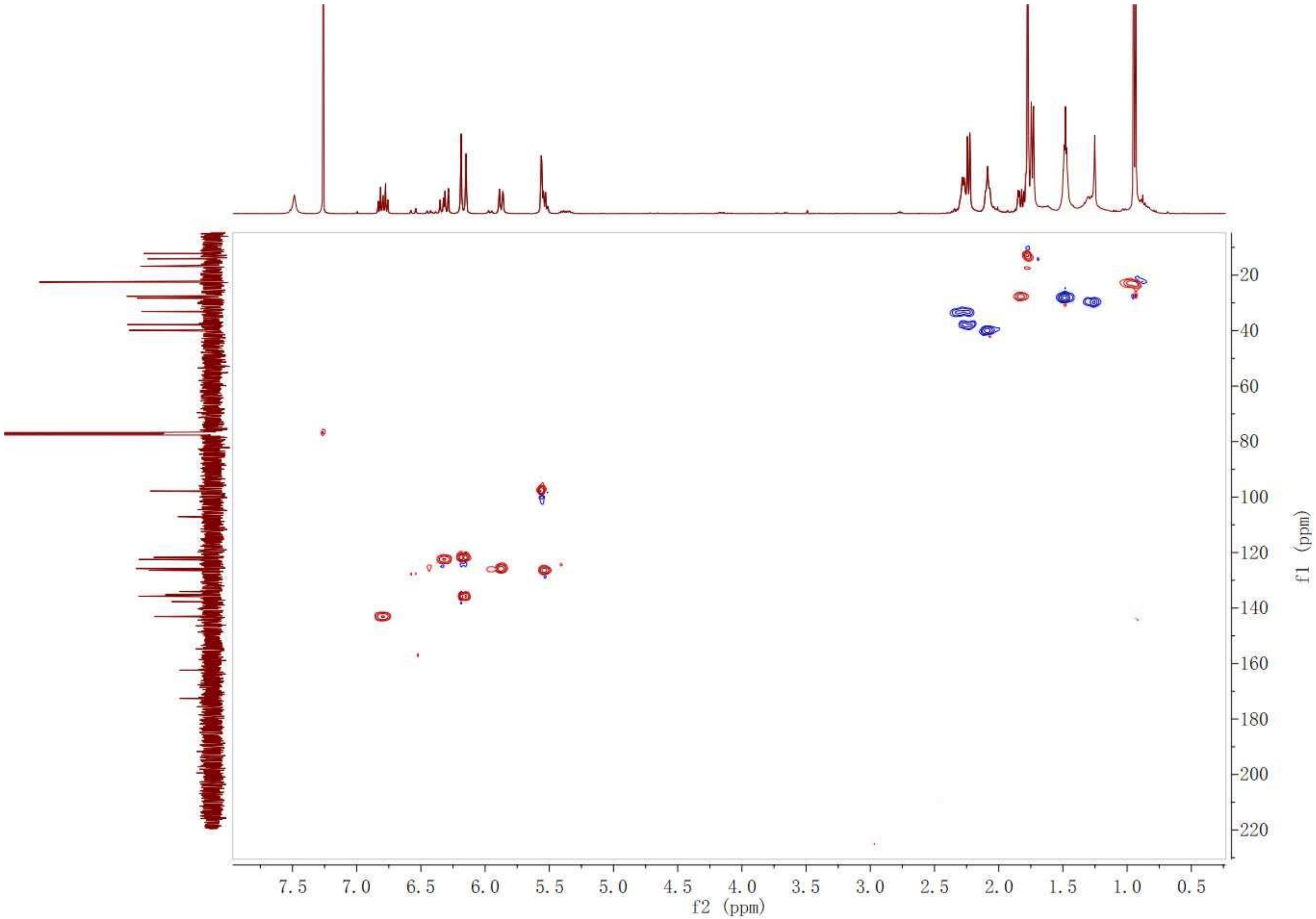

Supplementary Figure 47 HSQC spectrum of compound **3** in $CDCl_3$.

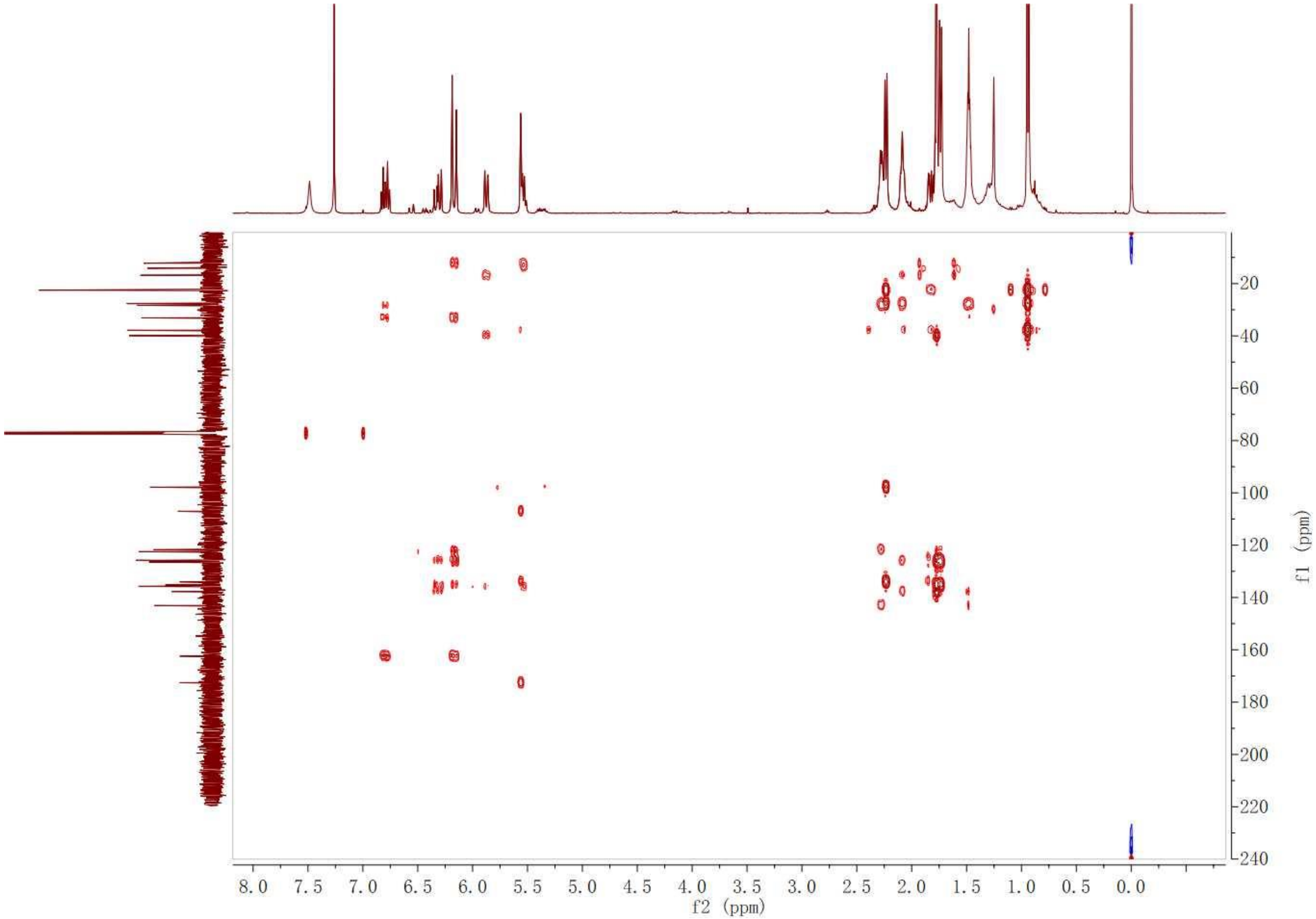

Supplementary Figure 48. HMBC spectrum of compound **3** in $CDCl_3$.

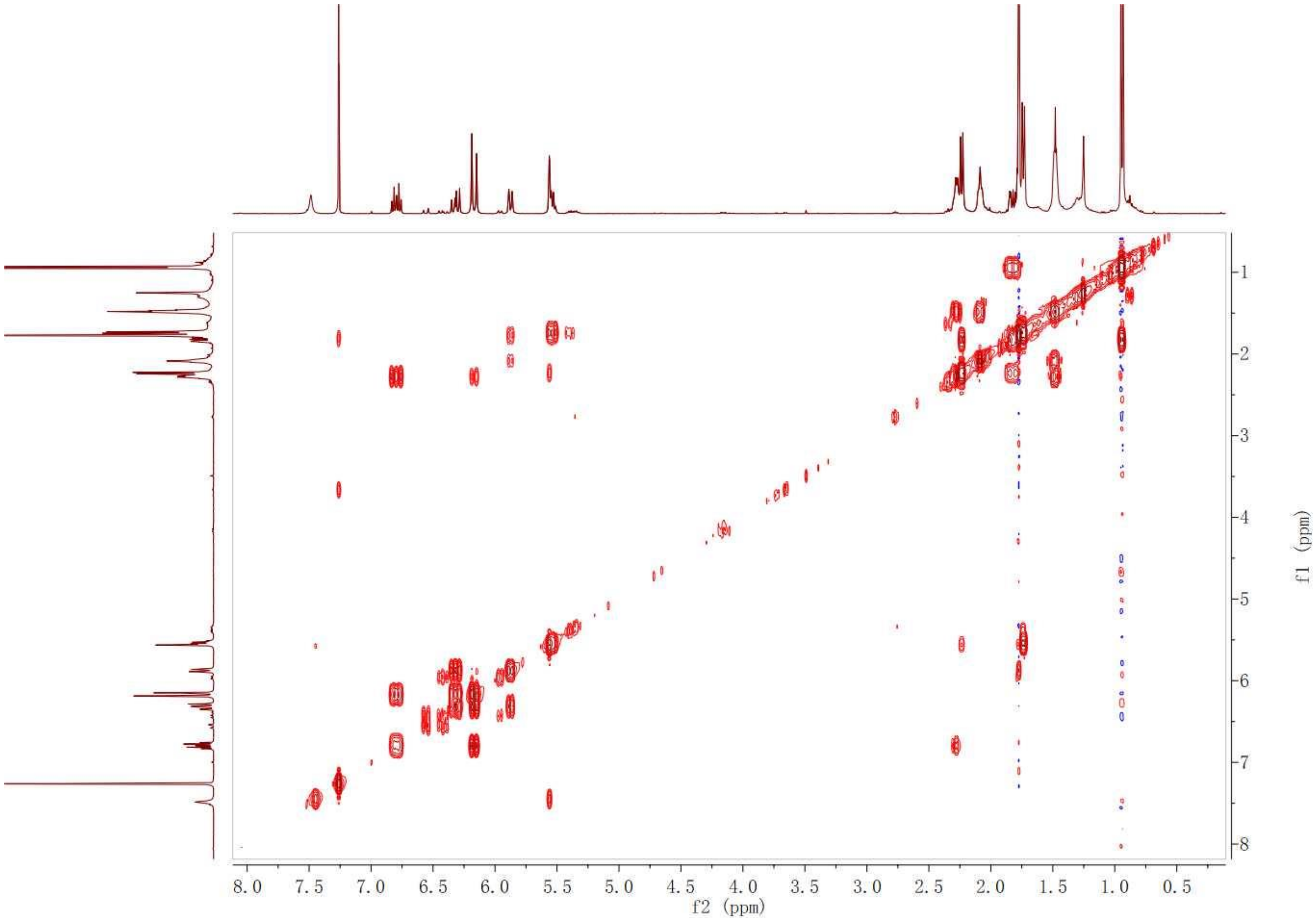

Supplementary Figure 49. ^1H-^1H COSY spectrum of compound **3** in $CDCl_3$.

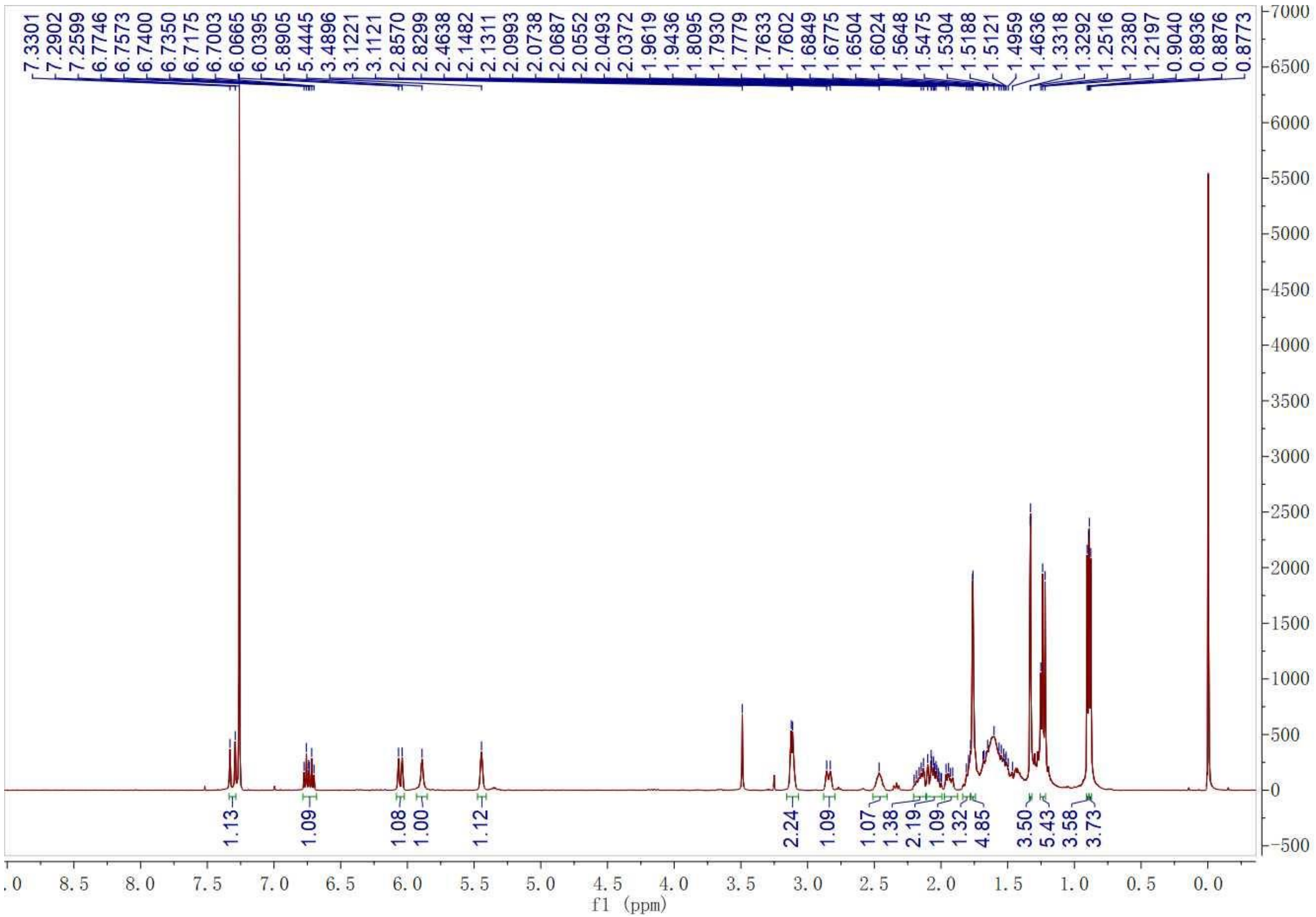

Supplementary Figure 50. ^{1}H NMR spectrum of compound **6** in $CDCl_3$ (400 MHz).

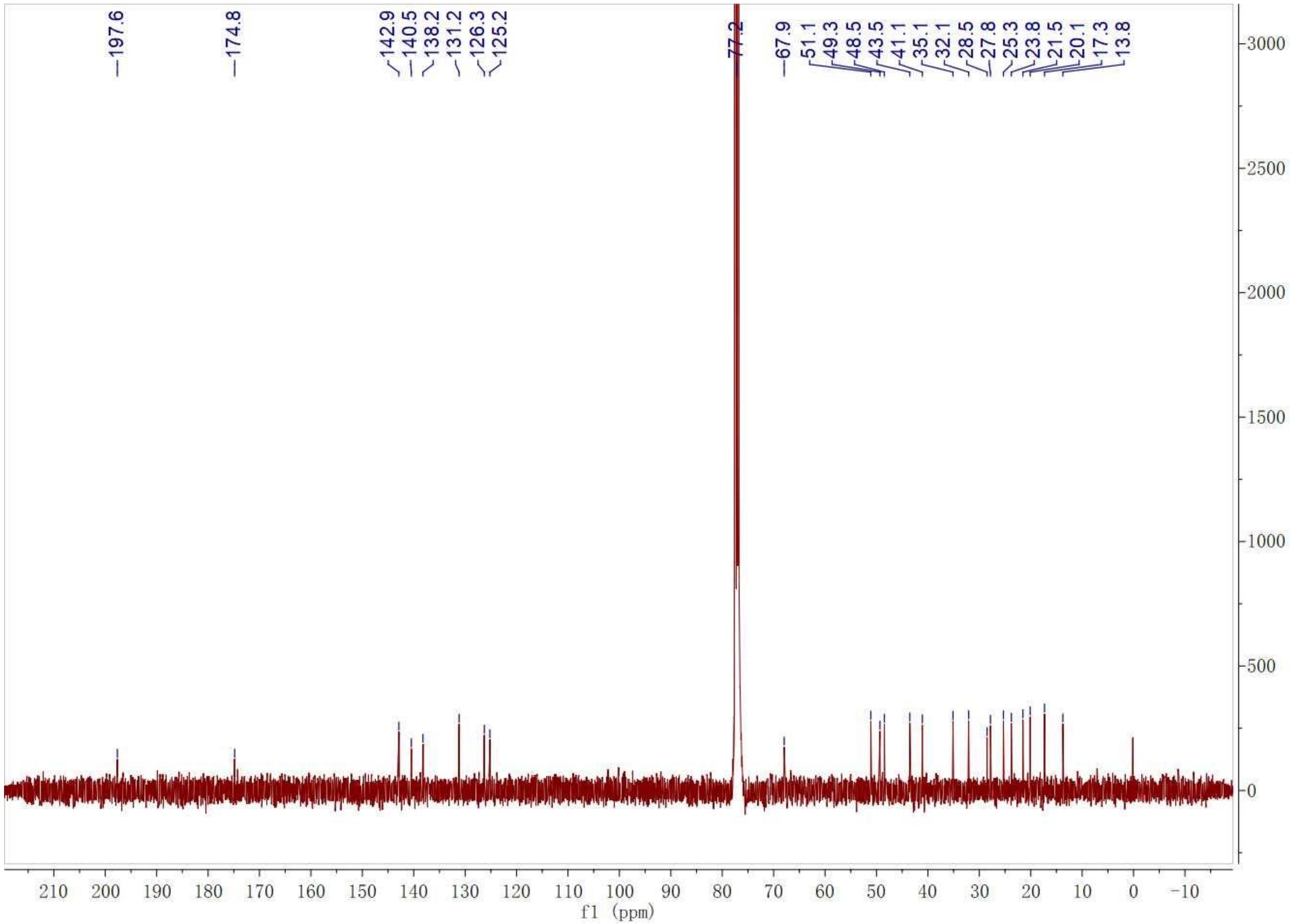

Supplementary Figure 51. ^{13}C NMR spectrum of compound **6** in $CDCl_3$ (100 MHz).

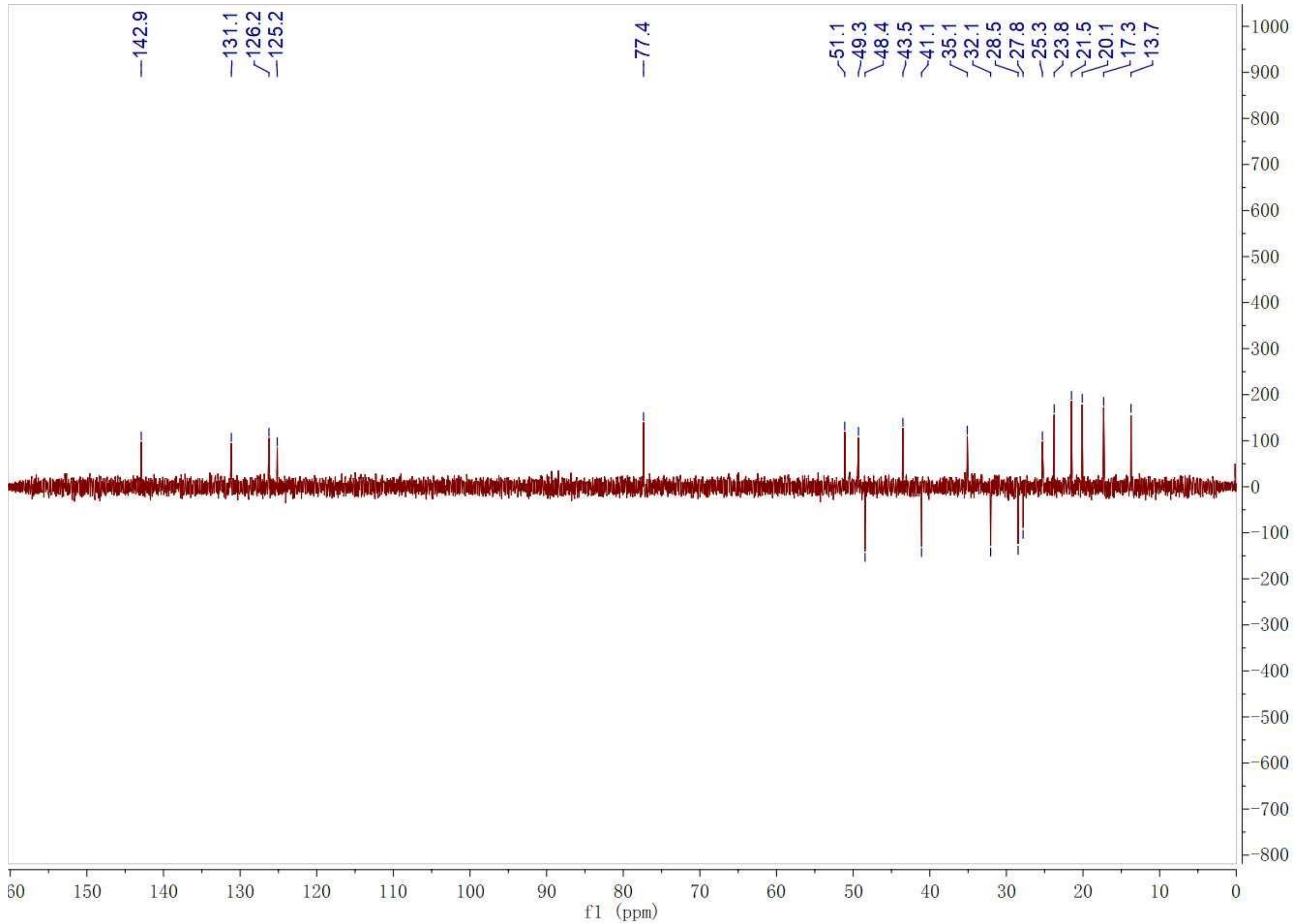

Supplementary Figure 52. DEPT-135° spectrum of compound **6** in $CDCl_3$.

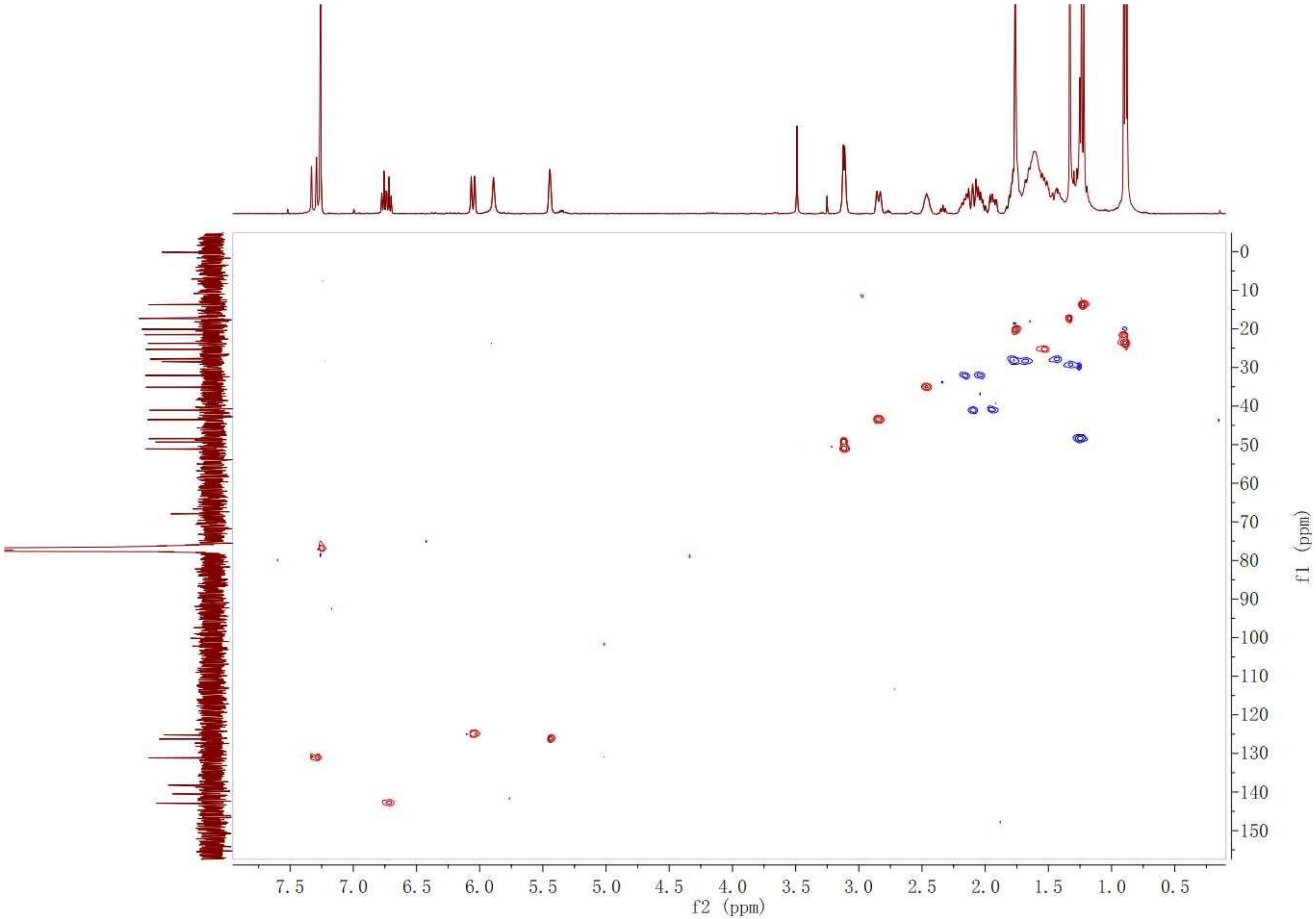

Supplementary Figure 53. HSQC spectrum of compound **6** in $CDCl_3$.

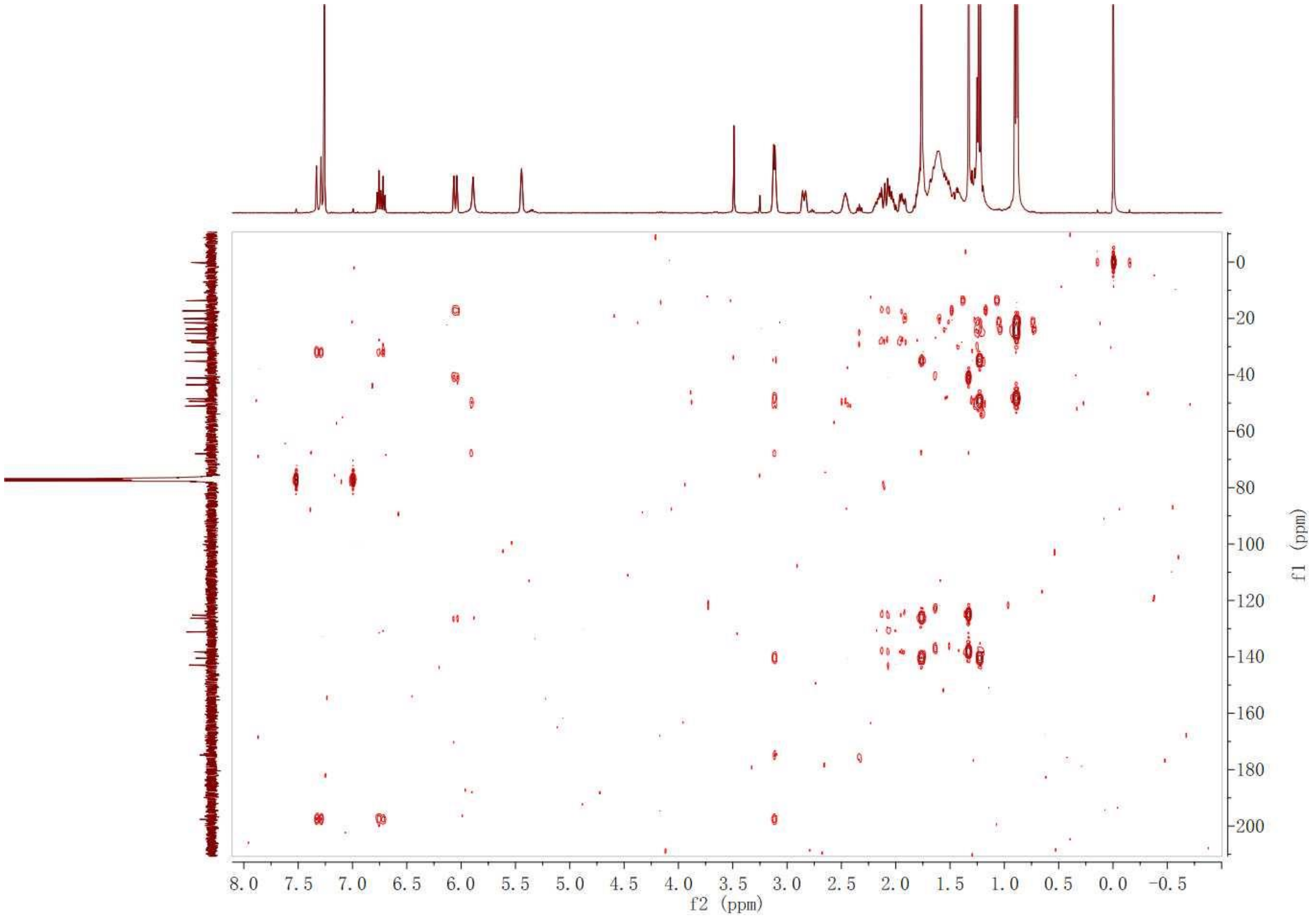

Supplementary Figure 54. HMBC spectrum of compound **6** in $CDCl_3$.

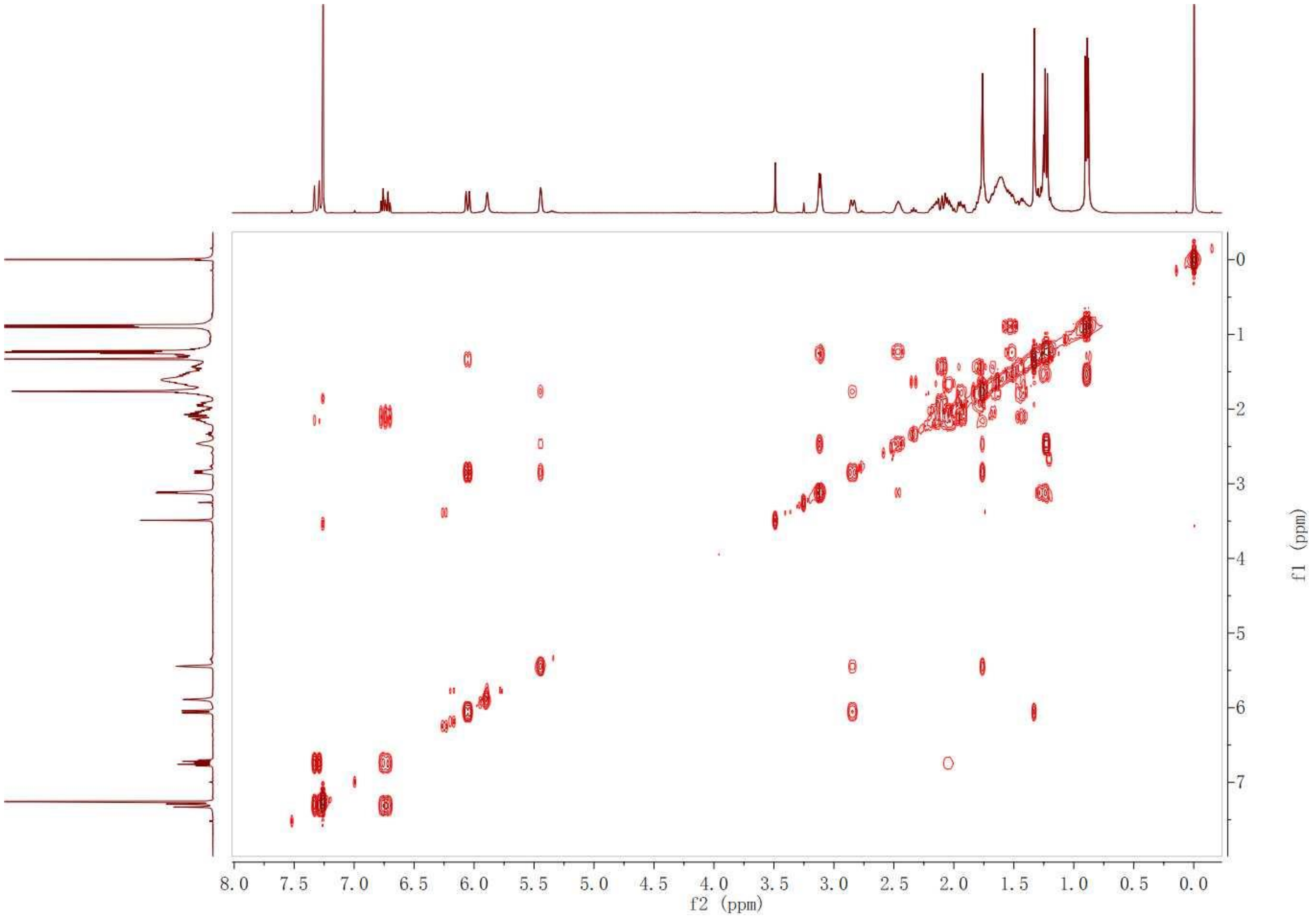

Supplementary Figure 55. ^{1}H-^{1}H COSY spectrum of compound **6** in $CDCl_3$.

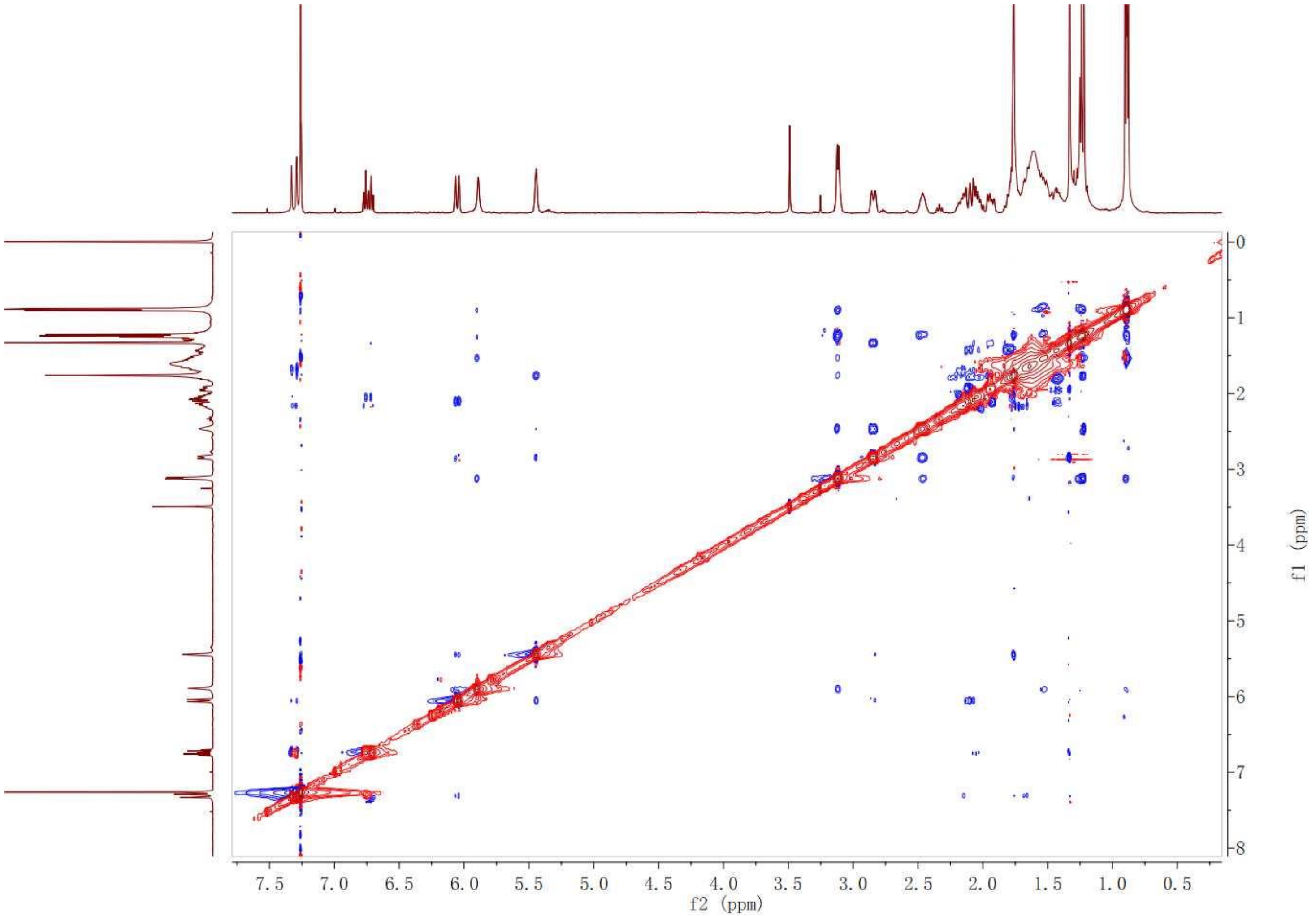

Supplementary Figure 56. ^{1}H-^{1}H NOESY spectrum of compound **6** in $CDCl_3$.

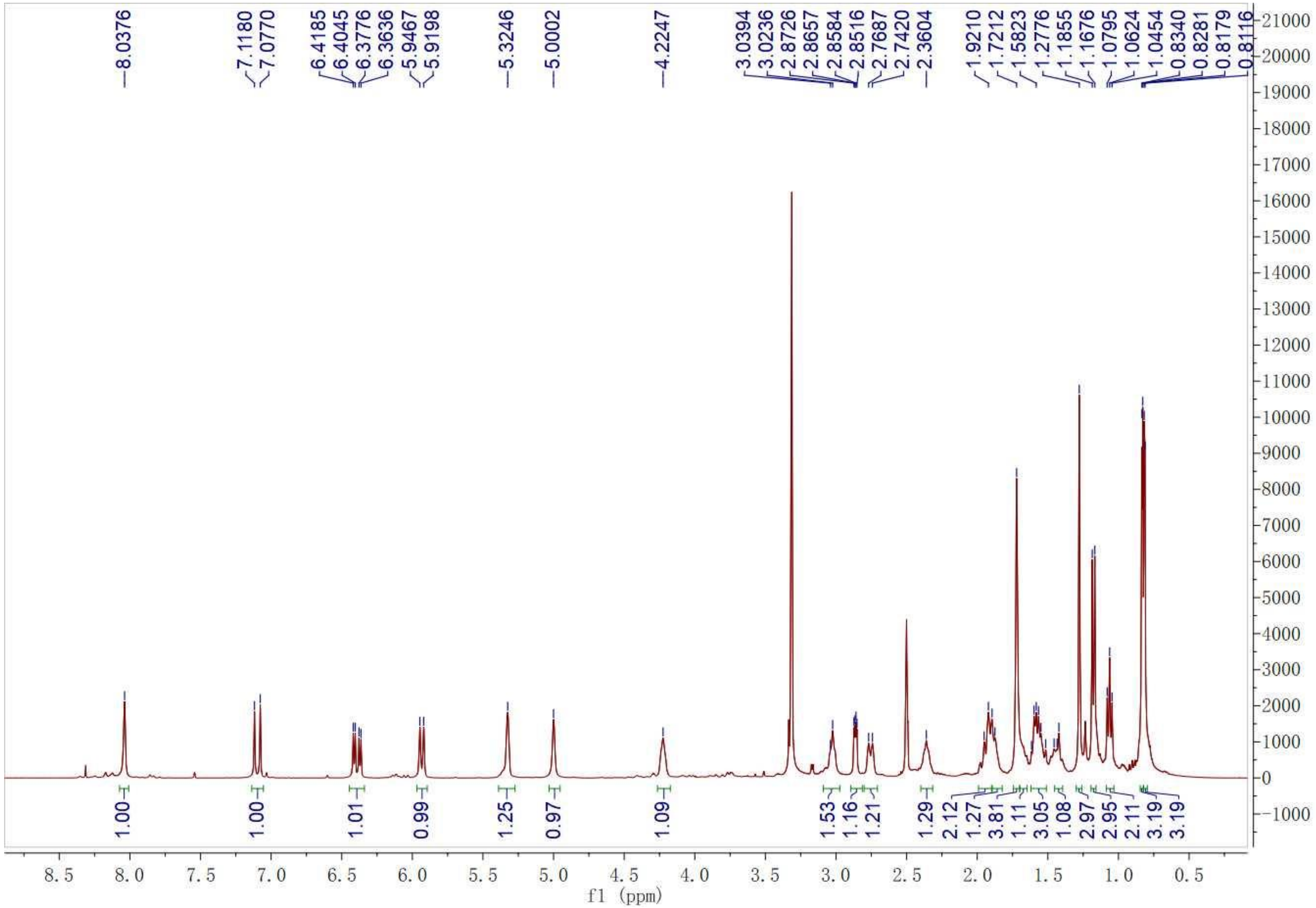

Supplementary Figure 57. ^{1}H NMR spectrum of compound **7** in DMSO-d_6 (400 MHz).

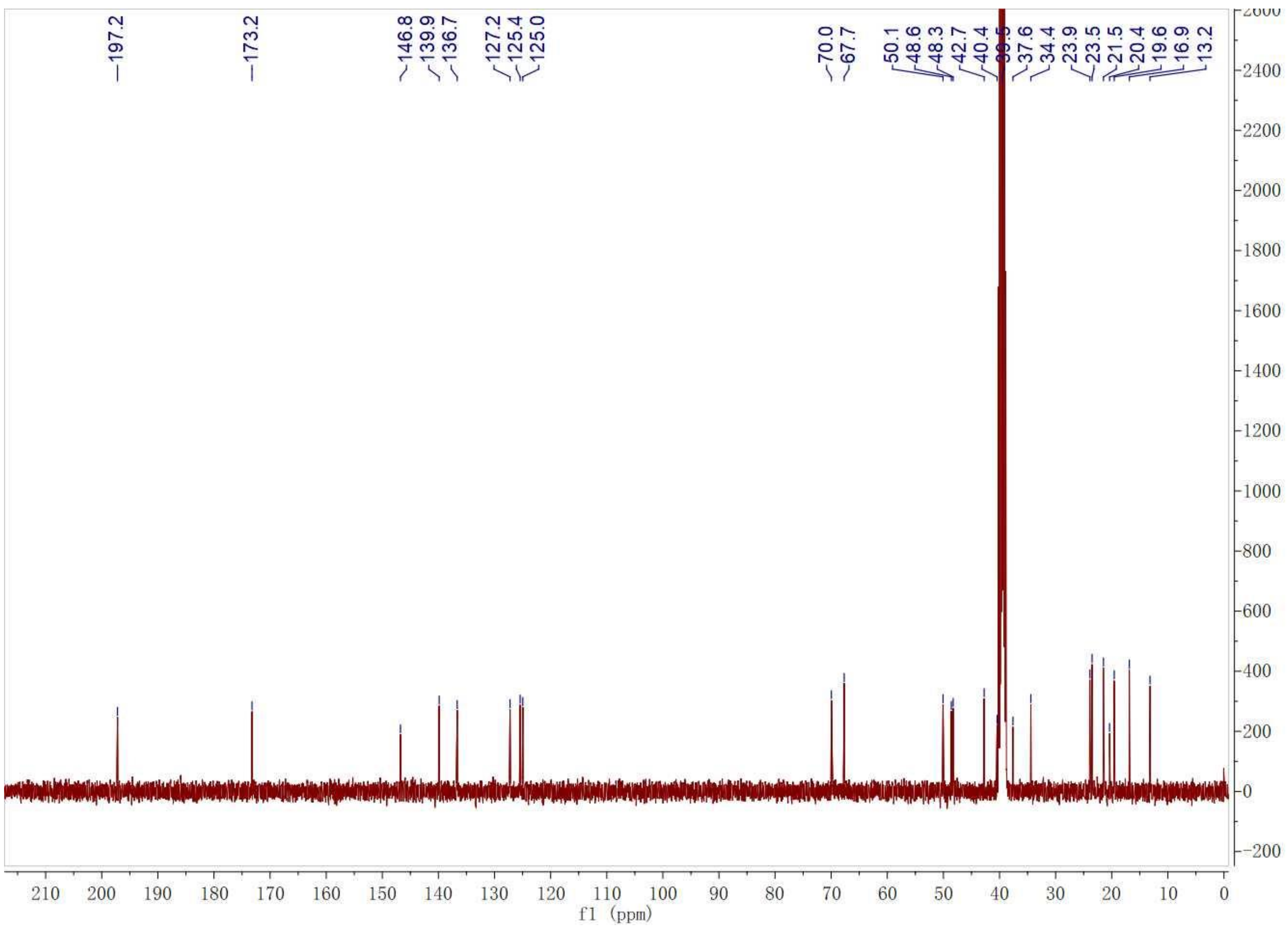

Supplementary Figure 58. ^{13}C NMR spectrum of compound **7** in DMSO-d_6 (100 MHz).

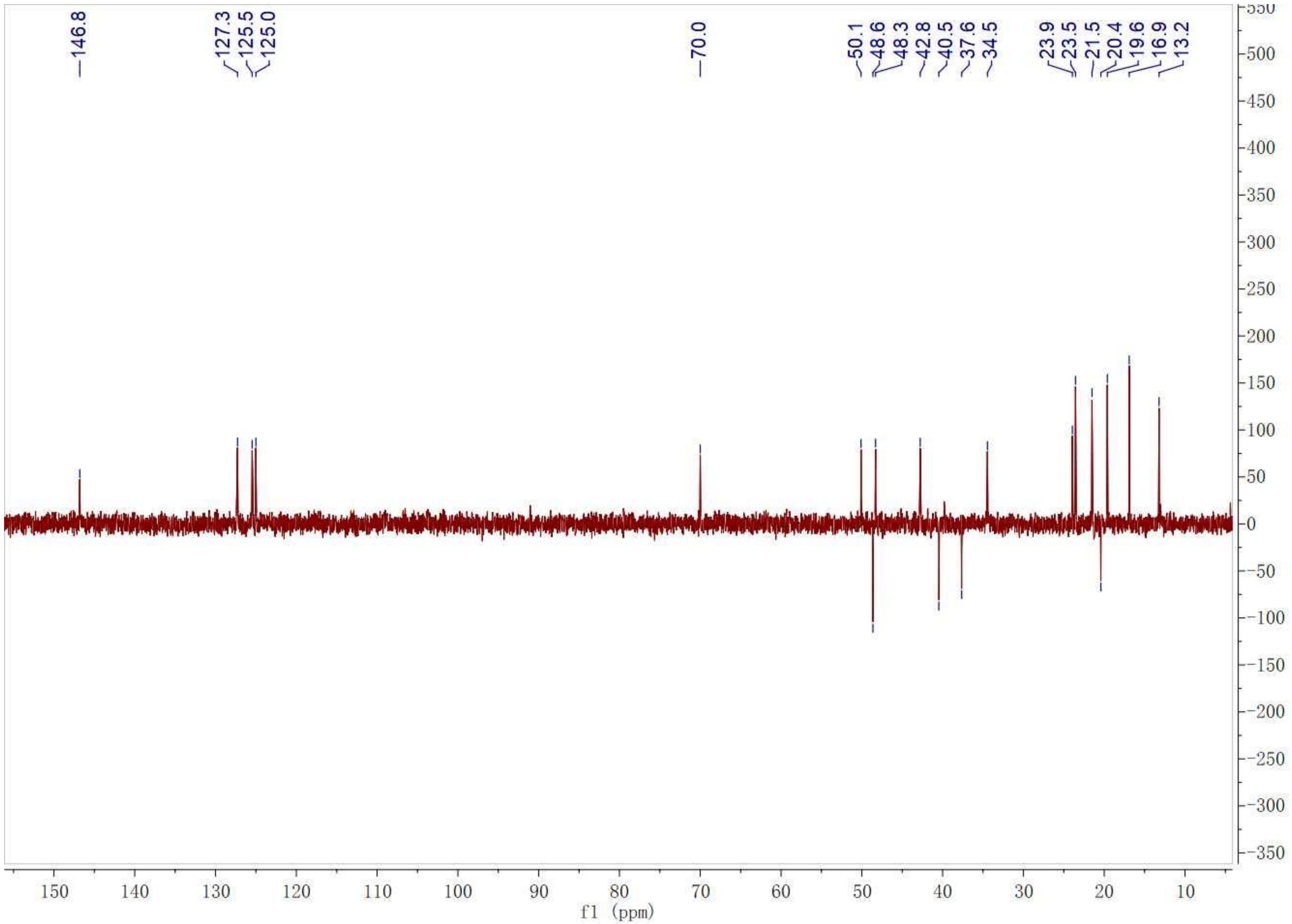

Supplementary Figure 59. DEPT-135° spectrum of compound **7** in DMSO-d_6.

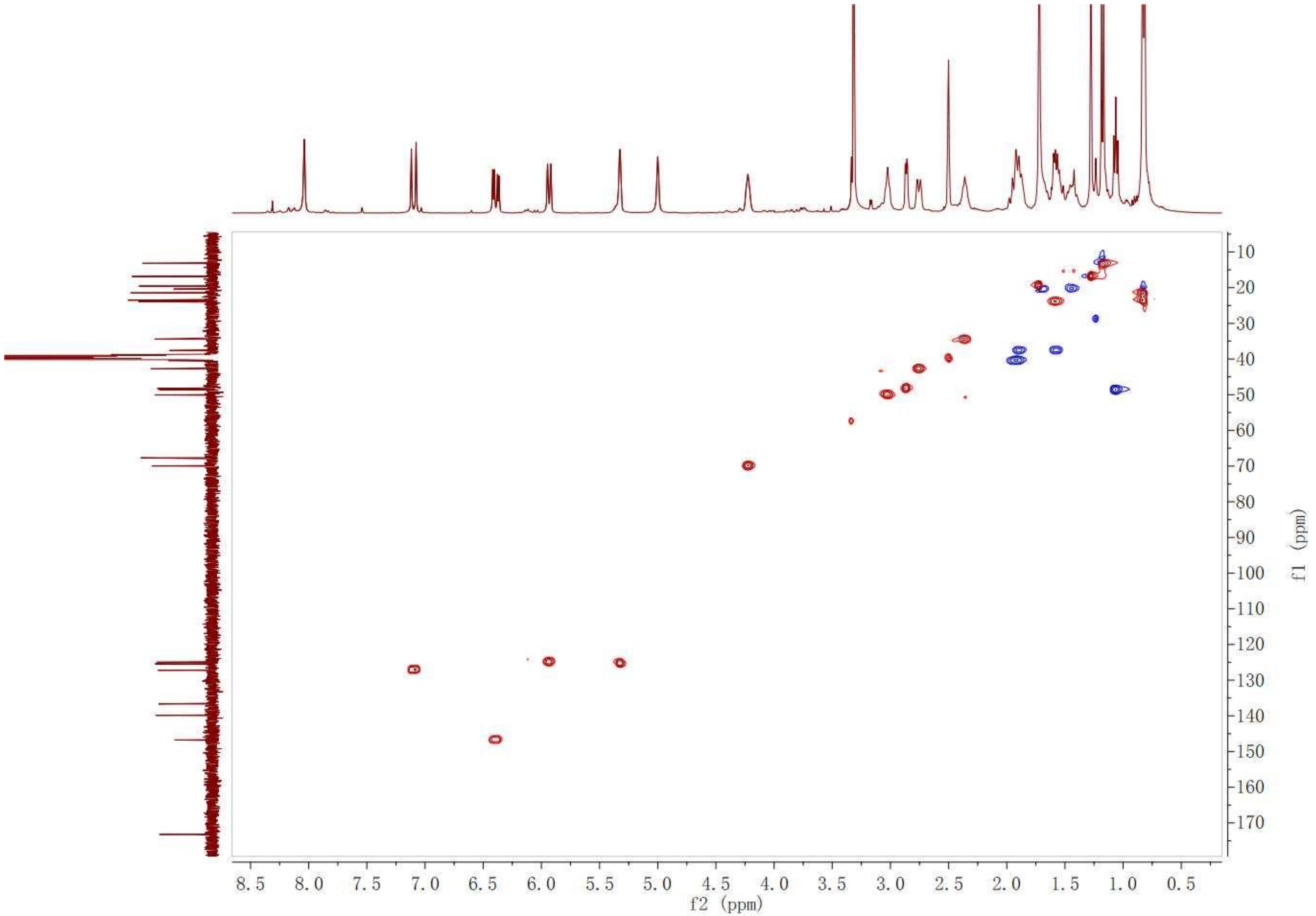

Supplementary Figure 60. HSQC spectrum of compound **7** in DMSO-d_6.

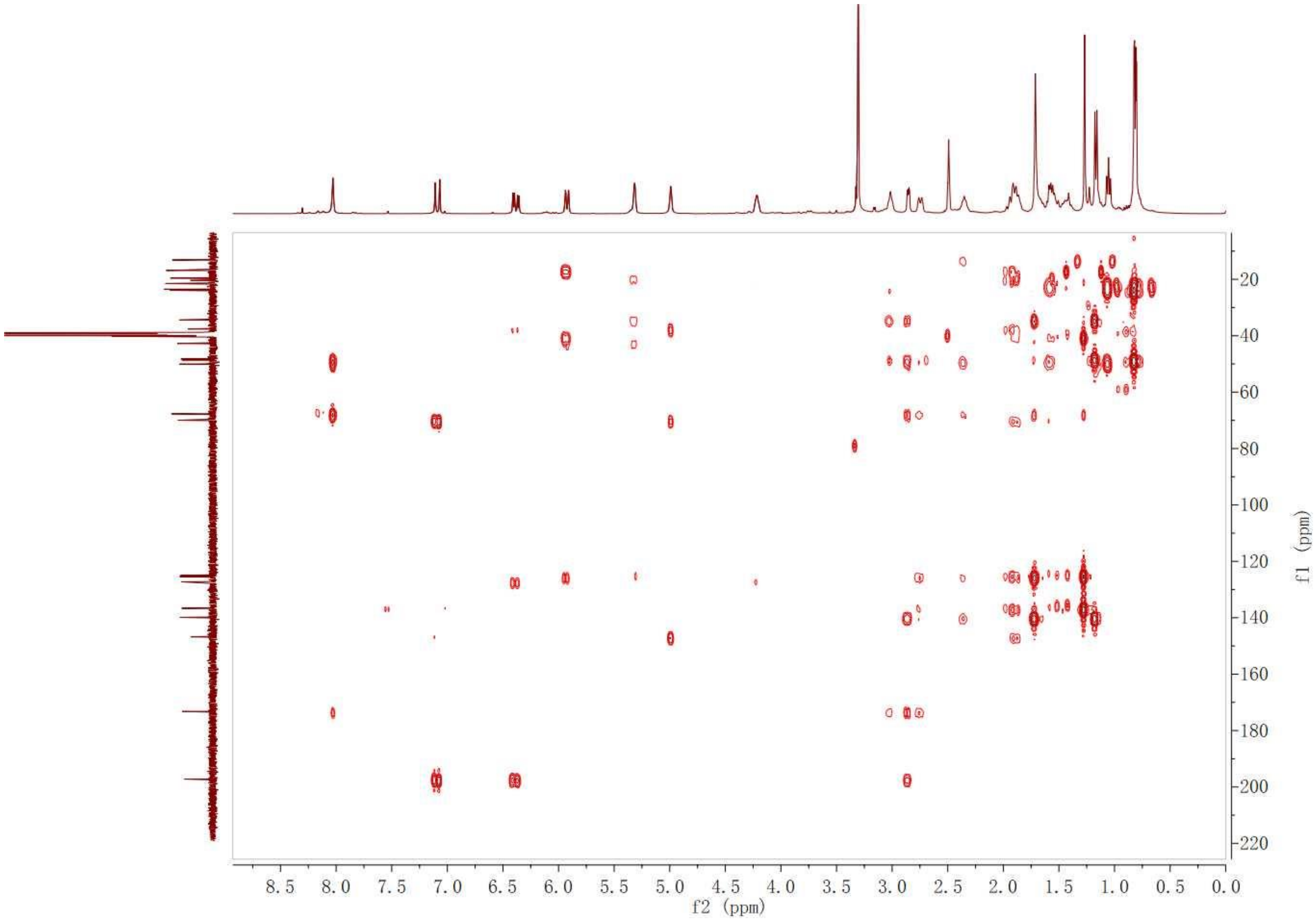

Supplementary Figure 61. HMBC spectrum of compound **7** in DMSO-d_6.

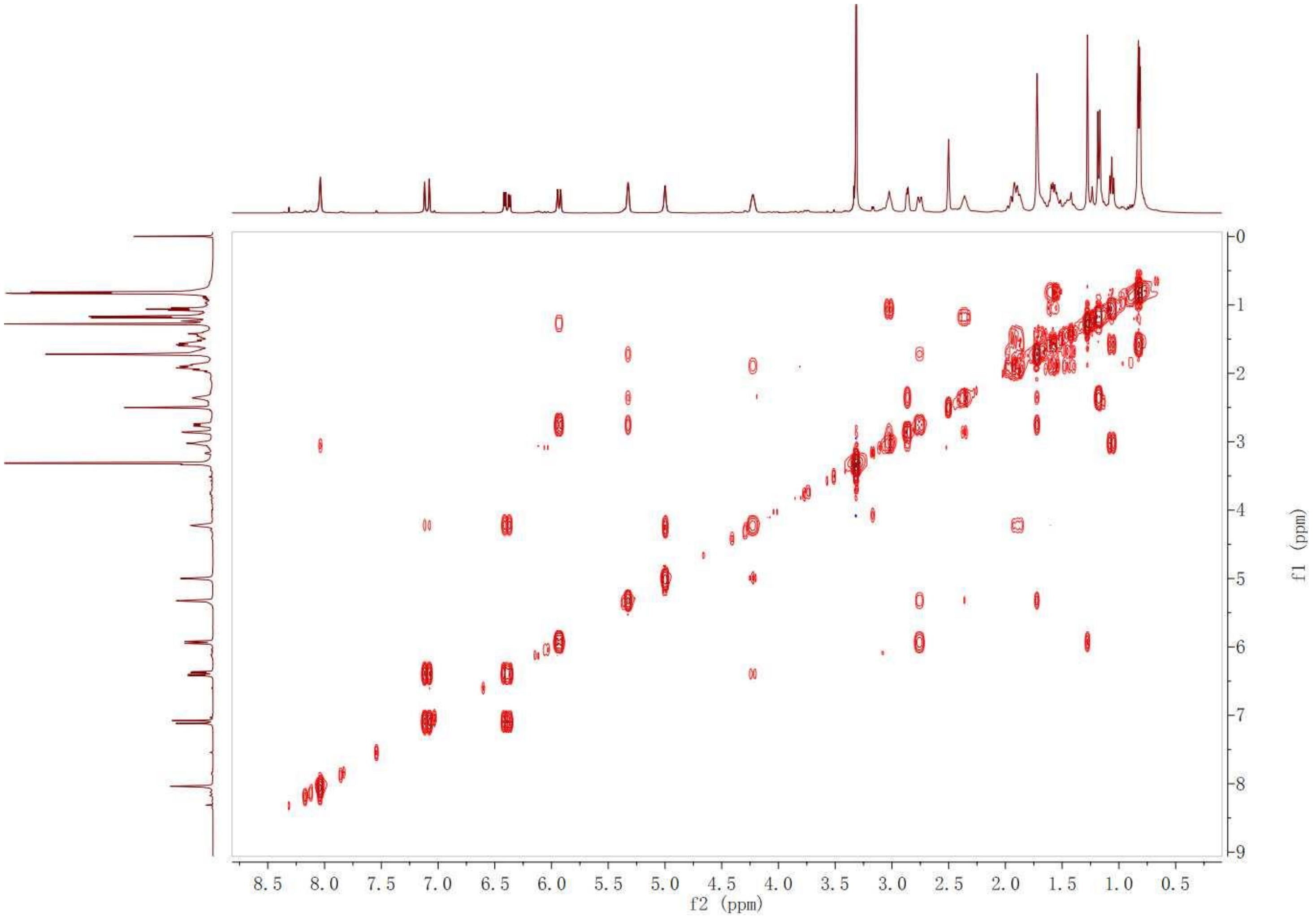

Supplementary Figure 62. ^{1}H-^{1}H COSY spectrum of compound **7** in DMSO-d_6.

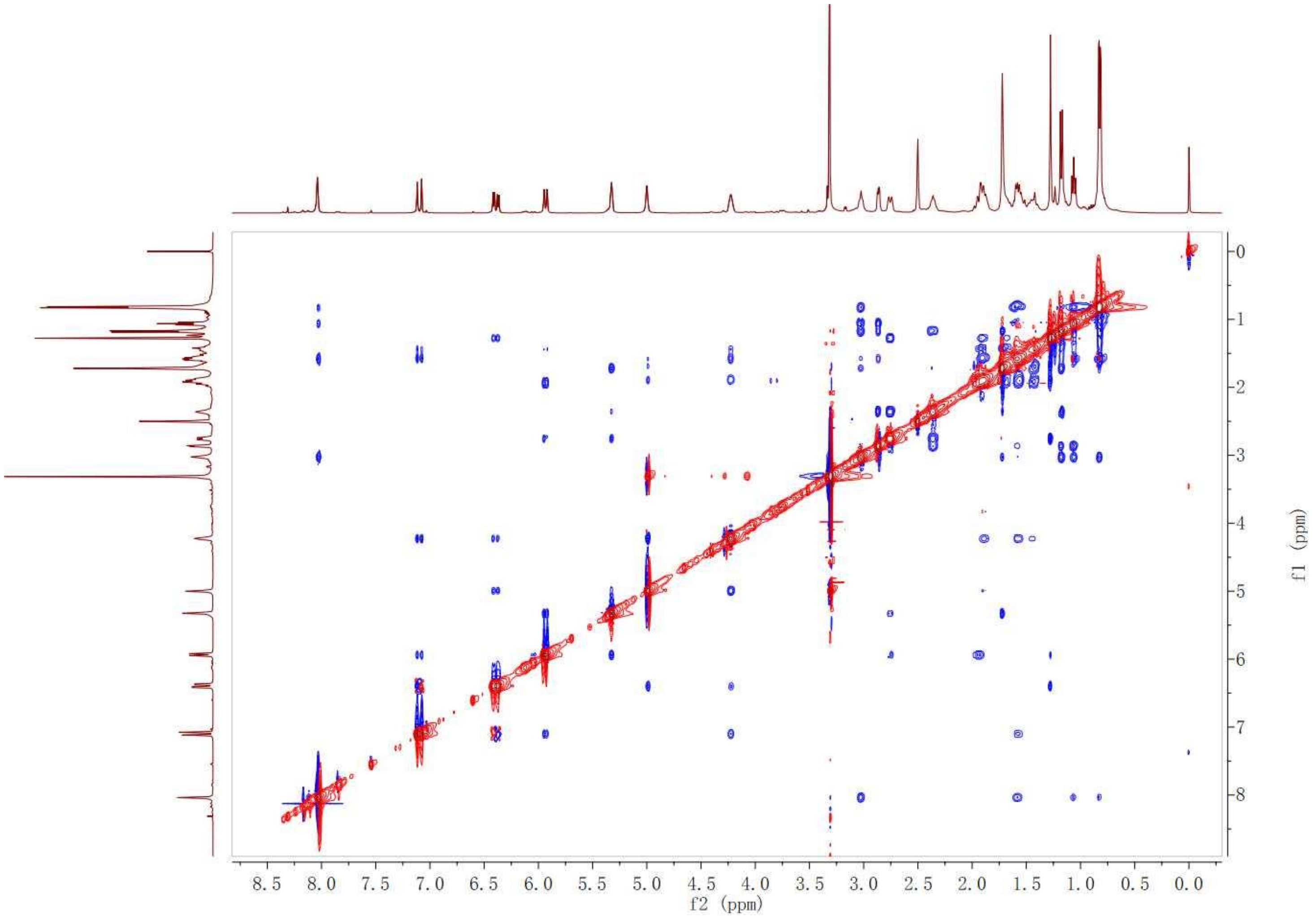

Supplementary Figure 63. ^{1}H-^{1}H NOESY spectrum of compound **7** in DMSO-d_6.

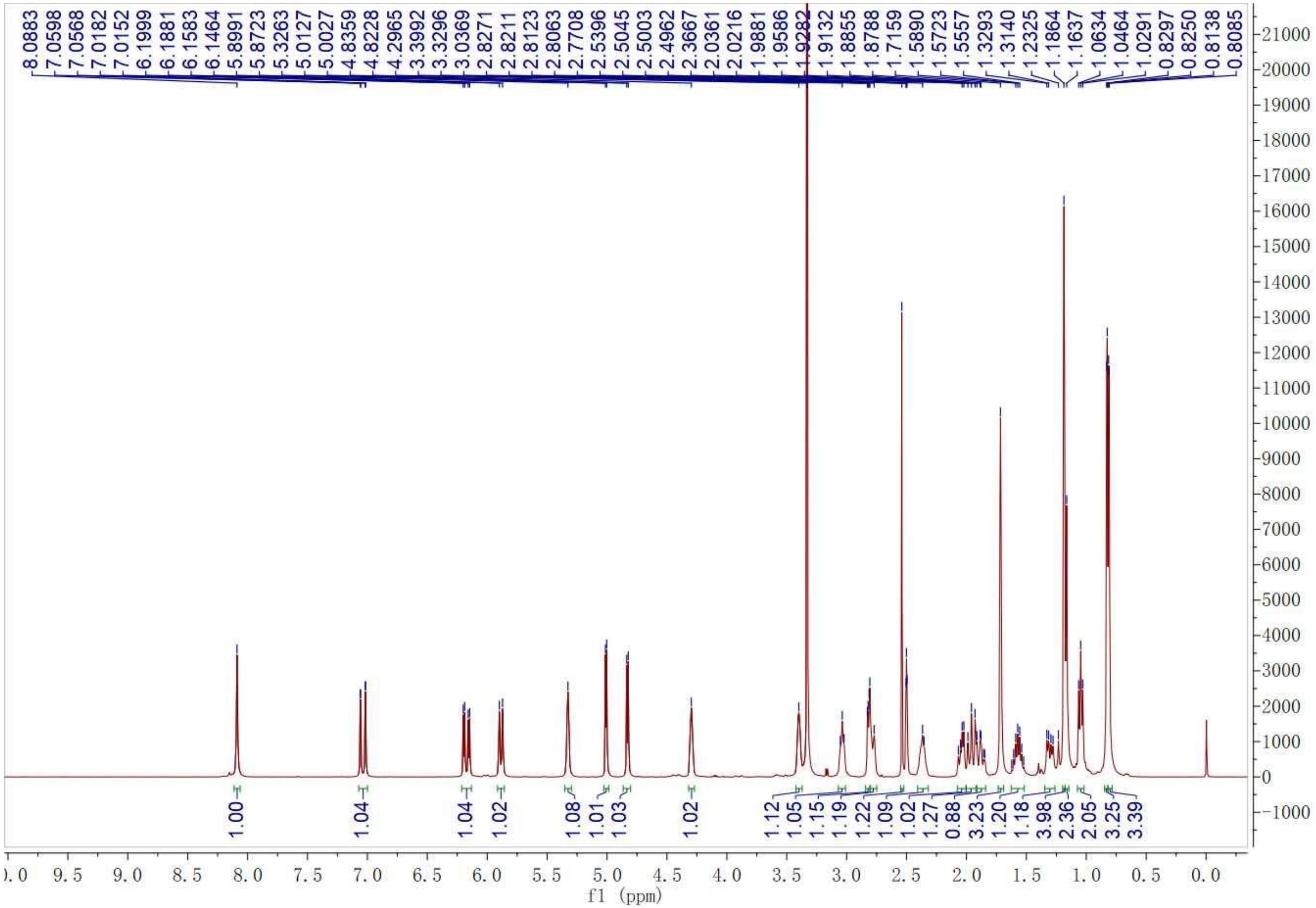

Supplementary Figure 64. ^{1}H NMR spectrum of compound **8** in DMSO-d_6 (400 MHz).

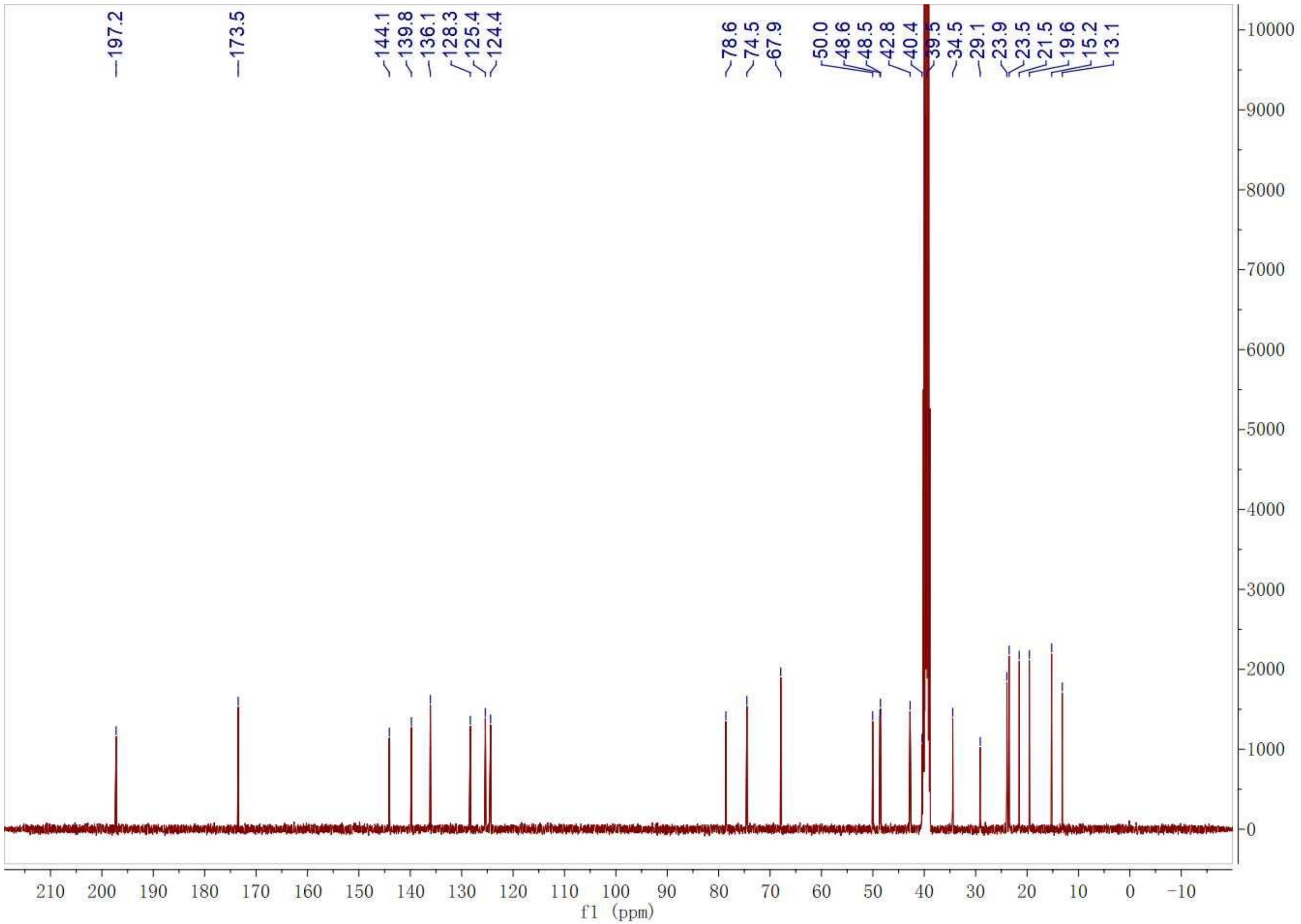

Supplementary Figure 65. ^{13}C NMR spectrum of compound **8** in DMSO-d_6 (100 MHz).

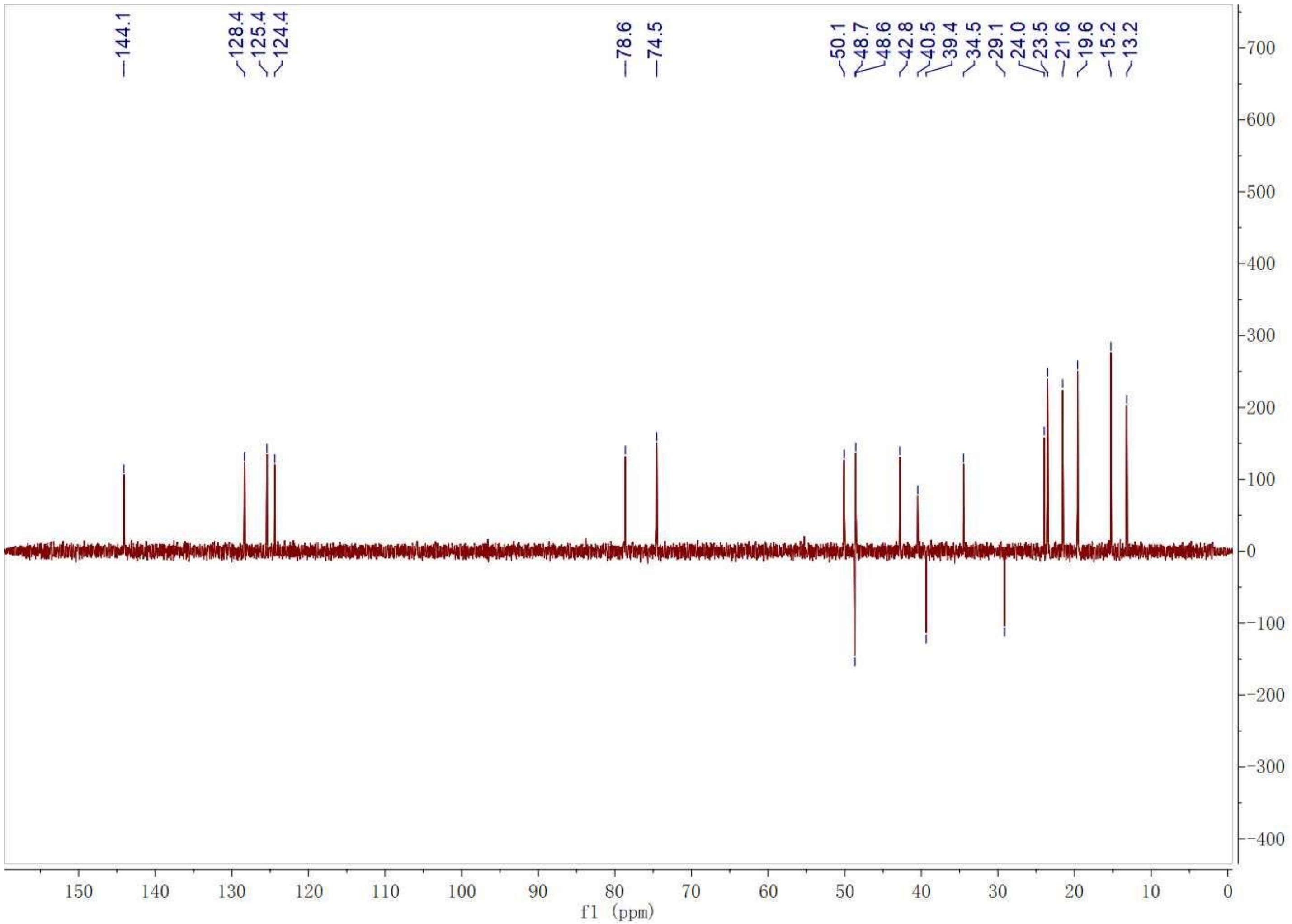

Supplementary Figure 66. DEPT-135° spectrum of compound **8** in DMSO-d_6.

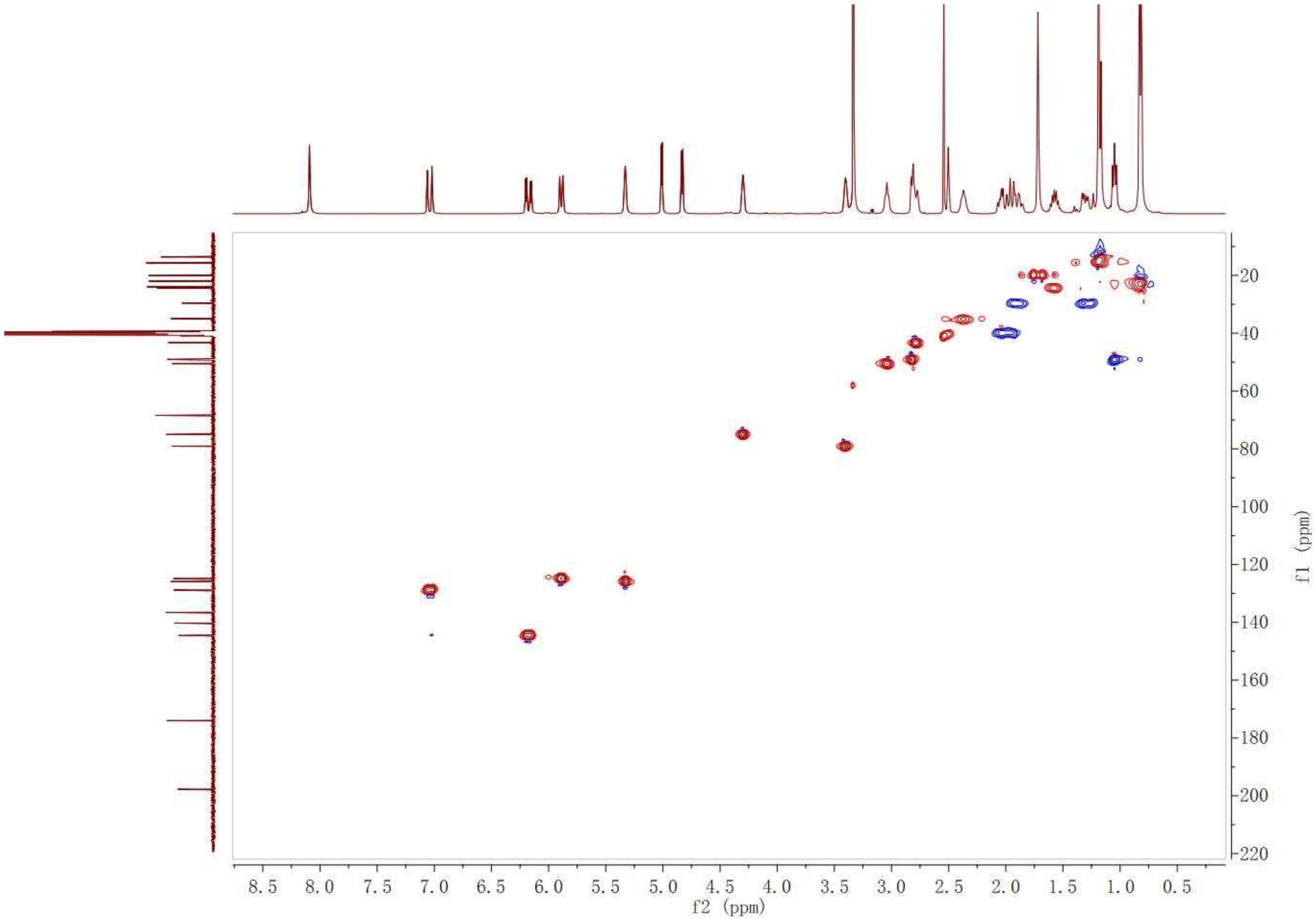

Supplementary Figure 67. HSQC spectrum of compound **8** in DMSO-d_6.

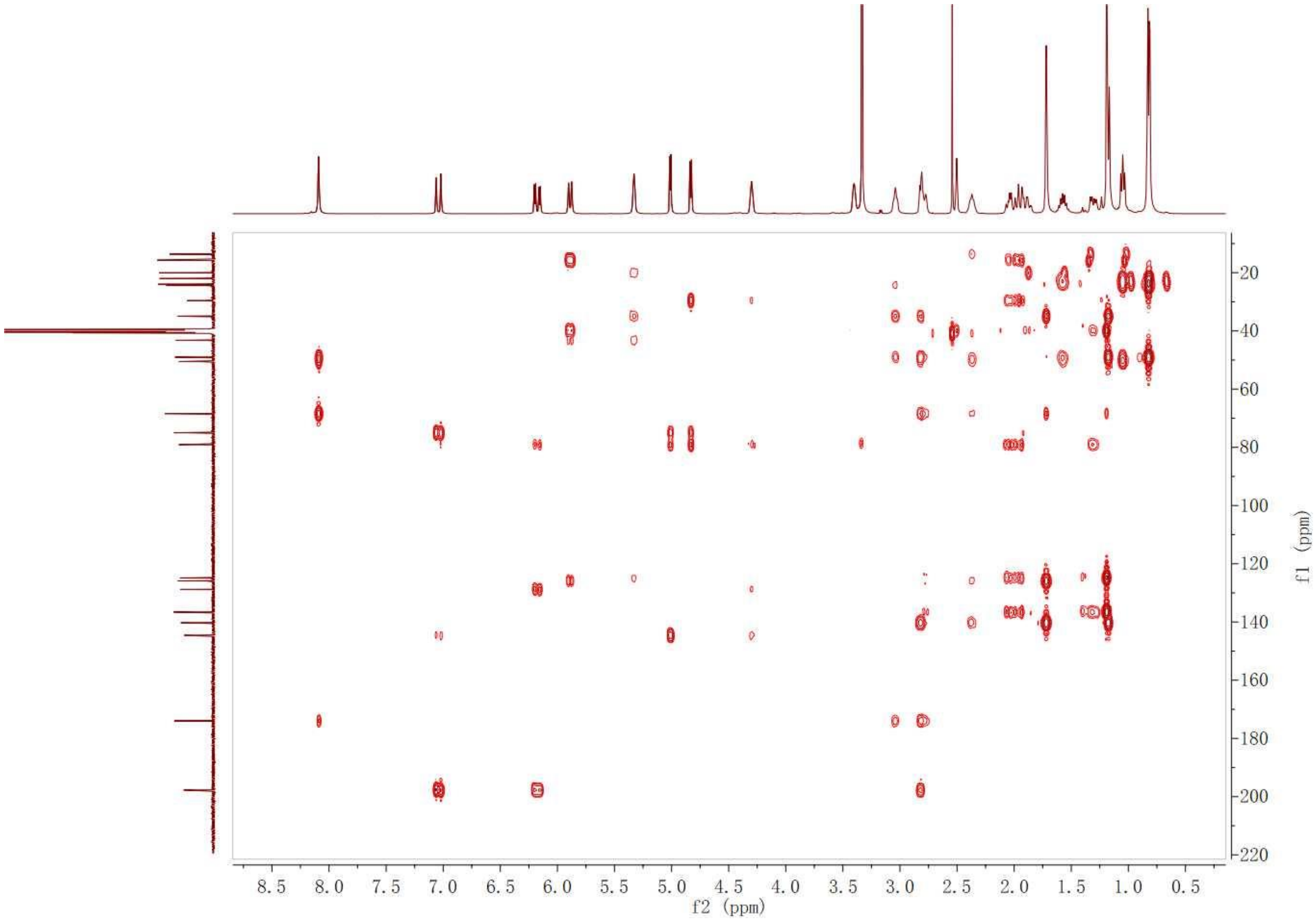

Supplementary Figure 68. HMBC spectrum of compound **8** in DMSO-d_6.

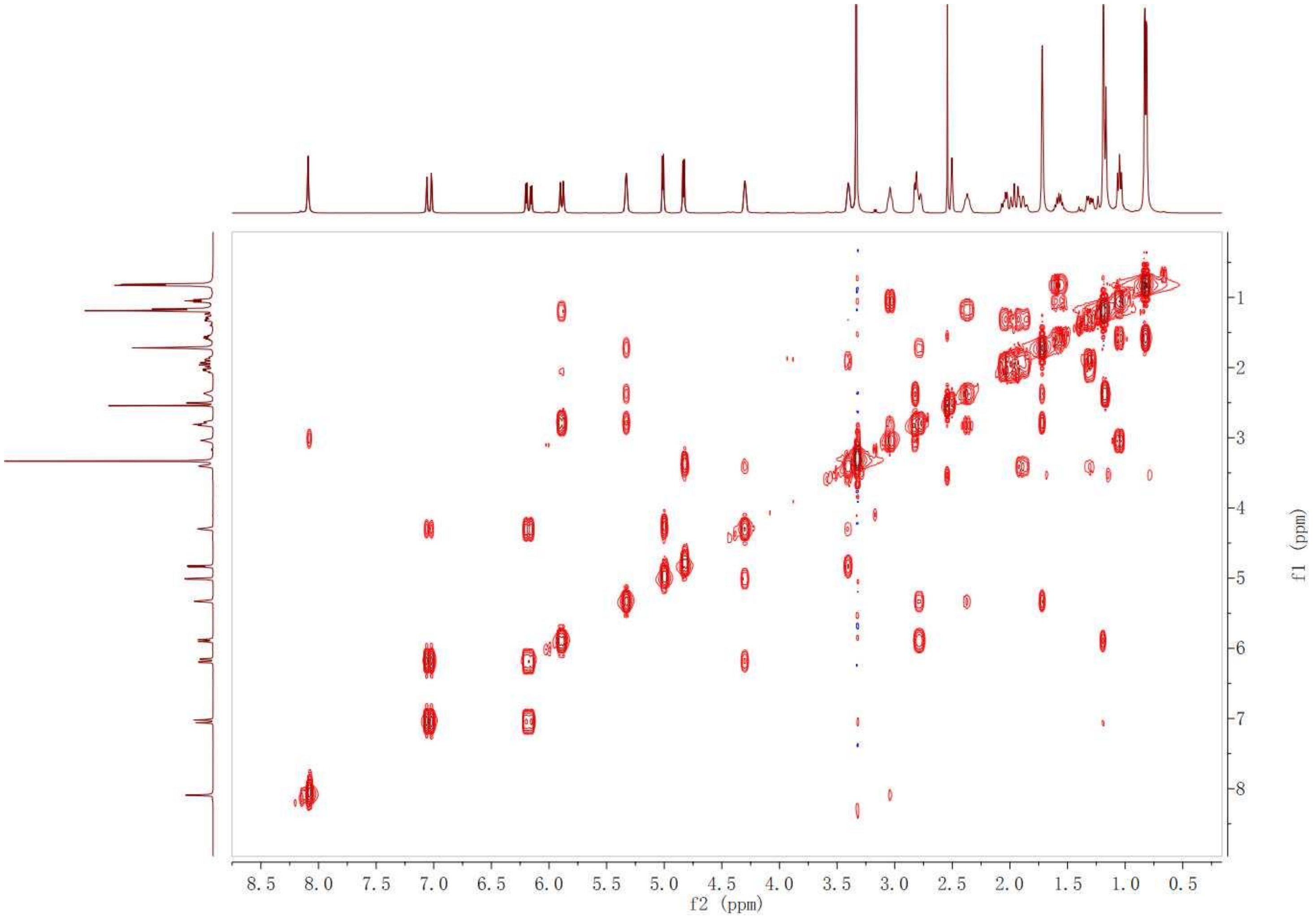

Supplementary Figure 69. ^{1}H-^{1}H COSY spectrum of compound **8** in DMSO-d_6.

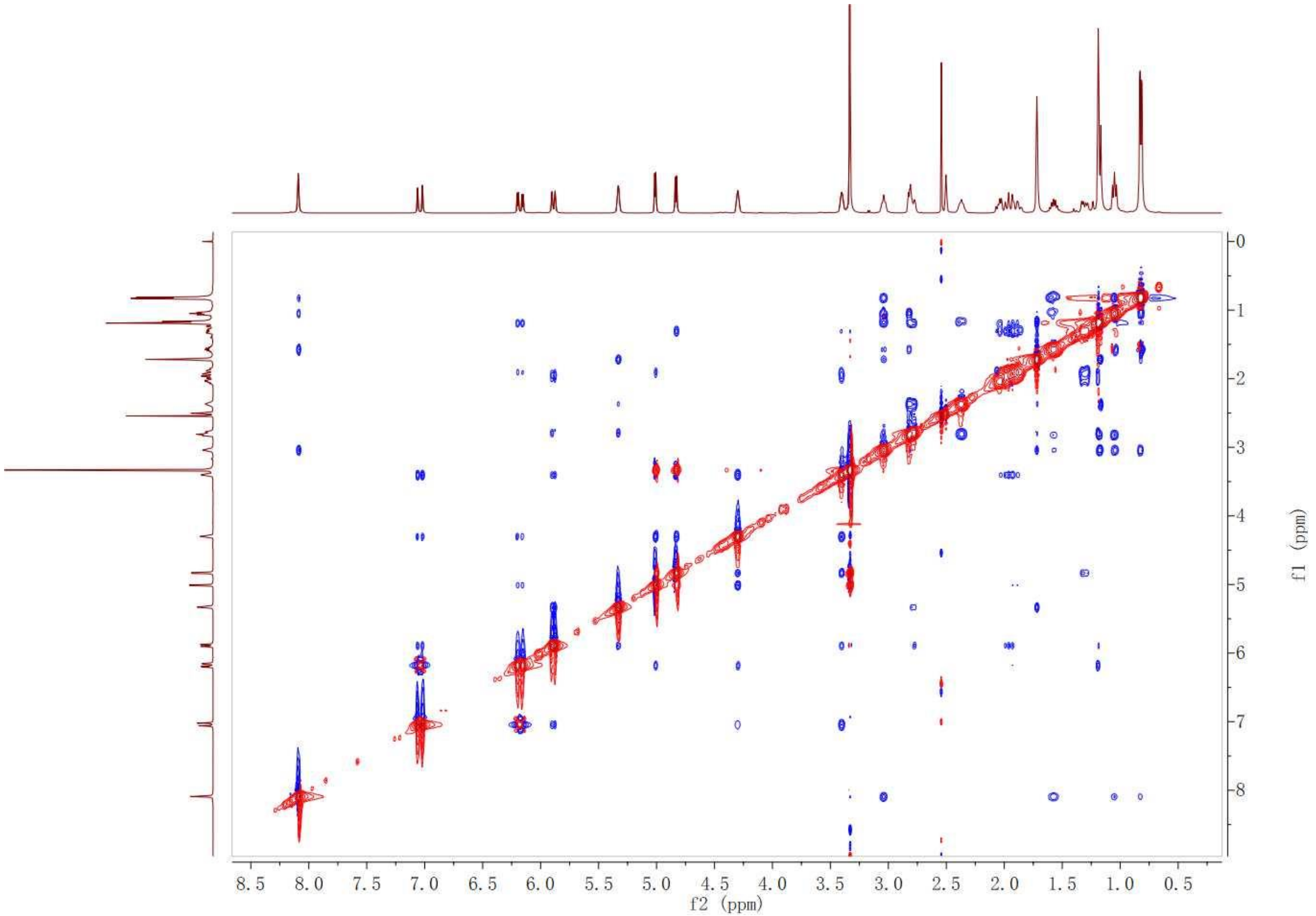

Supplementary Figure 70. ^{1}H-^{1}H NOESY spectrum of compound **8** in DMSO-d_6.

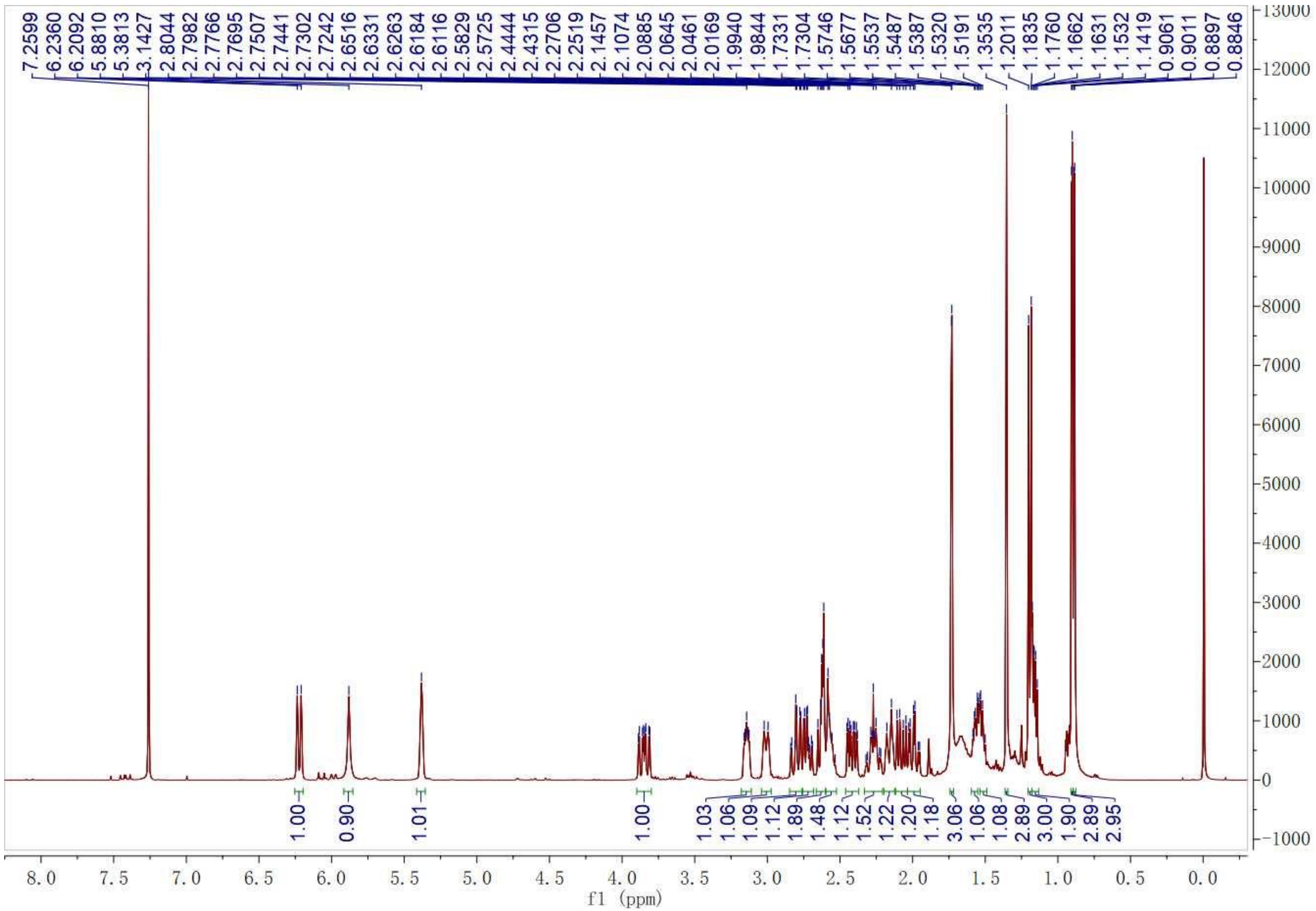

Supplementary Figure 71. ^{1}H NMR spectrum of compound **11** in $CDCl_3$ (400 MHz).

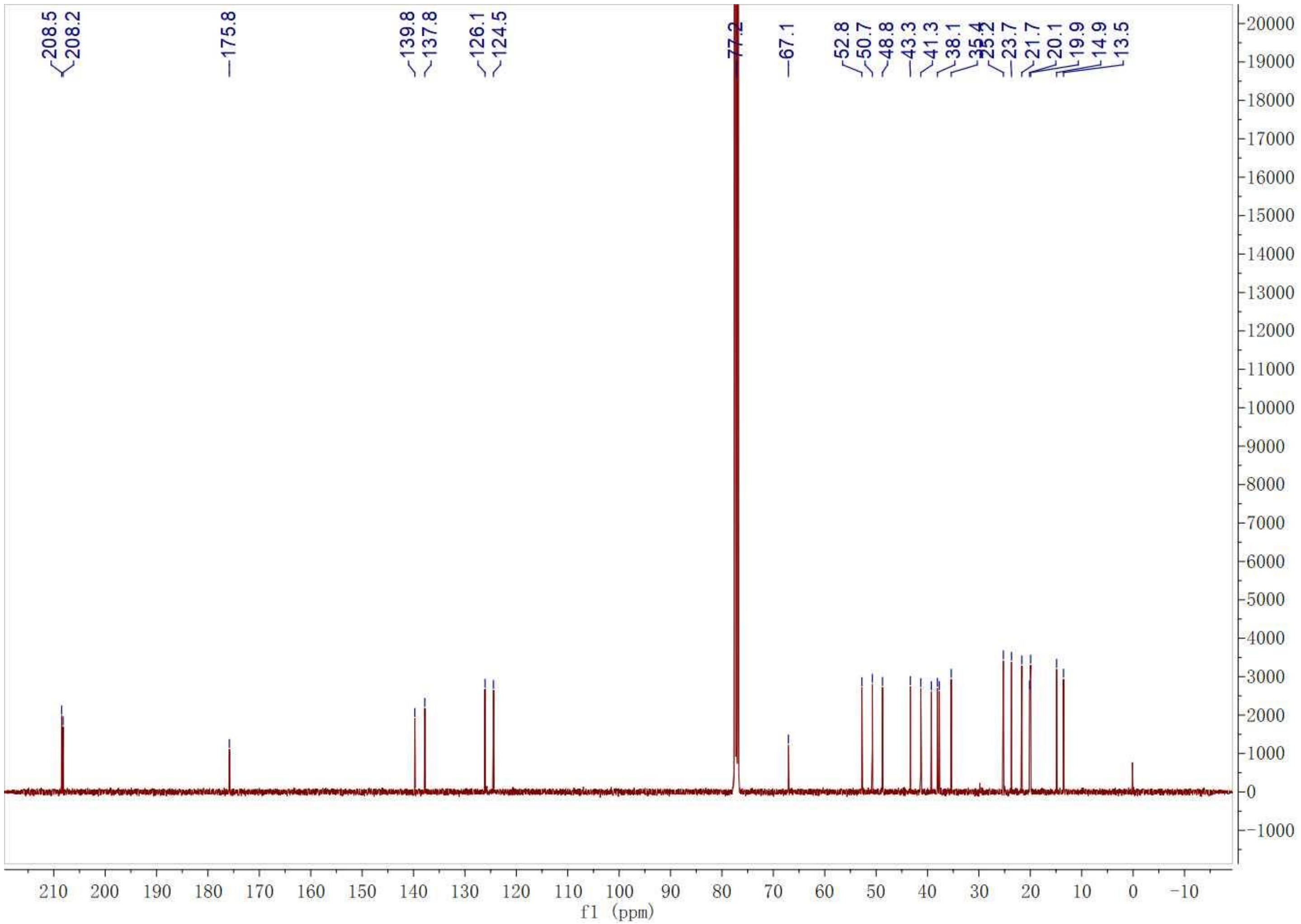

Supplementary Figure 72. ^{13}C NMR spectrum of compound **11** in $CDCl_3$ (100 MHz).

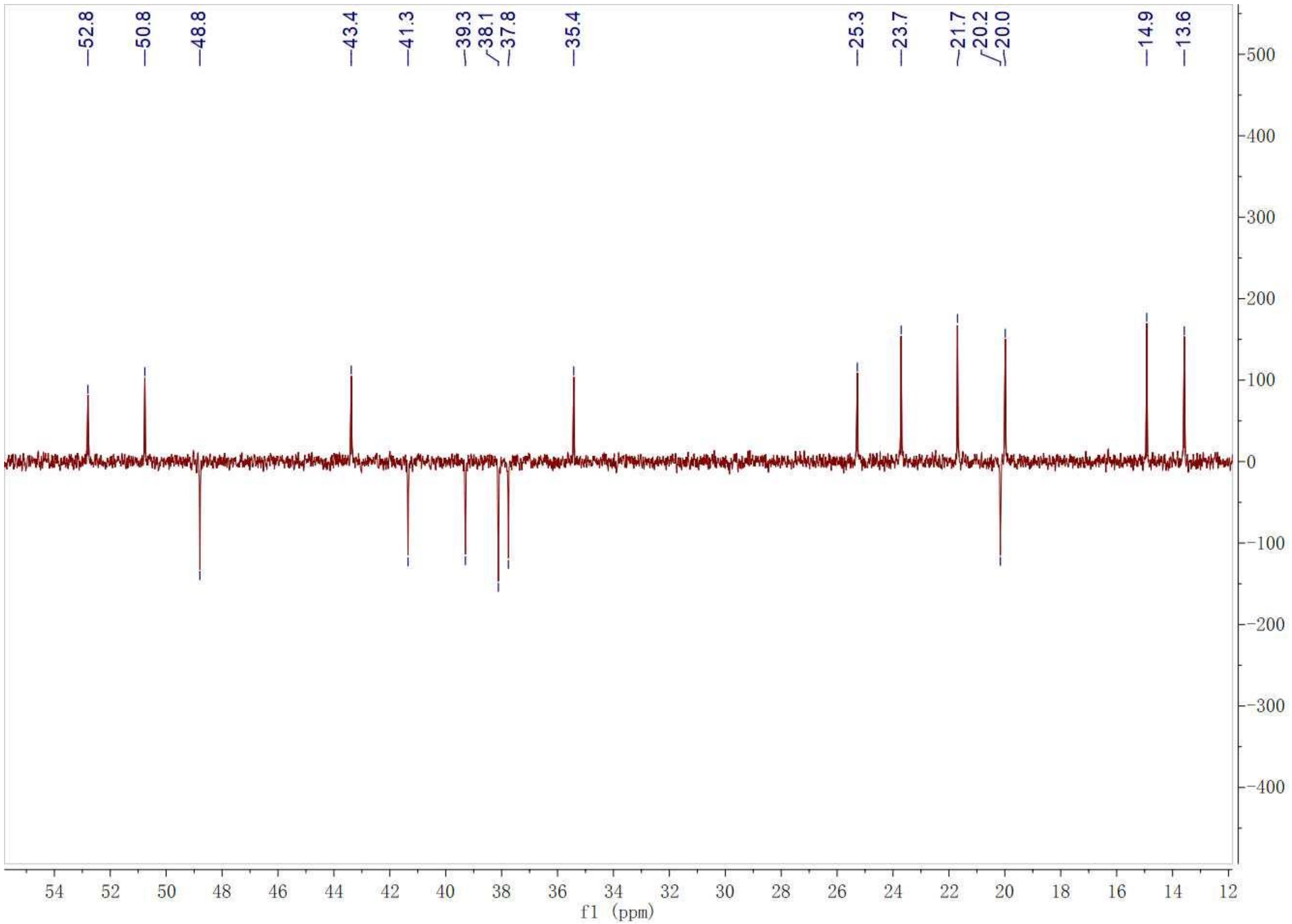

Supplementary Figure 73. DEPT-135° spectrum of compound **11** in $CDCl_3$.

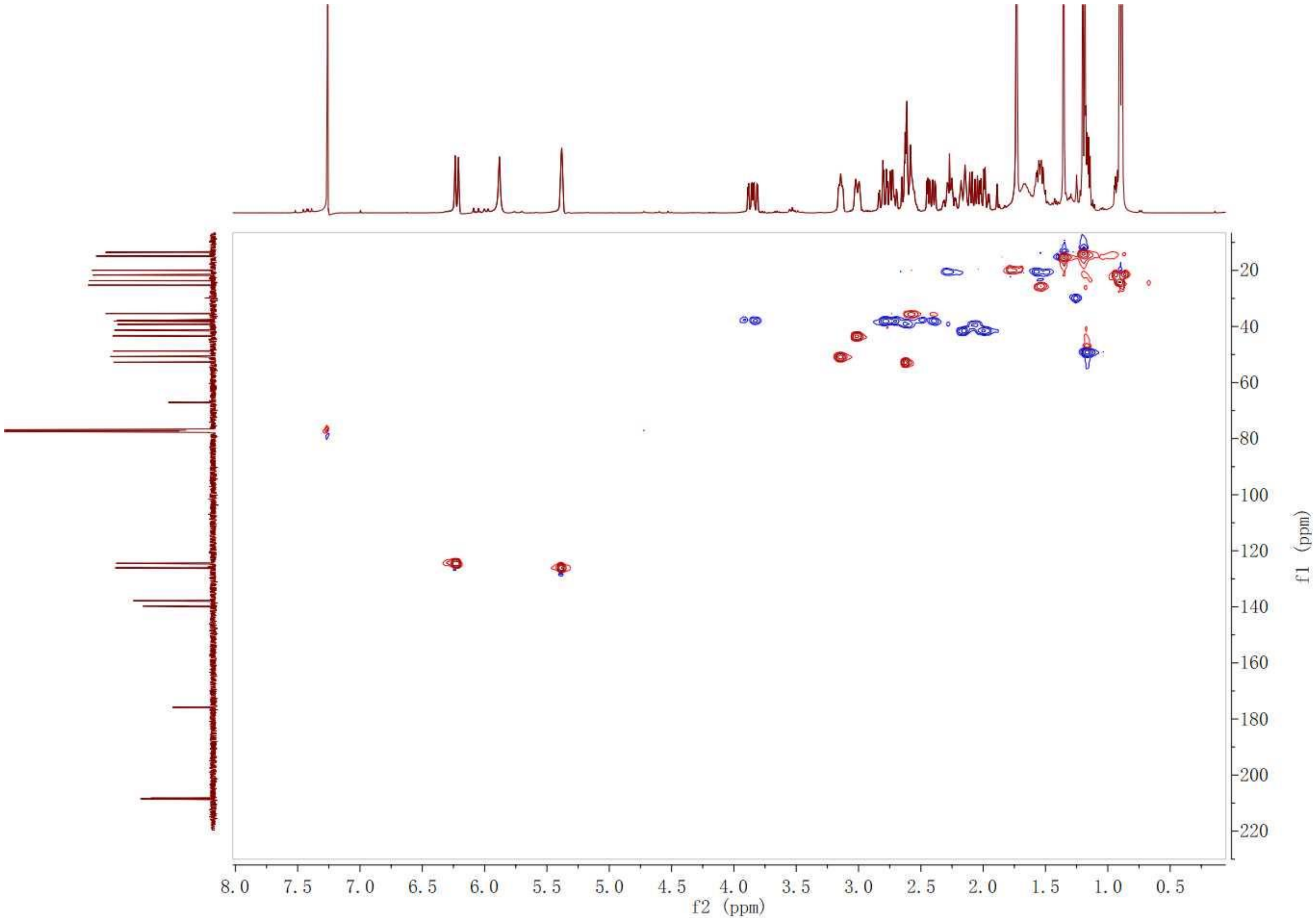

Supplementary Figure 74. HSQC spectrum of compound **11** in $CDCl_3$.

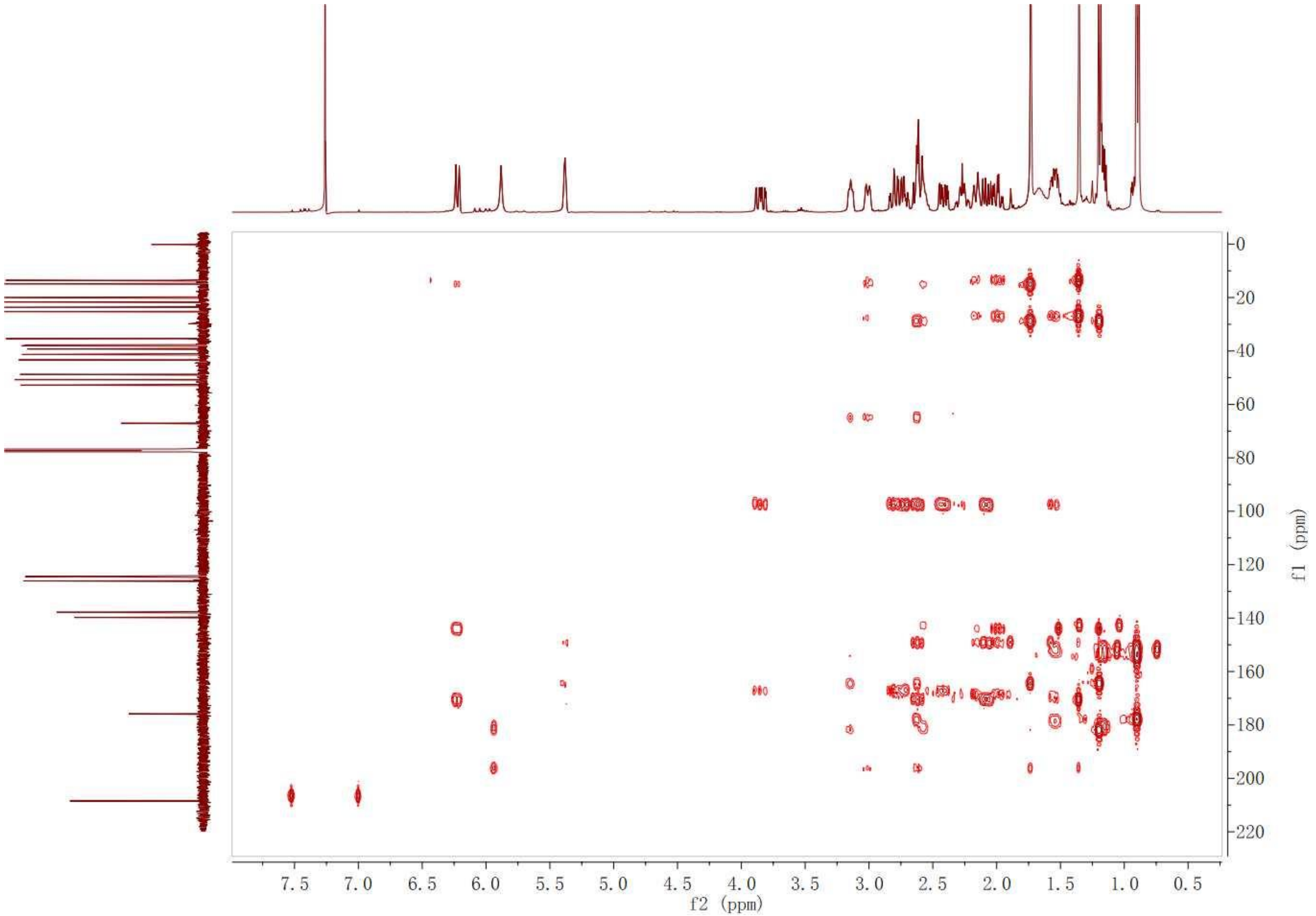

Supplementary Figure 75. HMBC spectrum of compound **11** in $CDCl_3$.

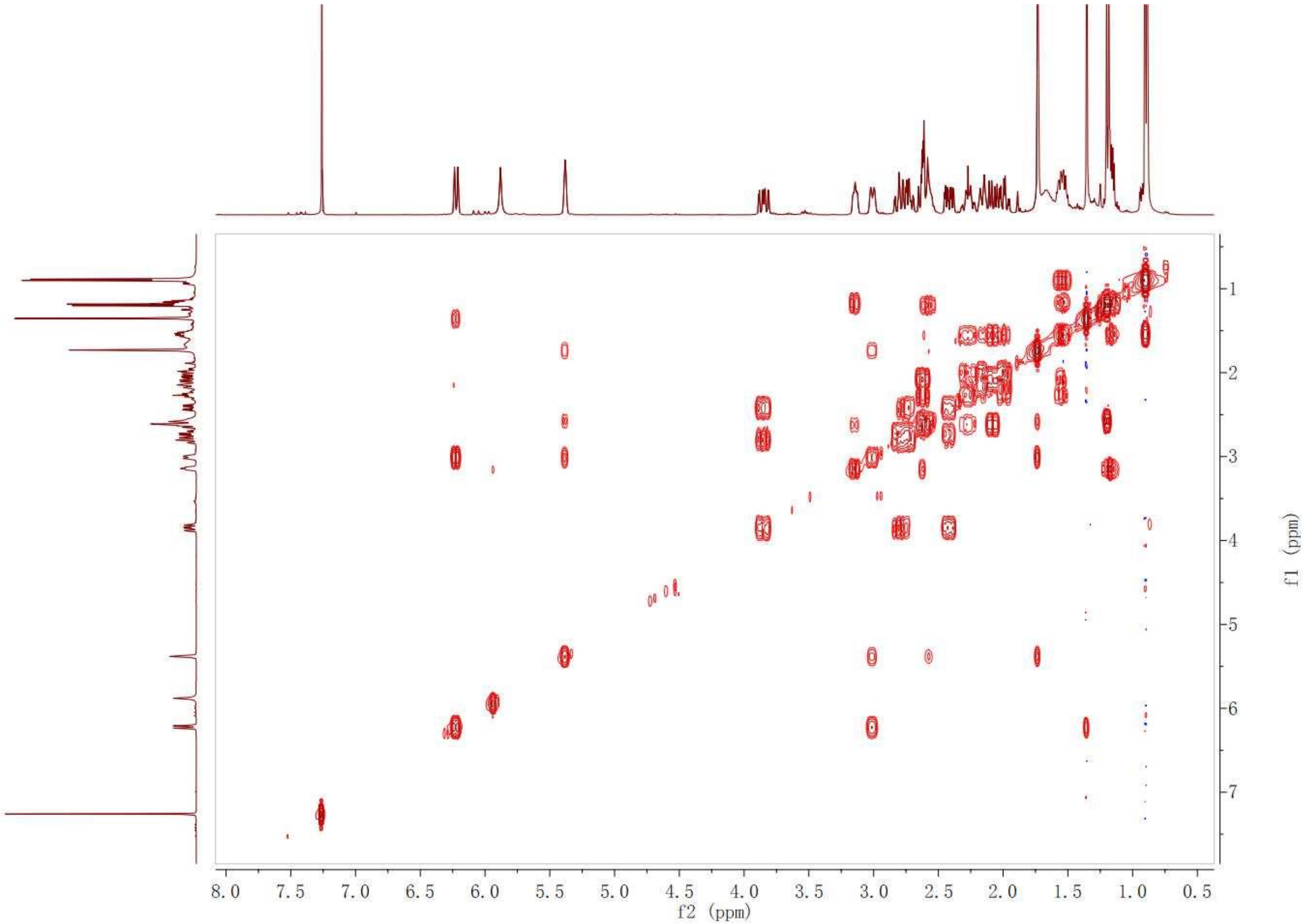

Supplementary Figure 76. ^{1}H-^{1}H COSY spectrum of compound **11** in $CDCl_3$.

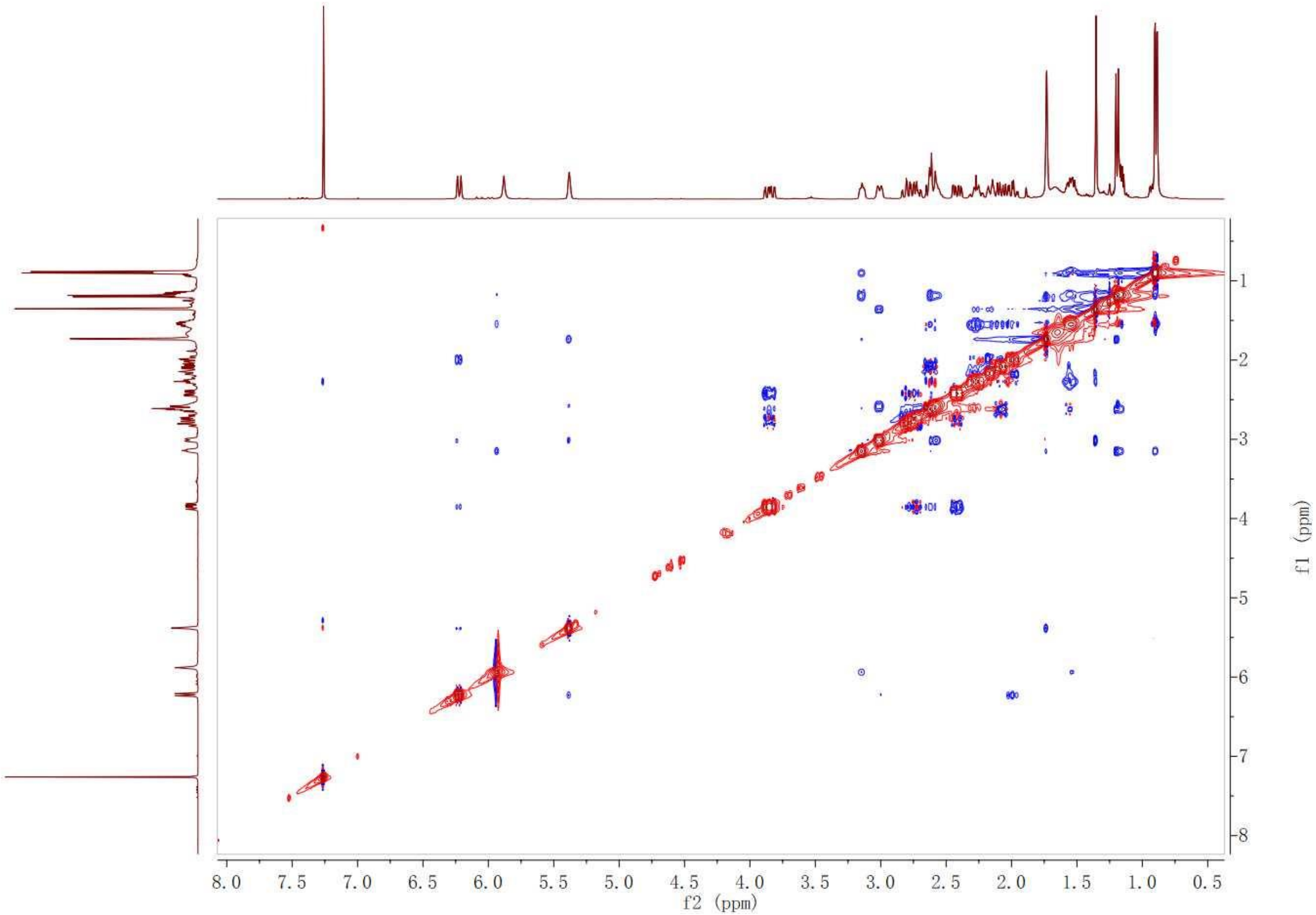

Supplementary Figure 77. ^{1}H-^{1}H NOESY spectrum of compound **11** in $CDCl_3$.

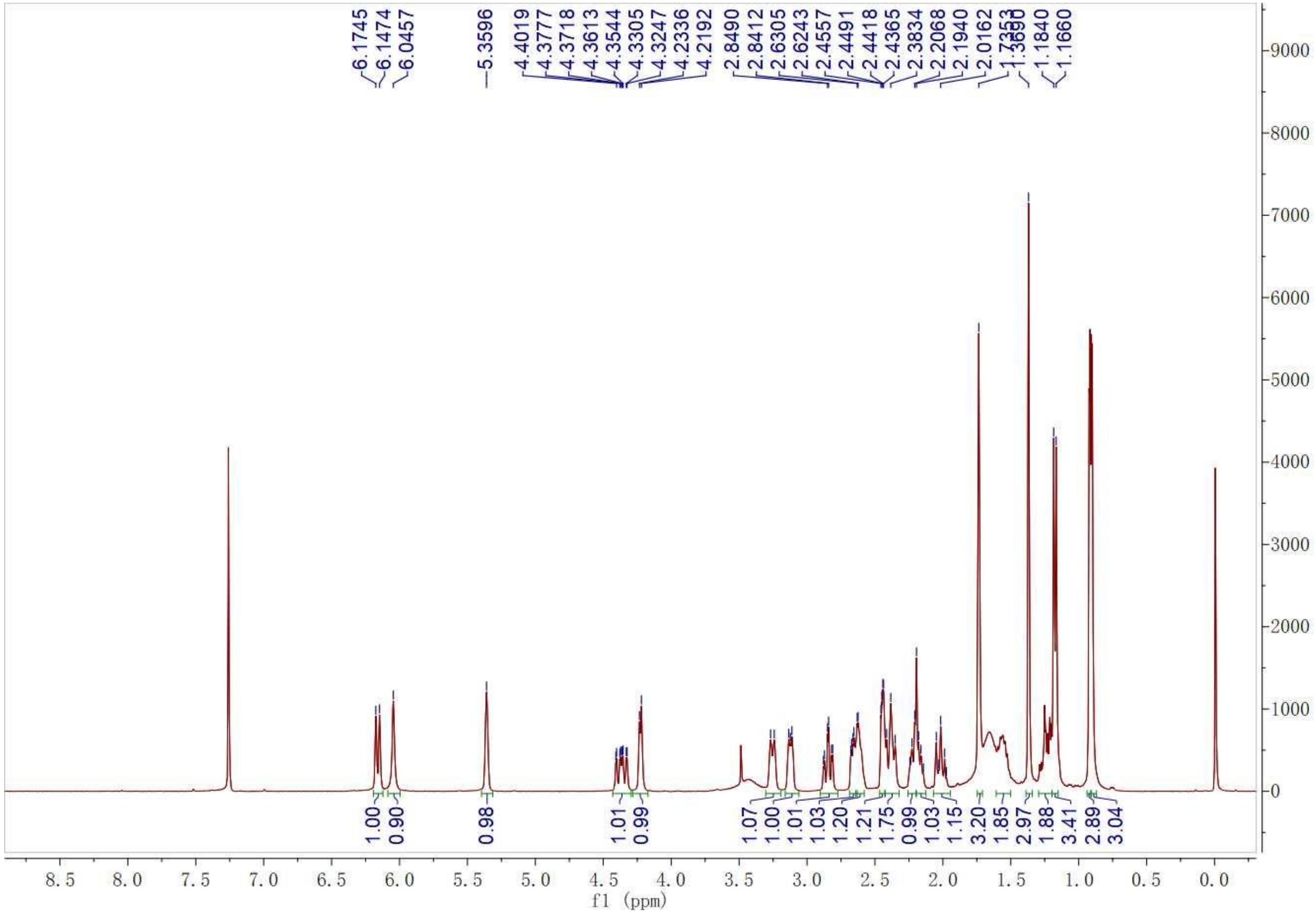

Supplementary Figure 78. ^{1}H NMR spectrum of compound **12** in $CDCl_3$ (400 MHz).

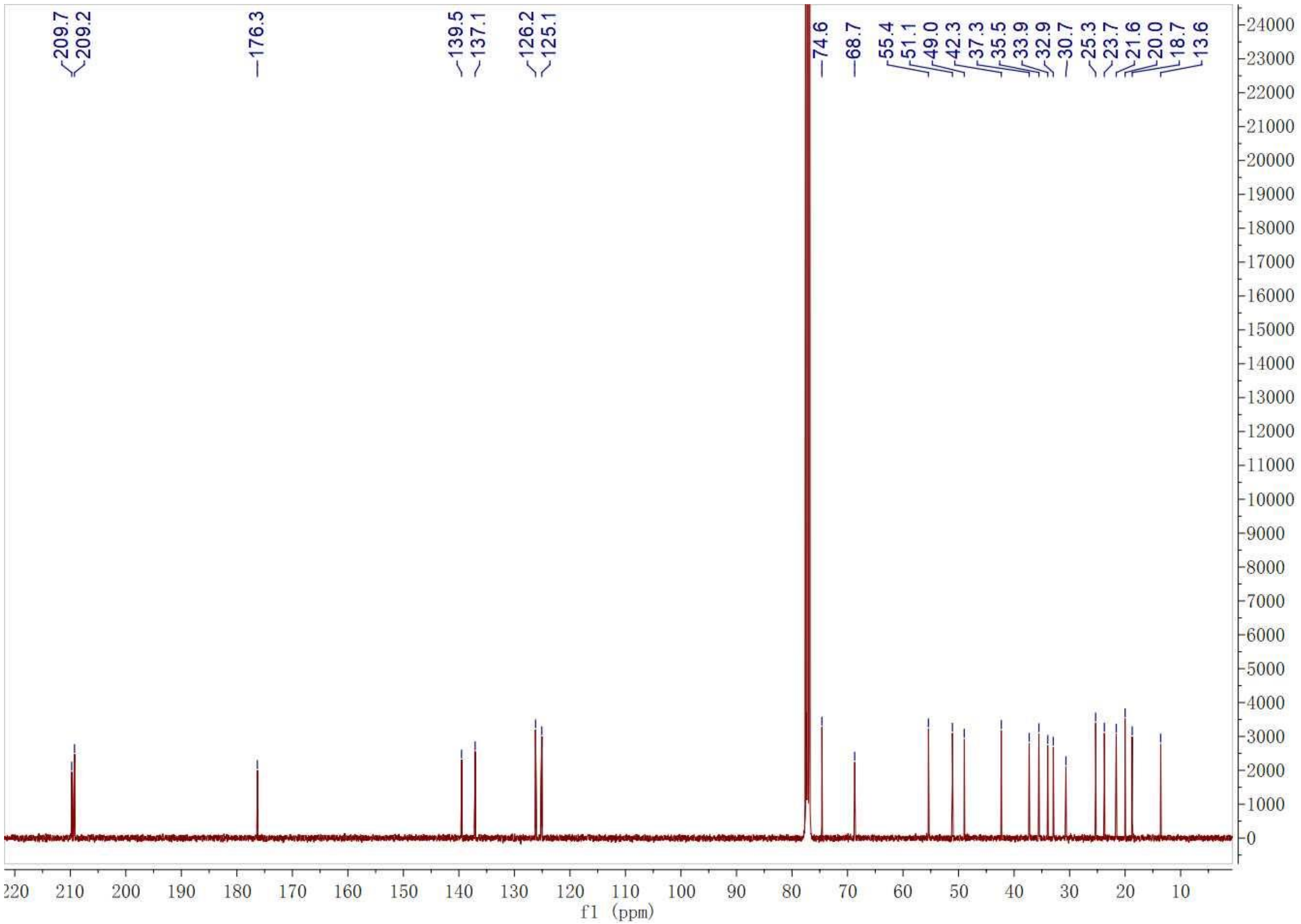

Supplementary Figure 79. ^{13}C NMR spectrum of compound **12** in $CDCl_3$ (100 MHz).

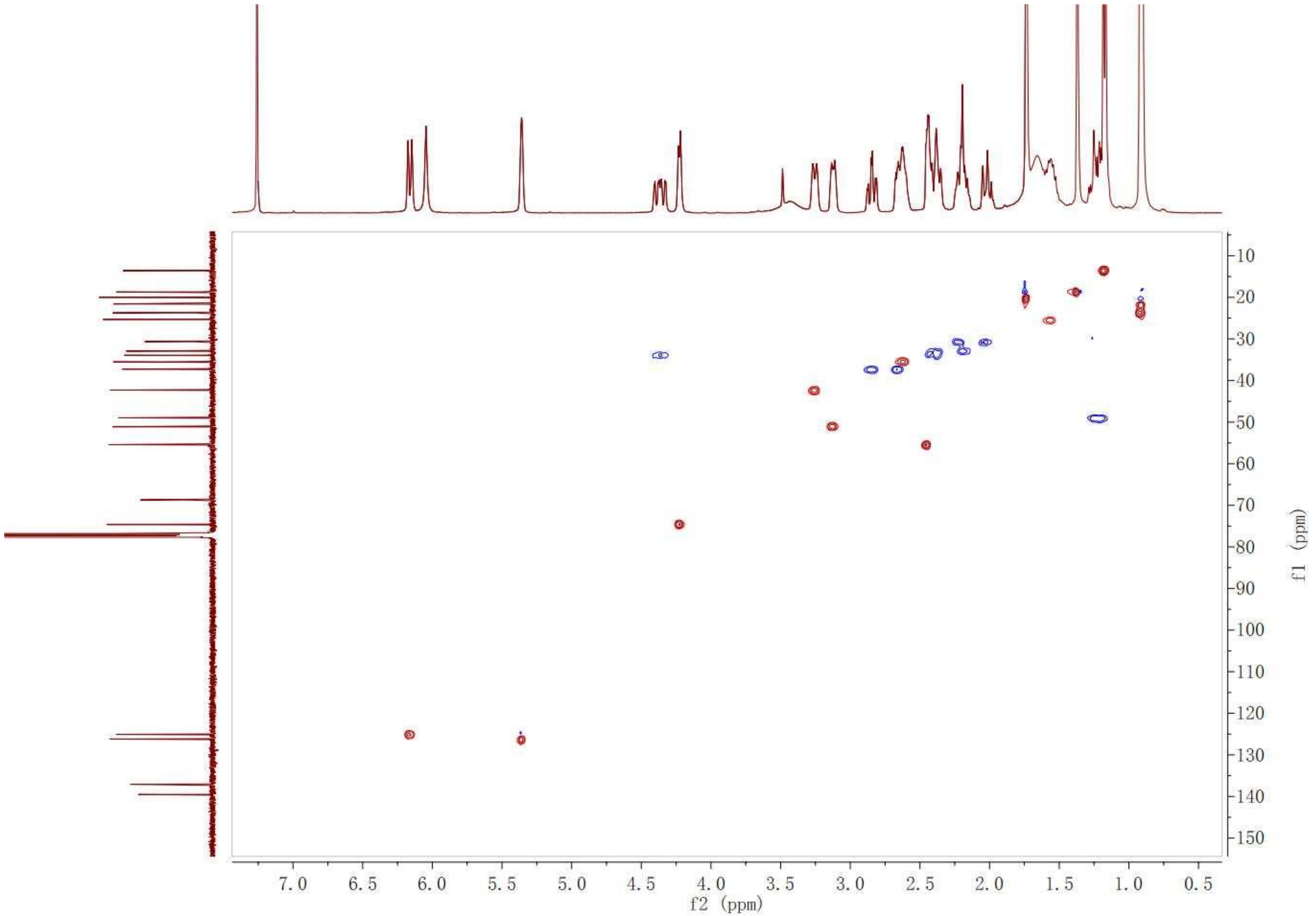

Supplementary Figure 80. HSQC spectrum of compound **12** in $CDCl_3$.

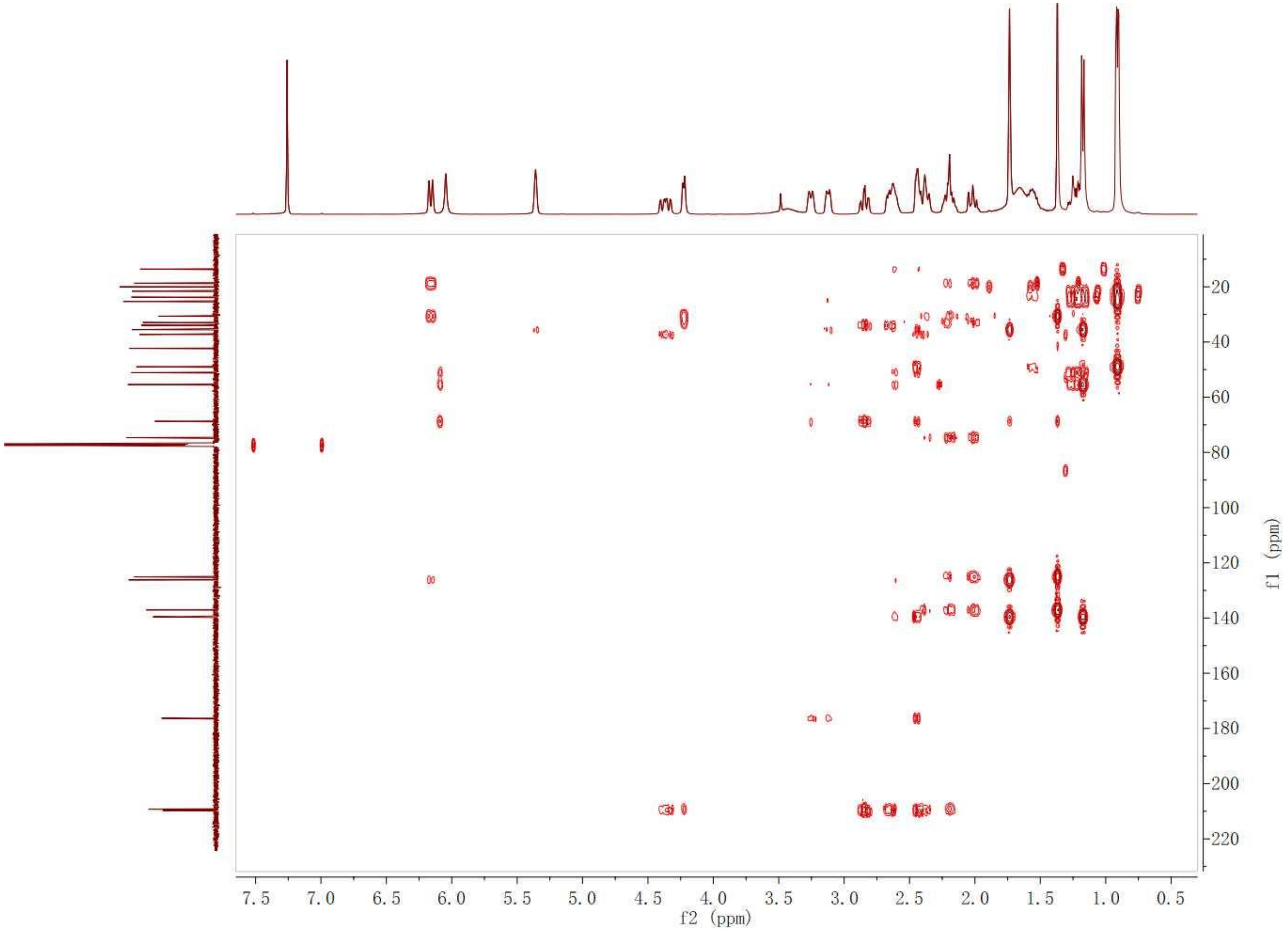

Supplementary Figure 81 . HMBC spectrum of compound **12** in $CDCl_3$.

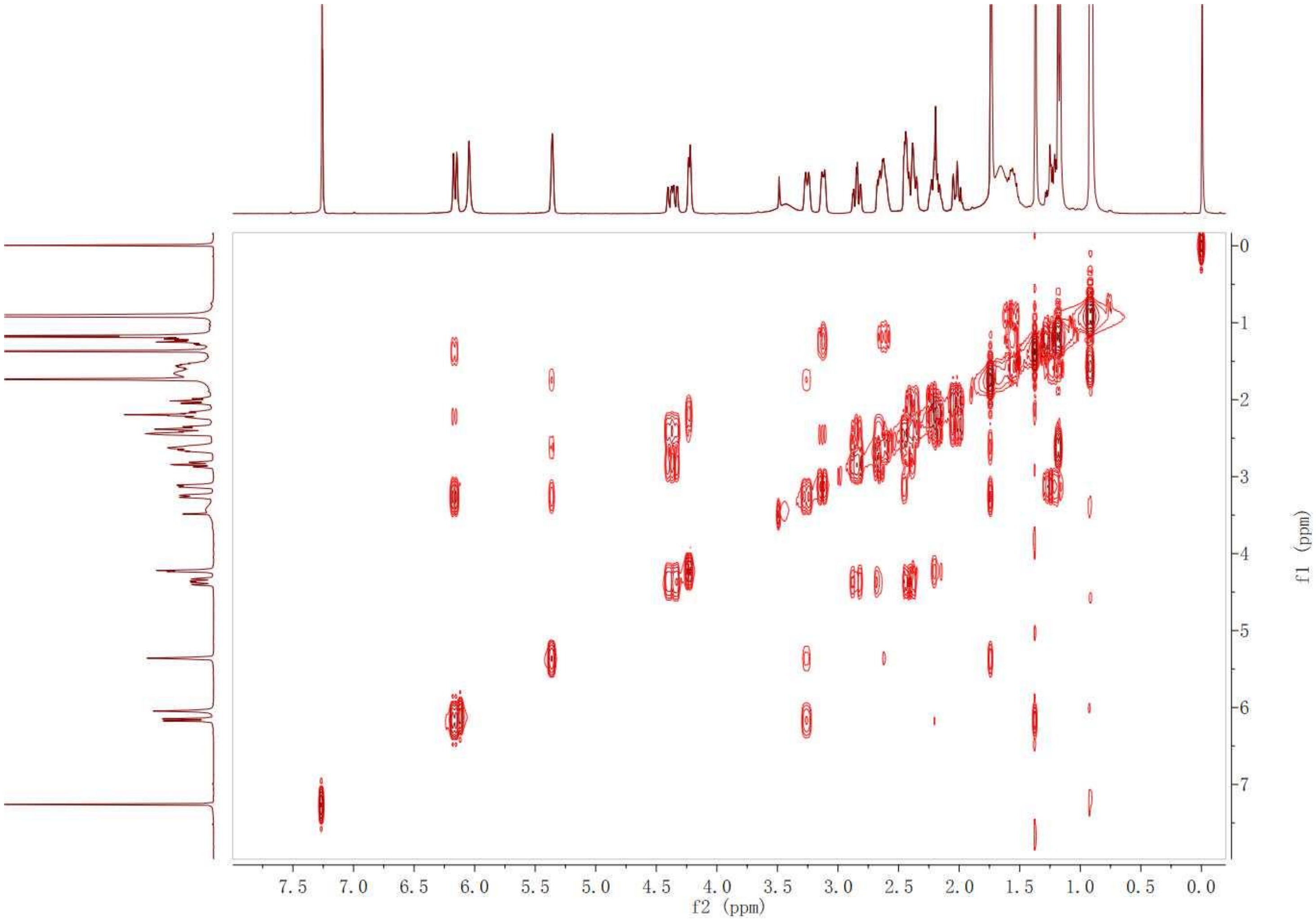

Supplementary Figure 82. ^{1}H-^{1}H COSY spectrum of compound **12** in $CDCl_3$.

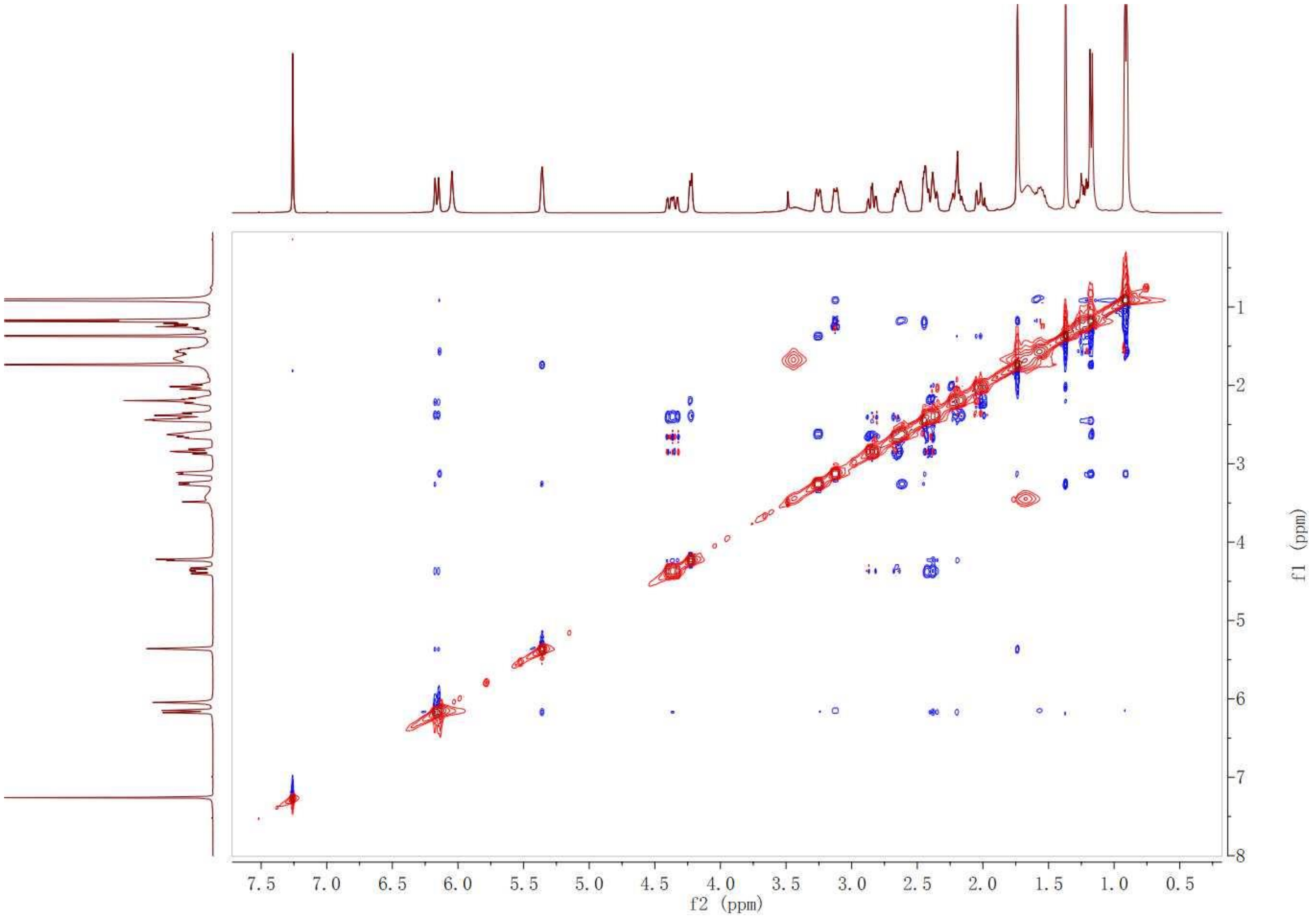

Supplementary Figure 83. ^{1}H-^{1}H NOESY spectrum of compound **12** in $CDCl_3$.

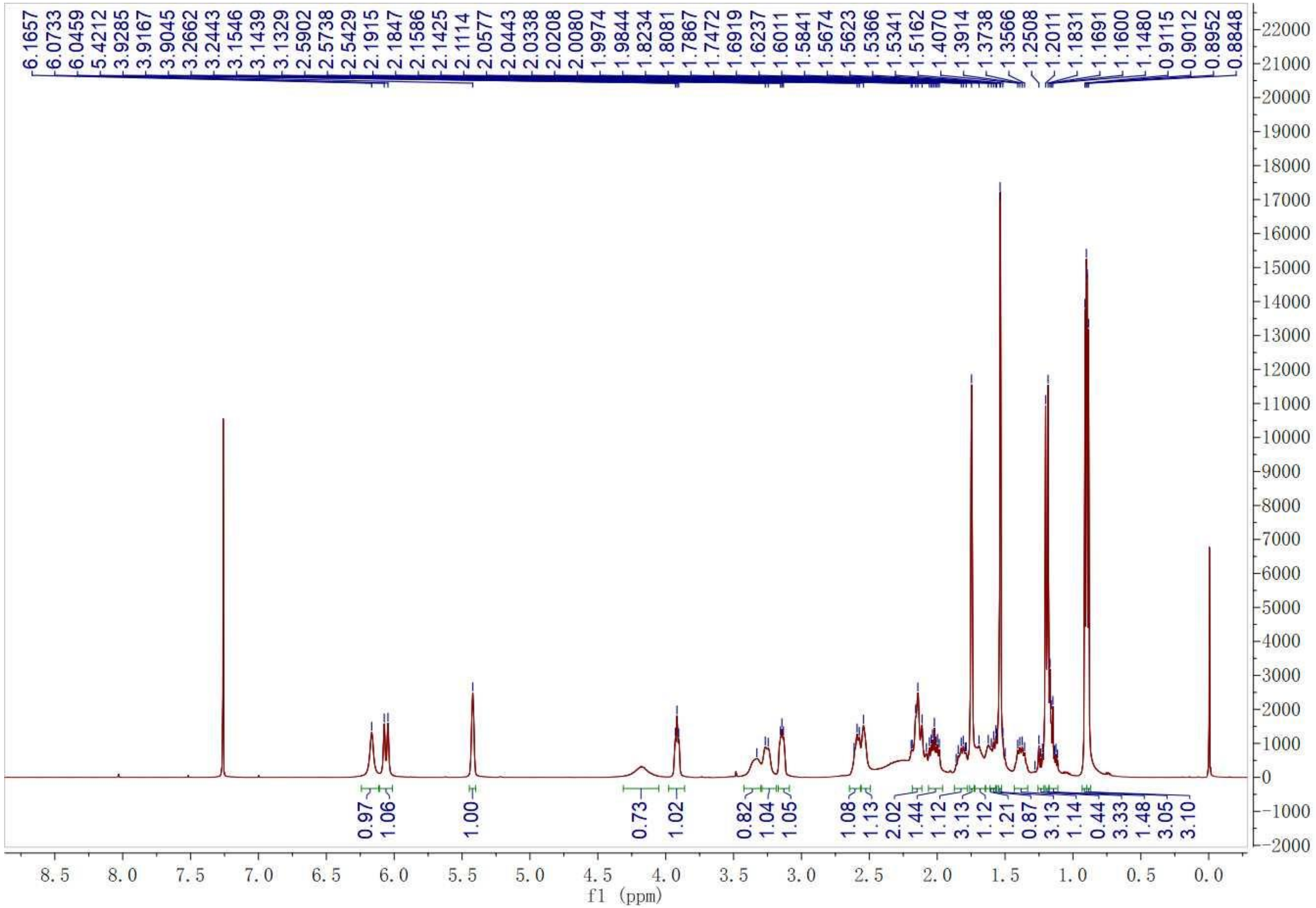

Supplementary Figure 84. ^{1}H NMR spectrum of compound **14** in $CDCl_3$ (400 MHz).

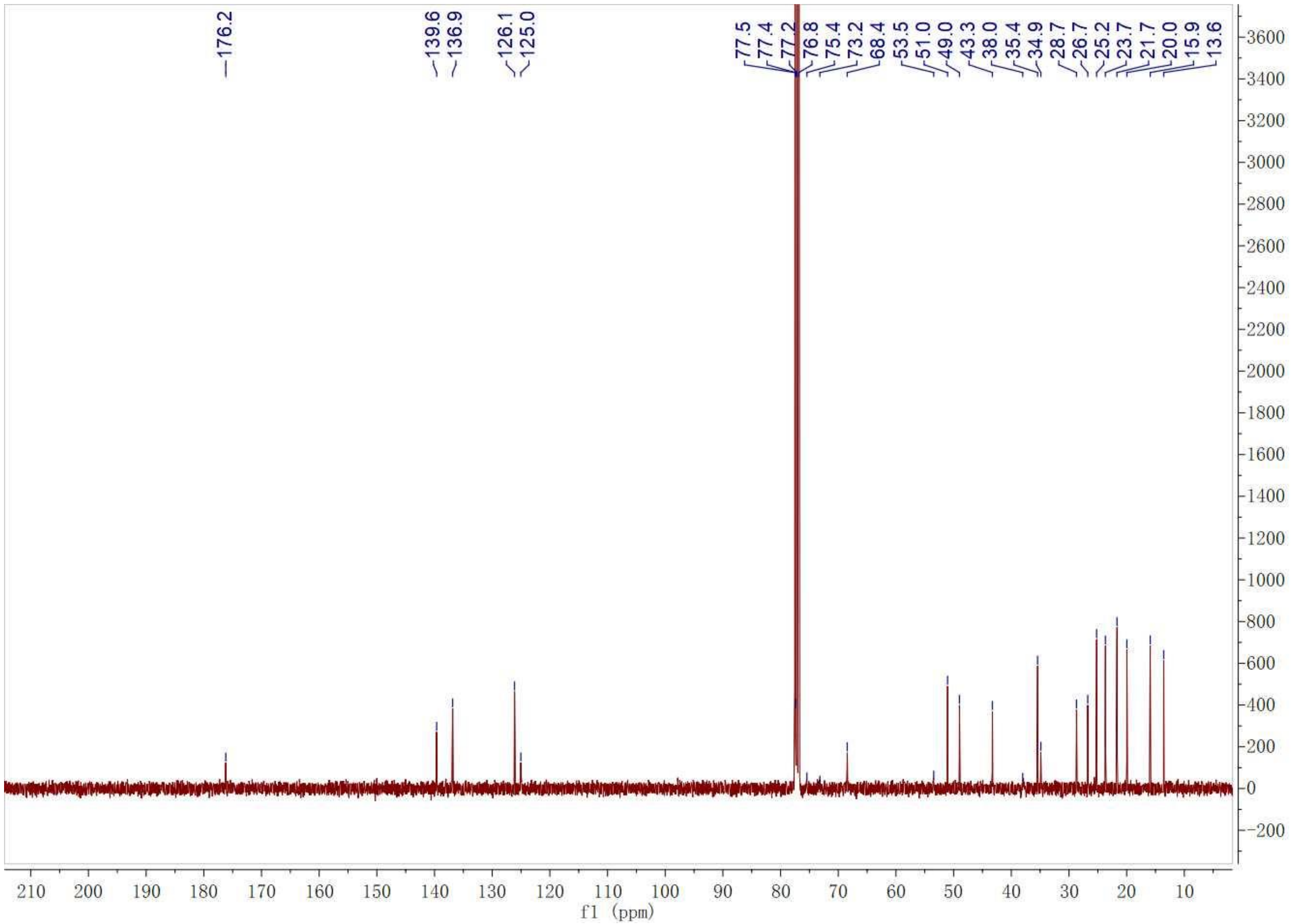

Supplementary Figure 85. ^{13}C NMR spectrum of compound **14** in $CDCl_3$ (100 MHz).

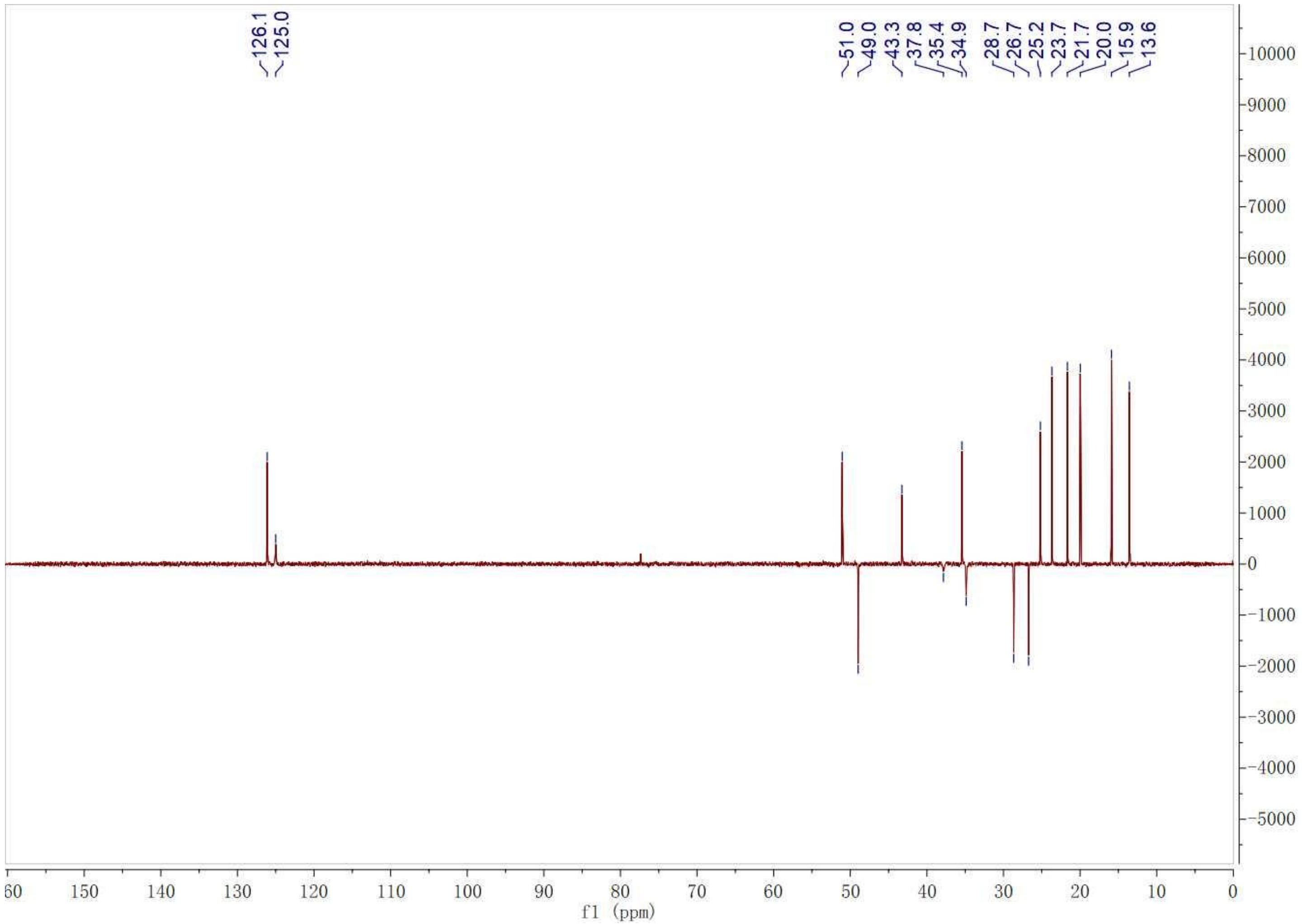

Supplementary Figure 86. DEPT-135° spectrum of compound **14** in $CDCl_3$.

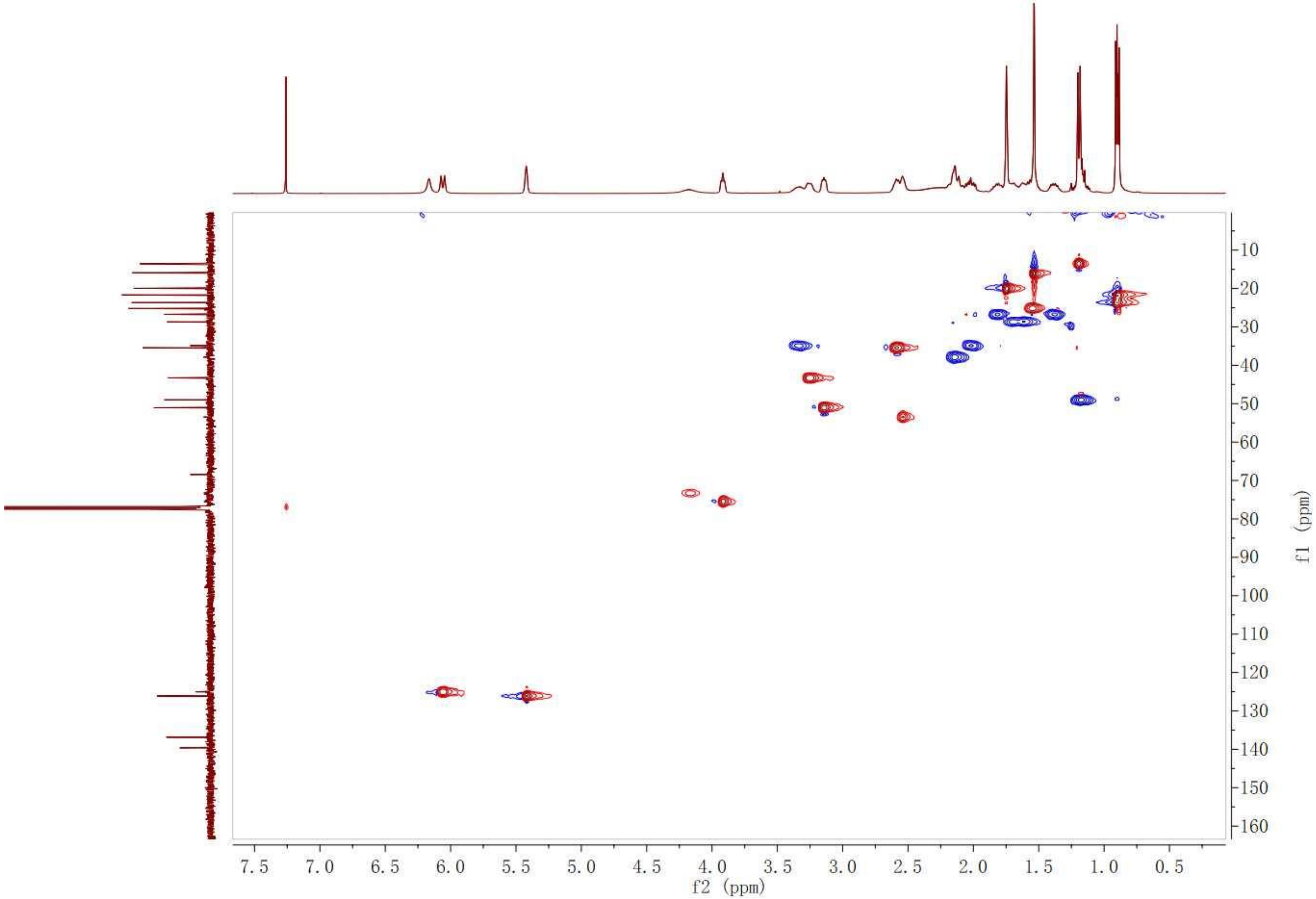

Supplementary Figure 87. HSQC spectrum of compound **14** in $CDCl_3$.

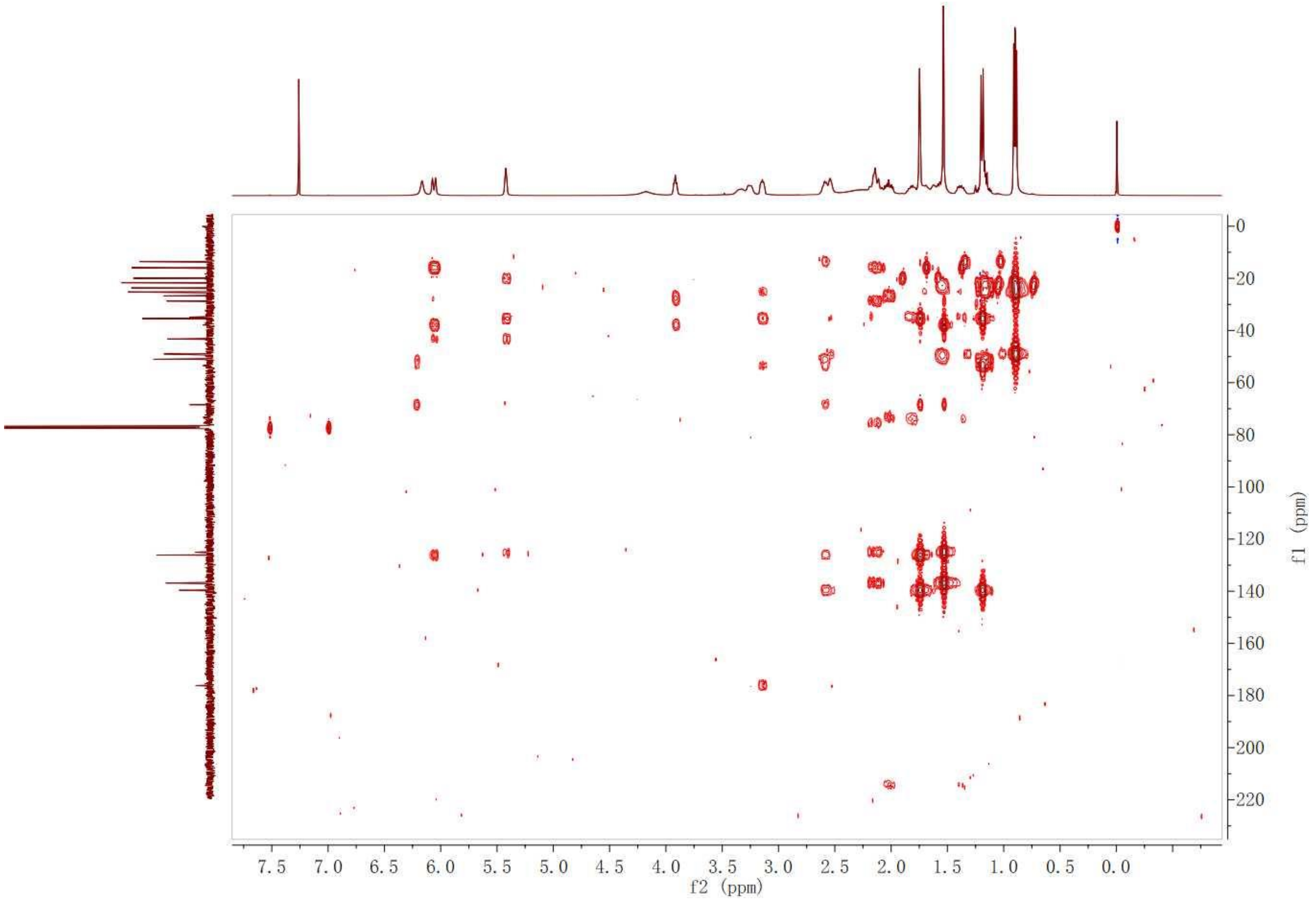

Supplementary Figure 88. HMBC spectrum of compound **14** in $CDCl_3$.

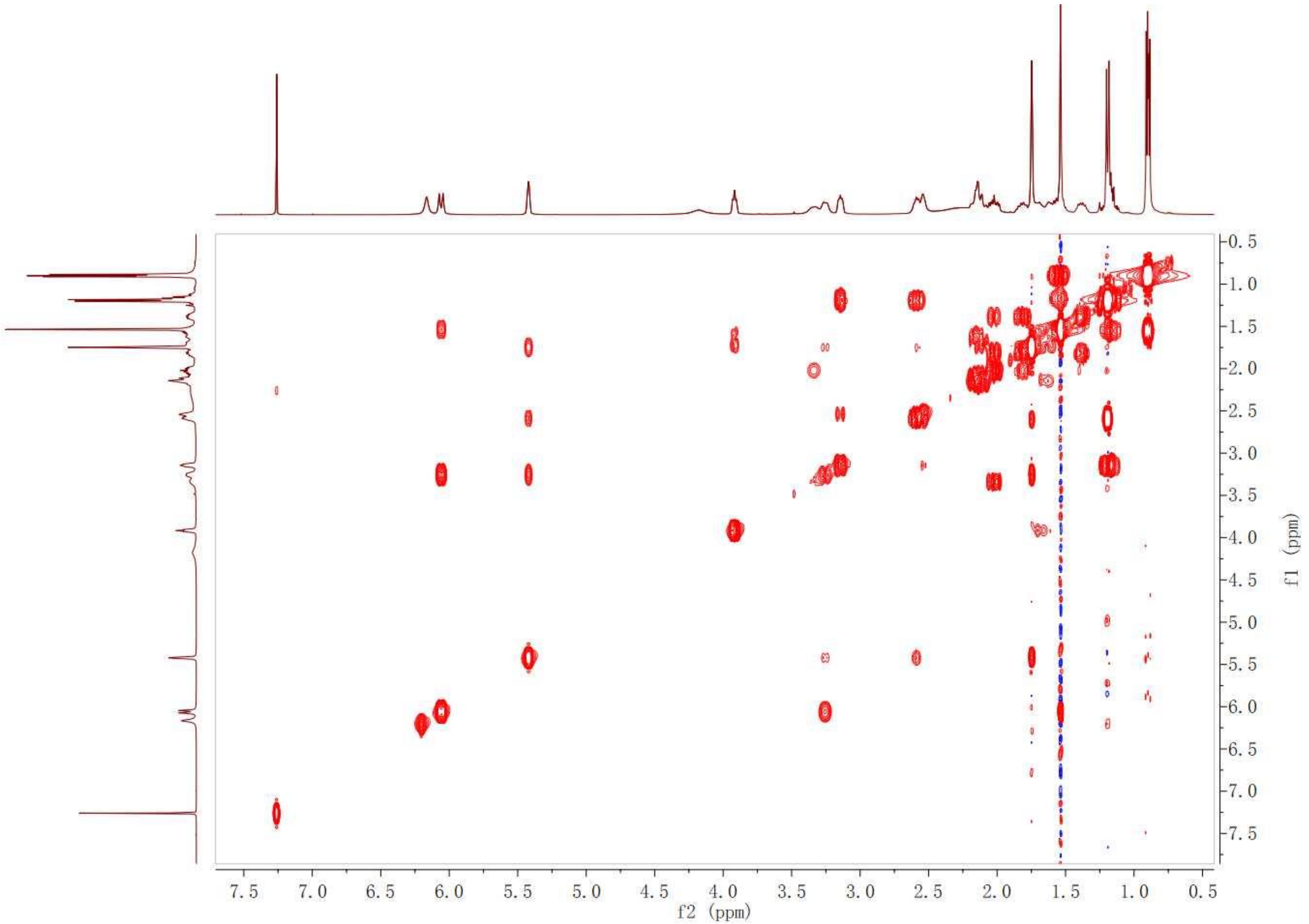

Supplementary Figure 89. ^{1}H-^{1}H COSY spectrum of compound **14** in $CDCl_3$.

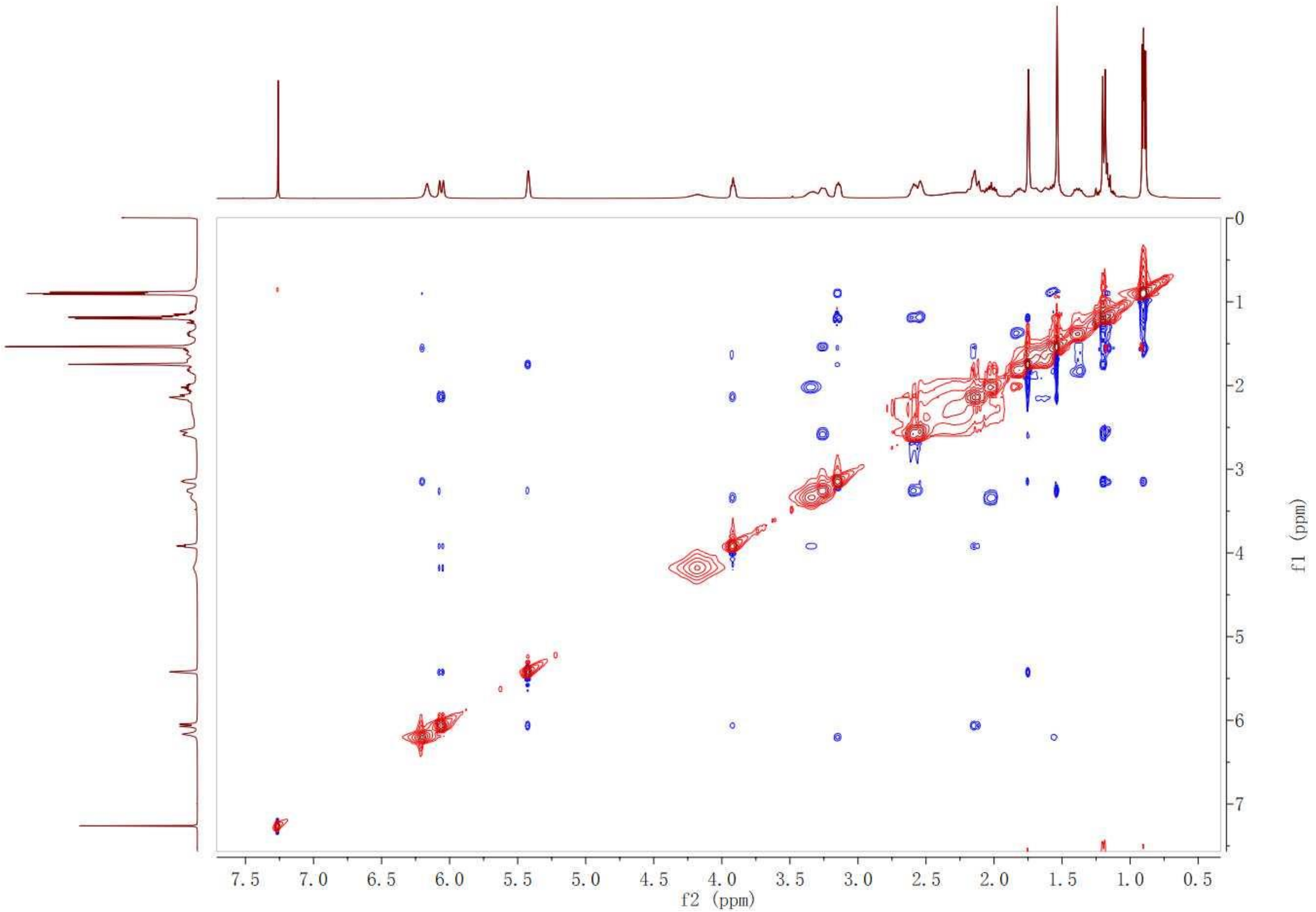

Supplementary Figure 90. ^1H-^1H NOESY spectrum of compound **14** in $CDCl_3$.

Sequence information

AspoA amino acid sequence

MRLTMRLITPAILALLPAAHAAATLQDWHFLNTTLDNRLHAVTPLALPCFSL
YNNHPHTPDQEACLNIQDHYTNASYRVDQVPAYVFSQSETCSPIPSQQCELDP
SNPSNPTAYTNISCNTGSLPAYYIDIQQASDVTAAFRFAAKTNTAISIKNSGHD
YNGRSSGPGSLSLRTRTLRSTTYHSSFTPSTCKTPIGKAVTLGAGVNFHEIYTF
AHDHKVTFLGGSGPTVGASGGWVLTGGHGVLSRAYGLGIDRVVEFELVTPD
GEHRVANACQNTDLFWALRGGGGSTYGVVLSSTHRVEPEAPLQLAYLALPA
NASNQTSSAFLDLLVNSTLSWAEDAWGGFGNGAATILATPLLSLSEAETSMA
DAIDFVTSHGGTGYVETLSSFYEMYTKYIIPSAGAVGSVRFNHNWLIPNSLFA
TATGQKKLREHMDWMGSVGLAPGLLETTPYLYSGNGNGKRNRNTTTSRVQ
PQPYRYTGGKDTTSSTPAWRNSAAVLIAEVGWGFNATVAEKKALAKTLVEA
SERVRELAPGSGAYANEAHPWVEDWQDAFWGGNYRRLAQLKKKFDPKGLL
GCWHCVGSDREEAWRAEVVGGKCLGRLI

AspoA gDNA nucleotide sequence

ATGAGACTCACCATGAGACTCATCACTCCAGCCATACTGGCTCTCCTCCCA
GCTGCCCACGCAGCAGCCACTCTCCAAGACTGGCACTTCCTCAACACCAC
CCTCGACAACCGTCTCCACGCCGTGACCCCTCTGGCCCTGCCATGTTTCTC
CCTCTACAACAACCACCCACACACCCCAGACCAAGAAGCCTGCCTCAACA
TCCAAGACCACTACACCAACGCCTCCTACCGCGTCGACCAAGTCCCCGCC
TACGTCTTCTCCCAGTCCGAAACCTGCTCCCCAATCCCCTCCCAGCAATGC
GAACTCGACCCCTCCAACCCCTCCAACCCAACCGCATACACCAACATCTC
CTGCAACACCGGCAGTCTCCCAGCCTACTACATCGACATACAGCAAGCAT
CCGACGTAACCGCCGCATTCCGCTTCGCCGCCAAAACCAACACCGCCATC
TCCATCAAAAACAGCGGCCACGACTATAACGGCCGAAGCAGCGGACCAG
GCTCTCTCTCCCTGCGCACCCGAACCCTCCGCTCAACCACCTACCACTCCT
CCTTCACCCCATCCACCTGCAAAACCCCCATCGGCAAAGCCGTCACCCTC
GGCGCAGGCGTCAACTTCCACGAAATCTACACCTTCGCCCACGACCACAA
AGTCACCTTCCTCGGCGGCTCAGGCCCCACAGTCGGTGCCTCCGGCGGCT
GGGTCCTAACCGGCGGCCACGGGGTGCTATCCCGCGCCTACGGACTCGGT
ATCGACCGGGTCGTCGAATTCGAACTCGTCACCCCCGACGGGGAACACCG
TGTCGCAAACGCCTGTCAAAACACCGATCTGTTCTGGGCACTCCGCGGCG
GCGGCGGAAGCACCTACGGCGTCGTCCTATCAAGTACCCATCGTGTCGAA
CCAGAAGCCCCTCTCCAACTCGCATACCTCGCCCTCCCCGCCAACGCATCG
AACCAGACATCCTCCGCCTTCCTCGACCTCCTCGTCAACTCCACCCTCTCC
TGGGCCGAAGACGCATGGGGCGGGTTCGGTAACGGCGCGGCCACCATCCT
CGCGACACCACTCCTCTCCCTCTCCGAAGCGGAGACGTCAATGGCCGACG
CGATCGACTTCGTCACCTCCCACGGCGGAACAGGGTACGTAGAGACACTA
TCCAGCTTCTACGAGATGTACACCAAGTATATCATCCCGTCTGCGGGGGC

GGTGGGGAGCGTGCGCTTCAACCACAACTGGCTCATACCGAATAGTCTGT
TCGCGACGGCTACCGGACAGAAGAAGCTGCGAGAGCATATGGATTGGAT
GGGGAGTGTTGGGCTCGCGCCGGGGTTACTCGAGACGACGCCGTACCTAT
ACAGTGGTAATGGTAATGGCAAGAGGAACAGAAACACCACCACGTCAAG
GGTCCAACCCCAACCCTACAGATACACCGGCGGAAAAGACACAACCTCCT
CGACCCCCGCCTGGCGAAACAGCGCGGCTGTCCTCATCGCCGAAGTCGGC
TGGGGATTCAACGCCACCGTCGCGGAAAAGAAAGCCCTGGCGAAGACGC
TGGTCGAGGCGAGTGAGCGGGTGCGAGAGCTTGCGCCGGGGAGTGGGGC
GTATGCGAATGAGGCGCATCCGTGGGTGGAGGATTGGCAGGATGCGTTCT
GGGGAGGGAATTATCGCCGGTTGGCGCAGTTGAAGAAGAAGTTCGATCCG
AAGGGGTTGTTGGGGTGTTGGCATTGTGTGGGGAGTGACAGGGAGGAGGC
GTGGAGGGCGGAGGTGGTGGGGGGGAAGTGTTTGGGGAGGTTGATTTAG

AspoB amino acid sequence

MTQTKLLLLSLFAVAAQSVSYDLPSQWDHQWLTQQPLGSDTTCTTSHLTAFQ
MSKTKDPIFFSTGGTDSFLSPKMLPLNSTAGEQWEFDGVSPDAKMAFVFGFY
RDPNYAILGSGNLRVSVEMLWPNGTRFAQVDYPTDSIIEECEWGTRGVWRA
DEFSYSFEVSRNLQTARVAMHTPQVSGVVYLDSESKPRYPDGKIYPSETSTSE
ALPYFHFVEPIPVAKSQVDLTILGEVYVWSDGVGGMERLWGAFSWFTCLQG
MNVVRLHAGPYALSLLSFTSNIKKGKEYPSIALFENGEPVFSSQRTEDSDTAD
YFTFTKTYDGKVTGTLRDKVTGYELELVSPGQKKHWTFIIDHESLAFEYILGG
GHGGSGFAGFVQGGRVGLEQFPGIALTEALTFPKKSPLFRAQYSED

AspoB gDNA nucleotide sequence

ATGACGCAAACCAAGCTCCTCCTTCTCTCTCTGTTCGCTGTCGCAGCGCAA
AGCGTCAGCTATGACCTCCCATCCCAATGGGACCACCAATGGCTCACCCA
ACAACCCCTCGGCAGCGACACCACCTGCACAACCTCCCACCTAACCGCCT
TCCAAATGTCCAAAACGAAAGACCCCATCTTCTTCAGCACCGGCGGAACC
GACTCCTTCCTCAGCCCAAAGATGCTCCCACTGAACAGCACCGCCGGCGA
ACAATGGGAATTCGACGGCGTTTCCCCAGACGCCAAAATGGCCTTCGTCT
TCGGCTTCTACCGCGATCCCAACTACGCGATCCTCGGCTCAGGCAACCTGC
GCGTGAGCGTGGAGATGCTCTGGCCGAACGGCACGCGGTTCGCGCAGGTG
GACTATCCCACTGACTCTATCATTGAAGAATGCGAATGGGGAACCCGCGG
GGTGTGGCGCGCCGACGAATTCAGCTACAGCTTCGAGGTATCGAGGAATC
TGCAGACCGCACGGGTAGCCATGCATACGCCGCAAGTATCTGGGGTGGTG
TATCTGGACTCAGAGAGCAAGCCGCGGTATCCGGATGGGAAGATCTACCC
CAGTGAGACGTCCACGTCAGAGGCGCTGCCGTATTTCCATTTCGTGGAGC
CGATCCCCGTCGCCAAGTCGCAGGTTGATCTGACGATTCTGGGGGAAGTG
TATGTGTGGAGTGACGGGGTAGGAGGCATGGAGAGACTGTGGGGGGCGT
TCAGCTGGTTTACGTGTCTGCAGGGGATGAACGTCGTGCGGCTGCATGCG
GGCCCGTATGCCCTGTCCTTGCTGTCGTTCACCTCGAATATCAAGAAGGGC
AAGGAGTATCCGTCGATTGCGCTATTTGAGAACGGGGAGCCTGTCTTCTCT

AGCCAGCGCACGGAGGACTCGGACACGGCGGACTACTTCACCTTCACGAA
GACGTATGATGGGAAGGTCACCGGGACACTTCGGGACAAAGTGACTGGCT
ACGAGCTGGAGCTGGTGTCTCCGGGGCAGAAGAAGCATTGGACGTTCATT
ATTGATCACGAGAGTCTGGCGTTTGAGTATATCCTTGGTGGAGGGCACGG
AGGCAGTGGATTCGCCGGCTTTGTCCAAGGCGGCCGAGTAGGACTGGAGC
AGTTCCCGGGGATTGCCCTGACAGAGGCGCTGACTTTCCCGAAGAAATCG
CCTCTGTTTCGCGCGCAGTATTCGGAGGACTAG

<u>AspoC amino acid sequence</u>
MTGSSAVRPGFATIHEFFPGNTFYNFECLRILSTAPYGGCDPAEFLTAIAAIKPS
DPETWATAWGHAASQAERIAEDALARGDTLAARDAFLRASSYTRASGYMRI
NGPTLDRHDPRALPVARKTQTLFRKALPFLDCDARIVDIPYRPTQYSKPVSLP
GYVYIPSPQNRLPDGKIPILLNTGGADSVQEELYYIHPQAGHARGYAVITFEGP
GQGIILREKGLYMRPDWEVVTGQVLDWLEGYAATLQQEEGLTLDLSRIAVV
GASMGGYYALRAASDPRIKACLSIDPFYDMWDFGTRHISGLFMAAWTGGWV
SDATIDRVIGAGMYLNFQLRWEVGVTTAFWGIESPARILREMKRYSLQGGFL
ARVQCPVFVSGAGKSLYFDTEEHTMRVFGDLKHLGERGRTLWMPSRPEEGG
LQAKIGAFGLVNTKAFGFLDGVFGVKRVVEADS

<u>AspoC gDNA nucleotide sequence</u>
ATGACCGGTTCCTCAGCAGTCCGCCCAGGTTTCGCCACCATCCACGAATTC
TTCCCCGGAAACACCTTCTACAACTTCGAATGTCTGCGGATCCTCTCCACG
GCCCCCTACGGCGGCTGCGATCCCGCTGAGTTCCTCACCGCAATCGCAGC
CATCAAGCCCAGTGACCCTGAGACCTGGGCCACAGCCTGGGGCCACGCCG
CCTCGCAAGCCGAGCGTATCGCAGAAGATGCCCTCGCAAGAGGCGACACC
CTCGCCGCGAGAGATGCCTTCCTCCGCGCAAGCTCGTACACCCGCGCAAG
CGGATACATGCGTATCAACGGACCGACGCTGGACCGACATGACCCTCGCG
CGTTGCCTGTCGCGCGAAAGACGCAGACGCTGTTCCGAAAGGCCCTACCG
TTTCTCGACTGTGATGCTCGGATCGTCGATATCCCCTATCGCCCGACGCAG
TATAGCAAACCGGTCTCCCTCCCGGGATACGTGTACATCCCCTCGCCCCAG
AACAGACTGCCAGACGGGAAGATCCCCATCCTGCTCAACACCGGCGGGGC
CGACTCCGTCCAAGAAGAGCTGTACTATATCCACCCGCAGGCAGGCCACG
CGCGCGGATACGCCGTCATCACCTTCGAAGGCCCCGGACAGGGGATCATC
CTGCGTGAGAAGGGCCTCTACATGCGGCCGGACTGGGAGGTGGTCACCGG
GCAAGTGCTCGACTGGCTAGAGGGGTACGCGGCCACTCTCCAGCAAGAAG
AAGGACTGACGCTGGACCTCTCACGGATTGCGGTTGTTGGCGCCAGCATG
GGTGGTTACTATGCTCTCCGTGCAGCCAGCGACCCGCGCATCAAAGCCTG
CCTCTCCATCGACCCGTTCTACGACATGTGGGACTTTGGCACGCGCCACAT
CTCCGGTCTGTTCATGGCGGCCTGGACGGGCGGCTGGGTCAGCGATGCAA
CGATCGACCGCGTCATCGGCGCGGGCATGTATTTGAATTTCCAGCTGCGG
TGGGAAGTCGGCGTCACGACGGCGTTCTGGGGGATTGAGAGTCCCGCGCG
GATCCTCCGCGAGATGAAGCGGTATTCCCTGCAGGGGGGATTCTTGGCCC

GGGTGCAGTGTCCGGTGTTTGTGTCGGGGGCGGGGAAGAGTCTCTATTTC
GACACTGAGGAGCATACGATGCGGGTGTTTGGCGATCTGAAGCATCTGGG
GGAGCGAGGGAGGACGCTGTGGATGCCGAGTCGGCCGGAGGAGGGCGGG
CTCCAGGCGAAGATTGGGGCGTTCGGGCTGGTGAATACGAAGGCGTTTGG
GTTTTTGGATGGGGTGTTTGGGGTGAAGCGGGTTGTCGAGGCTGATAGTT
AG

AspoD amino acid sequence

MAEAITSVPAKSSFSAFWWASKHPPADPTTSFAGKTVLITGPNAGLGYEAAL
KFAALGASQLIFGVRSLARGKEAKASIEAKTKCAPTVIHLLQLDMASYTSIESF
AQEVSSKFPVVHAAVLNAGVAPPAYKRSPEGWEMALQVNVISTAYLAILLLP
KLRATGIAAGEPTHLEFVTSVGHGDIAPEQVRDSRSILGKVNDEANFKFTAQY
SITKLLEMWVMRHVAAAARSSEVIVNGACPSLCKSSLGRDFSIMLRAPDSLM
KSIIGRTAEQGSRILVSAVTTDQKAHGGFWSHDRVAVPGVLVTSDEGKKLSE
QFWKEIMDELSKQNPDVEKLLSEGS

AspoD gDNA nucleotide sequence

ATGGCCGAAGCAATCACCTCCGTCCCCGCCAAATCCTCCTTCAGCGCCTTC
TGGTGGGCATCCAAACATCCCCCTGCAGACCCAACCACCTCCTTCGCCGG
CAAAACCGTCCTCATCACCGGCCCCAACGCCGGACTCGGCTACGAAGCCG
CGCTCAAGTTCGCCGCCCTGGGCGCATCCCAGCTCATCTTCGGCGTGCGCT
CCCTCGCCCGCGGCAAAGAAGCCAAAGCCAGCATCGAGGCGAAGACCAA
ATGCGCCCCCACCGTCATCCACCTCCTCCAGCTGGACATGGCCAGCTACA
CCTCCATCGAGTCTTTCGCGCAAGAAGTCAGCAGCAAGTTCCCCGTCGTG
CATGCCGCCGTGCTGAACGCCGGCGTCGCTCCGCCCGCGTACAAGCGCAG
CCCCGAGGGATGGGAGATGGCGCTACAGGTCAACGTCATCTCGACCGCGT
ACCTGGCTATCCTCCTCCTACCGAAGCTCCGCGCGACAGGCATCGCAGCC
GGCGAGCCCACGCATCTGGAGTTTGTAACCAGCGTCGGCCACGGCGATAT
CGCTCCGGAACAAGTGCGCGATTCGCGGAGTATCCTGGGCAAGGTCAACG
ACGAGGCGAACTTCAAGTTCACGGCGCAGTACTCCATCACCAAGTTGCTG
GAGATGTGGGTCATGAGACATGTTGCTGCTGCGGCGCGGTCTTCCGAGGT
GATTGTCAACGGGGCGTGTCCGAGTCTGTGTAAGAGTAGTCTGGGACGCG
ACTTTAGTATCATGTTGCGCGCGCCGGATAGTCTGATGAAGTCGATTATTG
GGCGGACGGCGGAGCAGGGGAGTCGCATTTTGGTGAGTGCTGTGACGAC
GGATCAGAAGGCGCATGGGGGGTTCTGGTCGCATGATCGGGTTGCTGTGT
GAGTTTTCTCTTTTCCTTCTTATATTCTTTCTATTCTGGTGTGAAGATGGGC
TAATGCTGTGTATAGACCTGGTGTGCTGGTGACGTCGGACGAGGGGAAGA
AACTCAGCGAGCAGTTCTGGAAGGAGATCATGGATGAGCTTTCGAAGCAG
AATCCGGATGTTGAGAAGCTTCTGTCGGAGGGGAGTTGA

AspoE amino acid sequence

MATTSATSTTKGHNQPPREPIAIVGSACRFPGGASSPSKLWKLLEQPRDVLKEI
PPDRFSVDGFYHPDNMHHGTSNVRHSYILDDDIRVFDAQFFGIKPVEANSIDP
QQRLLMETVYEGIESAGLQLNQMKGSQTGVYVGLMSNDYADMLGNDQESF
PTYFATGTARSIVSNRVSYFFDWHGPSMTIDTACSSSLVAMHQAVQYLRSGD
GSDVAIAAGTNILLNPDQYIAESKLKMLSPDGRSQMWDEKANGYARGDGIA
VVVLKTLSQALRDGDHIECIVRETHINQDGKTKGITMPSATAQTALIRATYKN
AGLDITKPSDRPQFFEAHGTGTPAGDPIEAEAIHTAFFGYKGLSKEIDALSVGSI
KTIIGHTEGTAGLAAVLKASLALQAGVIPPNLLFDKVNPKVKPFYGNLQIQTQ
SKPWPEIAPGAVRRASVNSFGFGGANAHAILEAYEPSSTPTEDTSANVFTALN
VSAMSETALRRTLKKYAEYLEENPSVDLRSLALTVNTRRSTFPVRTSVFGSTM
EELSQRLRERSEAEGKTLTPVAPTSLASSPKIFGVFTGQGAQWKRMGAILLDT
SPRVVAILDKLEKSLAELPDGPSWSIKGEILADENSRVNEAVISQPLCTAVQIV
LVDLLRSAGVKFHAVVGHSSGEIGAAYAAGYLSASDAIRIAYYRGLHLCLAQ
GPNGQQGAMMAVGTSFEDAQELCDLPAFKDRMSIAASNSSASVTLSGDLDAI
EWANDVFDDEKKFARLLKVDKAYHSHHMLACSDAYRKSMTDCGITVLQPE
RNGTTWISSVYGEDALDYRHEMSAEYWISNMVSPVLFSQAVEFALADQGPF
DIGIECGPHPALKGPALQVIQEMLGSSIPYTGLLSRGRPDNQALAEGISYLWQA
LGEGIVDYRLFDKFIAGPDAPEPQIVANLPSYAWDHDRAFWHESRQYWANRT
KEDPPHEILGTKCPDGTNQQHRWRNMLRPREIPWIAGHQIQEQMVFPAAGYV
SAALEAVKLVTRGQSIGAIEIEDFIIGQAIAFNDEYASVETQFTLTDISVEKDIW
SASFSFYSASPKSSRSLDLNASGKLRVTLGEPQDDLLPPHLSPEFNMIDVDSER
FYDALKKLGFGYTGPFKALGSLKRRMGVATGTIANPTSTDPAHDLLLHPATF
DNAIQSIILAYCYPNDGRLWSVQLPTGIKKIKINPVLCNQYAGKNALLCFKAS
TSDDRSAEIGGDVDIYDEQGNNALMQLEGLQTKPLANATAANDSPLFLETIW
DIEGPSREAAVADRPDMQLKTELAFDVERVAFFYLRYLDSVATREEREKAES
HHKIFFEYIDHTVANVKSGTAQFAKREWMYDTHDEILDIIGKYPDSLDMKLM
HAVGEHLLPVIRGETTMLEYMREDNLLNDFYVHAIGFDEYTENLAQQVSQFS
HRYPHMNILEIGAGTGGATKRIFSKLGKRFGSYTYTDISAGFFEKTRETFREYE
HMMTFKALNIEKDPVEQGFTEQSYDLVVASLVLHATHEMETTMRNVRRLLK
PGGYLIMLELGDYIEMRTGLIFGSLPGWWMGYDDGRKLSPCMSEEDWSTCM
QKTGFSGVDAIVPRQSELPISLAVLTGQAVDDHVNFLRDPLTPGSVNFVESNL
TIIGGTTSKISGVVEEAAKSLNRFYEKIVTARSLAEMDTAQVPFMGSALFLTDL
DEPIFENVTEEALTALKQLFKQSRTCLWVTQGARDDNPFQNMSVGLGRVVK
LEMTHLRLQSLDFDIDTEPSAAAIMQRLLQFEAMAQWEQSGESKDLLWSVEP
EIGYDHSKAIIPRLMPNPVRNARYNSSRRLITKYMEPASANLSLRWSGKSYDI
HEGEPAGTTSLVMDGRVQLEVSHSTLDAIGVTATDYAYLVLGKNVKSQQQV
IALAPKSDSVVRVFDSWTVPYSMNEDDALRLLPVIRTNLIALSVISRLSSGEML
VLVEPEDAFAQTLSRLAAERAISVVTLTARTDIKNSNWTYLHANSPKRLVRST
ISRNASWVVADHSQGGLAANVLECLPANCKVLSAESLTSKKPKLDTFSSMAF
IPSILRTAFVRAHDLKTTLEPPSVVAAADISPENQPSSEAIFFSWAASPRVPVQV
TPVDHGKLFSSEKTYWLVGLSGGLGLSLCDWMVKHGAKYVVITSRNPQVDT
RWEQHMKAQGAVVRIYANDITDRESVASVCKKIRAELPPVGGIAQGAMVLA

DTMFVDMDLPRVQKVVGPKVNGSIHLHEMFADVDLEFFVFFSSMAYVTGNQ
GQSIYAAANAYMTALAAQRRKRGLAGSAINIGTIIGNGYVTRQLTVAQQEYL
THMGNVFMSEQDFHQIFAEAVVAGRPTSNDIPEIMTGLRLAHLDDSDKVTWF
HNPKFSHCVLWPEEQGGKTVMSKQNVTVRAQLLLATTADEAREIIEESLAAK
LRSSLQIDATVSVINMNADQLGLDSLVAVDIRSWFIKELNVEMPVLKILGGYT
VAEMVAAAQEKFSPSLIPNMGKEVDPSLKAVAKAQVEEPVAVPEEKPITTEK
DEYADFDEENNEADIPTEDSLPEITVSDGSPDLSDREPGKFNFNASGFKKVGFS
PGPQTPMSEDDRSKWSSHGSPFDSDSDNASIRKSRTSAATSVAALDEYFSKPD
HTIFERTLPMSFGQTRFWFLKFYMEDQTTFNITTSIRLAGRLDVGRFSRAVHH
LGRRHEALRTAFFTDSNNQPMQAVLKEPVLRLEHARGEANVASEYRRIMNH
QYDIGRGETMKITLLSLSEKLHQLIIGYHHINMDGISLEVIIRDLQHLYDGKSLA
PVSIQYPDFSIMQYKEHSGGQWDDELAFWKNEFADIPEPLPILPPSTKAVRTPL
STYSSNTVKFEVGAELSGQIENACKRTKTSPFNFYLATFKVLLYRLAEGKATD
ICIGMADGGRNNDLVSQSVGFFLNLLPLRFKQQPSQVFSDALKESRSKVITAL
ANSKVPFDVFLNEVNAPRTATLSPLFQAFINYRQGVQEKRQFCGCESEATQFD
GSQTAYDLSLDILGNPGSGIVYLAGQSSLYSQSDVETIAQSYYALLKAFAKNP
ALRINRPSLYNPQAVEHALAIGKGPTNVGTWPETLVHRVDEIVKAHGSKVAL
KSANSKLTYTQMAGRVNAIASTLQSNGLSKVSRVGVFQDPSTDFFCTILAVLR
IGAVFVPLEPRLTAPRLATMVQDSGLNAIVYDKANQKALADLGSNSKKINVS
LVMAKSSTVVPNQATPDATAIILYTSGSTGKPKGILLSHSAWRNQIEASSQAW
GVQTGTGVHLQQSSWSFDISISQTFVALANGASLFITPKTMRGDSSAITKTIVS
DQVTHVQATPSELSSWLRFSDLAALRASKWHFAMTGGERMTPALIDGFRKL
AKTDLKLFNAYGPAETTLAVGSSEVNYTADDNLDTPFTVFPNYSVYILDGQK
QPVPAGIPGEVYIGGAGVAQGYLNQDMLTAERFLQDVFASTEYTQNKWTKM
HRSGDRGHLSEDGHLVLEGRIDGDTQIKLRGIRIDLQDIESAMVQQANGVLTE
AVVSVRKSEDTEFLVAHVVTSPTFSGNTQSYLDQLRTSLPIPQYMQPAIAVTL
DALPVNHSGKVDRKAIAALPILPTTTQSNASPQPRDSAEKLKEIWTQVLGQGI
TSLHHIDAQSDFFHVGGSSLALVEVQAKIKTIFQTEVSLVQLFENPTLGAMTR
MVDPAAFSAPVNVNVAIPSEVATAPPAFTTSVNTGPKQIDWEEETALTDDFY
DIEIDPTPKDQGLPYKTVVITGATGFLGKALLRRMLDDNHIDKIHAITLRRSRS
DLPAIFSDPKVHLHRGDLNAPRLGLSETAAAEIFAETDAVIHNGADVSFMKTY
RTLSKTNVGSTRELVKLCLPHRIPIHYVSSASVVHLSGLESYGEASVSSFEPPQ
DGTDGYTASKWASERFLERVSEKFSVPIWIHRPSSITGEDAPTLDLMTNMLSF
SKKLRKAPTSPAWQGTLDFVDVEKVATEIVEEVKNDSAHPGGLVKYMYESG
DLEIAVGDMKGSLERETGQTFQTLNLEEWTKAAAEEGLEPLVAAYLAGAGE
MPIVFPKLVRGPRRPVVVSSVSSAPAASSSFSLKGVVGRWFFS

AspoE gDNA nucleotide sequence

ATGGCCACCACCTCTGCCACCTCTACCACCAAGGGGCACAACCAACCTCC
CCGTGAGCCCATCGCCATCGTCGGCTCAGCCTGTCGTTTCCCCGGCGGCGC
CAGCTCGCCCTCGAAACTATGGAAGCTCCTCGAACAGCCCCGAGACGTGC
TGAAGGAGATTCCCCCGGACCGGTTCAGCGTCGACGGCTTCTACCACCCA

GACAACATGCACCATGGCACCTCCAACGTACGACATTCGTACATCCTCGA
CGACGACATCCGTGTCTTCGATGCCCAGTTCTTCGGGATCAAGCCCGTTGA
GGCCAACTCCATCGACCCCCAACAGCGCCTCCTCATGGAGACCGTCTACG
AGGGCATCGAGTCGGCAGGCCTACAACTCAACCAGATGAAAGGGTCACA
GACAGGTGTGTATGTCGGGCTCATGTCGAATGACTACGCAGATATGCTGG
GAAACGACCAGGAGAGCTTCCCCACGTATTTCGCCACGGGCACGGCTCGG
TCAATCGTGTCGAACCGCGTGTCGTACTTTTTCGACTGGCATGGTCCGTCT
ATGACTATCGATACTGCCTGTTCCTCGAGTCTGGTTGCCATGCATCAGGCT
GTGCAGTACTTACGGTCTGGAGATGGATCTGATGTCGCCATCGCTGCGGG
AACCAACATTCTCCTCAACCCTGACCAGTACATTGCGGAGTCGAAGCTGA
AAATGCTGAGTCCCGATGGCAGGAGTCAGATGTGGGATGAGAAGGCGAA
TGGGTATGCCCGCGGCGATGGTATTGCGGTTGTCGTGTTGAAGACGCTGT
CACAGGCACTCCGGGATGGAGATCACATTGAGTGTATTGTGCGTGAGACA
CATATCAACCAGGATGGTAAGACGAAGGGGATTACCATGCCGAGTGCGA
CGGCACAAACCGCCCTGATTCGGGCGACGTATAAGAATGCTGGGCTGGAT
ATCACGAAGCCGAGTGATCGGCCCCAGTTCTTCGAGGCCCATGGACAGG
AACACCGGTAGGACTCCCCGGATTGACTTTATTTGTGGCGTGTGGTCTTTA
TCTAACCATTTTATTCATTCTAGGCCGGCGATCCCATCGAAGCAGAGGCTA
TTCATACGGCATTCTTCGGCTACAAGGGACTGAGCAAAGAGATCGATGCA
CTGAGCGTTGGCTCTATCAAGACGATCATCGGTCACACAGAAGGGACGGC
CGGGCTAGCTGCTGTCCTGAAGGCGAGTCTTGCCTTGCAGGCGGGTGTGA
TTCCACCGAATCTACTCTTCGACAAGGTGAACCCCAAGGTCAAGCCGTTCT
ATGGAAACCTACAGATTCAGACACAGTCAAAGCCATGGCCTGAGATTGCT
CCCGGTGCTGTACGTCGTGCAAGTGTCAACAGCTTTGGCTTTGGTGGTGCA
AATGCCCATGCCATTCTGGAGGCGTATGAGCCATCTTCTACGCCCACAGA
GGACACCTCTGCGAACGTATTTACTGCACTGAATGTGTCTGCAATGTCGG
AAACCGCCCTGCGGAGAACGCTCAAGAAGTATGCCGAGTATCTCGAAGA
GAACCCCTCCGTCGACCTGCGCAGCCTGGCCTTGACAGTGAATACCCGCC
GCTCGACATTCCCAGTGAGAACTTCTGTATTCGGATCAACTATGGAAGAG
CTGTCCCAGAGACTACGCGAACGATCCGAGGCAGAAGGCAAGACGCTCA
CACCTGTGGCGCCAACCTCGTTGGCGTCCAGTCCCAAGATCTTCGGAGTCT
TCACCGGTCAAGGTGCGCAATGGAAGCGAATGGGTGCTATACTTCTAGAT
ACCTCACCGAGAGTTGTCGCCATTCTCGATAAACTCGAGAAGAGTCTTGC
TGAGCTACCAGATGGACCTTCATGGTCAATCAAAGGCGAGATCCTAGCGG
ACGAGAACTCCCGTGTGAACGAAGCCGTGATCAGTCAGCCCCTCTGCACT
GCAGTCCAGATAGTCTTGGTTGATCTGCTTCGGAGCGCTGGTGTCAAGTTC
CACGCTGTCGTTGGTCACTCCTCCGGTGAGATTGGCGCTGCCTATGCGGCC
GGCTACCTATCTGCCAGTGACGCTATCCGCATCGCTTACTACCGTGGCCTG
CATCTGTGTCTGGCCCAGGGACCCAACGGCCAGCAAGGAGCCATGATGGC
GGTTGGAACATCCTTCGAAGACGCCCAAGAGCTCTGTGATCTACCCGCTTT
CAAGGATCGGATGAGTATTGCCGCTAGTAACTCGTCTGCGAGCGTTACGC
TATCCGGTGACCTGGATGCGATTGAATGGGCCAACGATGTCTTCGATGAC

GAGAAGAAATTCGCCCGTCTCTTGAAGGTCGACAAGGCTTACCACTCACA
CCACATGCTGGCCTGCTCTGACGCCTATCGGAAATCCATGACTGACTGTG
GCATCACTGTTCTCCAGCCTGAGCGCAACGGCACCACGTGGATTTCTAGT
GTGTATGGTGAGGATGCATTGGACTATCGCCATGAGATGAGCGCCGAGTA
CTGGATCAGCAACATGGTTAGCCCTGTGCTGTTCTCGCAGGCGGTGGAGT
TTGCGCTGGCTGATCAGGGGCCATTCGATATTGGCATTGAATGTGGTCCTC
ACCCTGCCCTCAAGGGCCCAGCACTACAGGTTATCCAAGAGATGCTTGGC
AGTTCGATTCCCTATACAGGACTTCTGAGCCGTGGACGACCAGACAATCA
AGCGCTTGCGGAAGGTATCAGCTATCTCTGGCAGGCCTTGGGCGAGGGGA
TCGTCGACTATAGGTTGTTTGACAAGTTCATCGCCGGGCCTGATGCACCGG
AGCCTCAGATCGTGGCGAATCTGCCATCATATGCCTGGGACCATGATCGT
GCTTTCTGGCATGAGTCGCGACAATACTGGGCCAATCGCACCAAGGAAGA
TCCACCTCATGAGATTCTGGGAACCAAATGTCCCGATGGAACGAATCAGC
AGCACCGGTGGAGGAACATGCTTCGGCCCCGGGAGATTCCCTGGATTGCT
GGTCATCAAATCCAGGAGCAAATGGTCTTCCCAGCAGCTGGATACGTATC
TGCTGCCCTTGAAGCGGTCAAGTTGGTCACTCGAGGACAGTCCATTGGAG
CAATCGAGATTGAGGATTTCATCATCGGACAAGCCATTGCCTTTAACGAT
GAGTACGCGAGTGTCGAGACCCAGTTCACACTGACCGATATTTCCGTGGA
GAAGGACATCTGGTCTGCTTCTTTCTCCTTTTACTCTGCGTCTCCAAAAAG
CTCCCGTTCTTTGGATCTGAATGCCTCGGGAAAGCTTAGAGTCACACTCGG
CGAGCCACAAGATGACTTGCTCCCGCCTCATCTCAGCCCAGAGTTCAACA
TGATTGATGTCGACTCTGAGCGGTTTTACGATGCTTTGAAGAAGCTTGGTT
TTGGATATACCGGCCCCTTCAAGGCCCTGGGCTCTCTCAAGCGCAGAATG
GGGGTGGCGACTGGAACGATCGCGAATCCCACGAGTACTGACCCAGCCCA
TGATCTTCTACTGCACCCCGCCACATTCGACAATGCCATTCAGTCTATCAT
CTTGGCATACTGTTATCCCAATGACGGTCGCTTGTGGTCTGTGCAGCTGCC
TACTGGCATCAAGAAAATCAAGATCAACCCTGTCCTGTGCAACCAGTATG
CTGGCAAGAACGCATTGCTCTGCTTCAAGGCATCGACGAGCGATGATAGA
TCTGCCGAGATCGGTGGAGATGTCGACATCTACGATGAGCAGGGCAACAA
CGCTCTTATGCAGCTTGAAGGGCTACAGACCAAGCCTCTTGCAAATGCAA
CCGCGGCCAATGACTCTCCTCTGTTCCTCGAGACGATATGGGATATCGAG
GGCCCGAGTAGGGAGGCAGCTGTTGCGGATCGTCCGGACATGCAGCTAAA
GACAGAGCTCGCCTTCGATGTCGAACGTGTTGCCTTCTTCTATCTCCGTTA
TCTGGACTCCGTTGCGACGAGAGAAGAGCGGGAGAAGGCAGAGTCTCAC
CACAAGATATTTTTCGAGTACATTGACCACACTGTTGCAAATGTCAAGAG
CGGCACCGCCCAGTTCGCCAAACGCGAGTGGATGTACGATACTCATGATG
AGATATTGGACATTATTGGCAAGTATCCTGACAGTTTGGACATGAAGCTC
ATGCACGCCGTTGGTGAGCATCTGCTGCCTGTGATCCGCGGAGAGACAAC
CATGCTGGAGTACATGCGAGAGGACAACCTTTTGAACGACTTCTATGTTC
ACGCTATCGGGTTCGATGAGTATACGGAGAACCTCGCCCAACAGGTCAGC
CAGTTTAGCCACCGCTATCCCCACATGAACATCTTGGAAATCGGGGCGGG
TACCGGTGGTGCGACGAAGAGAATCTTCTCCAAGCTGGGCAAGCGATTCG

GCTCGTACACATATACCGATATCTCGGCTGGTTTCTTCGAGAAGACACGC
GAGACCTTCCGGGAGTATGAGCACATGATGACTTTCAAGGCGTTGAACAT
CGAGAAAGATCCGGTCGAGCAGGGCTTCACGGAACAGTCGTATGACTTGG
TCGTTGCTTCGCTTGTGTTGCATGCTACTCATGAGATGGAGACTACCATGC
GGAATGTTCGTCGCCTGCTCAAGCCTGGTGGGTATCTCATCATGCTCGAGC
TAGGTGACTATATTGAGATGCGAACTGGGCTTATCTTCGGCTCCCTCCCCG
GATGGTGGATGGGCTACGATGATGGACGCAAGCTGTCGCCTTGTATGTCT
GAAGAGGACTGGAGTACATGCATGCAGAAGACAGGTTTCTCCGGTGTTGA
TGCTATTGTCCCTCGACAGTCTGAGCTTCCCATATCCCTAGCTGTACTCAC
AGGACAGGCTGTGGATGATCATGTCAACTTCCTCCGCGATCCATTGACAC
CGGGGTCGGTCAACTTTGTCGAGTCCAATCTGACTATCATCGGTGGCACC
ACCTCAAAGATCTCTGGGGTTGTCGAAGAAGCCGCGAAGTCTCTCAACAG
ATTTTACGAGAAGATTGTGACAGCAAGATCCCTTGCTGAAATGGACACAG
CCCAAGTCCCGTTCATGGGATCGGCGTTGTTCCTCACGGATTTGGATGAGC
CAATTTTCGAGAATGTCACCGAGGAAGCCCTCACAGCACTCAAGCAGCTG
TTCAAGCAATCCCGAACCTGCCTCTGGGTTACCCAAGGCGCTAGAGATGA
CAACCCCTTCCAGAACATGTCTGTCGGACTTGGGCGGGTTGTGAAGCTGG
AAATGACACACTTACGTCTACAGTCTCTCGACTTTGACATTGACACGGAA
CCATCGGCAGCAGCTATCATGCAAAGACTGCTGCAATTTGAAGCCATGGC
TCAGTGGGAACAGAGCGGCGAATCGAAGGACTTGCTTTGGTCTGTGGAGC
CTGAGATCGGGTATGACCACAGCAAGGCGATTATTCCTCGATTGATGCCG
AACCCGGTTCGCAATGCTCGGTATAACTCGTCAAGGCGTCTAATCACCAA
GTACATGGAGCCTGCATCTGCTAACCTTAGTCTTCGCTGGTCTGGTAAGTC
TTACGACATTCATGAGGGAGAACCTGCAGGTACTACCTCCCTCGTCATGG
ATGGAAGAGTGCAGCTGGAAGTCAGCCACTCCACATTGGATGCTATCGGA
GTTACTGCGACAGATTATGCATATCTAGTTCTGGGAAAGAACGTTAAATCT
CAGCAGCAAGTTATTGCGCTCGCGCCGAAGAGTGACTCGGTCGTTCGAGT
GTTTGATTCGTGGACTGTCCCCTACAGCATGAACGAGGATGATGCCTTAC
GACTCCTTCCTGTGATTCGCACGAACCTGATTGCGCTTTCGGTTATCTCCA
GGCTGTCAAGTGGCGAAATGTTGGTCCTGGTCGAGCCAGAGGATGCATTT
GCCCAAACATTGTCTCGTCTGGCAGCTGAACGGGCGATCTCGGTGGTGAC
TCTCACGGCAAGAACAGACATCAAGAACAGCAACTGGACTTATTTGCACG
CCAACTCACCCAAACGACTTGTGCGGTCTACTATCTCTAGAAATGCTTCGT
GGGTGGTTGCAGACCACAGCCAGGGAGGTCTGGCAGCTAATGTGCTGGAG
TGTCTGCCCGCAAACTGCAAAGTTCTGTCGGCTGAATCACTCACCTCCAAG
AAGCCCAAGCTGGATACCTTCTCCTCGATGGCCTTCATTCCTTCAATTCTT
CGGACTGCCTTTGTTCGTGCTCATGATCTCAAGACTACCCTGGAACCCCCA
TCTGTGGTTGCTGCGGCCGATATTTCCCCCGAGAATCAGCCTTCTTCTGAA
GCAATCTTCTTCAGCTGGGCCGCATCTCCCAGGGTCCCTGTGCAGGTGACA
CCAGTTGACCACGGTAAACTATTCTCCTCGGAGAAGACCTACTGGCTGGT
CGGTCTCAGTGGAGGTCTCGGTCTCTCCTTGTGCGACTGGATGGTCAAGCA
TGGCGCGAAGTACGTCGTCATCACCAGTCGCAATCCACAGGTGGATACCA

GGTGGGAGCAGCATATGAAAGCACAGGGTGCTGTGGTCAGAATCTACGC
AAACGATATCACGGACCGAGAGTCGGTCGCTAGTGTCTGCAAGAAGATCC
GCGCTGAGCTTCCTCCCGTCGGCGGTATTGCTCAGGGTGCAATGGTCCTCG
CCGATACCATGTTCGTGGATATGGATCTACCCAGGGTCCAGAAGGTGGTG
GGACCGAAGGTCAACGGGTCCATCCATCTTCATGAGATGTTCGCCGACGT
CGACCTGGAGTTCTTCGTTTTCTTCTCGTCCATGGCCTATGTCACAGGTAA
CCAGGGCCAGTCGATATATGCTGCCGCCAACGCGTATATGACAGCACTTG
CTGCGCAGCGTAGGAAGCGAGGACTCGCTGGCTCTGCCATCAATATTGGA
ACTATCATTGGCAACGGCTACGTGACTCGTCAGCTTACAGTTGCTCAACA
AGAATACCTCACCCACATGGGCAACGTCTTCATGTCGGAGCAGGACTTCC
ACCAGATCTTCGCGGAAGCTGTGGTCGCTGGTCGTCCGACTTCAAACGAC
ATTCCGGAGATTATGACCGGGTTGCGTCTTGCCCATCTGGACGACTCCGAC
AAGGTGACCTGGTTCCATAATCCAAAGTTCTCTCACTGTGTCCTCTGGCCT
GAGGAGCAGGGAGGCAAGACTGTCATGTCGAAGCAGAATGTCACTGTTC
GGGCGCAGTTGCTCCTTGCTACCACGGCTGATGAAGCTAGAGAGATTATT
GAAGAGAGTCTGGCTGCCAAACTGAGGTCTTCTCTTCAAATCGACGCAAC
AGTCTCTGTTATCAACATGAATGCCGACCAGTTGGGCTTGGACTCCCTTGT
CGCTGTCGATATACGCAGTTGGTTCATCAAGGAGCTGAATGTTGAGATGC
CTGTGCTTAAGATCCTTGGTGGCTATACAGTGGCCGAGATGGTTGCTGCA
GCCCAGGAAAAGTTTTCCCCATCTCTGATTCCCAACATGGGCAAGGAAGT
CGATCCCTCGCTCAAGGCTGTGGCGAAAGCACAAGTCGAAGAGCCCGTTG
CAGTGCCAGAGGAGAAGCCCATCACGACTGAGAAAGACGAATACGCTGA
TTTTGATGAGGAGAACAACGAGGCAGACATTCCCACCGAAGATTCCCTGC
CAGAGATCACCGTGTCTGATGGGTCTCCCGATCTCTCTGACCGAGAGCCT
GGCAAGTTCAACTTCAACGCATCCGGATTCAAGAAAGTCGGGTTCAGTCC
CGGCCCTCAAACACCAATGAGTGAGGATGACCGAAGCAAATGGAGCAGC
CATGGAAGCCCCTTCGATTCTGACTCTGACAATGCTTCTATCCGCAAGTCT
CGTACATCTGCTGCTACCTCCGTCGCTGCGCTCGACGAGTACTTCTCCAAG
CCAGATCATACCATCTTTGAACGGACGCTGCCCATGTCCTTCGGGCAAAC
GCGATTCTGGTTCTTGAAGTTTTACATGGAGGACCAAACCACTTTCAACAT
TACGACTTCTATCCGTTTGGCTGGTAGGTTGGATGTTGGGCGGTTCTCACG
AGCTGTGCATCATCTCGGAAGACGACATGAAGCGTTGCGCACGGCATTCT
TCACCGACAGCAACAATCAGCCTATGCAGGCGGTGCTCAAGGAGCCTGTG
CTTCGATTGGAGCACGCCCGCGGTGAGGCTAACGTTGCTAGCGAGTATCG
CAGGATCATGAACCACCAGTACGATATCGGTCGTGGTGAGACAATGAAGA
TTACGCTACTATCGCTTTCCGAGAAGCTTCACCAGCTGATTATTGGGTACC
ACCATATCAACATGGATGGCATCAGTCTGGAAGTTATCATCAGGGATCTG
CAGCATTTGTATGATGGCAAATCCCTCGCGCCGGTTTCCATCCAATATCCG
GACTTCTCCATCATGCAGTACAAGGAGCACTCTGGTGGACAATGGGACGA
TGAACTCGCCTTCTGGAAGAACGAGTTCGCAGATATTCCCGAGCCGCTTC
CCATCTTGCCGCCGTCGACGAAGGCTGTTCGTACGCCATTGTCCACCTACA
GCAGCAACACCGTCAAATTTGAAGTTGGCGCCGAGCTATCCGGCCAGATA

GAGAATGCTTGCAAGAGAACTAAGACAAGCCCCTTCAACTTTTACCTTGC
CACATTCAAGGTCCTTCTTTACCGCCTGGCGGAAGGCAAAGCTACTGATA
TCTGCATTGGAATGGCAGATGGAGGACGCAACAATGACCTAGTCTCGCAG
AGTGTTGGTTTCTTCCTGAACCTACTGCCGCTTCGCTTCAAGCAGCAGCCC
TCGCAGGTGTTCAGCGATGCTCTGAAAGAGTCCCGTTCCAAGGTTATCACT
GCCCTTGCCAACAGCAAGGTTCCGTTTGATGTGTTCCTCAACGAAGTCAAT
GCGCCCAGAACAGCCACTCTCAGCCCGCTCTTCCAAGCGTTCATCAACTA
CCGACAGGGTGTCCAGGAGAAGCGTCAGTTCTGTGGCTGCGAAAGTGAGG
CTACTCAGTTTGATGGCAGCCAGACGGCCTACGATCTCAGTTTGGACATTC
TCGGAAACCCAGGCAGTGGAATCGTATACCTCGCTGGTCAGAGTTCACTG
TACAGTCAGTCAGACGTGGAGACAATTGCCCAGAGTTACTACGCCCTTCT
CAAGGCATTCGCAAAGAATCCAGCTCTTCGCATCAACCGTCCCTCGCTGT
ACAATCCCCAGGCTGTTGAACATGCATTGGCCATAGGTAAGGGTCCGACA
AATGTTGGTACCTGGCCGGAGACCCTGGTCCATCGAGTCGACGAGATTGT
CAAAGCCCATGGCTCCAAGGTGGCCTTGAAGAGCGCTAATTCAAAGTTGA
CCTACACTCAAATGGCTGGGCGGGTCAATGCTATTGCATCGACTTTGCAGT
CCAATGGACTGAGCAAGGTTTCGCGGGTTGGGGTCTTCCAGGATCCCTCT
ACCGACTTCTTCTGCACTATTCTGGCAGTCCTTCGCATTGGGGCCGTGTTT
GTCCCGCTTGAACCTCGTCTCACTGCTCCCCGTCTTGCTACGATGGTACAG
GACAGCGGTTTGAATGCGATCGTCTATGATAAAGCAAACCAGAAGGCCTT
GGCTGACCTAGGATCTAATTCGAAGAAGATCAATGTCTCCTTGGTCATGG
CAAAGAGCTCTACCGTTGTCCCCAACCAGGCCACGCCTGATGCAACCGCG
ATTATCTTGTACACCAGTGGCTCGACTGGAAAGCCCAAGGGCATTCTCCT
GAGCCATTCGGCATGGCGAAACCAAATCGAGGCATCCTCGCAGGCATGGG
GGGTACAGACTGGAACGGGAGTCCATCTCCAGCAGAGCTCGTGGAGTTTC
GATATCTCCATCTCTCAGACCTTTGTTGCCTTGGCCAACGGTGCTTCCCTG
TTTATTACTCCTAAGACTATGCGCGGGGACTCGTCTGCAATCACCAAGAC
GATTGTGTCCGATCAAGTCACCCACGTTCAGGCCACCCCGTCAGAGCTCA
GCAGCTGGCTCCGGTTTAGTGACCTTGCAGCTCTTCGCGCCTCCAAATGGC
ATTTCGCAATGACAGGCGGCGAGCGAATGACTCCCGCGTTGATTGATGGT
TTCCGAAAATTGGCGAAGACTGATCTTAAGCTCTTCAATGCCTACGGTCCT
GCCGAGACTACCCTTGCAGTGGGCAGTTCTGAAGTCAACTACACGGCCGA
CGACAACCTTGATACGCCTTTCACCGTTTTCCCTAACTACTCCGTGTATAT
TCTGGATGGTCAGAAGCAGCCGGTTCCTGCTGGCATTCCTGGAGAAGTTT
ACATCGGTGGAGCTGGTGTTGCTCAGGGCTACCTCAATCAGGATATGCTC
ACTGCCGAAAGATTCCTCCAAGATGTGTTTGCTTCCACTGAGTACACCCAG
AACAAGTGGACTAAGATGCACCGAAGCGGTGATCGTGGCCATCTCTCCGA
GGACGGACATCTTGTCTTGGAGGGTAGAATCGACGGTGACACTCAGATCA
AACTGCGCGGCATCCGCATCGACCTCCAGGATATTGAATCGGCTATGGTA
CAGCAAGCAAATGGTGTACTCACTGAAGCTGTCGTCTCCGTCCGCAAGTC
TGAAGACACCGAATTCCTTGTCGCTCATGTTGTCACATCTCCTACATTCAG
CGGCAACACCCAGTCCTACCTGGATCAGCTTCGCACATCATTGCCTATCCC

GCAGTACATGCAGCCTGCAATTGCAGTTACCTTGGATGCCCTGCCGGTGA
ATCACTCTGGAAAGGTTGACCGCAAAGCCATAGCAGCACTCCCGATTCTG
CCAACTACGACGCAGTCAAATGCTAGCCCACAGCCCAGAGACTCTGCTGA
GAAACTGAAGGAAATTTGGACCCAAGTTCTTGGTCAAGGAATCACGTCGC
TTCACCATATTGACGCTCAGTCTGATTTCTTCCATGTTGGCGGCAGCTCTTT
GGCGTTGGTTGAAGTACAGGCCAAGATCAAGACCATCTTCCAGACTGAAG
TCTCCCTAGTCCAGCTCTTCGAGAACCCTACTCTCGGCGCGATGACTCGCA
TGGTGGATCCCGCTGCATTCTCTGCTCCTGTGAATGTCAATGTCGCCATTC
CTTCCGAGGTCGCGACAGCACCCCCTGCATTCACCACTTCGGTTAACACCG
GCCCCAAGCAGATTGACTGGGAAGAAGAAACCGCATTGACCGACGACTTC
TACGATATTGAGATTGACCCCACTCCCAAGGACCAAGGTCTTCCCTACAA
GACAGTCGTCATCACCGGTGCCACAGGCTTCCTCGGTAAAGCGCTTCTGC
GCCGAATGCTGGACGACAACCACATCGACAAGATTCACGCCATCACCCTT
CGCCGCTCCCGCTCTGATCTACCAGCCATTTTCTCCGATCCCAAGGTGCAC
CTTCACCGGGGTGACCTGAATGCGCCTCGTCTCGGTTTGTCGGAGACTGCT
GCGGCGGAAATCTTCGCTGAAACAGATGCCGTGATTCACAATGGTGCTGA
CGTCTCCTTCATGAAGACCTACCGGACTCTGTCCAAGACCAATGTTGGTAG
CACCCGTGAGTTGGTGAAGTTGTGTCTCCCTCACCGCATTCCCATTCATTA
CGTCTCCTCTGCTAGTGTGGTTCATCTTTCCGGTCTGGAATCTTACGGAGA
GGCCTCTGTGTCCTCCTTCGAGCCACCGCAGGATGGAACAGATGGCTACA
CGGCAAGCAAATGGGCCAGCGAGCGCTTCCTAGAGCGCGTGAGTGAGAA
ATTCTCCGTTCCGATATGGATCCACCGCCCCAGCAGCATCACCGGCGAGG
ATGCACCCACCCTGGACCTGATGACAAACATGCTGAGTTTCTCGAAGAAA
CTGCGCAAAGCACCCACTTCACCTGCTTGGCAGGGTACCCTGGACTTTGTG
GATGTAGAGAAGGTCGCAACAGAAATTGTTGAGGAGGTGAAGAATGACA
GTGCCCACCCCGGTGGACTGGTGAAGTACATGTATGAGAGTGGGGATTTG
GAGATTGCCGTTGGTGATATGAAGGGTTCGTTGGAGCGGGAGACTGGTCA
GACTTTCCAGACATTGAATCTGGAGGAATGGACCAAGGCTGCAGCTGAGG
AAGGGTTGGAACCGCTGGTTGCAGCATATTTGGCTGGTGCTGGAGAGATG
CCTATCGTGTTCCCGAAGTTGGTCAGAGGACCTAGACGTCCGGTTGTTGTG
TCGTCTGTTTCTTCGGCGCCGGCGGCTTCGTCATCGTTCTCGTTGAAGGGT
GTCGTTGGAAGATGGTTCTTCTCATAG

AspoF amino acid sequence

MLQDIVEQWQIMQQALAPLRLSRWQLTKMFAAQVYRDHPVGALLGISLSVV
LLLWVVSVVTRPKKLEDVLGLPVLGGSRTLKSDFLRIIEEGKQRYPDTPYIVN
ASGLQYVVYPPIFFDEIKRLTEQEASAQDFFHTVTYGQWTHIGAETDALWKTI
AVDLARSVPVKVPSKQKDARIAFDKYVGYCPESKSVTIFDTMMKIVATTNAC
SFVGREVGTGEWPQVVQQLPMSVYFAVMTLSIVPRIFRPVLLPIVLIPALLVQR
KMRKILEPSIRKDMEEYERAADRKELLKPTEDGKLPFTQWLIARYKPGEATA
HQLATDHLLTSFESTVSTAATLYNIILDLAVRPELQDELRQEVEEIMVDGKLP
ATHLKELKKMDSMMRETFRVNPFALFSLYRITRKPIQLSSGPKLPAGTILCVD

SHHINNSASLFPEPTKFDPYRFLKKREEPGAENRFQFVSTGPTDPNWGDGTQA
CPGRFFANSTLKVCLAHVLLKYNVSLREGQERPKMVSMPNGIWAPDMAAQV
LFQSRD

AspoF gDNA nucleotide sequence
ATGTTGCAAGATATCGTCGAGCAATGGCAAATCATGCAGCAGGCACTGGC
GCCGCTGCGTTTGTCCCGTTGGCAGTTGACCAAGATGTTTGCTGCGCAAGT
ATATCGTGACCACCCAGTGGGCGCGCTGCTAGGCATCTCACTCAGCGTGG
TACTCCTGCTTTGGGTGGTGTCCGTGGTGACTCGACCCAAGAAACTGGAG
GATGTCCTTGGCCTGCCGGTCCTCGGCGGGTCGAGAACGCTGAAGAGTGA
CTTCTTGCGCATCATCGAGGAAGGCAAGCAGAGGGTAGGCTCTATATCAT
CCAATATATACCCAAAGGTGACCCATACTAACATGGATACAGTACCCAGA
CACTCCATACATTGTCAATGCATCTGGTCTACAATACGTAGTCTACCCGCC
CATCTTCTTCGACGAGATCAAGCGTCTCACCGAGCAAGAAGCCTCAGCGC
AGGACTTCTTCCACACGGTCACCTACGGGCAATGGACGCACATCGGCGCC
GAAACCGACGCGCTATGGAAGACCATCGCTGTCGACCTGGCCCGTAGTGT
ACCCGTCAAAGTCCCGTCGAAACAGAAAGACGCCCGTATCGCCTTCGACA
AGTACGTCGGATACTGCCCCGAGTCCAAATCCGTGACCATCTTCGACACG
ATGATGAAGATCGTCGCCACCACCAACGCCTGCTCGTTCGTCGGCCGTGA
AGTCGGCACTGGCGAGTGGCCGCAAGTCGTCCAGCAACTACCCATGTCTG
TGTACTTTGCCGTCATGACGCTGAGTATCGTCCCGCGCATTTTCCGCCCTG
TTCTCCTGCCCATCGTGCTCATCCCAGCGTTGCTGGTGCAGCGGAAGATGC
GGAAGATTCTCGAGCCTAGCATTCGGAAAGATATGGAGGAATATGAGCGC
GCTGCTGACCGGAAGGAGCTGCTTAAGCCGACAGAGGATGGCAAATTGCC
GTTTACACAGTGGCTCATAGCGCGGTATAAGCCTGGTGAGGCGACGGCGC
ATCAGTTGGCAACGGATCATTTGCTTACCAGCTTTGAGTCGACTGTGAGCA
CTGCCGCAACGTTGTATAACATAATTTTGGATCTGGCTGTTCGCCCTGAAT
TACAGGACGAACTCCGCCAGGAAGTAGAGGAGATTATGGTGGATGGGAA
GCTTCCCGCAACGCATCTGAAGGAGTTGAAGAAGATGGATAGTATGATGC
GGGAGACGTTCCGGGTGAATCCTTTTGCTCTATGTAAGTTTGGACCATGGC
TTTCTGAGGTACACTCGATATGTTCTAACAAATACTTCTAGTCTCTCTCTAT
CGCATCACTCGCAAGCCTATCCAACTCTCCTCTGGCCCGAAGCTCCCAGCA
GGAACCATCCTCTGTGTTGATTCCCACCACATCAACAATTCTGCTTCCTTG
TTCCCTGAGCCCACCAAATTCGACCCCTACCGGTTCCTGAAGAAGCGCGA
GGAGCCAGGCGCAGAGAATCGCTTCCAGTTTGTCTCAACTGGCCCTACCG
ATCCCAACTGGGGTGATGGTACCCAGGCTTGCCCCGGGCGTTTCTTTGCAA
ACAGTACTTTAAAAGTGTGCCTTGCTCATGTACTCTTGAAGTATAATGTCT
CTCTCCGGGAGGGTCAAGAACGCCCCAAGATGGTCAGCATGCCGAACGG
AATTTGGGCACCGGACATGGCTGCGCAGGTGTTGTTCCAGTCGCGGGATT
AG

AspoH amino acid sequence

MSPARTLESSPLPTTQTAIIQGEGGILSIQHNAPLPTLRPDRILVKVAYVAINPC
DWKMADRFPTPGCVDGCDFSGTVVALGSDWSKTGRFKIGDRVCGGVHGSN
PIDQSTGCFADYVSADAQFTFHVPEYMGMEDAAAVGGTGIGTLGLALKRSLG
LPGSPRDPIPEGEGVQVLVYAASTSVGTLATQLLRMSGHKPIGVCSAKNFDM
VKSYGAVKLFDYHSPTCAQDIRAYTKNTLAHILDPITEPKTTELCYAAMGRA
GGKYCALEAFAEEFCTRRVVKPELVMGMAILGGRIALDYGYESEADPEKRVF
GVSWYEEMQELLDSGRLRNHPVRSFPGGFEGIMKGLHLLKTKQVSGEKLIVQ
LG

<u>AspoH gDNA nucleotide sequence</u>

ATGTCCCCCGCTCGCACATTAGAATCTTCTCCCCTCCCCACTACTCAAACC
GCCATCATCCAGGGCGAAGGCGGCATCCTCTCCATCCAACACAATGCTCC
CCTCCCTACCCTCCGGCCTGACCGCATCCTCGTGAAGGTCGCATACGTAGC
CATCAACCCCTGTGACTGGAAGATGGCGGACCGGTTCCCGACCCCGGGCT
GTGTCGACGGGTGCGATTTCTCCGGCACCGTCGTCGCCCTAGGCAGTGAC
TGGTCCAAGACGGGGCGATTCAAGATTGGAGATCGTGTCTGTGGCGGTGT
TCATGGCTCGAACCCTATCGACCAGAGCACGGGGTGTTTTGCGGATTATG
TCTCTGCTGACGCGCAGTTTACATTCCACGTCCCGGAGTATATGGGCATGG
AAGACGCGGCGGCTGTTGGGGGCACGGGGATAGGAACTCTCGGCTTGGC
GTTAAAGAGATCACTGGGGCTTCCTGGGAGTCCTAGAGACCCCATTCCTG
AGGGTGAAGGCGTCCAGGTGTTGGTTTATGCGGCTAGTACATCAGTGGGG
ACGCTGGCGACGCAGCTGCTGAGGATGTAAGTGGACGCAGAATCCGATCA
TCCTAGGAATGCTTGCCACAACGCAACGCAACATCTCGATTACCCTTTCTG
GAAAGCCATATGCTGATGTATGATGACAGGTCTGGTCACAAGCCCATCGG
CGTCTGCTCCGCCAAAAACTTCGACATGGTCAAGTCGTACGGCGCAGTCA
AGCTCTTCGACTACCACTCCCCGACTTGTGCACAGGACATCCGCGCTTACA
CGAAGAACACTCTCGCGCACATTCTCGACCCCATCACCGAGCCAAAGACC
ACCGAACTATGCTACGCCGCTATGGGCCGGGCCGGAGGTAAGTACTGCGC
GCTGGAGGCGTTCGCGGAGGAATTTTGCACACGCAGGGTGGTTAAACCCG
AGTTGGTCATGGGAATGGCGATTCTTGGGGGCCGAATTGCGCTCGACTAC
GGGTATGAGAGCGAGGCGGACCCGGAGAAACGGGTGTTTGGAGTGAGCT
GGTATGAAGAAATGCAGGAGTTGTTGGATTCGGGGCGGCTGCGGAACCAC
CCCGTGCGGTCTTTCCCGGGCGGCTTTGAGGGGATCATGAAGGGGCTACA
TTTGCTCAAAACAAAGCAGGTTTCGGGAGAAAAGCTGATTGTACAGTTGG
GATAA

Supplementary references

1. Lin, Z.J. *et al.* Spicochalasin A and New Aspochalasins from the Marine-Derived Fungus *Spicaria elegans. Eur. J. Org. Chem.* **2009**, 3045-3051 (2009)
2. Xu, W. *et al.* Analysis of Intact and Dissected Fungal Polyketide Synthase-Nonribosomal Peptide Synthetase in Vitro and in *Saccharomyces cerevisiae*. *J. Am. Chem. Soc.*, **132**, 13604–13607 (2010)
3. Fang, J. *et al*. Cloning and Characterization of the Tetrocarcin A Gene Cluster from *Micromonospora chalcea* NRRL 11289 Reveals A Highly Conserved Strategy for Tetronate Biosynthesis in Spirotetronate Antibiotics. *J. Bacteriol*. **190**, 6014-6025 (2008).
4. Zeng, H.C. *et al*. Unprecedented [5.5.5.6]Dioxafenestrane Ring Construction in Fungal Insecticidal Sesquiterpene Biosynthesis. *Angew. Chem. Int. Ed.*, **58**, 6569 –6573 (2019).
